# Targeting bacterial kinases as a strategy to counteract antibiotic resistance

**DOI:** 10.1038/s42004-025-01794-7

**Published:** 2025-12-04

**Authors:** Vanessa Buffa, Julien Kowalewski, Guoman Qi, Robin Deutscher, Matijas Cica, Marion Richardoz, Mathilde Tomaszczyk, Andreas Krämer, Stefan Knapp, Catherine Dunyach-Remy, Katharina Rox, Jean-Francois Guichou, Corinne Lionne, Felix Hausch

**Affiliations:** 1 Department of Chemistry, Institute of Organic Chemistry and Biochemistry, Technical University Darmstadt, Peter-Grünberg-Straße 4, Darmstadt, Germany; 2 Centre de Biologie Structurale (CBS), 29 rue de Navacelles, University of Montpellier, CNRS UMR 5048, INSERM U1054, Montpellier, France; 3 Virulence Bactérienne et Infections Chroniques, INSERM U1047, Department of Microbiology and Hospital Hygiene, Université de Montpellier, Nîmes, France; 4 Institute of Pharmaceutical Chemistry and Structural Genomics Consortium (SGC), Max von Lauestrasse 9, Frankfurt, am Main, Germany; 5 Department of Chemical Biology, Helmholtz Centre for Infection Research (HZI), Inhoffenstrasse 7, Braunschweig, Germany; 6 German Center for Infection Research (DZIF), partner site Hannover-Braunschweig, Inhoffenstrasse 7, Braunschweig, Germany; 7 Center for Synthetic Biology, Technical University, Darmstadt, Germany

**Keywords:** Structure-based drug design, Drug discovery and development, Chemical libraries, Kinases

## Abstract

Antibiotic resistance is rapidly emerging as one of the most critical health threats, with resistant microorganisms progressively diminishing the effectiveness of established antibiotics. As a result, the development of therapeutic approaches that effectively target resistant pathogens is of utmost importance. In this study, we developed inhibitors for APH(2”)-IVa, a bacterial kinase conveying resistance to aminoglycoside antibiotics. Starting from a hit of a fragment-based screening, we explored the inhibitory motif by structure-based design, ultimately leading to a series of triazole analogues. Advanced analogues displayed promising ADME properties, emerging selectivity *vs* a panel of human kinases, permeability in both Gram-positive and Gram-negative bacteria, and a moderate antibiotic efficacy for clinical strains of *P. aeruginosa*. Taken together, our results suggest inhibition of bacterial kinases could be a promising option to reinstall the efficacy of aminoglycoside antibiotics.

## Abstract (web summary)

Antibiotic resistance poses a significant threat to global health, necessitating new strategies to combat resistant pathogens. Here, the authors develop triazole analogues targeting the bacterial kinase APH(2”)-IVa, demonstrating promising selectivity, permeability, and moderate efficacy against *P. aeruginosa*, suggesting a potential pathway to restore the efficacy of aminoglycoside antibiotics.

## Introduction

Antibiotic resistance is one of the most pressing public health challenges nowadays. Aminoglycosides are extensively used in the clinic, due to their effectiveness against both Gram-positive and Gram-negative species. However, emerging resistance toward aminoglycosides increasingly jeopardizes the efficacy of this class of antibiotics and demands for additional therapeutic modalities^1^. Given the essential role of aminoglycosides in treating infections caused by pathogens listed as critical and high priority by the World Health Organization (WHO)^2^, strategies to safeguard the clinical efficacy of aminoglycosides are urgently needed to address the growing threat of multidrug-resistant (MDR) bacteria. Resistance to aminoglycosides can occur through multiple mechanisms, among which deactivation by bacterial enzymes is one of the most prominent^3^. Aminoglycoside-modifying enzymes encompass three main classes: i) acetyltransferases (AACs), ii) nucleotidyltransferases (ANTs), and iii) phosphotransferases (APHs). APHs inactivate aminoglycosides by catalyzing the transfer of a phosphoryl group from a phosphoryl donor, ATP or GTP, to a hydroxyl group present on aminoglycosides^4,5^. Targeting APHs holds potential as a target to tackle resistance since (i) APHs are often implicated in MDR phenotypes, especially in high-risk pathogens; (ii) APH phosphorylation often impacts a broad range of aminoglycosides, therefore APHs are considered clinically more relevant^6^. In light of these considerations, developing small molecule inhibitors of APHs could pave the way to counteracting aminoglycoside resistance^7^.

In the past, significant efforts have been dedicated to discovering compounds targeting APHs. Key strategies have included repurposing existing eukaryotic protein kinase inhibitors, targeting novel allosteric pockets, and designing novel antibiotics or de novo APH inhibitors.

The structural similarity between APHs and eukaryotic protein kinases (ePKs) has inspired the repurposing of ePK inhibitors to address APH-mediated resistance. Shakya et al. characterized the inhibition of different APHs by diverse protein kinase inhibitors (PKI), revealing differences in binding modes compared to ePKs and identifying compounds that restore aminoglycoside efficacy against resistant bacteria^8^. Several natural products show activity against APHs. Flavonoids like quercetin, a broad-spectrum kinase inhibitor, were found to bind to several APH isoforms^7^.

In a study by Daigle et al., the interactions of kinase inhibitors from three distinct structural classes—indole carbazoles, flavonoids, and isoquinoline sulfonamides—were evaluated against several APH enzymes. Notably, members of the isoquinoline sulfonamide class demonstrated significant inhibitory activity against APH(3’)-IIIa and the bifunctional AAC(6’)-APH(2’) enzyme, both found in *S. aureus* and *Enterococci*. However, despite their in vitro potency, these compounds failed to restore aminoglycoside efficacy in bacterial culture assays. Nevertheless, the findings provided an important proof-of-concept for targeting APHs with kinase-directed scaffolds^9^.

In a separate investigation, the casein kinase 1 inhibitor CKI-7 exhibited an inhibition constant (Kᵢ) of approximately 65 µM against APH(3’)-IIIa^10^, further supporting the viability of this strategy.

Building upon this concept, subsequent studies explored additional ePK inhibitors with distinct scaffolds and mechanisms of kinase selectivity. For instance, the anthrapyrazolone SP600125, a canonical JNK inhibitor, was found to inhibit APH(3′)-Ia from *E. coli* in vitro and exhibited modest synergy when combined with kanamycin. However, this effect was insufficient to fully restore antibacterial activity. Still, its performance as a chemical probe reinforced the druggability of the APH ATP-binding pocket and its potential as a target for rational inhibitor design.

Extending this rationale further, the pyrazolopyrimidine (PP) inhibitors 1-NA-PP1 and 1-NM-PP1, originally developed as selective kinase inhibitors for prostate cancer research and found to be inactive against wild-type ePKs due to steric clashes with a “gatekeeper” residue in the ATP-binding site^11^, were shown to bind APH(3’)-Ia (K_i_ = 21.5 µM and K_i_ = 34.4 µM, respectively). When tested in a hyperpermeable *E. coli* Δ*tolC* Δ*bamB* strain expressing *aphA1*, both inhibitors were able to almost completely restore kanamycin effectiveness. However, they failed to resensitize wild-type *E. coli* strains expressing *aphA1*, likely due to reduced permeability and/or active efflux^12^.

Exploiting distinct allosteric sites often offers a way to bypass cross-reactivity between bacterial phosphotransferases and mammalian kinases, which commonly share conserved ATP-binding domains.

The docking study performed by Leban et al. identified compound NL8 as an ATP-competitive APH(3’)-IIIa inhibitor, and NL6, which showed activity against both APH(3’))-IIIa from *S. aureus* and *Enterococci* and APH(2”)-IVa from *E. casseliflavus*. NL6, identified as a non-competitive APH inhibitor was further optimized, unfortunately resulting into analogs which displayed lower efficiency^13^.

In a recent study by Kaplan et al., a combination of an in silico screening and molecular dynamics simulations led to the discovery of compound EK3, currently the most potent allosteric inhibitor of APH(2”)-IVa identified to date. Although EK3 stood out as a promising allosteric inhibitor, the authors emphasize that further structure-based optimization is required to improve its in vitro potency^8^.

Alternative strategies to counteract enzyme-mediated antibiotic resistance have focused on developing next-generation antibiotics, such as plazomicin^14^, whose structure has been chemically modified to specifically evade inactivation by some AMEs^15,16^. Despite its significant antibacterial potential against MDR *Enterobacteriaceae*, plazomicin faces several limitations that restrict its clinical use. These include susceptibility to certain AMEs, including APH(2”)-IVa^16^, and challenges in achieving sustainable market access due to financial and commercial hurdles^17^. Another example is represented by propylamycin and analogs thereof, developed with the aim of reducing ototoxicity and overcoming inactivation by APH(3’) enzymes^18^.

Further alternative approaches have utilized a combinatorial library of designed ankyrin repeat (AR) proteins as allosteric inhibitors of APH(3’)-IIIa, representing promising starting points for rational drug design^19,20^. Although progress has been made, their clinical application remains distant and requires further studies.

The de novo design of APH inhibitors is still in its early stages, with structure-based design and virtual screening emerging as promising but still underexplored strategies. For example, a library of 1,3-diamines was rationally designed to mimic the 2-deoxystreptamine ring of the aminoglycoside pharmacophore without the sugar backbone, showing competitive inhibition of key AMEs such as ANT(2”) and APH(3’)^21^.

More recently, a virtual screening performed by Parulekar et al. identified ZINC71575479 as a competitive inhibitor of APH(5) that could completely inhibit APH(5) from the multidrug-resistant organism *Bacillus subtilis* subsp. *subtilis* strain RK. In silico toxicity studies showed that ZINC71575479 has an acceptable toxicity profile, indicating its potential as a lead compound for developing inhibitors targeting APH enzymes^22^.

**Fig. 1:**
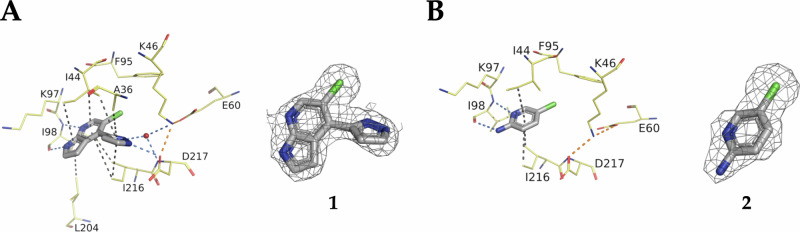
Crystal structures of APH(2”)-IVa in complex with the two identified hit scaffolds, **1** and **2**. Crystal structures of APH(2”)-IVa in complex with **1** (**A**, PDB 9QOD) and **2** (**B**, PDB 9QNQ) and their corresponding omit maps contoured at a sigma level of ± 1. Inhibitors are represented in grey sticks, water molecule as red sphere and residues involved in interactions are shown as yellow lines. Interactions are shown as dashed lines: Van der Waals interactions in grey, hydrogen bonds in blue and ionic bonds in orange.

In the accompanying paper^23^, we report a fragment-based screening in which we identified the 7-azaindole **1** and the pyridine-2-amines like **2** as ATP-competitive inhibitors of APH(2”)-IVa, APH(3’)-IIa from *K. pneumoniae* and APH(3’)-IIb from *P. aeruginosa* (Fig. 1). Here, we present a structure-activity relationship (SAR) study to explore the scope of these scaffolds as APH inhibitors and as potential anti-resistance agents.

## Results

Based on the cocrystal structure of **1** with APH(2”)-IVa (Fig. 1A), we initially focused on the functionalization of position 3’ of the pyrazole ring of **1**, by assessing the tolerability toward various substitutions. The addition of a residue at this position would extend the molecule toward the catalytic triads consisting of Lys46, Glu60 and Asp217, which play a role in the coordination of the phosphates of the endogenous substrates ATP or GDP^24^. Therefore, engaging into new interactions could improve binding affinity of the resulting APH inhibitors.

**Fig. 2:**
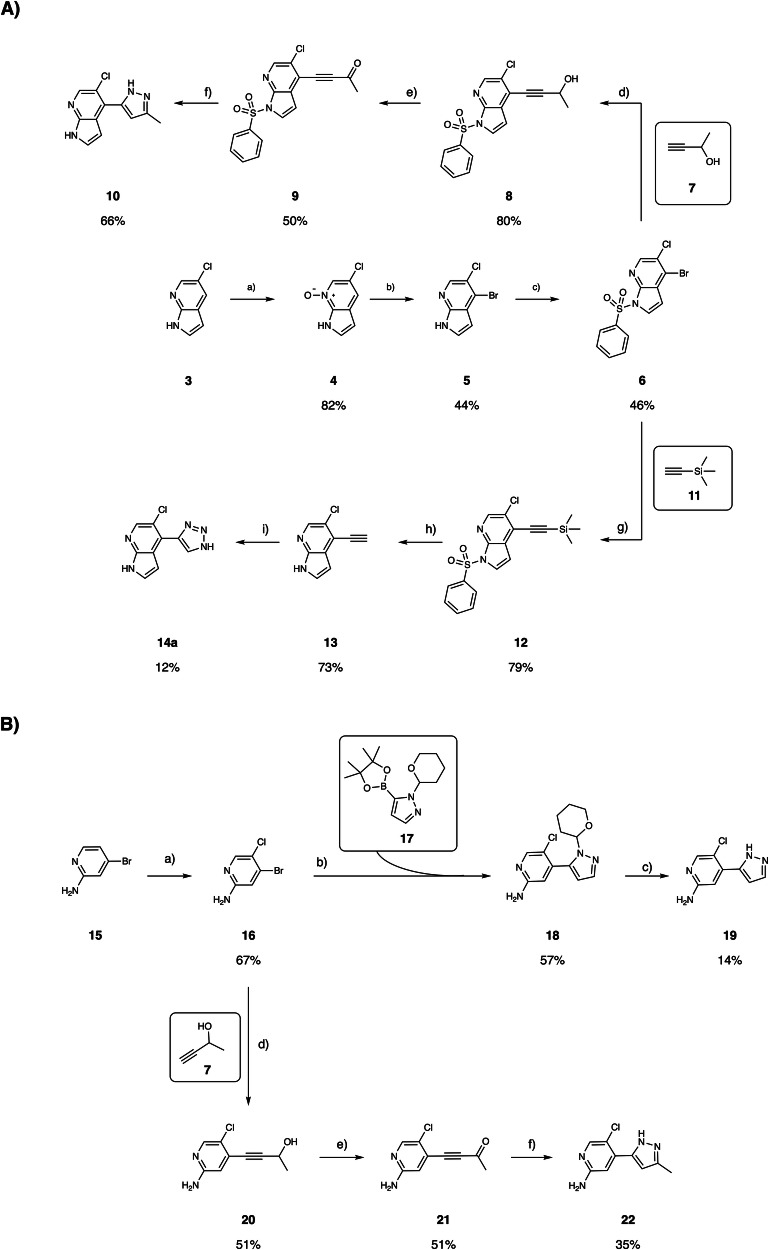
Synthesis of analogs of **1** and **2**. **A** Synthesis of 7-azaindole derivatives **10** and **14a**:**a**) a) *m*-CPBA, DME:*n*-Hexane (2:1, v/v), rt; b) tetramethylammonium bromide, methanesulfonic anhydride, DMF, -10 °C to rt; **c**) c) benzenesulfonyl chloride; NaH, DMF, 0 °C to rt; d) Pd(dppf)Cl_2_, TMEDA/DMF (1:1) 90 °C; **e**) DMP, DCM, 0 °C to rt; f) NH_2_NH_2_ • H_2_O, H_2_SO_4_, NaOH, EtOH, reflux to rt; **g**) Pd(PPh_3_)_4_, TMEDA/DMF (1:1) 80 °C; h) K_2_CO_3_, MeOH, rt; i) NaN_3_, sodium ascorbate, CuSO_4_(H_2_O)_5_, H_2_O/*t*-BuOH (1:1), rt. **B** Synthesis of the pyridine-2-amine derivatives **19** and **22**. **a**) NCS, DMF, -20 °C to rt; b) Pd(dppf)Cl_2_, XPhos, K_2_CO_3_, dioxane/H_2_O (6:1), 80 °C, 4 h; c) 3 M HCl, MeOH; d) CuI, PPh_3_, Pd(OAc)_2_, ACN/Et_3_N, 85 °C, 20 h; e) Jones reagent, acetone, 0 °C to rt; f) NH_2_NH_2_•H_2_O, EtOH.

To investigate the tolerability toward substitution in position 3’ of the pyrazole ring, we first synthesized the methyl-substituted analog **10**. Toward this, the commercially available **3** was treated with m-CPBA to provide N-oxide **4**, which was in turn regioselectively brominated to yield **5**. Treatment of the latter with benzenesulfonyl chloride gave **6** which was further subjected to a Sonogashira reaction with but-3-yn-2-ol **7** to give intermediate **8** in 80% yield. Subsequent oxidation with Dess-Martin Periodinane (DMP) successfully converted **8** into the ynone **9**, obtained in 50% yield. Finally, treatment with hydrazine and H_2_SO_4_ gave the desired pyrazole-containing analogue **10** in 61% yield (Fig. 2A). We also pursued the triazole derivative **14a**, as the additional nitrogen atom aligned with the desired exit vector in the 3’-position. Toward this end, **6** was subjected to a Sonogashira reaction with trimethylsilylacetylene **11** to provide **12** in 79% yield. Subsequently, treatment with K_2_CO_3_ successfully removed both the silyl and the phenylsulfonyl groups yielding **13** in 73% yield. Finally, Cu-catalyzed reaction of alkyne **13** with NaN_3_ provided the desired triazole **14a** (Fig. 2A).

In parallel, we tested the versatility of the pyridine-2-amine scaffold (Fig. 2B) by synthesizing **19** and **22** as direct analogs of **1**, **10**, and **2**. Toward this, the commercially available 4-bromopyridin-2-amine **15** was treated with N-chlorosuccinimide (NCS) and the desired regioisomer **16** was isolated in 31% yield (Fig. 2B). Suzuki coupling between **16** and the commercially available 1-(tetrahydro-2H-pyran-2-yl)-5-(4,4,5,5-tetramethyl-1,3,2-dioxaborolan-2-yl)-1H-pyrazole **17** gave **18** in moderate yield (57%). Finally, treatment with 3 M HCl successfully cleaved the THP protecting group and afforded the desired aminopyridine derivative **19** in 67% yield. **22** was synthesized following the same pathway used for **10** (Fig. 2B).

The effects of **10, 14a, 19** and **22** on the thermostability and activity of APH(2”)-IVa were measured by thermal shift assay and using the Pyruvate Kinase-Lactate Dehydrogenase-coupled assay (Table 1). Overall, these orthogonal assays correlated, provided a reliable assessment of the inhibitory activity of the compounds, and confirmed the ATP-competitive mode of inhibition.

**Table 1:** Biochemical characterization of compounds **10**, **14a**, **19** and **22** and comparison with **1**

Entry	Structure	ΔTm (°C) at 500 µM	Inhibition (%) at 10 µM	K_i_ (nM)	X-ray structure resolution (Å)
**1**		3.23	90%	388 ± 36	2.29
**2**		1.25	0.5%	~500,000	2.38
**10**		2.27	89%	360 ± 15	ND
**14a**		1.31	83%	1,300 ± 55	2.30
**19**		0.39	26	20,000 ± 2,100	2.44
**22**		0.51	40%	9,950 ± 420	ND

ND: not determined.

**10** showed an inhibition constant comparable to that of **1** (K_i_ = 388 nM, Table 1), while the inhibition efficacy of the unsubstituted triazole analogue **14a** slightly dropped. The enzyme inhibition assay revealed a 52- and 25- fold decrease in binding affinity for pyridine-2-amine analogs **19** and **22** when compared to **1**. As a result, we discontinued further exploration of the pyridine-2-amine scaffold (Table 1 and Supplementary Fig. 6).

**Fig. 3:**
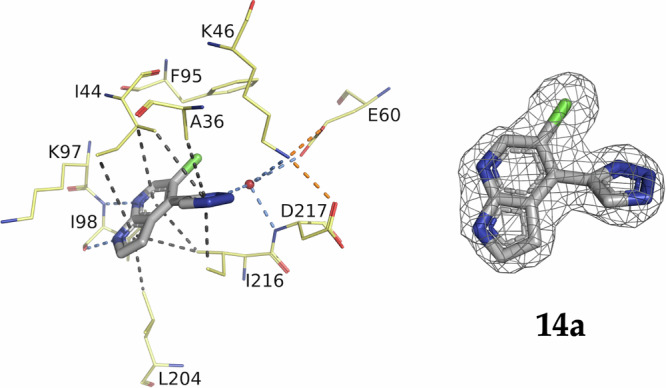
Crystal structure of APH(2”)-IVa in complex with 14a. Crystal structure of APH(2”)-IVa in complex with **14a** (PDB 9QOT) and its corresponding omit map contoured at a sigma level of ± 1. Inhibitor is represented in grey sticks, residues involved in interactions are shown as yellow lines and water molecule is represented as red sphere. Interactions are shown as dashed lines: van der Waals interactions in grey, hydrogen bonds in blue and ionic bonds in orange.

**Fig. 4:**
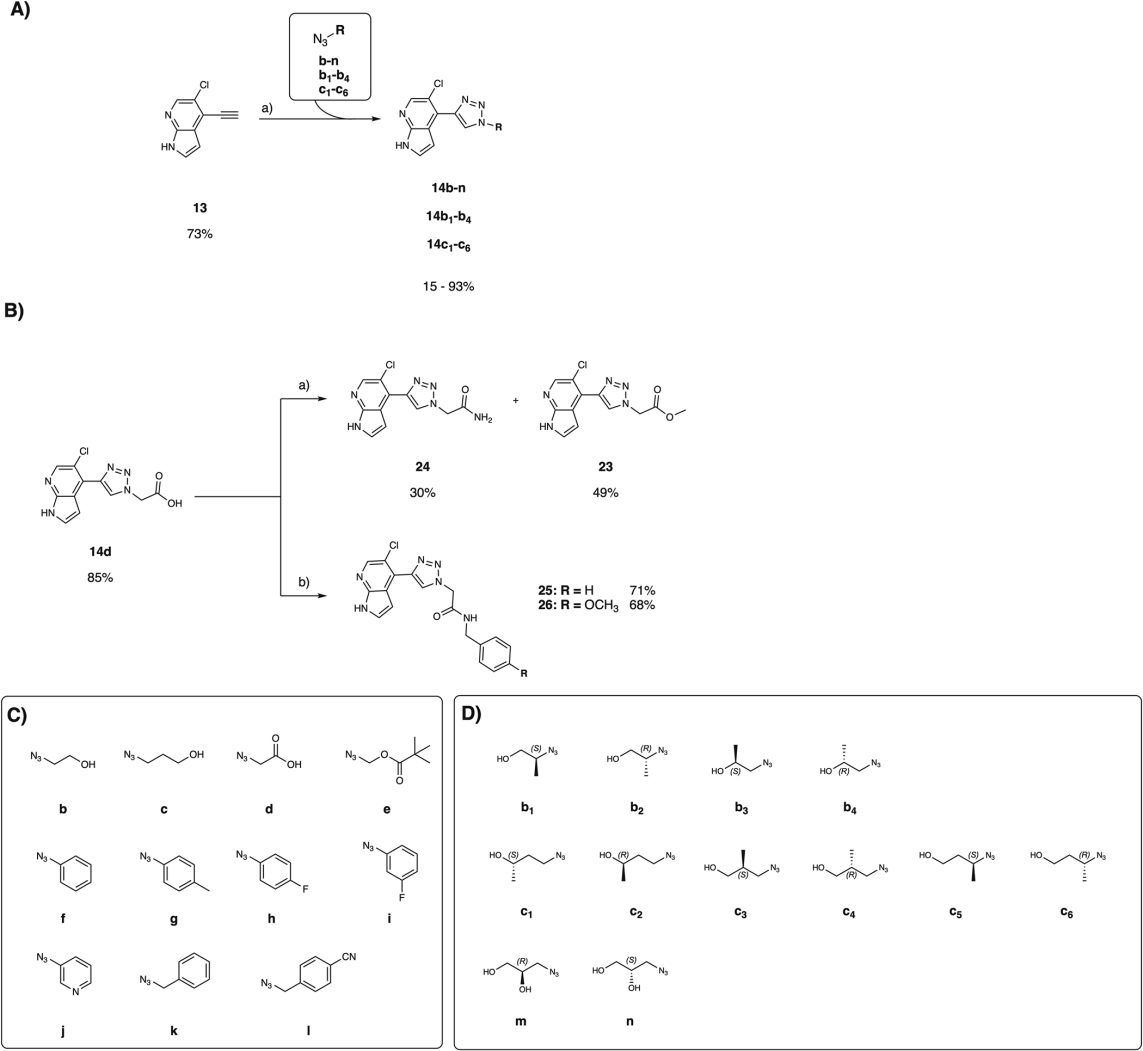
Synthesis of the triazole analogs **14b-n**, **14b1**-**14b4**, **14c1**-**14c6**. **A** a) Azides, sodium ascorbate, CuSO_4_(H_2_O)_5_, H_2_O/t-BuOH (1:1), rt. **B** a) di(1H-imidazol-1-yl)methanone, NH_3_, DMF, rt; b) Benzylamine or p-methoxybenzylamine, DIPEA, HATU, DMF, 25 °C. **C** Commercially available azides used for derivatization. **D** Custom-made azides used for derivatization.

Intrigued by the performance of **14a**, we solved the co-crystal structure in complex with APH(2”)-IVa (Fig. 3). This confirmed the desired binding mode and revealed the nitrogen in position 3’ as a promising exit vector to engage residues such as Asp217, Glu60 and Lys46. Therefore, a first series of triazole analogs (**14b-l**) was synthesized (Fig. 4A), following the same route used for **14a** and using the commercially available azides (Fig. 4C) The acid derivative **14 d** was further amidated or esterified to provide **23**, **24**, **25** and **26** (Fig. 4).

The effects of triazole analogs were measured as before by thermal shift assay, and in the enzymatic-coupled assay (Table 2). The latter revealed that introducing the charged carboxylic acid (**14 d**, K_i_ = 14,500 nM) compromised binding, whereas unsubstituted amide derivative **24** (K_i_ = 799 nM) and the ester **23** (K_i_ = 425 nM) improved binding compared to **14a**. The presence of the hydroxyl group in compound **14 d** probably works against this stacking. In general, introducing aromatic residues was not tolerated, with the notable exception of **14j** (K_i_ = 376 nM). We were pleased to observe that the alcohol **14c** (K_i_ = 458 nM) had improved affinity compared to **14a** (Table 2).

**Table 2:** Biological data of the first series of triazole analogs 14a-l and 23-26

**Entry**	**Structure**	**R**	Δ**Tm (°C) at 500 µM**	**Inhibition (%) at 10 µM**	**K**_**i**_ **(nM)**	**X-ray structure resolution (Å)**
**14a**		-H	1.31	83%	1,300 ± 55	2.30
**14b**			1.12	77%	2,400 ± 320	2.06
**14c**			1.20	88%	458 ± 37	ND
**14 d**			1.36	24%	14,500 ± 1,400	ND
**23**			1.48	91%	425 ± 32	2.30
**14e**			2.04	82%	2,400 ± 160	ND
**24**			0.45	82%	799 ± 81	2.45
**25**			-0.20	62%	9,900 ± 600	ND
**26**			-0.33	25%	>10,000	ND
**14 f**			0.49	87%	916 ± 78	2.50
**14 g**			-0.20	40%	>10,000	ND
**14 h**			0.23	73%	6,300 ± 1,000	ND
**14i**			0.16	72%	2,700 ± 450	ND
**14j**			1.03	84%	376 ± 21	ND
**14k**			0.37	63%	6,500 ± 400	2.55
**14 l**			0.26	44%	16,200 ± 3,700	ND

ND: not determined.

In light of the promising biochemical activities of the 7-azaindole triazoles, we determined their first-line ADME properties to assess their principal suitability as potential drugs (Table 3). With the exception of **14e**, **14 f**, **14 g**, **14i**, all tested 7-azaindole triazoles were highly stable in murine and human microsomes. Most compounds were stable in human plasma, but several of them were only partially stable in mouse plasma, which could be attributed to slightly different enzymes available, highlighting the importance of the multiparameter optimization. Several compounds were highly bound to human plasma proteins, which was slightly less pronounced for mouse plasma proteins. A certain extent of plasma protein binding is highly accepted, as it is thought to lower renal clearance, because only the free fraction will be excreted. By contrast, high plasma protein binding (>95-99%) is associated with the concern of low efficacy as a result of only a small free fraction. However, also drugs on the market, like dalbavancin, harbor high plasma protein binding (>99%) and are still effective^25^. An even higher plasma protein binding capacity has been observed for a drug class in preclinical development, cystobactamids, harboring a plasma protein binding of 100% in in vitro assays. Despite that, efficacy was observed leading to the hypothesis that plasma protein binding capacities might be altered during infection^26^. Although plasma protein binding was high here, overall, the inhibitors had favorable ADME properties, with good metabolic and plasma stability and an acceptable plasma protein binding in light of the indication for treatment of bacterial infections.

**Table 3:** ADME properties of compounds **1**, **10**, **14a-14k**, **24-26** and **14c2-c4**

Entry	t_1/2_ mouse plasma (min)	t_1/2_ human plasma (min)	PPB mouse (%)	PPB human (%)	t_1/2_ mouse microsomes (min)	Cl mouse (μL∙min^-1^ ∙ g^-1^ protein)	t_1/2_ human microsomes (min)	Cl human (μL∙min^-1^ ∙ g^-1^ protein)
**1**	> 240	> 240	71.5 ± 19.3	74.9 ± 14.2	>60	<23	>60	<23
**10**	180.1	250.8	84.2 ± 9.6	94.8 + 3.4	>60	<23	>60	<23
**14a**	175.8	>240	77.9 ± 13.6	98.9 ± 0.5	75.26	18.4	>60	<23
**14b**	165.5	>240	72.0 ± 18.0	88.7 ± 3.7	>60	<23	>60	<23
**14c**	>240	>240	62.0 ± 6.2	61.0 ± 10.8	>60	<23	>60	<23
**14e**	<5	79.6	n.d.	n.d.	40.44	34.22	2.92	473.2
**14 f**	127.3	>240	98.0 ± 0.7	98.8 ± 0.3	25.7	53.9	19.3	72.0
**14 g**	114.6	241.0	99.5 ± 0.1	99.4 ± 0.5	19.3	72.0	19.3	72.0
**14 h**	123.5	<5	97.3 ± 0.8	97.2 ± 1.8	>60	<23	>60	<23
**14i**	115.8	>240	98.7 ± 0.6	99.2 ± 0.4	2.63	526.7	1.76	784.5
**14j**	133.5	>240	81.6 ± 8.2	89.9 ± 8.0	>60	<23	>60	<23
**14k**	134.3	>240	99.6 ± 0.6	98.3 ± 1.7	>60	<23	>60	<23
**24**	116.2	201.4	19.3 ± 10.7	55.3 ± 9.9	>60	<23	>60	<23
**25**	146.4	>240	82.0 ± 17.7	95.2 ± 4.5	>60	<23	>60	<23
**26**	127.7	>240	81.1 ± 10.4	95.4 ± 0.9	>60	<23	>60	<23
**14c2**	> 240	> 240	10.1 ± 2.2	24.6 ± 6.8	>60	<23	>60	<23
**14c3**	> 240	> 240	11.2 ± 8.6	26.4 ± 11.1	>60	<23	>60	<23
**14c4**	> 240	> 240	11.5 ± 10.6	22.0 ± 14.6	>60	<23	>60	<23

*PPB* plasma protein binding, *Cl* clearance.

**Fig. 5:**
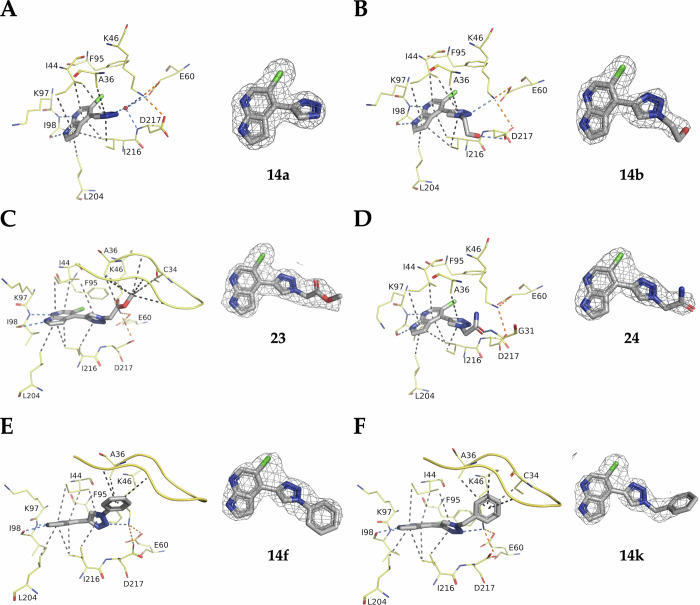
Crystal structures of APH(2”)-IVa in complex with the triazole analogs **14a**, **14b**, **23**, **24**, **14f** and **14k.** Crystal structures of APH(2”)-IVa in complex with **14a** (**A**, PDB 9QOT),**14b** (**B**, PDB 9QOX), **23** (**C**, PBD 9RL1), **24** (**D**, PBD 9QOW), **14 f** (**E**, PDB 9QOZ) and **14k** (**F**, PDB 9QOU) and their corresponding omit maps contoured at a sigma level of ± 1. Inhibitors are represented in grey sticks, residues involved in interactions are shown as yellow lines. Interactions are shown as dashed lines: van der Waals interactions in grey, hydrogen bonds in blue and ionic bonds in orange.

The crystal structures of APH(2”)-IVa in complex with compounds **14b**, **23**, **24**, **14 f**, and **14k** confirmed a binding mode similar to **14a**. These structures helped us explore structurally the binding pocket (Fig. 5). Indeed, even if these compounds were not better at inhibiting the enzyme, they showed interactions with the loop of the β-sheet located just above the ATP binding site. For example, the presence of the methoxy group in compound **23** favors stacking on the strands 1 and 2 of the neighbouring beta sheet. The presence of the hydroxyl group in compound **14d** probably works against this stacking. Moreover, the short and/or rigid extensions do not allow flexibility to interact with the loop that is highly flexible itself. This suggests that by adding a longer extension to these compounds, we could potentially stabilize them with this flexible loop and make new interactions with the protein that may increase their inhibitory effect.

To further improve affinity, we systematically introduced methyl groups at the hydroxy ethylene substituent of **14b** by employing a set of substituted azidoethanol building blocks (Fig. 4D), yielding **14b1-b4** (Fig. 4A)^27^. This indeed improved affinity in all four cases when compared to **14b** (Table 4).

**Table 4:** Biochemical characterization of ethanol- or propanol-containing triazole analogs

Entry	Structure	R	ΔTm (°C) at 500 µM	Inhibition (%) at 10 µM	K_i_ (nM)	X-ray structure resolution (Å)
**14b**			1.12	77%	2,400 ± 320	2.06
**14b1**			1.94	88%	613 ± 76	2.35
**14b2**			1.09	74%	1,004 ± 92	ND
**14b3**			1.60	86%	534 ± 51	ND
**14b4**			1.27	87%	443 ± 25	ND
**14c**			1.20	88%	458 ± 37	ND
**14c1**			1.31	90%	325 ± 25	2.68
**14c2**			1.33	91%	444 ± 26	ND
**14c3**			1.51	91%	375 ± 25	2.65
**14c4**			1.47	91%	367 ± 21	2.05
**14c5**			1.58	86%	482 ± 36	2.00
**14c6**			1.43	90%	348 ± 27	2.35
**14 m**			1.56	85%	479 ± 45	2.50
**14n**			1.21	90%	404 ± 35	2.50

The promising affinity and excellent ADME profile of **14c** motivated us to further synthesize analogs **14c1-c6**, containing substituted azidopropanol units including the diols **14 m** and **14n**. Gratifyingly, all analogs retained high binding to APH(2”)-IVa ( < 500 nM, Table 4). Slight improvements over **14c** were observed for several analogs such as **14c1** (K_i_ = 325 nM), **14c3** (K_i_ = 375 nM), **14c4** (K_i_ = 367 nM), and **14c6** (K_i_ = 348 nM).

**Fig. 6:**
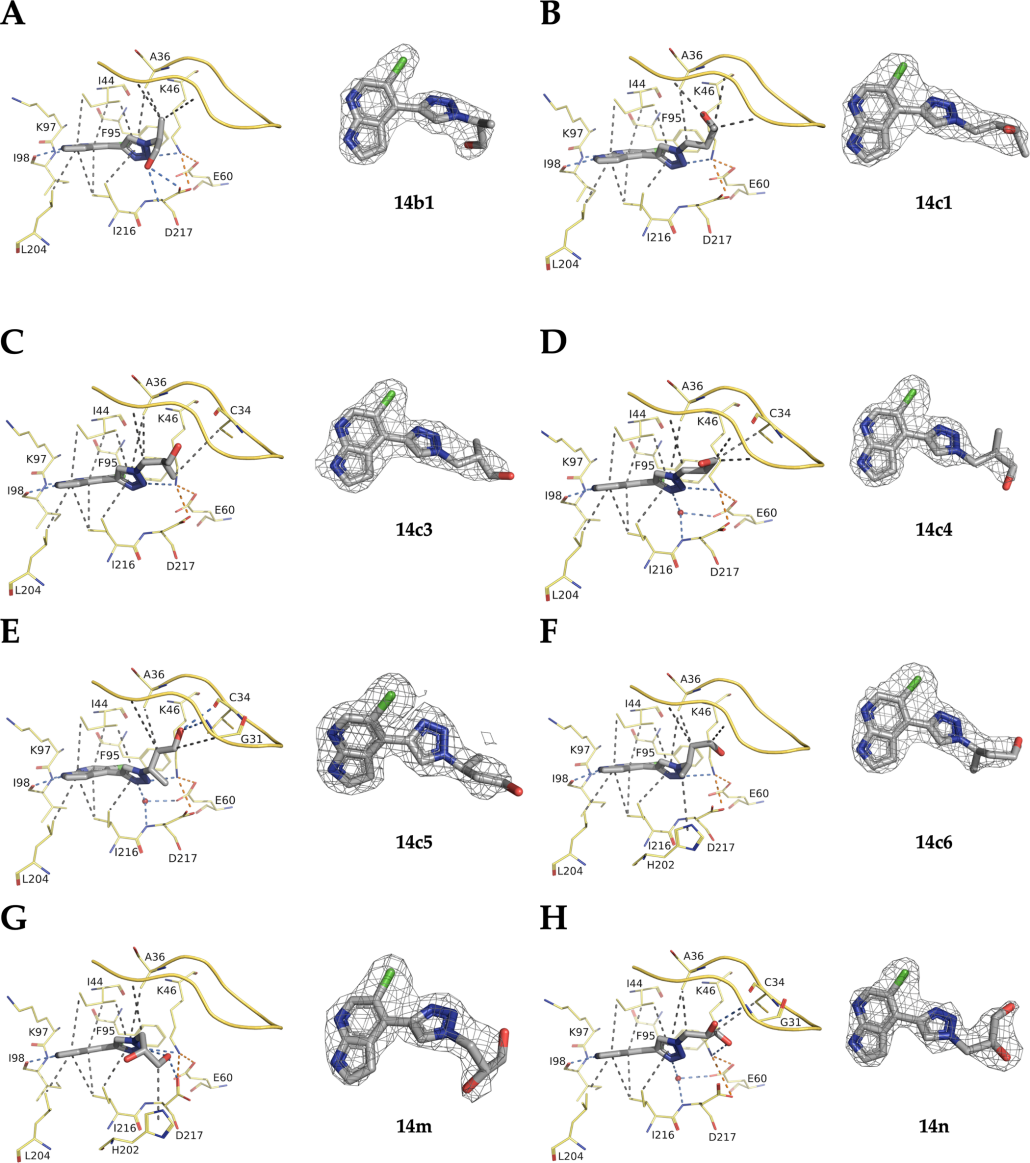
Crystal structures of APH(2”)-IVa in complex with the triazole analogs **14b1**, **14c1**, **14c3**, **14c4**, **14c5**, **14c6**, **14m** and **14n**. Crystal structures of APH(2”)-IVa in complex with **14b1** (**A**, PDB 9QP6), **14c1** (**B**, PDB 9QP0), **14c3** (**C**, PDB 9QP1), **14c4** (**D**, PDB 9QP2), **14c5** (**E**, PDB 9QP3), **14c6** (**F**, PDB 9QP5) **14m** (**G**, PDB 9QP7), **14n** (**H**, PDB 9QPA) and their corresponding omit maps contoured at a sigma level of ± 1. Inhibitors are represented in grey sticks, residues involved in interactions are shown as yellow lines and water molecules are represented as red spheres. Interactions are shown as dashed lines: van der Waals interactions in grey, hydrogen bonds in blue and ionic bonds in orange.

Inspired by the results, we solved the crystal structures of APH(2”)-IVa in complex with some of the synthesised analog compounds (**14b1**, **14c1**, **14c3-c6**, **14 m** and **14n**), showing interactions with the loop of the β-sheet located just above the binding pocket (Fig. 6). The extension arm of these compounds is stabilised via Van der Waals interactions with either the backbone or the side chains of the residues forming this loop. The fact that the efficacy of most of these compounds is not diminished compared to **14c** is promising as this region of the protein can be highly flexible. Moreover, considering that we can stabilise this loop without losing effect, we can now focus on targeting specifically the residues located at the junction of the ATP and the aminoglycoside binding sites to increase selectivity.

Exemplarily, compounds **14c2, 14c3 and 14c4** were characterized for their ADME properties (Table 3). This confirmed that the modifications in the triazole substituent part retained the excellent drug-like profile of **14c**.

Additionally, the most advanced APH inhibitors were tested in a broad kinase screening to assess the selectivity versus human kinases.^28,29^ Toward this goal, a panel of 101 purified human kinases and common kinase off-targets were tested in a thermal shift assay. The major human off-targets identified were BIKE, AAK1, AurA, MSK1_b, MAP3K5, DRAK1 and AURKB (Table 5 and Supplementary Table 2). We assume that the affinity for the off-targets lies in the high micromolar to millimolar range. As an example, a waterfall plot illustrating the activity of **1** is provided in the Supplementary Fig. 2. Notably, Erk-2 was only minimally inhibited by **1** (Supplementary Fig. 3), consistent with the cocrystal structure of **1** with Erk-2 (PDB 9QQJ, Supplementary Fig. 5), which revealed an opposite/reversed binding mode to that observed when **1** interacts with APHs. Inhibition of these human targets has to be considered in assays with human or mammalian cells. Further development of these APH inhibitors should aim at improving the selectivity ratio vs these human kinases.

**Table 5:**
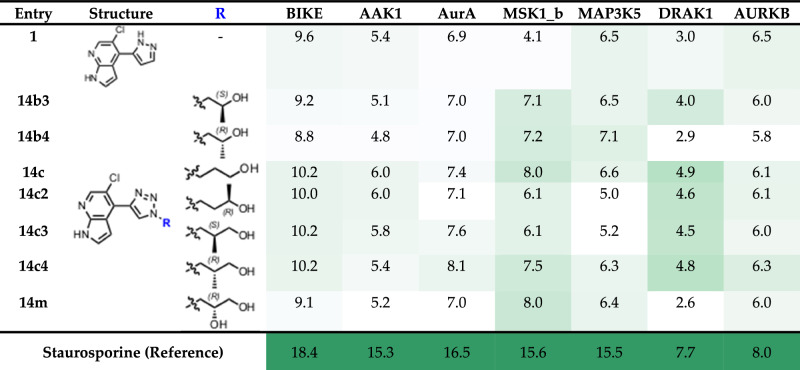
Kinase panel screening of compounds 14b3-b4, 14c, 14c2-4 and 14 m

ΔTm data (°C) were collected at 10 μM inhibitor concentration by differential scanning fluorimetry (DSF).

**Fig. 7:**
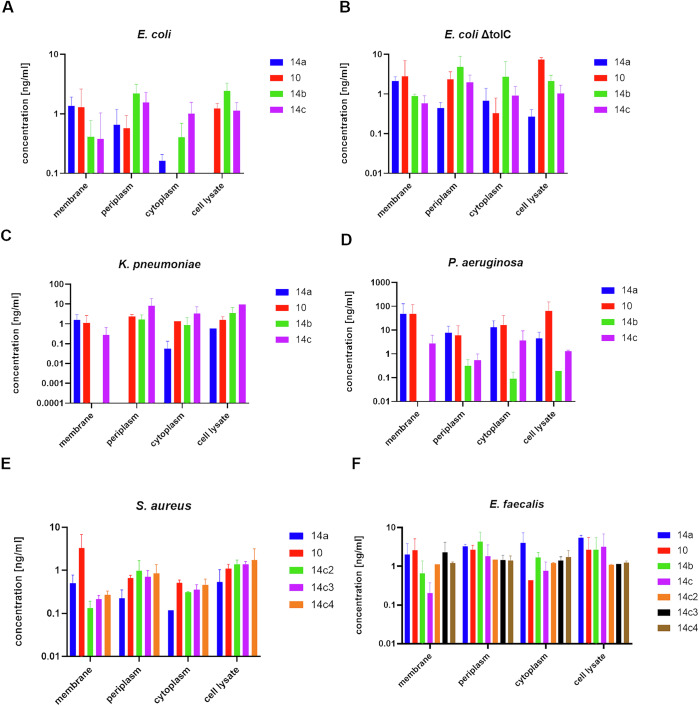
Uptake studies for **14a**, **10**, **14b**, **14c**, **14c2**, **14c3** and **14c4** in Gram-negative and Gram-positive bacterial pathogens. **A** Uptake assay for **14a**, **10**, **14b** and **14c** in *E. coli*. **B** Uptake assay for **14a**, **10**, **14b** and **14c** in *E. coli* Δ*tolC* strain. **C**) Uptake assay for **14a**, **10**, **14b** and **14c** in *K. pneumoniae*. **D**) Uptake assay for **14a**, **10**, **14b** and **14c** in *P. aeruginosa*. **E)** Uptake assay for **14a**, **10**, **14b**, **14c, 14c2, 14c3** and **14c4** in *E. faecalis*. **F)** Uptake assay for **14a**, **10**, **14b**, **14c, 14c2, 14c3** and **14c4** in methicillin-resistant *S. aureus*. Assays were conducted in biological triplicate. The results are displayed as mean and standard deviation. Concentrations were assessed in membrane, periplasm, cytoplasm and cell lysate (from left to right). Blue bars **14a**, red bars **10**, green bars **14b**, purple bars **14c**, orange bars **14c2**, black bars **14c3**, brown bars **14c4**.

Based on the promising biochemical activity and the positive ADME assessment, diazole **10** and the triazoles **14a**, **14b** and **14c** were further characterized in several bacterial uptake/permeability assays. First, we validated our uptake assay using ciprofloxacin as an established positive control^30^ with the wild-type *E. coli* and the Δ*tolC* strain, deficient of an active efflux pump. As previously described, higher concentrations of ciprofloxacin were observed in the cytoplasm when the Δ*tolC* strain was used (Supplementary Fig. 1). Gratifyingly, the alcohols **14b** and **14c**, but not **10** and only partially **14a** showed cellular uptake for wild-type *E. coli* (Fig. 7A). Primarily, compounds were detected in the periplasm, but only in very low concentration in the cytoplasm. When using the *E. coli* Δ*tolC* strain, higher concentration of all four compounds were found in the cyto- and periplasm (Fig. 7B). Next, we investigated the cellular uptake for other Gram-negative bacteria, such as *K. pneumoniae* (wild type) and *P. aeruginosa* (wild type). It was observed that slightly higher concentrations compared to *E. coli* were detected in the cytoplasm, but also in the periplasm. Overall, compound levels remained low (Fig. 7C, D). Whereas the alcohols **14b** and **14c** had slightly higher cellular uptake in *K. pneumoniae*, **10** and **14a** had a more favorable uptake in *P. aeruginosa* (Fig. 7D). We hypothesize that this might be attributed to slight differences in transporters and the cell membrane in these different bacterial pathogens. Moreover, ten-fold higher concentrations in cytoplasm, membrane and periplasm were observed for all compounds tested with *P. aeruginosa*. We can only hypothesize that this might be attributed to a better attachment to the membrane resulting in subsequent higher concentrations being taken. As Gram-positive strains typically harbor structurally different efflux pumps, i.e. RND family efflux pumps, such as AcrAB / *tolC*, which are only present in Gram-negative bacteria and mainly responsible for efflux of aminoglycosides, as well as a different membrane structure^31–33^ we investigated cellular uptake in *E. faecalis* and methicillin-resistant *S. aureus* (MRSA). Moreover, we tested three additional compounds, i.e. **14c2**, **14c3** and **14c4**. As expected, high levels in periplasm and cytoplasm were observed for all tested compounds. Compounds **14c2**, **14c3** and **14c4** had higher levels in cytoplasm compared to periplasm, for both *E. faecalis* and MRSA (Fig. 7E, F). Thus, it can be concluded that the structural variations introduced in **14c2**, **14c3** and **14c4** led to an increased uptake into cytoplasm in the Gram-positive strains tested herein compared to the first-generation derivatives **10**, **14a**, **14b** and **14c**.

**Fig. 8:**
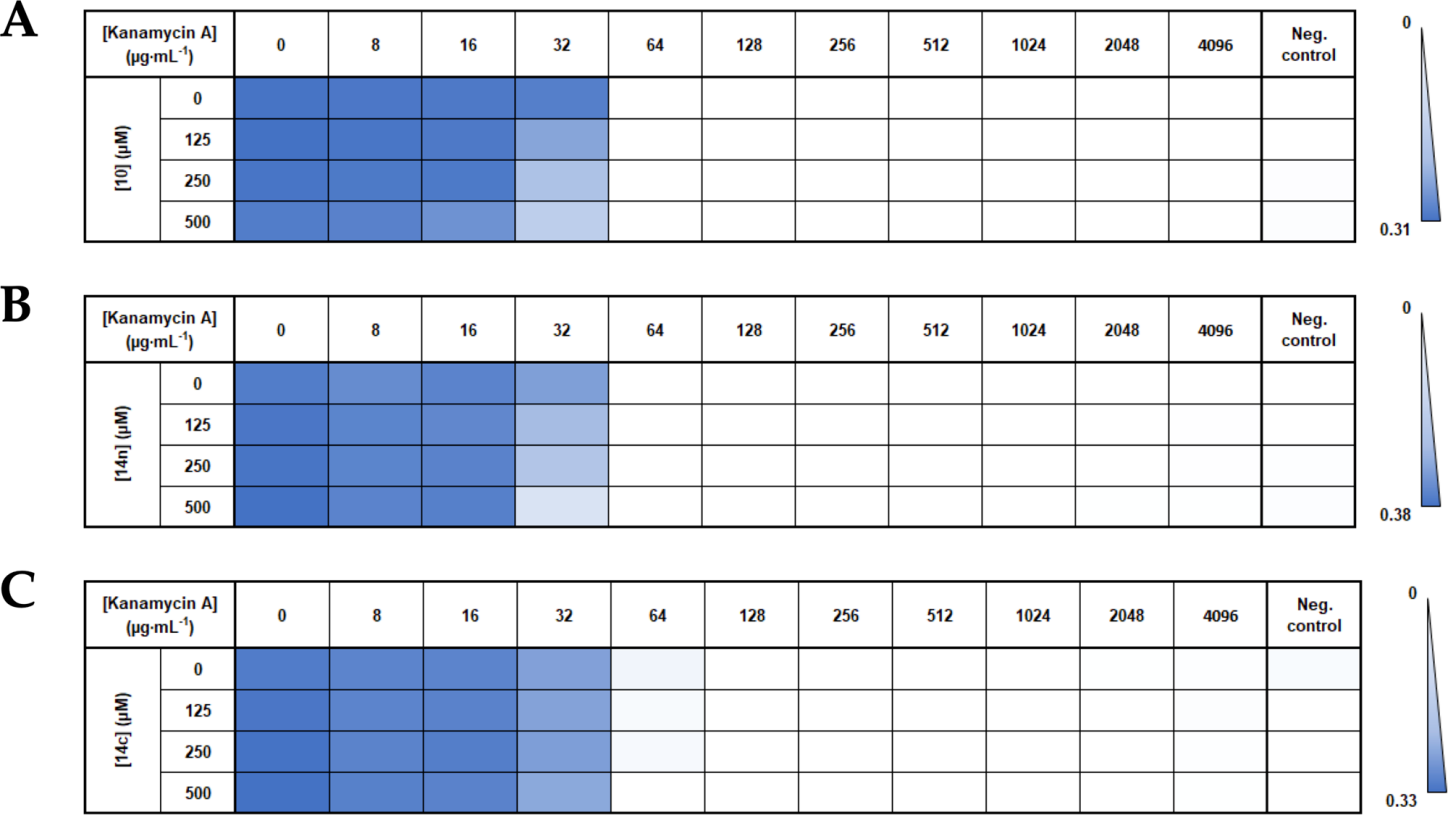
Checkerboard assays. MIC determinations with *P. aeruginosa* C0214 in the presence of increasing concentrations of kanamycin A (from left to right) and of **10** **A**, **14n** **B** or **14c** **C** (from top to bottom). Cells of the table are stained with a linear color gradient according to OD_600nm_, from white at 0 to dark blue at the maximum value in the plate, indicated next to the color scale. The first column shows the effect of inhibitors in the absence of kanamycin A. The last column represents the negative control (medium with inhibitors but without bacteria). Growth curves are shown in Supplementary Fig. 4.

Since **10** had a more favorable uptake in *P. aeruginosa* (Fig. 7D), we monitored its effect on bacterial growth of the clinical *P. aeruginosa* strain C0214 (Fig. 8A). Increasing kanamycin concentrations progressively inhibited growth, with a MIC of 64 µg mL^**-1**^. The addition of **10** did not change the MIC value but retarded bacterial growth in a concentration-dependent manner, which was most evident for a kanamycin concentration of 32 µg mL^**-1**^ (Fig. 8A and Supplementary Fig. 4A). As expected, the addition of compound **10** alone did not affect bacterial growth (first column of the checkerboard assay and black curves in Supplementary Fig. 4A). Similar results were observed for compound **14n** and **14c** (Fig. 8B, C and Supplementary Fig. 4B, C). Overall, these results indicate a slight effect the APH inhibitor on the bacteria growth at the concentration below the MIC of kanamycin A. However, the impact of these compounds on bacterial growth remains moderate. This weak effect observed on bacteria may be due to the high intracellular concentration of ATP which competes with the APH inhibitors that have sub-micromolar affinities. Further optimization is therefore still needed to improve their efficacy.

## Conclusion

Developing targeted strategies to counter antibacterial resistance mechanisms is of utmost importance to restore efficacy of established antibiotics. In this context, inhibiting APHs represents a compelling approach to directly tackle a key antibiotic resistance mechanism. By concomitantly administering the aminoglycoside and APH inhibitor, antibiotic efficacy may be achieved by neutralizing one of the most relevant mechanism of bacterial resistance to aminoglycosides. Moreover, the co-administration of APH inhibitors with aminoglycosides might help to prevent the emergence of resistance development in strains that have not yet acquired APH activity.

Starting from the fragment screening hit **1**, we explored the pyrazole ring of **1**. This resulted in **10** or the triazole analogs **14a** or **14c**, which potently inhibited APH and displayed good ADME profile, incl. a more favorable uptake in multiple, particularly Gram-positive, bacterial strains. Moreover, several of the advanced compounds showed beneficial properties and enhanced the efficacy of the aminoglycoside kanamycin on a clinical *P. aeruginosa* strain expressing APH(3’)-IIb, although further optimization will be necessary before potential clinical application.

A broad profiling of human kinases revealed that sparing human kinases is in general possible. However, some human kinases such as Bike, AurA, or MSK_b displayed cross-reactivity, which must be addressed in future optimization studies.

In summary, we show that the bacterial kinases such as APHs are valid targets to tackle antibiotic resistance that can be reversed with drug-like molecules and are highly suitable for structure-based optimization.

## Methods

Synthetic procedures, NMR spectra and analytical data for the synthesized compounds, details of the X-ray crystallographic analyses, ADME and uptake studies, bacterial assays and kinase screening are given as Supplementary Information.

## Supplementary information

Supporting Information

Description of Additional Supplementary Files

Supplementary Data 1

nr-reporting-summary

## Data Availability

The authors declare that the data supporting the findings of this study are available within the paper and Supplementary Data. Raw data used to generate figures and tables are provided as supplementary material in a spreadsheet named Supplementary Data 1.
