# Supplementary material for: Targeting bacterial kinases as a strategy to counteract antibiotic resistance
Source: Commun Chem. 2025 Dec 4;8:390. doi: 10.1038/s42004-025-01794-7 (PMC12678819; doi:10.1038/s42004-025-01794-7)
Supplement: Supplementary file 1 — Supporting Information [file 42004_2025_1794_MOESM1_ESM.pdf]

Supplementary Information

Targeting bacterial kinases as a strategy to counteract antibiotic resistance

Vanessa Buffa [1], Julien Kowalewski [2], Guoman Qi [1], Robin Deutscher [1], Matijas Cica[1], Marion Richardoz [3], Mathilde Tomaszczyk [2], Andreas Krämer [4], Stefan Knapp [4], Catherine Dunyach-Remy [3], Katharina Rox [5,6], Jean-Francois Guichou [2], Corinne Lionne [2], and Felix Hausch [1,7,*]

[1] Department of Chemistry and Biochemistry Clemens-Schöpf-Institute, Technical University Darmstadt, Peter-Grünberg-Straße 4, 64287 Darmstadt, Germany.
[2] Centre de Biologie Structurale (CBS), 29 rue de Navacelles, University of Montpellier, CNRS UMR 5048, INSERM U1054, 34090 Montpellier, France.
[3] Virulence Bactérienne et Infections Chroniques, INSERM U1047, Department of Microbiology and Hospital Hygiene, Université de Montpellier, 30908 Nîmes, France.
[4] Institute of pharmaceutical Chemistry and Structural Genomics Consortium (SGC), Max von Lauestrasse 9, Frankfurt am Main, Germany.
[5] Department of Chemical Biology, Helmholtz Centre for Infection Research (HZI), Inhoffenstrasse 7, 38124 Braunschweig, Germany.
[6] German Center for Infection Research (DZIF), partner site Hannover-Braunschweig, Inhoffenstrasse 7, 38124 Braunschweig, Germany.
[7] Center for Synthetic Biology, Technical University Darmstadt, Germany.

*corresponding author: felix.hausch@tu-darmstadt.de

Table of contents

Chemistry ... **4**

General information ... **4**

Schemes ... **5**

General procedures ... **5**

Synthesis ... **6**

Azide building blocks ... **6**

(S)-2-Azidopropan-1-ol (**b1**) ... 6
(R)-2-Azidopropan-1-ol (**b2**) ... 7
(S)-1-Azidopropan-2-ol (**b3**) ... 8
(R)-1-Azidopropan-2-ol (**b4**) ... 8
(R)-3-azidopropane-1,2-diol (**m**) ... 9
(S)-3-azidopropane-1,2-diol (**n**) ... 9

Synthesis of the 7-azaindole precursor ... **9**

5-chloro-1H-pyrrolo[2,3-b]pyridine 7-oxide (**4**) ... 9
4-bromo-5-chloro-1H-pyrrolo[2,3-b]pyridine (**5**). ... 9
4-bromo-5-chloro-1-(phenylsulfonyl)-1H-pyrrolo[2,3-b]pyridine (**6**) ... 10

Synthesis of the pyrazole derivative 10 ... **10**

4-(5-chloro-1-(phenylsulfonyl)-1H-pyrrolo[2,3-b]pyridin-4-yl)but-3-yn-2-ol (**8**) ... 10
4-(5-chloro-1-(phenylsulfonyl)-1H-pyrrolo[2,3-b]pyridin-4-yl)but-3-yn-2-onem (**9**) ... 11
5-chloro-4-(3-methyl-1H-pyrazol-5-yl)-1H-pyrrolo[2,3-b]pyridine (**10**) ... 11

Synthesis of the triazole derivatives ... **12**

5-chloro-1-(phenylsulfonyl)-4-((trimethylsilyl)ethynyl)-1H-pyrrolo[2,3-b]pyridine (**12**) ... 12
5-chloro-4-ethynyl-1H-pyrrolo[2,3-b]pyridine (**13**) ... 12
5-chloro-4-(1H-1,2,3-triazol-4-yl)-1H-pyrrolo[2,3-b]pyridine（**14a**） ... 12
2-(4-(5-chloro-1H-pyrrolo[2,3-b]pyridin-4-yl)-1H-1,2,3-triazol-1-yl)ethan-1-ol (**14b**) ... 13
3-(4-(5-chloro-1H-pyrrolo[2,3-b]pyridin-4-yl)-1H-1,2,3-triazol-1-yl)propan-1-ol (**14c**) ... 13
2-(4-(5-chloro-1H-pyrrolo[2,3-b]pyridin-4-yl)-1H-1,2,3-triazol-1-yl)acetic acid (**14d**) ... 14
(4-(5-chloro-1H-pyrrolo[2,3-b]pyridin-4-yl)-1H-1,2,3-triazol-1-yl)methyl pivalate (**14e**) ... 14
5-chloro-4-(1-phenyl-1H-1,2,3-triazol-4-yl)-1H-pyrrolo[2,3-b]pyridine (**14f**) ... 14
5-chloro-4-(1-(p-tolyl)-1H-1,2,3-triazol-4-yl)-1H-pyrrolo[2,3-b]pyridine (**14g**) ... 15
5-chloro-4-(1-(4-fluorophenyl)-1H-1,2,3-triazol-4-yl)-1H-pyrrolo[2,3-b]pyridine (**14h**) ... 15
5-chloro-4-(1-(3-fluorophenyl)-1H-1,2,3-triazol-4-yl)-1H-pyrrolo[2,3-b]pyridine (**14i**) ... 15
5-chloro-4-(1-(pyridin-3-yl)-1H-1,2,3-triazol-4-yl)-1H-pyrrolo[2,3-b]pyridine (**14j**) ... 16
4-(1-benzyl-1H-1,2,3-triazol-4-yl)-5-chloro-1H-pyrrolo[2,3-b]pyridine (**14k**) ... 16
4-((4-(5-chloro-1H-pyrrolo[2,3-b]pyridin-4-yl)-1H-1,2,3-triazol-1-yl)methyl)benzonitrile (**14l**) ... 17
2-(4-(5-chloro-1H-pyrrolo[2,3-b]pyridin-4-yl)-1H-1,2,3-triazol-1-yl)acetamide (**24**) ... 17
Methyl 2-(4-(5-chloro-1H-pyrrolo[2,3-b]pyridin-4-yl)-1H-1,2,3-triazol-1-yl)acetate (**23**) ... 17
N-benzyl-2-(4-(5-chloro-1H-pyrrolo[2,3-b]pyridin-4-yl)-1H-1,2,3-triazol-1-yl)acetamide (**25**) ... 18
2-(4-(5-chloro-1H-pyrrolo[2,3-b]pyridin-4-yl)-1H-1,2,3-triazol-1-yl)-N-(4-methoxybenzyl)acetamide (**26**) ... 18
(S)-2-(4-(5-chloro-1H-pyrrolo[2,3-b]pyridin-4-yl)-1H-1,2,3-triazol-1-yl)propan-1-ol (**14b1**). ... 19
(R)-2-(4-(5-chloro-1H-pyrrolo[2,3-b]pyridin-4-yl)-1H-1,2,3-triazol-1-yl)propan-1-ol (**14b2**) ... 19
(S)-1-(4-(5-chloro-1H-pyrrolo[2,3-b]pyridin-4-yl)-1H-1,2,3-triazol-1-yl)propan-2-ol (**14b3**) ... 20
(R)-1-(4-(5-Chloro-1H-pyrrolo[2,3-b]pyridin-4-yl)-1H-1,2,3-triazol-1-yl)propan-2-ol (**14b4**) ... 20
(S)-4-(4-(5-Chloro-1H-pyrrolo[2,3-b]pyridin-4-yl)-1H-1,2,3-triazol-1-yl)butan-2-ol (**14c1**) ... 20
(R)-4-(4-(5-Chloro-1H-pyrrolo[2,3-b]pyridin-4-yl)-1H-1,2,3-triazol-1-yl)butan-2-ol (**14c2**) ... 21

(S)-3-(4-(5-Chloro-1H-pyrrolo[2,3-b]pyridin-4-yl)-1H-1,2,3-triazol-1-yl)-2-methylpropan-1-ol (**14c3**)...... 21
(R)-3-(4-(5-Chloro-1H-pyrrolo[2,3-b]pyridin-4-yl)-1H-1,2,3-triazol-1-yl)-2-methylpropan-1-ol (**14c4**)..... 22
(S)-3-(4-(5-chloro-1H-pyrrolo[2,3-b]pyridin-4-yl)-1H-1,2,3-triazol-1-yl)butan-1-ol (**14c5**) 22
(R)-3-(4-(5-chloro-1H-pyrrolo[2,3-b]pyridin-4-yl)-1H-1,2,3-triazol-1-yl)butan-1-ol (**14c6**).......................... 22
(R)-3-(4-(5-chloro-1H-pyrrolo[2,3-b]pyridin-4-yl)-1H-1,2,3-triazol-1-yl)propane-1,2-diol (**14m**)............... 23
(S)-3-(4-(5-chloro-1H-pyrrolo[2,3-b]pyridin-4-yl)-1H-1,2,3-triazol-1-yl)propane-1,2-diol (**14n**)................. 23

Synthesis of the amino-2-pyridine derivatives **25**
4-bromo-5-chloropyridin-2-amine (**16**) 25
5-chloro-4-(1-(tetrahydro-2H-pyran-2-yl)-1H-pyrazol-5-yl)pyridin-2-amine (**18**)........................... 25
5-chloro-4-(1H-pyrazol-5-yl)pyridin-2-amine (**19**) 25
4-(2-amino-5-chloropyridin-4-yl)but-3-yn-2-ol (**20**) 26
4-(2-amino-5-chloropyridin-4-yl)but-3-yn-2-one (**21**) 26
5-chloro-4-(3-methyl-1H-pyrazol-5-yl)pyridin-2-amine (**22**). 26

Biochemistry and Biology *28*

APH expression and purification and structure determination, Thermal shift assay, enzymatic assay, and Antibiotic susceptibility assays **28**

Uptake assays **28**
Bacterial strains 28
Cellular uptake assay 28

Determination of *in vivo* ADME properties **30**
ADME assays (plasma stability, metabolic stability and plasma protein binding) 30

Human kinase panel screening **30**
DSF-based selectivity screening against a curated kinase library 30
Supplementary Table 2. Full kinase panel screening of compounds **1**, **14c**, **14f**, **14m**, **14b3-4**, **14c2-4** and **24**. 31
Waterfall plot of compound **1** 31
Inhibition data of compound **1** for Erk-2 35

Bacterial Growth Assessment in the Presence of Antibiotics and Test Compounds **36**
Bacterial Strains and Culture Conditions 36
Preparation of compounds and antibiotic 36
Bacterial growth assay 36
Bacterial growth curve for compound **10**, **14n** and **14c**. 37

X-ray crystallography and structure refinement *38*

Additional figures **38**
Compound **1** in complex with Erk-2. 38
Compound **19** in complex with APH(2”)-IVa.. 38

Supplementary Tables 3-10: Data collection and refinement statistics **39**

HPLC profiles *79*

Supplementary references *85*

Chemistry

General information

Reactions

Air and water-sensitive reactions were performed under an argon atmosphere with commercially available dry solvents. All commercially available chemicals and solvents were used as received. All reactions were monitored with TLC performed on precoated aluminum plates with a fluorescence indicator from Merck (silica gel 60 F254) or LCMS.

Analytical HPLC

Analytical LC–MS (liquid chromatography–mass spectrometry) measurements were performed on an Agilent 1260 Infinity II System consisting of a 1260 Infinity II flexible pump, a vial sampler, a multicolumn thermostat fitted with a Poroshell 120 3 mm × 150 mm, 2.7 µm EC-C18 column or a Poroshell 120 50 mm × 2.1 mm, 1.9 µm EC-C18, and a diode array detector connected to a 6125B MSD single quadrupole detector. Eluents were 0.1% formic acid in water (solvent A) and 0.1% formic acid in acetonitrile (solvent B).

Purification

Chromatographic separations were performed by manual flash chromatography on silica (SiO_2, particle size 4 - 63 µm) or by automated flash chromatography on a Biotage Isolera with Biotage ® Sfär Silica HC D columns. Eluents are specified at the respective experiments. All final compounds had a purity >95% as determined by reverse phase HPLC (detection at 220 nm), unless otherwise noted. The only exceptions were compounds **14f**, **14h**, **24**, **26** and **19** which had purities of 83%, 90%, 80%, 93% and 92%, respectively.

Preparative HPLC

Reverse-phase purifications were performed with an Interchim puriFlash 5.250 system fitted with a Luna® 5 µm C18(2) 100 Å, LC column (250 x 21.2 mm). Eluents were 0.1% TFA in water (solvent A) and 0.1% TFA in acetonitrile (solvent B).

HR-MS

HRMS measurements were obtained by the Mass Spectrometry Department of the Technical University of Darmstadt using a Bruker Daltonics Impact II mass spectrometer (quadrupole time-of- flight).

NMR

All ^{1}H and ^{13}C-NMR spectra have been measured at the NMR facility at the department of chemistry at Technische Universit‰t Darmstadt (TUD) on a Bruker AC 300, AR300 or DRX500. Chemical shifts for ^{1}H and ^{13}C were given in ppm (δ). Deuterated chloroform ($CDCl_3$) and dimethyl sulfoxide (DMSO- *d*6) were used as solvents, and the spectra were calibrated according to their corresponding peak. The multiplicities were abbreviated as follows: singlet (s), doublet (d), triplet (t), quartet (q), pentet (p), doublet of doublets (dd), (doublet of triplets (dt), doublet of quartets (dq), doublet of doublets of triplets (ddt), triplet of doublets (td), quartet of doublets (qd), pentet of doublets (pd), doublet of doublets of doublets (ddd), doublet of triplets of doublets (dtd), quartet of doublets of doublets (qdd) and multiplet (m).

Schemes

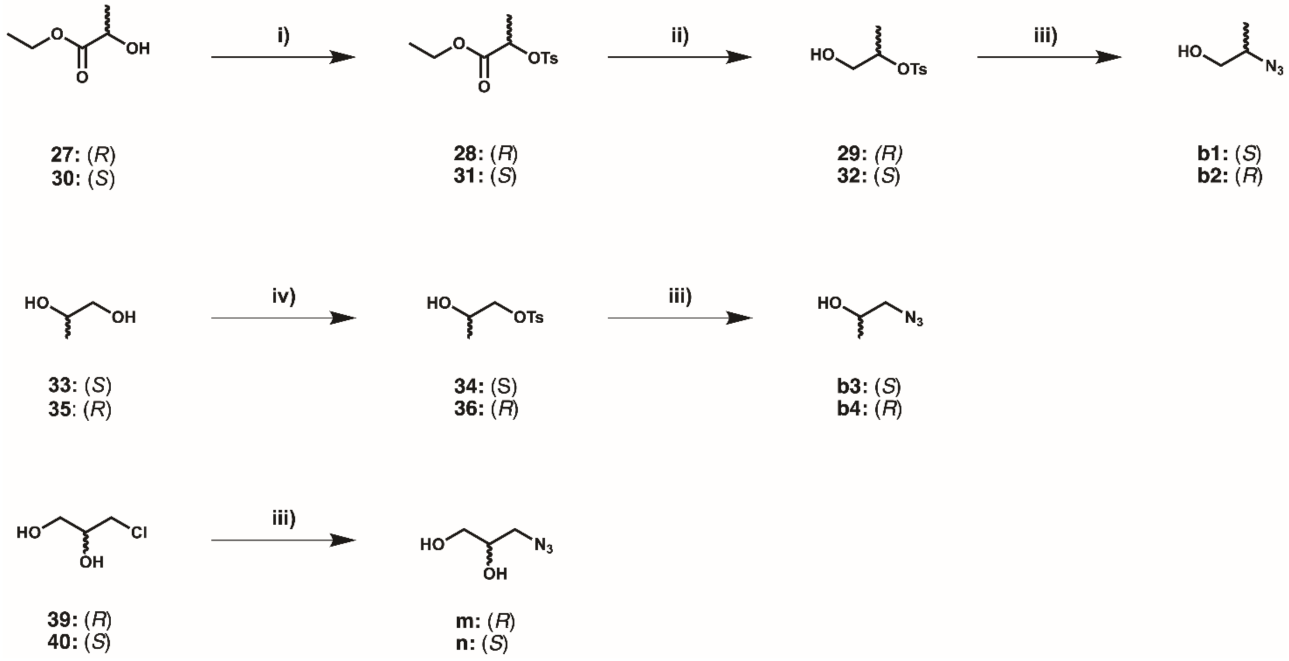

Supplementary Scheme 1: Synthesis of Azido Building Blocks. Reagents and conditions: i) TsCl, Et_3N, DMAP, DCM, 0°C to rt; ii) $NaBH_4$, LiCl, EtOH/THF, -5°C to rt; iii) NaN_3, DMF, 80°C; iv)TsCl, pyridine, MeCN, 0°C to rt.

General procedures

General Procedure A for the Synthesis of 7-azaindole triazole derivatives.

Under an inert atmosphere **13** (1.00 equiv) was suspended in a 1:1 mixture of water/t-BuOH (c = 0.5 M). Then, the corresponding azide (1.00 – 2.00 equiv), sodium ascorbate (1.0 M, 0.1 – 0.5 equiv) and $CuSO_4(H_2O)_5$ (0.5 M, 0.05 – 0.20 equiv) were added and the reaction mixture was stirred at room temperature. Upon completion, the reaction mixture was quenched with 0.2 mL - 0.5 mL of a 10% ammonia solution, diluted with H_2O and extracted with DCM. The organic phases were combined, dried over $MgSO_4$, filtered and the solvent was removed under reduced pressure. The resulting crude product was purified by flash chromatography or preparative HPLC.

General procedure B for the sythesis of azides b1-b4 and m-n.

The corresponding tosyl-alcohol/chloride (1.00 equiv) and sodium azide (2.00 - 4.00 equiv) were dissolved in DMF (c = 0.5 M) and the reaction mixture was stirred overnight at 70°C. Afterwards, the reaction mixture was diluted with H_2O (10 mL) and extracted with Et_2O (3 x 10 mL). The combined organic layers were dried over $MgSO_4$ and the solvent was removed under reduced pressure.

General procedure C for compounds 34 and 36.

The corresponding diol (1.00 equiv) was dissolved in MeCN (c = 0.5 M) and the reaction mixture wad cooled to 0°C before adding pyridine (1.20 equiv). After stirring for 15 minutes, tosylchloride (1.00 equiv) was added and the reaction mixture was stirred at 0°C for 5 h. The reaction mixture was quenched with 1 M HCl solution and the aqueous layer was extracted with Et_2O (3 x 50 mL). The combined organic layers were dried over

$MgSO_4$ and the solvent was removed under reduced pressure. The crude product was purified by flash column chromatography.

General procedure D for compounds 28 and 31.

The corresponding commercially available ester (1.00 equiv), Et_3N (2.00 equiv) and DMAP (0.20 equiv) were dissolved in DCM (c = 0.5 M). The solution was cooled to 0°C before adding tosylchloride (1.40 equiv). The reaction mixture was stirred at room temperature overnight. Upon completion, the reaction mixture was filtered through celite and washed with DCM. The organic phase was dried over $MgSO_4$, the solvent was removed under reduced pressure and the product was purified by flash column chromatography.

General procedure E for compounds 29 and 32.

The tosyl ester (1.00 equiv) and LiCl (3.00 equiv) were dissolved in EtOH/THF (2:1 mixture, v/v, c = 0.13). The solution was cooled to -5°C (ice/NaCl) before adding sodium borohydride portionwise (3.00 equiv). The ice bath was removed and the reaction mixture was stirred for three days at room temperature. Afterwards, acetone (50 mL) was added and the solution was stirred for 30 min. Then, chloroform (450 mL) and $MgSO_4$ was added and the mixture stirred for another hour. After filtering over celite, the solvent was removed under reduced pressure and the crude mixture was purified with flash column chromatography.

Synthesis

Compounds **3**, **7**, **11**, **15**, **27**, **30**, **33**, **35**, **37**, **38**, **39**, **40** and azides **b-l** were commercially available.

Azide building blocks

Azides **c1**, **c2**, **c3**, **c4**, **c5** and **c6** were synthesized following procedures indicated in [1].

(S)-2-Azidopropan-1-ol (**b1**)

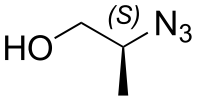

b1 was synthesized following general procedure B starting from **29** (1.15 g, 5.00 mmol, 1.00 equiv) with NaN_3 (1.2 g, 20 mmol, 4.00 equiv) in DMF (10 mL). The crude product was used without further purification.

(R)-1-Hydroxypropan-2-yl 4-methylbenzenesulfonate **(29)**

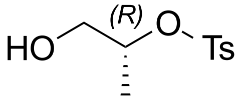

29 was synthesized following general procedure E starting from **28** (10 g, 36.69 mmol, 1.00 equiv) with LiCl (4.67 g, 110 mmol, 3.00 equiv) and $NaBH_4$ (4.16 g, 110 mmol, 3.00 equiv) in EtOH/THF (2:1 mixture, v/v, 282 mL). The crude product was purified with flash column chromatography (CH/EA = 0-100%) to give the title compound **29** (2.94 g, 35 %) as a colorless oil. **TLC**: (EA:CH = 1:1) R_f = 0.34. **^{1}H NMR:** (500 MHz, $CDCl_3$): δ 7.80 (d, J = 8.3 Hz, 2H, tosyl Ar-CH), 7.33 (d, J = 8.1 Hz, 2H, tosyl Ar-CH), 4.66 (pd, J = 6.4, 3.6 Hz, 1H, OH), 3.65 – 3.56 (m, 2H, CH_2), 2.43 (s, 3H, Ar-CH_3), 2.24 (s, 1H, CH), 1.22 (d, J = 6.5 Hz, 3H, CH_3). **^{13}C NMR:** (126 MHz, $CDCl_3$): δ = 145.0, 133.9, 129.9, 127.9, 80.7, 65.6, 21.7, 17.0. **HPLC:** (5-100% Solvent B, 2 min): R_t = 1.65 min, purity (220 nm) >99%.

Ethyl (R)-2-(tosyloxy)propanoate **(28)**

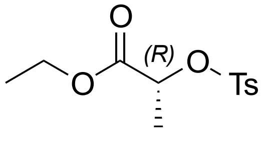

28 was synthesized following general procedure D starting from ethyl (R)-2-hydroxypropanoato **27** (5.91 g, 50 mmol, 1.00 equiv), with Et_3N (13.86 mL, 100 mmol, 2.00 equiv), DMAP (1.22 g, 10 mmol, 0.20 equiv) and tosylchloride (11.44 g, 60 mmol, 1.20 equiv) in DCM (100 mL). The crude product was purified by flash column chromatography (CH/EA = 0-100%) to obtain the title compound **28** (9.99 g, 73 %) as a colorless oil. **TLC:** (CH/EA = 2:1, v/v) R_f = 0.58. **^{1}H NMR:** (300 MHz, $CDCl_3$): δ 7.90 – 7.73 (m, 2H, tosyl Ar-CH), 7.41 – 7.24 (m, 2H, tosyl Ar-CH), 4.91 (q, J = 6.9 Hz, 1H, CH), 4.10 (qd, J = 7.1, 0.8 Hz, 2H, CH_2), 2.43 (s, 3H, tosyl Ar-CH_3), 1.49 (d, J = 6.9 Hz, 3H, CH_3), 1.19 (t, J = 7.1 Hz, 3H, CH_3). **^{13}C NMR:** (75 MHz, $CDCl_3$): δ 169.1, 145.1, 133.5, 129.9, 128.1, 74.3, 61.9, 21.7, 18.5, 14.0. **HPLC:** (5-100% Solvent B, 2 min): R_t = 2.00 min, purity (220 nm) = 98%.

(R)-2-Azidopropan-1-ol (**b2**)

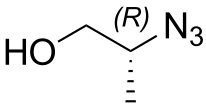

b2 was synthesized following general procedure B starting from (S)-1-hydroxypropan-2-yl 4-methylbenzenesulfonate **46** (1.15 g, 5.00 mmol, 1.00 equiv) with NaN_3 (1.2 g, 20 mmol, 4.00 equiv) in DMF (10 mL). The crude product was used without further purification.

(S)-1-Hydroxypropan-2-yl 4-methylbenzenesulfonate (**32**)

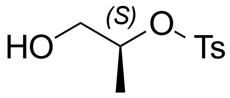

32 was synthesized following general procedure E starting from **31** (8.90 g, 32.68 mmol, 1.00 equiv) with LiCl (4.16 g, 98 mmol, 3.00 equiv) and $NaBH_4$ (3.71 g, 98mmol, 3.00 equiv) in EtOH/THF (2:1 mixture, v/v, 250 mL). The crude product was purified with flash column chromatography (CH/EA = 0-100%) to give the title compound **32** (2.22 g, 30%) as a colorless oil. **TLC**: (CH/EA = 1:1, v/v) R_f = 0.36. **^{1}H NMR:** (300 MHz, $CDCl_3$): δ = 7.83 – 7.77 (m, 2H, tosyl Ar-CH), 7.37 – 7.31 δ (m, 2H, tosyl Ar-CH), 4.68 (qdd, J = 6.5, 5.7, 3.9 Hz, 1H, CH), 3.64 – 3.59 (m, 2H, CH_2), 2.44 (s, 3H, tosyl Ar-CH_3), 2.11 (s, 1H, OH), 1.23 (d, J = 6.5 Hz, 3H, CH_3). **^{13}C NMR:** (75 MHz, $CDCl_3$): δ = 145.0, 134.0, 130.0, 127.9, 80.8, 65.7, 21.8, 17.0. HPLC (5-100% Solvent B, 2 min): R_t = 1.64 min, purity (220 nm) = 99%.

Ethyl (S)-2-(tosyloxy)propanoate (**31**)

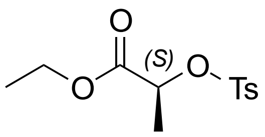

31 was synthesized following general procedure D starting from ethyl (S)-2-hydroxypropanoate **30** (5.91 g, 50 mmol, 1.00 equiv), with Et_3N (13.86 mL, 100 mmol, 2.00 equiv), DMAP (1.22 g, 10 mmol, 0.20 equiv) and

tosylchloride (11.44 g, 60 mmol, 1.20 equiv) in DCM (100 mL). The crude product was purified by flash column chromatography (CH/EA = 0-100%) to obtain the title compound **31** (8.92 g, 66%) as a colorless oil. **TLC:** (CH/EA = 3:1) R_f = 0.58. **^{1}H NMR:** (300 MHz, $CDCl_3$): δ 7.80 (d, J = 8.3 Hz, 2H, Tosyl Ar-CH), 7.33 (dt, J = 8.0, 0.7 Hz, 2H, Tosyl Ar-CH), 4.91 (q, J = 6.9 Hz, 1H, CH), 4.10 (qd, J = 7.2, 0.8 Hz, 2H, CH_2), 2.43 (s, 3H, Tosyl Ar-CH_3), 1.49 (d, J = 6.9 Hz, 3H, CH_3), 1.19 (t, J = 7.1 Hz, 3H, CH_3). **^{13}C NMR:** (75 MHz, $CDCl_3$): δ = 169.0, 145.0, 133.5, 129.8, 128.0, 74.2, 61.8, 21.6, 18.38, 13.9. **HPLC:** (5-100% Solvent B, 2 min): R_t = 2.03 min, purity (220 nm) = 98%.

(S)-1-Azidopropan-2-ol (**b3**)

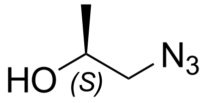

b3 was synthesized following general procedure B starting from **34** (677 mg, 2.94 mmol, 1.00 equiv) with NaN_3 (765 mg, 11.76 mmol, 4.00 equiv) in DMF (10 mL). The crude product was used without further purification.

(S)-2-Hydroxypropyl 4-methylbenzenesulfonate (**34**)

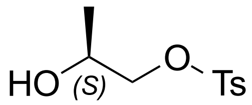

34 was synthesized following general procedure C starting from (S)-(-)1,2-propanediol **33** (0.73 mL, 10 mmol, 1.00 equiv), with pyridine (0.969 mL, 12 mmol, 1.20 equiv) and tosylchloride (1.91 g, 10 mmol, 1.00 equiv) in 20 mL of MeCN. The crude product was purified by flash chromatography (CH/EA = 0-100%) to obtain the title compound **34** (1.0 g, 43%). **TLC:** (CH/EA = 1:1, v/v) R_f = 0.40. **^{1}H NMR:** (300 MHz, $CDCl_3$): δ 7.83 – 7.77 (m, 2H, Tosyl Ar-CH), 7.39 – 7.32 (m, 2H, Tosyl Ar-CH), 4.12 – 4.01 (m, 1H, OH), 3.98 (dd, J = 10.1, 3.2 Hz, 1H, Ha of CH_2), 3.88 – 3.81 (m, 1H, Hb of CH_2), 2.44 (d, J = 0.9 Hz, 3H, Tosyl Ar-CH_3), 2.17 (s, 1H, CH), 1.15 (d, J = 6.4 Hz, 3H, CH_3). **^{13}C NMR:** (75 MHz, $CDCl_3$): δ 145.2, 132.8, 130.1, 128.1, 74.9, 65.7, 21.8, 18.6. **HPLC:** (5-100% Solvent B, 2 min): R_t = 1.65 min, purity (220 nm) > 99%.

(R)-1-Azidopropan-2-ol (**b4**)

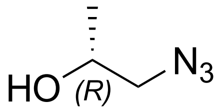

b4 was synthesized following general procedure B starting from **36** (659 mg, 2.86 mmol, 1.00 equiv) with NaN_3 (744 mg, 11.44 mmol, 4.00 equiv) in DMF (10 mL). The crude product was used without further purification.

(R)-2-Hydroxypropyl 4-methylbenzenesulfonate (**36**)

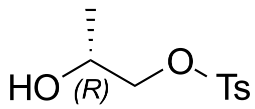

36 was synthesized following general procedure C starting from (R)-(-)1,2-propanediol **35** (0.73 mL, 10 mmol, 1.00 equiv), with pyridine (0.969 mL, 12 mmol, 1.20 equiv) and tosylchloride (1.91 g, 10 mmol, 1.00 equiv) in

20 mL of MeCN. The crude product was purified by flash chromatography (CH/EA = 0-100%) to obtain the title compound **36** (0.98 g, 43 %) as a colorless oil. **TLC:** (CH/EA = 1:1, v/v) R_f = 0.4. **^{1}H NMR:** (300 MHz, $CDCl_3$): δ 7.81 (d, J = 8.3 Hz, 2H, Tosyl Ar-CH), 7.37 (dt, J = 8.0, 0.7 Hz, 2H, Tosyl Ar-CH), 4.17 – 4.03 (m, 1H, OH), 4.00 (dd, J = 10.0, 3.1 Hz, 1H, Ha of CH_2), 3.86 (dd, J = 9.9, 7.1 Hz, 1H, Ha of CH_2), 2.47 (s, 3H, Tosyl Ar-CH_3), 2.22 (d, J = 2.7 Hz, 1H, CH), 1.17 (d, J = 6.4 Hz, 3H, CH_3). **^{13}C NMR:** (75 MHz, $CDCl_3$): δ 145.1, 132.7, 130.0, 128.0, 74.8, 65.6, 21.7, 18.5. **HPLC:** (5-100% Solvent B, 2 min): R_t = 1.65 min, purity (220 nm) > 99%.

(R)-3-azidopropane-1,2-diol (**m**)

m was synthesized following general procedure B starting from **39** (5.50 g, 50.00 mmol, 1.00 equiv) with NaN_3 (6.50 g, 100.00 mmol,2.00 equiv) in DMF (60 mL). The crude product was used without further purification.

(S)-3-azidopropane-1,2-diol (**n**)

n was synthesized following general procedure B starting from **40** (1.10 g, 10.00 mmol, 1.00 equiv) with NaN_3 (1.30 g, 20.00 mmol,2.00 equiv), NaOH (0.04 g, 1.00 mmol, 0.10 equiv) in DMF (20 mL). The crude product was used without further purification.

Synthesis of the 7-azaindole precursor

5-chloro-1H-pyrrolo[2,3-b]pyridine 7-oxide (**4**)

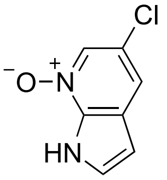

5-chloro-1*H*-pyrrolo[2,3-*b*]pyridine **3** (9.0 g, 58.99 mmol, 1.00 equiv) was dissolved in 1,2 DME and *n*-hexane (2:1, 450 mL). Then m-chloroperoxybenzoic acid (77 wt %, 21.15 g, 94.38 mmol, 1.60 equiv) was added. The reaction mixture was stirred at room temperature for 26 h then kept at -20°C overnight. The resulting solid was washed with n-hexane (2 × 20 mL) and air dried. Afterwards, the solid was dissolved in a saturated aqueous solution of $NaHCO_3$ (80 mL) and DCM (100 mL). The aqueous phase was extracted with DCM (3 × 100 mL). The resulting organic phases were combined and dried over $MgSO_4$, and the solvent was removed under reduced pressure. The title compound **4** was obtained as a white solid (8.11 g, 82%) and was used without further purification. **^{1}H NMR:** (300 MHz, DMSO-*d*6) δ 12.72 (s, 1H, NH), 8.36 (d, J = 1.6 Hz, 1H, Ar-CH adjacent to Cl), 7.78 (d, J = 1.6 Hz, 1H, Ar-CH), 7.56 – 7.49 (m, 1H, Ar-CH adjacent to NH), 6.55 (d, J = 2.9 Hz, 1H, Ar-CH). **^{13}C NMR:** (75 MHz, DMSO-*d*6) δ 137.5, 130.2, 127.9, 123.4, 121.1, 119.4, 102.1. **HPLC:** (5-100% Solvent B, 2 min): R_t = 1.19 min, purity = 88%.

4-bromo-5-chloro-1H-pyrrolo[2,3-b]pyridine (**5**).

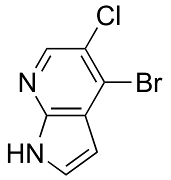

4 (8.10 g, 48 mmol, 1.00 equiv) and tetramethylammonium bromide (11.10 g, 72 mmol, 1.50 equiv) were suspended in dry DMF (200 mL). Methanesulfonic anhydride (16.74 g, 96 mmol, 2.00 equiv) was dissolved in dry DMF (20 mL) and slowly added via cannula while maintaining the internal temperature at -15°C to -10°C. The reaction mixture was warmed up to room temperature and stirred overnight. After 24 h, the reaction mixture was quenched with 600 mL iced H_2O, the pH of solution was adjusted to 7 with a 1.0 M solution of NaOH and the reaction mixture was stored at 4°C overnight. The reaction mixture was filtered, the precipitate was collected, washed with iced H_2O and dried under reduced pressure. The resulting crude product was purified by flash column chromatography 4 times (CH/EA = 0-20%) to provide the title compound **5** (4.85 g, 44%) as a light brown solid. **TLC:** (DCM/MeOH = 10:1, v/v), R_f = 0.50. **^{1}H NMR:** (300 MHz, DMSO-*d6*) δ 12.26 (s, 1H, NH), 8.29 (s, 1H, Ar-CH adjacent to Cl), 7.63 (t, 1H, Ar-CH adjacent to NH), 6.44 (m, 1H, Ar-CH). **^{13}C NMR:** (75 MHz, DMSO-*d6*) δ 146.0, 141.3, 122.7, 122.6, 122.2, 100.2. **HPLC:** (5-100% Solvent B, 2 min): R_t = 1.97 min, purity = 96%.

4-bromo-5-chloro-1-(phenylsulfonyl)-1H-pyrrolo[2,3-b]pyridine (**6**)

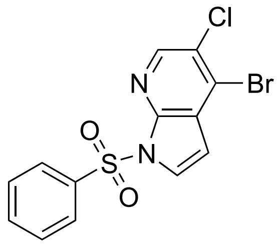

NaH (60% suspension in mineral oil, 1.51 g, 37.71 mmol, 1.80 equiv) was added to a solution of **5** (4.85 g, 20.95 mmol, 1.00 equiv) in DMF (80 mL) at 0°C. After 10 min, benzenesulfonyl chloride (4.02 mL, 31.43 mmol, 1.50 equiv) was added dropwise and the reaction mixture was warmed to room temperature. After 20 hours, EA (100 mL) and H_2O (150 mL) were added. The precipitate was filtered and dried under reduced pressure. The aqueous phase was extracted three times with EA. The combined organic layers were washed with H_2O, brine and dried over $MgSO_4$. The solvent was removed under reduced pressure and the crude product was purified by flash column chromatography (CH/EA, 0-100%) to provide the title compound **6** (3.58 g, 46%) as a white/yellow solid. **TLC:** (CH/EA = 3:1, v/v), R_f = 0.58. **^{1}H NMR:** (500 MHz, $CDCl_3$) δ 8.37 (d, J = 1.4 Hz, 1H, Ar-CH adjacent to Cl), 8.17 (d, J = 7.9 Hz, 2H, phenyl CH), 7.83 – 7.80 (m, 1H, phenyl CH), 7.61 (t, J = 7.5 Hz, 1H, Ar-CH adjacent to NH), 7.51 (t, J = 7.7 Hz, 2H, phenyl CH), 6.64 (dd, J = 4.1, 1.4 Hz, 1H, Ar-CH). **^{13}C NMR:** (126 MHz, $CDCl_3$) δ 144.5, 144.2, 138.0, 134.6, 129.3, 128.4, 128.3, 128.1, 125.7, 125.4, 105.6. **HPLC** :(5-100% Solvent B, 2 min): R_t = 2.351 min, purity = 98%.

Synthesis of the pyrazole derivative 10

4-(5-chloro-1-(phenylsulfonyl)-1H-pyrrolo[2,3-b]pyridin-4-yl)but-3-yn-2-ol (**8**)

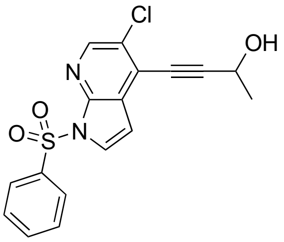

6 (100.0 mg, 0.269 mmol, 1.0 equiv) was dissolved in a solution of DMF/TMEDA (1:1 mixture v/v, 10 mL) and degassed for 15 minutes. Then, **7** (94.3 mg, 2.7 mmol, 10 eq.), Pd(dppf)Cl_2 (21.97 mg, 0.027 mmol 0.1 equiv) and CuI (25.62 mg, 0.135 mmol, 0.5 equiv) were added and the reaction mixture was stirred at 90 ºC for 21 h.

Upon completion, the reaction mixture was diluted with HCl (1.0 M, 3 mL) and H_2O (10 mL) and extracted with DCM (4 x 50 mL). The organic phase were combined, washed with brine (1 x 50 mL), dried over $MgSO_4$, filtered, and concentrated under reduced pressure. The resulting crude product was purified by flash column chromatography (CH/EA = 0-80%) to give the title compound **8** as a white solid (77.7 mg, 80%). **TLC:** R_f = 0.53 (CH/:EA = 1:1).^{1}**H NMR:** (500 MHz, $CDCl_3$) δ 8.34 (d, J = 1.3 Hz, 1H, Ar-CH adjacent to Cl), 8.14 – 8.10 (m, 2H, phenyl CH), 7.73 (dd, J = 4.0, 1.3 Hz, 1H, Ar-CH adjacent to NH), 7.57 (td, J = 7.5, 1.4 Hz, 1H, phenyl CH), 7.50 – 7.44 (m, 2H, phenyl CH), 6.64 (dd, J = 4.0, 1.3 Hz, 1H, Ar-CH), 4.57 (q, J = 6.7 Hz, 1H, CH), 2.58 – 2.56 (m, 1H, OH), 1.45 (dd, J = 6.6, 1.4 Hz, 3H, CH_3). ^{13}**C NMR:** (126 MHz, $CDCl_3$) δ 145.0, 143.9, 137.9, 134.5, 129.3, 128.3, 128.3, 128.1, 125.0, 123.2, 104.9, 104.2, 81.4, 58.7, 24.0. **HPLC:** (5-100% Solvent B, 2 min): R_t = 2.01 min, purity = 88%.

4-(5-chloro-1-(phenylsulfonyl)-1H-pyrrolo[2,3-b]pyridin-4-yl)but-3-yn-2-onem (**9**)

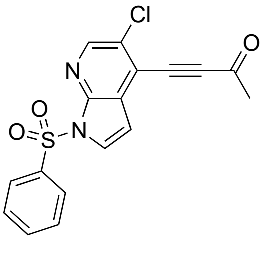

8 (46.2 mg, 0.128 mmol, 1.0 equiv) was dissolved in dry DCM (15 mL). The reaction was cooled down to 0 ºC. Then DMP (217.02 mg, 0.512 mmol, 4.0 equiv) was added under argon. The reaction mixture was stirred at 0 ºC for 2 h. Precipitate was filtered off and washed with DCM. Filtrate was extracted with DCM and saturated $NaHCO_3$ solution. The organic phase was dried over $MgSO_4$, filtered, and removed solvents under reduced pressure. The resulting crude product was purified by flash chromatography (CH/EA = 0-100%) to obtain the title compound **9** as a white solid (23.0 mg, 50%). **TLC:** (CH/EA = 1:1, v/v), R_f = 0.55. ^{1}**H NMR:** (500 MHz, $CDCl_3$) δ 8.43 (d, J = 1.2 Hz, 1H, Ar-CH adjacent to Cl), 8.16 (dt, J = 8.7, 1.3 Hz, 2H, phenyl CH), 7.85 (dd, J = 4.1, 1.2 Hz, 1H, Ar-CH adjacent to NH), 7.65 – 7.57 (m, 1H, phenyl CH), 7.54 – 7.47 (m, 2H, phenyl CH), 6.72 (dd, J = 4.0, 1.2 Hz, 1H, Ar-CH), 2.52 (d, J = 1.3 Hz, 3H, CH_3). ^{13}**C NMR:** (126 MHz, $CDCl_3$) δ 183.7, 145.1, 144.0, 137.9, 134.7, 129.4, 129.4, 129.3, 128.3, 125.3, 120.4, 104.4, 97.2, 81.1, 33.1. **HPLC:** (5-100% Solvent B, 2 min): R_t = 2.16 min, purity = 90%.

5-chloro-4-(3-methyl-1H-pyrazol-5-yl)-1H-pyrrolo[2,3-b]pyridine (**10**)

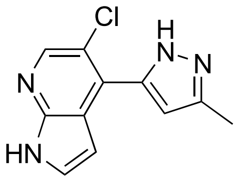

9 (18.0 mg, 0.050 mmol, 1.0 equiv) was suspended in EtOH (3 mL). Then $NH_2NH_2 \cdot H_2O$ (3.77 mg, 0.075 mmol, 1.5 equiv) was added and the reaction mixture was stirred at 80ºC. After 1 h, H_2SO_4 (2.5 mg, 0.025 mmol, 0.5 equiv) was added to reaction mixture. After 29 h, the reaction was cooled down to rt and NaOH (16.0 mg, 0.351 mmol, 8.0 eq.) was added. After 45 h, 15 mL 1-BuOH and 5 mL water were added. The organic phase was separated and removed solvents under reduced pressure. The crude product was purified with preparative HPLC (ACN/H_2O, 5:95 → ACN/H_2O, 4:6) to obtain the title compound **10** as a white solid (7.68 mg, 66%). **TLC:** (CH:EA = 1:1, v/v), R_f = 0.15. ^{1}**H NMR:** (500 MHz, DMSO-*d6*) δ 11.86 (s, 1H, NH), 8.26 (s, 1H, Ar-CH adjacent to Cl), 7.52 (t, J = 3.0 Hz, 1H, Ar-CH adjacent to NH), 6.73 (dd, J = 3.7, 1.9 Hz, 1H, Ar-CH), 6.62 (s, 1H, Ar-CH, pyrazole), 2.33 (s, 3H, CH_3). ^{13}**C NMR:** (126 MHz, DMSO-*d6*) δ 158.9, 158.6, 147.7, 142.7, 131.5, 128.1, 120.4, 119.6, 106.5, 102.0, 11.2. **HPLC:** (5-100% Solvent B, 2 min): R_t = 1.57 min, purity = 100%.

Synthesis of the triazole derivatives

5-chloro-1-(phenylsulfonyl)-4-((trimethylsilyl)ethynyl)-1H-pyrrolo[2,3-b]pyridine (**12**)

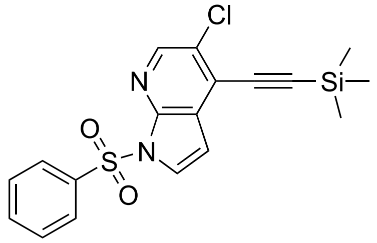

6 (1.0 g, 2.69 mmol, 1.00 equiv) was dissolved in TMEDA/DMF (2:1 mixture, v/v, 50 mL). The solution was degassed with argon for 30 min before adding CuI (256 mg, 1.35 mmol, 0.50 equiv), Pd(dppf)Cl_2 (439 mg, 0.54 mmol, 0.20 equiv) and trimethylsilylacetylene **11** (5.6 mL, 40.36 mmol, 15 equiv). The reaction mixture was stirred for 1 hour at 80°C. Afterwards, 300 mL of 1 M HCl and 600 mL of DCM were added. The aqueous phase was extracted with DCM (2 x 100 mL), the combined organic layers were washed brine (3 x 100 mL) and dried over $MgSO_4$. The solvent was removed under reduced pressure and the crude product was purified by flash column chromatography (CH/EA = 0-100%) to yield the title compound **12** (829 mg, 79%) as a white solid. **TLC:** (CH/EA = 3:1, v/v) R_f = 0.58. **^{1}H NMR:** (500 MHz, $CDCl_3$) δ 8.37 (d, *J* = 1.2 Hz, 1H, Ar-CH adjacent to Cl), 8.14 (d, *J* = 7.9 Hz, 2H, orto phenyl CH), 7.76 (dd, *J* = 4.1, 1.2 Hz, 2H, meta phenyl CH), 7.61 – 7.56 (m, 1H, para phenyl CH), 7.49 (t, *J* = 7.7 Hz, 1H, Ar-CH adjacent to NH), 6.69 (dd, *J* = 4.0, 1.3 Hz, 1H, Ar-CH), 0.30 (d, *J* = 1.3 Hz, 9H, TMS CH_3). **^{13}C NMR:** (126 MHz, $CDCl_3$) δ 145.2, 144.0, 138.1, 134.5, 129.3, 128.5, 128.2, 128.1, 125.2, 123.6, 109.4, 105.1, 96.8, -0.2. **HPLC:** (30-100% Solvent B, 2 min): R_t = 2.450 min, purity = 91%.

5-chloro-4-ethynyl-1H-pyrrolo[2,3-b]pyridine (**13**)

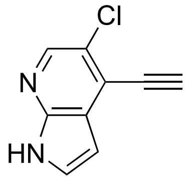

12 (980 mg , 2.52mmol, 1.00 equiv) was dissolved in MeOH (60 mL). Afterwards, K_2CO_3 (731 mg, 5.29 mmol, 2.10 equiv) was added and the reaction mixture was stirred at room temperature overnight. The reaction mixture was diluted with H_2O (50 mL) and DCM (100 mL). The aqueous phase was extracted with DCM (3 x 50 mL) and the combined organic layers were dried over $MgSO_4$. The solvent was removed under reduced pressure and the crude product was purified with flash column chromatography (CH/EA = 0-50%) to yield the title compound **13** (325 mg, 73%) as a brown solid. **TLC:** (DCM/MeOH = 98:2, v/v) R_f = 0.38. **^{1}H NMR:** (500 MHz, DMSO-*d*6): δ 12.13 (s, 1H, NH), 8.29 (s, 1H, Ar-CH adjacent to Cl), 7.67 (q, J = 3.0, 2.3 Hz, 1H, Ar-CH adjacent to NH), 6.50 (dt, J = 3.5, 1.7 Hz, 1H, Ar-CH), 5.04 (s, 1H, CH). **^{13}C NMR:** (126 MHz, DMSO-*d*6) δ 146.2, 140.7, 129.1, 123.4, 121.5, 119.3, 98.8, 91.5, 77.0. **HPLC:** (5-100% Solvent B, 2 min): R_t = 1.764 min, purity (220 nm): > 99%.

5-chloro-4-(1H-1,2,3-triazol-4-yl)-1H-pyrrolo[2,3-b]pyridine（**14a**）

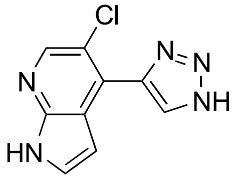

13 (15.0 mg, 0.084 mmol, 1.0 eq.) and NaN_3 (8.23 mg, 0.126 mmol, 1.5 eq.) were suspended in H_2O/*t*-BuOH/DMSO (1:1:0.35 mixture v/v/v, 1.6 mL,). Then, sodium ascorbate (0.5 mmol, 0.5 mL of a freshly prepared 1.0 M solution in H_2O) was added, followed by $CuSO_4(H_2O)_5$ (0.1 mmol, in 0.2 mL water). The reaction mixture was stirred at rt. After 22 h, sodium ascorbate (1.00 equiv) and $CuSO_4(H_2O)_5$ (1.00 equiv) were added. After 7 days, the reaction was diluted with water (2 mL) and cooled in an ice bath. The resulting yellow precipitate was collected by filtration and washed with cold water three times. The crude product was purified by preparative HPLC (ACN/H_2O, 5:95 → ACN/H_2O, 4:6) to yield the desired compound **14a** (2.2 mg, 12%) as a yellow solid. **^{1}HNMR:** (500 MHz, DMSO-*d6*) δ 15.40 (s, 1H, NH triazole), 11.99 (s, 1H, Ar-NH), 8.58 (s, 1H, Ar-CH triazole), 8.33 (s, 1H, Ar-CH adjacent to Cl), 7.60 (t, J = 3.0 Hz, 1H, Ar-CH adjacent to NH), 6.85 (s, 1H, Ar-CH). **HPLC:** (5-100% Solvent B, 2 min): R_t = 1.38 min, purity = 99%. **HR-MS** (ESI): calculated (m/z) = 220.0384 [M + H^+], found (m/z) = 220.0385.

2-(4-(5-chloro-1H-pyrrolo[2,3-b]pyridin-4-yl)-1H-1,2,3-triazol-1-yl)ethan-1-ol (**14b**)

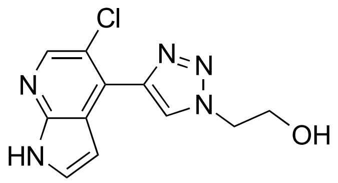

14b was synthesized following general procedure A starting from **13** (12.10 mg, 0.069 mmol, 1.00 equiv)**, 2-**azidoethan-1-ol **b** (8.95 mg, 0.103 mmol, 1.50 equiv), sodium ascorbate (2.73 mg, 1.0 M, 0.014 mmol, 0.20 equiv) and $CuSO_4(H_2O)_5$ (1.71 mg, 0.5 M, 0.007 mmol, 0.05 equiv). The crude product was purified with flash column chromatography (DCM/MeOH = 0-20%) to give the title compound **14b** (15.7 mg, 87%) as a pale yellow solid. **TLC:** (DCM/MeOH = 10:1 v/v) R_f = 0.33. **^{1}H NMR:** 11.96 (s, 1H, NH), 8.82 (s, 1H, Ar-CH triazole), 8.32 (s, 1H, Ar-CH adjacent to Cl), 7.60 (d, *J* = 3.3 Hz, 1H, Ar-CH adjacent to NH), 7.03 (d, *J* = 3.4 Hz, 1H, Ar-CH), 4.56 (t, *J* = 5.4 Hz, 2H, CH_2), 3.88 (t, *J* = 5.4 Hz, 2H, CH_2 adjacent to OH). **^{13}C NMR** (126 MHz, DMSO-*d6*) δ 147.8, 142.7, 141.7, 128.5, 128.3, 127.0, 119.7, 118.8, 102.2, 60.2, 52.9. **HPLC:** (5-100% Solvent B, 2 min): R_t = 1.34 min, purity = 99%. **HR-MS** (ESI): calculated (m/z) = 264.06509 [M + H^+], found (m/z) = 264.06466.

3-(4-(5-chloro-1H-pyrrolo[2,3-b]pyridin-4-yl)-1H-1,2,3-triazol-1-yl)propan-1-ol (**14c**)

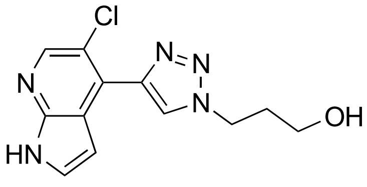

14c was synthesized following general procedure A starting from **13** (10.60 mg, 0.060 mmol, 1.00 equiv), 3-azidopropan-1-ol **14c** (9.10 mg, 0.09 mmol, 1.50 equiv), sodium ascorbate (2.39 mg, 1.0 M, 0.012 mmol, 0.20 equiv) and $CuSO_4(H_2O)_5$ (1.50 mg, 0.5 M, 0.006 mmol, 0.05 equiv).The crude product was purified with flash column chromatography (DCM/MeOH = 0-20%) to give the title compound **14c** (10.9 mg, 65%) as a pale yellow solid. **TLC:** (DCM/MeOH = 10:1 v/v) R_f = 0.36. **^{1}H NMR:** (500 MHz, DMSO-*d6*) δ 11.96 (s, 1H, NH), 8.85 (s, 1H, Ar-CH triazole), 8.32 (s, 1H, Ar-CH adjacent to Cl), 7.60 (dd, J = 3.4, 2.5 Hz, 1H, Ar-CH adjacent to NH), 7.00 (dd, J = 3.4, 2.0 Hz, 1H, Ar-CH), 4.72 (t, J = 5.0 Hz, 1H, OH), 4.57 (t, J = 7.1 Hz, 2H, CH_2), 3.47 (td, J = 6.0, 4.9 Hz, 2H, CH_2 adjacent to OH), 2.11 – 2.02 (m, 2H, CH_2 middle). **^{13}C NMR:** (126 MHz, DMSO-*d6*) δ 147.4, 142.2, 141.3, 128.0, 127.7, 126.1, 119.3, 118.4, 101.7, 57.5, 47.0, 32.9. **HPLC:** (5-100% Solvent B, 2 min): R_t = 1.362 min, purity > 99%. **HR-MS** (ESI): calculated (m/z) = 278.08075 [M + H^+], found (m/z) = 278.08031.

2-(4-(5-chloro-1H-pyrrolo[2,3-b]pyridin-4-yl)-1H-1,2,3-triazol-1-yl)acetic acid (**14d**)

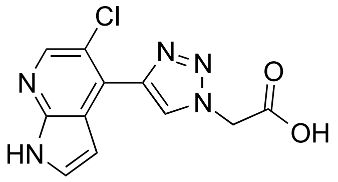

14d was synthesized following general procedure A starting from **13** (50.0 mg, 0.283 mmol, 1.0 equiv), 2-azidoacetic acid **d** (42.92 mg, 0.425 mmol, 1.5 eq.), sodium ascorbate (5.64 mg, 1.0 M, 0.028 mmol, 0.10 equiv) and $CuSO_4(H_2O)_5$ (3.53 mg, 0.5 M, 0.014 mmol, 0.05 eq.) The resulting crude product was purified by preparative HPLC (ACN/H_2O, 5:95 → ACN/H_2O, 4:6) to give the title compound **14d** (66.9 mg, 85%) as a white solid. **^{1}H NMR:** (500 MHz, DMSO-*d6*) δ 12.00 (s, 1H, NH), 8.93 (s, 1H, Ar-CH triazole), 8.34 (s, 1H, Ar-CH adjacent to Cl), 7.62 (t, J = 3.0 Hz, 1H, Ar-CH adjacent to NH), 7.05 (dd, J = 3.5, 2.0 Hz, 1H, Ar-CH), 5.45 (s, 2H, CH_2) ppm. **^{13}C NMR:** (126 MHz, DMSO-*d6*) δ 169.0, 147.9, 142.7, 141.8, 128.6, 128.0, 127.9, 119.7, 118.8, 102.2, 51.2. **HPLC:** (5-100% Solvent B, 2 min): R_t = 1.31 min, purity = 98%. **HR-MS** (ESI): calculated (m/z) = 278.04393 [M + H$^+$], found (m/z) = 278.04401.

(4-(5-chloro-1H-pyrrolo[2,3-b]pyridin-4-yl)-1H-1,2,3-triazol-1-yl)methyl pivalate (**14e**)

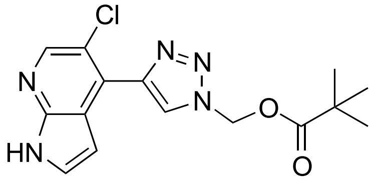

14e was synthesized following general procedure A starting from **13** (20.00 mg, 0.113 mmol, 1.00 equiv), azidomethyl pivalate **e** (17.80 mg, 0.113 mmol, 1.00 equiv), sodium ascorbate (2.25 mg, 1.0 M, 0.011 mmol, 0.10 equiv) and $CuSO_4(H_2O)_5$ (1.41 mg, 0.5 M, 0.006 mmol, 0.05 eq.). The crude product was purified with flash column chromatography (DCM/MeOH = 0-20%) to give the title compound **14e** (35 mg, 93%) as a pale yellow solid. **TLC:** (DCM/MeOH = 20:1, v/v) R_f = 0.18. **^{1}H NMR:** (500 MHz, $CDCl_3$) δ 10.77 (s, 1H, NH), 8.63 (s, 1H, Ar-CH triazole), 8.50 – 8.26 (m, 1H, Ar-CH adjacent to Cl), 7.45 (d, *J* = 3.5 Hz, 1H, Ar-CH adjacent to NH), 7.31 (s, 1H, Ar-CH), 6.38 (s, 2H, CH_2), 1.21 (s, 9H, *t*Butyl CH_3). **^{13}C NMR:** (126 MHz, $CDCl_3$) δ 178.2, 147.9, 143.7, 128.3, 127.5, 126.8, 103.6, 70.2, 39.3, 27.2. **HPLC:** (5-100% Solvent B, 2 min): R_t = 1.92 min, purity = 98%. **HR-MS** (ESI): calculated (m/z) = 334.0 [M + H$^+$], found (m/z) = 334.11.

5-chloro-4-(1-phenyl-1H-1,2,3-triazol-4-yl)-1H-pyrrolo[2,3-b]pyridine (**14f**)

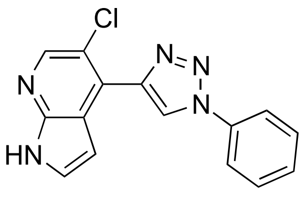

14f was synthesized following general procedure A starting from **13** (10.00 mg, 0.057 mmol, 1.00 equiv) azidobenzene **f** (10.12 mg, 0.085 mmol, 1.50 equiv), sodium ascorbate (2.25 mg, 1.0 M, 0.011 mmol, 0.20 equiv) and $CuSO_4(H_2O)_5$ (1.41 mg, 0.5 M, 0.006 mmol, 0.10 equiv). The crude product was purified with flash column chromatography (DCM/MeOH = 0-20%) to give the title compound **14f** (12.8 mg, 76%) as a pale yellow solid. **TLC:** (DCM/MeOH = 20:1 v/v) R_f = 0.17. **^{1}H NMR:** (500 MHz, DMSO-*d6*) δ 12.04 (s, 1H, NH), 9.41 (s, 1H, Ar-CH triazole), 8.37 (s, 1H, Ar-CH adjacent to Cl), 8.09 – 8.03 (m, 2H, ortho phenyl CH), 7.69 – 7.62 (m, 3H, meta phenyl CH + Ar-CH adjacent to NH), 7.59 – 7.52 (m, 1H, para phenyl CH), 6.98 (dd, J = 3.5, 2.0 Hz, 1H, Ar-CH).

^{13}C **NMR:** (126 MHz, DMSO-*d6*) δ 147.4, 142.4, 142.2, 136.4, 130.0, 129.1, 128.4, 127.2, 123.9, 120.6, 119.8, 118.7, 101.4. **HPLC:** (5-100% Solvent B, 2 min): R_t = 1.92 min, purity = 83%. **HR-MS** (ESI): calculated (m/z) = 296.06994 [M + H^+], found (m/z) = 296.06975.

5-chloro-4-(1-(p-tolyl)-1H-1,2,3-triazol-4-yl)-1H-pyrrolo[2,3-b]pyridine (**14g**)

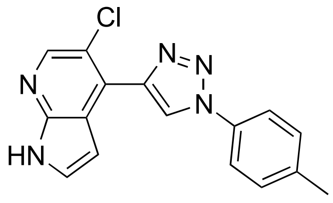

14g was synthesized following general procedure A starting from **13** (10.00 mg, 0.057 mmol, 1.00 equiv), 1-azido-4-methylbenzene **g** (10.12 mg, 0.085 mmol, 1.50 equiv), sodium ascorbate (2.25 mg, 1.0 M, 0.011 mmol, 0.20 equiv) and $CuSO_4(H_2O)_5$ (1.41 mg, 0.5 M, 0.006 mmol, 0.10 equiv). The crude product was purified with flash column chromatography (DCM/MeOH = 0-20%) to give the title compound **14g** (9.5 mg, 54%) as a pale yellow solid. **TLC:** (DCM/MeOH = 20:1 v/v) R_f = 0.29. ^{1}**H NMR:** (500 MHz, DMSO-*d6*) δ 12.03 (s, 1H, NH), 9.36 (s, 1H, Ar-CH triazole), 8.37 (s, 1H, Ar-CH adjacent to Cl), 7.97 – 7.90 (m, 2H, ortho phenyl CH), 7.64 (t, J = 3.0 Hz, 1H, Ar-CH adjacent to NH), 7.45 (d, J = 8.2 Hz, 2H, meta phenyl CH), 6.98 (dd, J = 3.4, 1.8 Hz, 1H, Ar-CH), 2.41 (s, 3H, CH_3). ^{13}**C NMR:** (126 MHz, DMSO-*d6*) δ 147.4, 142.3, 142.1, 138.7, 134.2, 130.3, 128.4, 127.2, 123.7, 120.5, 119.7, 118.7, 101.4, 20.6. **HPLC:** (5-100% Solvent B, 2 min): R_t = 2.03 min, purity = 100%. **HR-MS** (ESI): calculated (m/z) = 310.08570 [M + H^+], found (m/z) = 310.08540.

5-chloro-4-(1-(4-fluorophenyl)-1H-1,2,3-triazol-4-yl)-1H-pyrrolo[2,3-b]pyridine (**14h**)

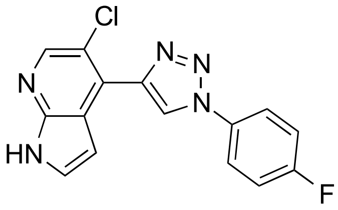

14h was synthesized following general procedure A starting from **13** (10.00 mg, 0.057 mmol, 1.00 equiv), 1-azido-4-fluorobenzene **h** (11.65 mg, 0.085 mmol, 1.50 equiv), sodium ascorbate (2.25 mg, 1.0 M, 0.011 mmol, 0.20 equiv) and $CuSO_4(H_2O)_5$ (1.41 mg, 0.5 M, 0.006 mmol, 0.10 equiv).The crude product was purified with flash column chromatography (DCM/MeOH = 0-20%) to give the title compound **14h** (12.6 mg, 71%) as a pale yellow solid. **TLC:** (DCM/MeOH = 10:1 v/v) R_f = 0.66. ^{1}**H NMR:** (500 MHz, DMSO-*d6*) δ 12.04 (s, 1H, NH), 9.40 (s, 1H, Ar-CH triazole), 8.38 (s, 1H, Ar-CH adjacent to Cl), 8.15 – 8.07 (m, 2H, ortho phenyl CH), 7.65 (t, *J* = 3.0 Hz, 1H, Ar-CH adjacent to NH), 7.55 – 7.47 (m, 2H, meta phenyl CH), 6.99 (dd, *J* = 3.5, 1.8 Hz, 1H, Ar-CH). ^{13}**C NMR:** (126 MHz, DMSO-*d6*) δ 162.4 (d, J = 246.1 Hz), 147.9, 142.9, 142.6, 133.5 (d, J = 3.2 Hz), 128.9, 127.6, 124.7, 123.5 (d, J = 9.0 Hz), 120.2, 119.2, 117.2 (d, J = 23.3 Hz), 101.8. **HPLC:** (5-100% Solvent B, 2 min): R_t = 1.95 min, purity = 90%. **HR-MS** (ESI): calculated (m/z) = 314.06032 [M + H^+], found (m/z) = 314.06033.

5-chloro-4-(1-(3-fluorophenyl)-1H-1,2,3-triazol-4-yl)-1H-pyrrolo[2,3-b]pyridine (**14i**)

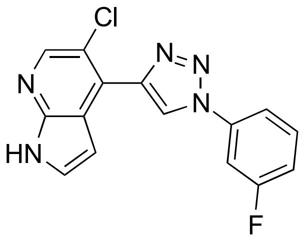

14i was synthesized following general procedure A starting from **13** (10.00 mg, 0.057 mmol, 1.00 equiv) 1-azido-3-fluorobenzene **i** (11.65 mg, 0.085 mmol, 1.50 equiv), sodium ascorbate (2.25 mg, 1.0 M, 0.011 mmol, 0.20 equiv) and $CuSO_4(H_2O)_5$ (1.41 mg, 0.5 M, 0.006 mmol, 0.10 equiv). The crude product was purified with flash column chromatography (DCM/MeOH = 0-20%) to give the title compound **14i** (10.2 mg, 57%) as a pale yellow solid. **TLC:** (DCM/MeOH = 10:1 v/v) R_f = 0.41. **^{1}H NMR:** (500 MHz, DMSO) δ 12.06 (s, 1H, NH), 9.48 (s, 1H, Ar-CH triazole), 8.39 (s, 1H, Ar-CH adjacent to Cl), 8.04 (dt, *J* = 10.0, 2.3 Hz, 1H, ortho phenyl CH), 7.97 (ddd, *J* = 8.1, 2.1, 0.9 Hz, 1H, ortho phenyl CH), 7.75 – 7.62 (m, 2H, Ar-CH adjacent to NH + meta phenyl CH), 7.41 (tdd, *J* = 8.5, 2.5, 0.8 Hz, 1H, para phenyl CH), 6.98 (dd, *J* = 3.5, 1.9 Hz, 1H, Ar-CH).**^{13}C NMR:** (126 MHz, DMSO) δ 162.9 (d, J = 245.1 Hz), 147.9, 143.0, 142.6, 138.1 (d, J = 10.6 Hz), 132.3 (d, J = 9.1 Hz), 129.0, 127.4, 124.5, 120.3, 119.2, 117.0 (d, J = 3.0 Hz), 116.2 (d, J = 21.1 Hz), 108.6 (d, J = 26.7 Hz), 101.8. **HPLC:** (5-100% Solvent B, 2 min): R_t = 1.97 min, purity = 96%. **HR-MS** (ESI): calculated (m/z) = 314.06056 [M + H^+], found (m/z) = 314.06033.

5-chloro-4-(1-(pyridin-3-yl)-1H-1,2,3-triazol-4-yl)-1H-pyrrolo[2,3-b]pyridine (**14j**)

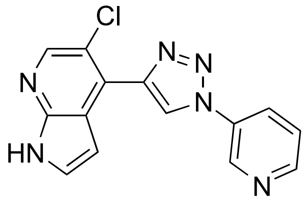

14j was synthesized following general procedure A starting from **13** (10.00 mg, 0.057 mmol, 1.00 equiv), 3-azidopyridine **j** (10.20 mg, 0.085 mmol, 1.50 equiv), sodium ascorbate (2.25 mg, 1.0 M, 0.011 mmol, 0.20 equiv) and $CuSO_4(H_2O)_5$ (1.41 mg, 0.5 M, 0.006 mmol, 0.10 equiv). The crude product was purified with flash column chromatography (DCM/MeOH = 0-20%) to give the title compound **14j** (11.7 mg, 70%) as a pale yellow solid. **TLC:** (DCM/MeOH = 10:1 v/v) R_f = 0.47. **^{1}H NMR:** (500 MHz, DMSO-*d6*) δ 12.06 (s, 1H, NH), 9.53 (s, 1H, Ar-CH triazole), 9.30 (d, *J* = 2.6 Hz, 1H, CH ortho to N of pyridine), 8.75 (dd, *J* = 4.7, 1.4 Hz, 1H, CH para to N of pyridine), 8.50 (ddd, *J* = 8.3, 2.7, 1.4 Hz, 1H, CH meta to N of pyridine), 8.38 (s, 1H, Ar-CH adjacent to Cl), 7.71 (ddd, *J* = 8.3, 4.8, 0.8 Hz, 1H, CH ortho to N of pyridine), 7.65 (dd, *J* = 3.5, 2.5 Hz, 1H, Ar-CH adjacent to NH), 6.99 (dd, *J* = 3.4, 1.9 Hz, 1H, Ar-CH). **^{13}C NMR:** (126 MHz, DMSO-*d6*) δ 150.0, 147.4, 142.6, 142.2, 141.8, 133.2, 128.6, 128.5, 126.9, 124.6, 124.4, 119.8, 118.7, 101.3. **HPLC:** (5-100% Solvent B, 2 min): R_t = 1.60 min, purity: >99%. **HR-MS** (ESI): calculated (m/z) = 297.06508 [M + H^+], found (m/z) = 297.06500.

4-(1-benzyl-1H-1,2,3-triazol-4-yl)-5-chloro-1H-pyrrolo[2,3-b]pyridine (**14k**)

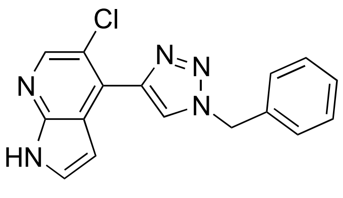

14k was synthesized following general procedure A starting from **13** (20.00 mg, 0.113 mmol, 1.00 equiv), (azidomethyl)benzene **k** (15.08 mg, 0.113 mmol, 1.00 equiv), sodium ascorbate (2.25 mg, 1.0 M, 0.011 mmol, 0.10 equiv) and $CuSO_4(H_2O)_5$ (1.41 mg, 0.5 M, 0.006 mmol, 0.05 eq.) The crude product was purified with

preparative HPLC (ACN/H_2O, 5:95 → ACN/H_2O, 4:6) to give the title compound **14k** 23.6 mg, 67%) as a pale yellow solid. **TLC:** (DCM/MeOH = 20:1, v/v) R_f = 0.29.**^{1}H NMR:** (500 MHz, DMSO-*d6*) δ 11.99 (s, 1H, NH), 8.99 (s, 1H, Ar-CH triazole), 8.43 (s, 1H, Ar-CH adjacent to Cl), 7.59 (s, 1H, Ar-CH adjacent to NH), 7.40 (d, *J* = 5.3 Hz, 5H, phenyl CH), 7.02 (s, 1H, Ar-CH), 5.76 (s, 2H, CH_2). **^{13}C NMR:** (126 MHz, DMSO-*d6*) δ 147.3, 142.0, 141.6, 135.9, 128.8, 128.2, 128.04, 127.95, 127.4, 126.2, 101.7, 52.9. **HPLC:** (5-100% Solvent B, 2 min): R_t = 1.85 min, purity = 100%. **HR-MS** (ESI): calculated (m/z) = 310.08540 [M + H^+], found (m/z) = 310.08601.

4-((4-(5-chloro-1H-pyrrolo[2,3-b]pyridin-4-yl)-1H-1,2,3-triazol-1-yl)methyl)benzonitrile (**14l**)

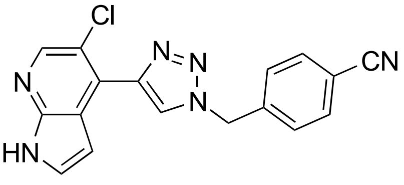

14l was synthesized following following general procedure A starting from **13** (20 mg, 0.113 mmol, 1.00 equiv), 4-(azidomethyl)benzonitrile **l** (17.91 mg, 0.113 mmol, 1.00 equiv), sodium ascorbate (2.25 mg, 1.0 M, 0.011 mmol, 0.10 equiv) and $CuSO_4(H_2O)_5$ (1.41 mg, 0.5 M, 0.006 mmol, 0.05 equiv). The crude product was purified with preparative HPLC (ACN/H_2O, 5:95 → ACN/H_2O, 4:6) to give the title compound **14l** (5.5 mg, 15%) as a white solid. **TLC:** (DCM/MeOH = 10:1, v/v), R_f = 0.54. **^{1}H NMR:** (500 MHz, DMSO-*d6*) δ 11.98 (s, 1H, NH), 9.06 (s, 1H, Ar-CH triazole), 8.32 (s, 1H, Ar-CH adjacent to Cl), 7.92 – 7.86 (m, 2H, ortho phenyl CH), 7.60 (t, *J* = 3.0 Hz, 1H, Ar-CH adjacent to NH), 7.56 (d, *J* = 8.0 Hz, 2H, meta phenyl CH), 7.02 – 6.98 (m, 1H, Ar-CH), 5.88 (s, 2H, CH_2). **^{13}C NMR:** (126 MHz, DMSO-*d6*) δ 147.9, 142.7, 142.2, 141.9, 133.3, 129.3, 128.6, 127.8, 127.1, 119.8, 119.0, 118.8, 111.5, 102.2, 52.8. **HPLC:** (5-100% Solvent B, 2 min): R_t = 1.76 min, purity = 100%. **HR-MS** (ESI): calculated (m/z) = 335.08065 [M + H^+], found (m/z) = 335.08102.

2-(4-(5-chloro-1H-pyrrolo[2,3-b]pyridin-4-yl)-1H-1,2,3-triazol-1-yl)acetamide (**24**)

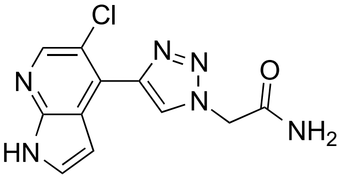

14d (23.0 mg, 0.082 mmol, 1.00 equiv) was dissolved in DMF (2 mL). Then di(1H-imidazol-1-yl)methanone (16.12 mg, 0.099 mmol, 1.2 equiv) was added to reaction mixture. After stirring at rt for 1 h, ammonia in methanol (1.69 mg, 7.0 M, 0.099 mmol, 1.2 equiv) was added to reaction mixture. After 4 h, H_2O (5 mL) and DCM (10 mL) were added to reaction mixture. The aqueous phase was extracted with DCM (3 × 10 mL). The organic phases were combined and the solvents were removed under reduced pressure. The resulting crude product was purified by flash column chromatography (DCM/MeOH = 0-18% for 10 CV, 18%-100% for 2 CV) to give the title compound **24** (11.2 mg, 30%) as a white solid. **TLC:** (DCM/MeOH = 10:1, v/v) R_f = 0.26. **^{1}H NMR:** (500 MHz, DMSO-*d6*) δ 11.97 (s, 1H, NH), 8.84 (s, 1H, Ar-CH triazole), 8.33 (s, 1H, Ar-CH adjacent to Cl), 7.61 (q, J = 2.9 Hz, 1H, Ar-CH adjacent to NH), 7.05 (dd, J = 3.7, 1.7 Hz, 1H, Ar-CH), 5.24 (s, 2H, CH_2). **^{13}C NMR:** (126 MHz, DMSO) δ 167.2, 147.5, 142.3, 141.2, 128.0, 127.7, 127.6, 119.2, 118.3, 101.8, 51.6. **HPLC:** (5-100% Solvent B, 2 min): R_t = 1.245 min, purity = 80%. **HR-MS** (ESI): calculated (m/z) = 277.05991 [M + H^+], found (m/z) = 277.06014.

Methyl 2-(4-(5-chloro-1H-pyrrolo[2,3-b]pyridin-4-yl)-1H-1,2,3-triazol-1-yl)acetate (**23**)

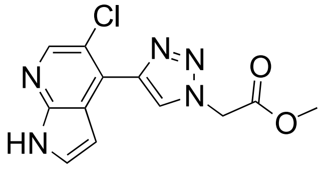

23 (7.7 mg, 49%) was obtained as a side product during the synthesis of **24**. **^{1}H NMR**: (500 MHz, DMSO-*d6*) δ 12.01 (s, 1H, NH), 8.94 (s, 1H, Ar-CH triazole), 8.34 (s, 1H, Ar-CH adjacent to Cl), 7.62 (t, *J* = 2.9 Hz, 1H, Ar-CH adjacent to NH), 7.03 (dd, *J* = 3.3, 1.8 Hz, 1H, Ar-CH), 5.57 (s, 2H, CH_2), 3.76 (s, 3H, CH_3). **^{13}C NMR** (126 MHz, DMSO-*d6*) δ 167.7, 147.4, 142.2, 141.5, 128.2, 127.6, 127.3, 119.3, 118.3, 101.7, 52.6, 50.5. **HPLC:** (5-100% Solvent B, 2 min): R_t = 1.536 min, purity = 99%.

N-benzyl-2-(4-(5-chloro-1H-pyrrolo[2,3-b]pyridin-4-yl)-1H-1,2,3-triazol-1-yl)acetamide (**25**)

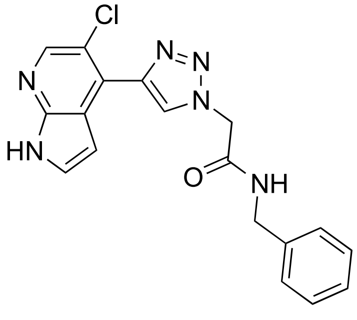

14d (37.0 mg, 0.133 mmol, 1.0 equiv) was dissolved in dry DMF (2 mL) and phenylmethanamine **37** (35.7 mg, 0.333 mmol, 2.50 equiv), DIPEA (51.67 mg, 0.399 mmol, 3.00 equiv) and HATU (88.67 mg, 0.233 mmol, 1.75 equiv) were added to reaction mixture under argon. Reaction mixture was stirred at rt for 1.5 days then diluted with water (4 mL) and DCM (10 mL). The aqueous phase was extracted with DCM (10 mL × 3). The organic phase was combined and washed with brine (5 mL), dried over $MgSO_4$, filtered, and removed solvents under reduced pressure. The resulting crude product was purified by flash chromatography (DCM/MeOH = 0-18% for 10 CV, 18%-100% for 2 CV) to give the title compound **25** (34.7 mg, 71%) as a white solid. **TLC:** (DCM/MeOH = 10:1) R_f = 0.45. **^{1}H NMR:** (500 MHz, DMSO-*d6*) δ 11.99 (s, 1H, NH), 8.92 (t, *J* = 6.0 Hz, 1H, NH amide), 8.89 (s, 1H, Ar-CH triazole), 8.34 (s, 1H, Ar-CH adjacent to Cl), 7.62 (t, *J* = 3.3 Hz, 1H, Ar-CH adjacent to NH), 7.38 – 7.29 (m, 4H, phenyl CH), 7.29 – 7.23 (m, 1H, para phenyl CH), 7.06 (dt, *J* = 3.2, 1.5 Hz, 1H, Ar-CH), 5.36 (s, 2H, CH_2), 4.37 (d, *J* = 5.8 Hz, 2H, CH_2). **^{13}C NMR:** (126 MHz, DMSO-*d6*) δ 165.4, 147.5, 142.3, 141.3, 138.7, 128.4, 128.1, 127.7, 127.5, 127.4, 127.0, 119.3, 118.3, 101.8, 51.8, 42.5. **HPLC:** (5-100% Solvent B, 2 min): R_t = 1.68 min, purity = 99%. **HR-MS** (ESI): calculated (m/z) = 367.10711 [M + H$^+$], found (m/z) = 367.10686.

2-(4-(5-chloro-1H-pyrrolo[2,3-b]pyridin-4-yl)-1H-1,2,3-triazol-1-yl)-N-(4-methoxybenzyl)acetamide (**26**)

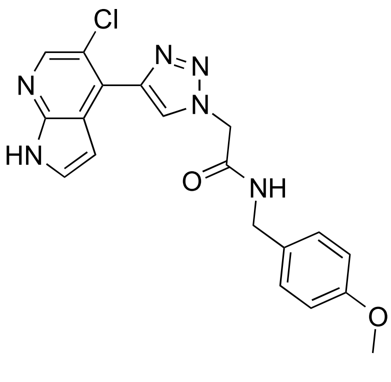

14d (10 mg, 0.036 mmol, 1.00 equiv) was dissolved in dry DMF (0.8 mL). (4-methoxyphenyl)methanamine **38** (12.35 mg, 0.090 mmol, 2.50 equiv), DIPEA (13.96 mg, 0.108 mmol, 3.00 equiv) and HATU (34.92 mg, 0.091 mmol, 2.55 equiv) were added in sequence under argon. The reaction mixture was stirred at rt for overnight. 3 mL water and 10 mL DCM were added. The product was extracted with DCM (3× 38 mL). The organic phase was combined and removed solvents under reduced pressure. The resulting crude product was purified by flash chromatography (DCM/MeOH = 0-18% for 10 CV, 18%-100% for 2 CV) to give the title compound **26** (9.7 mg, 68%) as a white solid. **TLC:** (DCM/MeOH = 10:1) R_f = 0.34. **^{1}H NMR:** (500 MHz, DMSO-*d6*) δ 12.01 (s, 1H, NH), 8.89 (t, *J* = 5.8 Hz, 1H, NH amide), 8.87 (s, 1H, Ar-CH triazole), 8.33 (s, 1H, Ar-CH adjacent to Cl), 7.61 (t, *J* = 3.0 Hz, 1H, Ar-CH adjacent to NH), 7.25 – 7.22 (m, 2H, phenyl CH), 7.04 (dd, *J* = 3.5, 2.0 Hz, 1H, Ar-CH), 6.95 – 6.87 (m, 2H, phenyl CH), 5.31 (s, 2H, CH_2), 4.28 (d, *J* = 5.7 Hz, 2H, CH_2 adjacent to amide), 3.73 (s, 3H, OCH_3). **^{13}C NMR:** (126 MHz, DMSO-*d6*) δ 165.2, 158.4, 147.5, 142.2, 141.2, 130.7, 128.8, 128.1, 127.7, 127.5, 119.2, 118.3, 113.8, 101.8, 55.1, 51.8, 41.9. **HPLC:** (5-100% Solvent B, 2 min): R_t = 1.69 min, purity = 93%. **HR-MS** (ESI): calculated (m/z) = 397.11794 [M + H$^+$], found (m/z) = 397.11743.

(S)-2-(4-(5-chloro-1H-pyrrolo[2,3-b]pyridin-4-yl)-1H-1,2,3-triazol-1-yl)propan-1-ol (**14b1**).

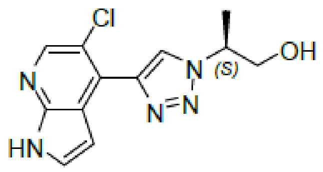

14b1 was synthesized following general procedure A starting from **13** (10.00 mg, 0.057 mmol, 1.00 equiv), (S)-2-azidopropan-1-ol **b1** (11.45 mg, 0.011 mmol, 2.00 equiv), sodium ascorbate (2.24 mg, 0.011 mmol, 0.20 equiv) and $CuSO_4(H_2O)_5$ (2.83 mg, 0.011 mmol, 0.20 equiv). Purification was carried out by preparative HPLC (ACN/H_2O, 5:95 → ACN/H_2O, 4:6) to provide the title compound **14b1** (10.5 mg, 67%) as a light yellow solid. **TLC:** (DCM/MeOH = 20:1, v/v) R_f = 0.14. **^{1}H NMR:** (500 MHz, DMSO-*d6*): δ 11.95 (s, 1H, NH), 8.81 (s, 1H, Ar-CH triazole), 8.32 (s, 1H, Ar-CH adjacent to Cl), 7.59 (t, *J* = 3.0 Hz, 1H, Ar-CH adjacent to NH), 7.03 (dd, *J* = 3.5, 1.9 Hz, 1H, Ar-CH), 4.85 (td, *J* = 6.9, 5.2 Hz, 1H, CH), 3.80 (dd, *J* = 5.9, 2.6 Hz, 2H, CH_2), 1.55 (d, *J* = 7.0 Hz, 3H, CH_3). **^{13}C NMR:** (126 MHz, DMSO-*d6*): δ 147.4, 142.2, 141.1, 128.0, 127.9, 125.0, 119.2, 118.4, 101.9, 64.4, 58.8, 17.0. **HPLC:** (5-100% Solvent B, 2 min): R_t = 1.436 min, purity (220 nm): >100%. **HR-MS** (m/z): (ESI) calculated [M+H]+: 278.08031, found 278.08027.

(R)-2-(4-(5-chloro-1H-pyrrolo[2,3-b]pyridin-4-yl)-1H-1,2,3-triazol-1-yl)propan-1-ol (**14b2**)

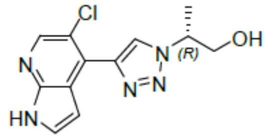

14b2 was synthesized following general procedure A starting from **13** (10.00 mg, 0.057 mmol, 1.00 equiv), (R)-2-azidopropan-1-ol **b2** (11.45 mg, 0.011 mmol, 2.00 equiv), sodium ascorbate (2.24 mg, 0.011 mmol, 0.20 equiv) and $CuSO_4(H_2O)_5$ (2.83 mg, 0.011 mmol, 0.20 equiv). Purification was carried out by preparative HPLC (ACN/H_2O, 5:95 → ACN/H_2O, 4:6) to provide the title compound **14b2** (14.3 mg, 91%) as a light yellow solid. **TLC:** (DCM/MeOH = 20:1, v/v) R_f = 0.14. **^{1}H NMR:** (500 MHz, DMSO-*d6*): δ 11.95 (s, 1H, NH), 8.81 (s, 1H, Ar-CH triazole), 8.32 (s, 1H, Ar-CH adjacent to Cl), 7.59 (t, *J* = 3.0 Hz, 1H, Ar-CH adjacent to NH), 7.03 (dt, *J* = 3.3, 1.3 Hz, 1H, Ar-CH), 4.85 (td, *J* = 7.0, 5.2 Hz, 1H, CH), 3.84 – 3.76 (m, 2H, CH_2), 1.55 (d, *J* = 6.9 Hz, 3H, CH_3). **^{13}C NMR:** (126 MHz, DMSO-*d6*): δ 147.6, 142.4, 141.2, 128.1, 128.0, 125.2, 119.4, 118.5, 102.0, 64.6, 59.0, 17.2. **HPLC:** (5-100% Solvent B, 2 min): R_t = 1.43 min, purity (220 nm): > 99 %. **HR-MS** (m/z): (ESI) calculated [M+H]+: 278.08031, found 278.08021.

(S)-1-(4-(5-chloro-1H-pyrrolo[2,3-b]pyridin-4-yl)-1H-1,2,3-triazol-1-yl)propan-2-ol (**14b3**)

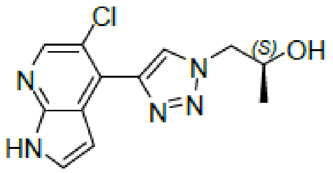

14b3 was synthesized following general procedure A starting from **13** (10.00 mg, 0.057 mmol, 1.00 equiv), (S)-1-azidopropan-2-ol **b3** (11.45 mg, 0.011 mmol, 2.00 equiv), sodium ascorbate (2.24 mg, 0.011 mmol, 0.20 equiv) and $CuSO_4(H_2O)_5$ (2.83 mg, 0.011 mmol, 0.20 equiv). Purification was carried out by preparative HPLC (ACN/H_2O, 5:95 → ACN/H_2O, 4:6) to yield the title compound **14b3** (10.8 mg, 69%) as a white solid. **TLC:** (DCM/MeOH = 9:1, v/v) R_f = 0.37. **^{1}H NMR:** (500 MHz, DMSO-*d6*): δ 11.95 (s, 1H, NH), 8.77 (s, 1H, Ar-CH triazole), 8.31 (s, 1H, Ar-CH, adjacent to Cl), 7.59 (t, J = 3.0 Hz, 1H, Ar-CH, adjacent to NH), 7.02 (dd, J = 3.4, 1.9 Hz, 1H, Ar-CH), 5.13 (d, J = 5.1 Hz, 1H, OH), 4.49 (dd, J = 13.7, 4.1 Hz, Ha of CH_2), 4.37 (dd, J = 13.6, 7.4 Hz, 1H, Hb of CH_2), 4.11 (ddd, J = 9.5, 6.9, 4.8 Hz, 1H, CH), 1.13 (d, J = 6.3 Hz, 3H, CH_3). **^{13}C NMR:** (126 MHz, DMSO) δ 147.4, 142.2, 141.1, 128.0, 127.7, 126.7, 119.2, 118.3, 101.8, 65.2, 56.6, 20.8. **HPLC:** (5-100% Solvent B, 2 min): R_t = 1.41 min, purity (220 nm): >99 %. **HR-MS** (m/z): (ESI) calculated [M+H]+: 278.08031, found 278.08024.

(R)-1-(4-(5-Chloro-1H-pyrrolo[2,3-b]pyridin-4-yl)-1H-1,2,3-triazol-1-yl)propan-2-ol (**14b4**)

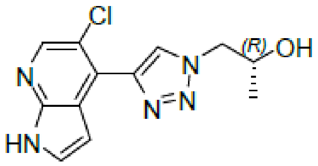

14b4 was synthesized following general procedure A starting from **13** (10.00 mg, 0.057 mmol, 1.00 equiv), (R)-1-azidopropan-2-ol **b4** (11.45 mg, 0.011 mmol, 2.00 equiv), sodium ascorbate (2.24 mg, 0.011 mmol, 0.20 equiv) and $CuSO_4(H_2O)_5$ (2.83 mg, 0.011 mmol, 0.20 equiv). Purification was carried out by preparative HPLC (ACN/H_2O, 5:95 → ACN/H_2O, 4:6) to obtain the title compound **14b4** (6.2 mg, 40%) as a white solid. **TLC:** (DCM/MeOH = 95:5, v/v) R_f = 0.11. **^{1}H NMR:** (500 MHz, DMSO-*d6*): δ 11.96 (s, 1H, NH), 8.78 (s, 1H, Ar-CH triazole), 8.32 (s, 1H, Ar-CH adjacent to Cl), 7.60 (d, *J* = 3.5 Hz, 1H, Ar-CH adjacent to NH), 7.03 (d, *J* = 3.3 Hz, 1H, Ar-CH), 5.13 (d, *J* = 5.1 Hz, 1H, OH), 4.49 (dd, *J* = 13.6, 4.1 Hz, 1H, Ha of CH_2), 4.38 (dd, *J* = 13.7, 7.5 Hz, 1H Hb of CH_2), 4.16 – 4.07 (m, 1H, CH), 1.13 (d, *J* = 6.3 Hz, 3H, CH_3). **^{13}C NMR:** (126 MHz, DMSO-*d6*): δ 147.4, 142.2, 141.1, 128.0, 127.7, 126.7, 119.2, 118.3, 101.8, 65.2, 56.6, 20.8. **HPLC:** (5-100% Solvent B, 2 min): R_t = 1.415 min, purity (220 nm): > 99%. **HR-MS** (m/z): (ESI) calculated [M+H]+: 278.08031, found 278.08025.

(S)-4-(4-(5-Chloro-1H-pyrrolo[2,3-b]pyridin-4-yl)-1H-1,2,3-triazol-1-yl)butan-2-ol (**14c1**)

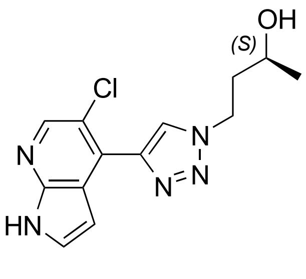

14c1 was synthesized following general procedure A starting from **13** (15.00 mg, 0.085 mmol, 1.00 equiv), (S)-4-azidobutan-2-ol **c1** (19.56 mg, 0.017 mmol, 2.00 equiv), sodium ascorbate (3.37 mg, 0.017 mmol, 0.20 equiv) and $CuSO_4(H_2O)_5$ (4.24 mg, 0.017 mmol, 0.20 equiv). Purification was carried out by preparative HPLC (ACN/H_2O, 5:95 → ACN/H_2O, 4:6) to provide the title compound **14c1** (16.7 mg, 67%) as a white solid. **TLC:** (DCM:MeOH = 95:5, v/v) R_f = 0.25. **^{1}H NMR:** (500 MHz, DMSO-*d*6): = 11.96 (s, 1H, NH), 8.85 (s, 1H, Ar-CH triazole), 8.32 δ (s, 1H, Ar-CH adjacent to Cl), 7.59 (t, *J* = 3.0 Hz, 1H, Ar-CH adjacent to NH), 7.01 – 6.98 (m, 1H, Ar-CH), 4.57 (td, *J* = 7.5, 6.8, 2.7 Hz, 2H, CH_2), 3.64 (ddd, *J* = 8.3, 6.2, 4.1 Hz, 1H, CH), 2.03 (dtd, *J* = 15.6, 7.8, 4.1 Hz, 1H, Ha of CH_2), 1.92 (dtd, *J* = 13.8, 7.9, 6.0 Hz, 1H, Hb of CH_2), 1.12 (d, *J* = 6.2 Hz, 3H, CH_3). **^{13}C NMR:** (126 MHz, DMSO-*d*6): δ = 147.9, 142.6, 141.7, 128.5, 128.2, 126.5, 119.8, 118.9, 102.2, 63.7, 47.5, 39.6, 24.1. **HPLC:** (5-100% Solvent B, 2 min): R_t = 1.470 min, purity (220 nm) >99%. **HR-MS** (m/z): (ESI) calculated [M+H]+: 292.09596, found 292.09593

(R)-4-(4-(5-Chloro-1H-pyrrolo[2,3-b]pyridin-4-yl)-1H-1,2,3-triazol-1-yl)butan-2-ol (**14c2**)

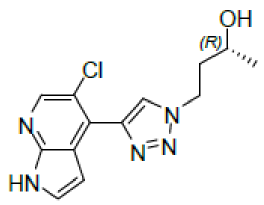

14c2 was synthesized following general procedure A starting from **13** (15.00 mg, 0.085 mmol, 1.00 equiv), (R)-4-azidobutan-2-ol **c2** (19.56 mg, 0.017 mmol, 2.00 equiv), sodium ascorbate (3.37 mg, 0.017 mmol, 0.20 equiv) and $CuSO_4(H_2O)_5$ (4.24 mg, 0.017 mmol, 0.20 equiv). Purification was carried out by preparative HPLC (ACN/H_2O, 5:95 → ACN/H_2O, 4:6) to obtain the title compound **14c2** (18.2 mg, 73%) as a white solid. **TLC:** (EA/CH = 2:1, v/v) R_f = 0.18. **^{1}H NMR:** (500 MHz, DMSO-*d*6): = 11.96 (s, 1H, NH), 8.85 (s, 1H, Ar-CH triazole), 8.32 δ (s, 1H, Ar-CH adjacent to Cl), 7.62 – 7.57 (m, 1H, Ar-CH adjacent to NH), 7.00 (dd, *J* = 3.6, 1.9 Hz, 1H, Ar-CH), 4.57 (td, *J* = 7.5, 6.7, 2.7 Hz, 2H, CH_2), 3.68 – 3.61 (m, 1H, CH), 2.03 (dtd, *J* = 15.7, 7.8, 4.1 Hz, 1H, Ha of CH_2), 1.92 (dtd, *J* = 13.8, 7.9, 6.1 Hz, 1H, Hb of CH_2), 1.12 (d, *J* = 6.1 Hz, 3H, CH_3). **^{13}C NMR**: (126 MHz, DMSO-*d*6) δ 147.4, 142.2, 141.3, 128.0, 127.8, 126.1, 119.3, 118.4, 101.7, 63.2, 47.0, 23.6. **HPLC:** (5-100% Solvent B, 2 min): R_t = 1.472 min, purity (220 nm): >99 %. **HR-MS** (m/z): (ESI) calculated [M+H]+: 292.09596, found 292.09593.

(S)-3-(4-(5-Chloro-1H-pyrrolo[2,3-b]pyridin-4-yl)-1H-1,2,3-triazol-1-yl)-2-methylpropan-1-ol (**14c3**)

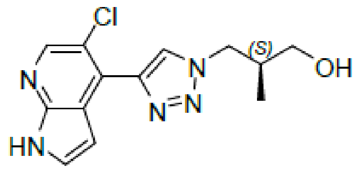

14c3 was synthesized following general procedure A starting from **13** (15.00 mg, 0.085 mmol, 1.00 equiv), (S)-3-azido-2-methylpropan-1-ol **c3** (19.56 mg, 0.017 mmol, 2.00 equiv), sodium ascorbate (3.37 mg, 0.017 mmol, 0.20 equiv) and $CuSO_4(H_2O)_5$ (4.24 mg, 0.017 mmol, 0.20 equiv). Purification was carried out by preparative HPLC (ACN/H_2O, 5:95 → ACN/H_2O, 4:6) to yield the title compound **14c3** (16.9 mg, 68%) as a white solid. **TLC:** (DCM/MeOH = 95:5, v/v) R_f = 0.21. **^{1}H NMR:** (500 MHz, DMSO-*d*6) δ 11.96 (s, 1H, NH), 8.82 (s, 1H, Ar-CH triazole), 8.32 (s, 1H, Ar-CH adjacent to Cl), 7.60 (t, *J* = 3.0 Hz, 1H, Ar-CH adjacent to NH), 6.99 (dd, *J* = 3.4, 1.9 Hz, 1H, Ar-CH), 4.56 (dd, *J* = 13.6, 6.1 Hz, 1H), 4.36 (dd, *J* = 13.6, 7.6 Hz, 1H, Ha of CH_2), 3.35 (qd, *J* = 10.7, 5.8 Hz, 2H, CH_2 adjacent to OH), 2.22 (dq, *J* = 13.1, 6.5 Hz, 1H, Hb of CH_2), 0.86 (d, *J* = 6.8 Hz, 3H, CH_3). **^{13}C**

NMR: (126 MHz, DMSO-*d6*): δ = 147.4, 142.2, 141.2, 128.1, 127.7, 126.6, 119.3, 118.4, 101.7, 63.1, 52.5, 36.7, 14.3. **HPLC:** (5-100% Solvent B, 2 min): R_t = 1.478 min, purity (220 nm) = > 99%. **HR-MS** (m/z): (ESI) calculated [M+H]+: 292.09596, found 292.09588

(R)-3-(4-(5-Chloro-1H-pyrrolo[2,3-b]pyridin-4-yl)-1H-1,2,3-triazol-1-yl)-2-methylpropan-1-ol (**14c4**).

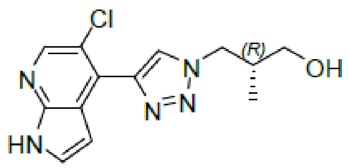

14c4 was synthesized following general procedure A starting from **13** (15.00 mg, 0.085 mmol, 1.00 equiv), (R)-3-azido-2-methylpropan-1-ol **c4** (19.56 mg, 0.017 mmol, 2.00 equiv), sodium ascorbate (3.37 mg, 0.017 mmol, 0.20 equiv) and $CuSO_4(H_2O)_5$ (4.24 mg, 0.017 mmol, 0.20 equiv). Purification was carried out by preparative HPLC (ACN/H_2O, 5:95 → ACN/H_2O, 4:6) to provide the title compound **14c4** (16.2 mg, 65%) as a white solid. **TLC:** (DCM:MeOH = 20:1, v/v) R_f = 0.1 **^{1}H NMR:** (500 MHz, DMSO-*d6*): δ = 11.96 (s, 1H, NH), 8.82 (s, 1H, Ar-CH triazole), 8.32 (s, 1H, Ar-CH adjacent to Cl), 7.61 – 7.58 (m,1H, Ar-CH adjacent to NH), 6.99 (dd, *J* = 3.5, 2.0 Hz, 1H, Ar-CH), 4.55 (dd, *J* = 13.6, 6.1 Hz, 1H, Ha of CH_2), 4.35 (dd, *J* = 13.6, 7.7 Hz, 1H, Hb of CH_2), 3.34 (qd, *J* = 10.7, 5.8 Hz, 2H, Ha of CH_2 adjacent to OH + CH), 2.21 (dq, *J* = 13.0, 6.5 Hz, 1H, Hb of CH_2 adjacent to OH), 0.85 (d, *J* = 6.9 Hz, 3H, CH_3). **^{13}C NMR:** (126 MHz, DMSO-*d6*): = 147.4, 142.2, 141.2, 128.0, δ 127.7, 126.6, 119.3, 118.4, 101.7, 63.1, 52.5, 36.7, 14.3. **HPLC:** (5-100% Solvent B, 2 min): R_t = 1.475 min, purity (220 nm): > 99%. **HR-MS** (m/z): (ESI) calculated [M+H]+: 292.09596, found 292.09597

(S)-3-(4-(5-chloro-1H-pyrrolo[2,3-b]pyridin-4-yl)-1H-1,2,3-triazol-1-yl)butan-1-ol (**14c5**)

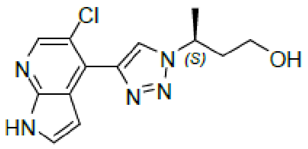

14c5 was synthesized following general procedure A starting from **13** (15.00 mg, 0.085 mmol, 1.00 equiv), (S)-3-azidobutan-1-ol **c5** (19.56 mg, 0.017 mmol, 2.00 equiv), sodium ascorbate (3.37 mg, 0.017 mmol, 0.20 equiv) and $CuSO_4(H_2O)_5$ (4.24 mg, 0.017 mmol, 0.20 equiv). Purification was carried out by preparative HPLC (ACN/H_2O, 5:95 → ACN/H_2O, 4:6) to provide the title compound **14c5** (4.9 mg, 20%) as a white solid. **TLC:** (DCM/MeOH = 20:1, v/v) R_f = 0.14. **^{1}H NMR:** (500 MHz, DMSO-*d6*): δ 11.96 (s, 1H, NH), 8.86 (s, 1H, Ar-CH triazole), 8.32 (s, 1H, Ar-CH adjacent to Cl), 7.59 (t, *J* = 3.0Hz, 1H, Ar-CH adjacent to NH), 6.99 (dd, *J* = 3.5, 1.9 Hz, 1H, Ar-CH), 5.04 – 4.94 (m, 1H, OH), 4.64 (t, *J* = 4.9 Hz, 1H, CH), 3.40 (dq, *J* = 10.9, 5.6 Hz, 1H, Ha of CH_2 adjacent to OH), 3.31 – 3.25 (m, 1H, Hb of CH_2 adjacent to OH), 2.14 (ddt, *J* = 14.1, 8.5, 5.7 Hz, 1H, Ha of CH_2), 2.03 (ddt, *J* = 13.6, 7.5, 5.9 Hz, 1H, Hb of CH_2), 1.60 (d, *J* = 6.8 Hz, 3H, CH_3). **^{13}C NMR:** (126 MHz, DMSO-*d6*): δ 147.4, 142.2, 141.2, 128.0, 127.8, 124.6, 119.3, 118.4, 101.7, 57.09, 54.1, 39.2, 21.0. **HPLC:** (5-100% Solvent B, 2 min): R_t = 1.471 min, purity (220 nm): >99 %. **HR-MS** (m/z): (ESI) calculated [M+H]+: 292.09596, found 292.09601.

(R)-3-(4-(5-chloro-1H-pyrrolo[2,3-b]pyridin-4-yl)-1H-1,2,3-triazol-1-yl)butan-1-ol (**14c6**)

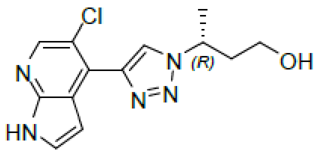

14c6 was synthesized following general procedure A starting from **13** (15.00 mg, 0.085 mmol, 1.00 equiv), (R)-3-azidobutan-1-ol **c6** (19.56 mg, 0.017 mmol, 2.00 equiv), sodium ascorbate (3.37 mg, 0.017 mmol, 0.20 equiv) and $CuSO_4(H_2O)_5$ (4.24 mg, 0.017 mmol, 0.20 equiv). Purification was carried out by preparative HPLC (ACN/H_2O, 5:95 → ACN/H_2O, 4:6) to yield the title compound **14c6** (7.2 mg, 29%) as a white solid. **TLC:** (DCM/MeOH = 20:1, v/v) R_f = 0.14. **^{1}H NMR:** (500 MHz, DMSO-*d*6): δ 11.96 (s, 1H, NH), 8.85 (s, 1H, Ar-CH triazole), 8.32 δ (s, 1H, Ar-CH adjacent to Cl), 7.59 (t, *J* = 3.0 Hz, 1H, Ar-CH adjacent to NH), 6.99 (dd, *J* = 3.5, 1.9 Hz, 1H, Ar-CH), 5.04 – 4.94 (m, 1H, OH), 4.65 (t, *J* = 4.9 Hz, 1H, CH), 3.40 (dq, *J* = 11.0, 5.7 Hz, 1H, Ha of CH_2 adjacent to OH), 3.31 – 3.23 (m, 1H, Hb of CH_2 adjacent to OH), 2.14 (ddt, *J* = 14.1, 8.4, 5.7 Hz, 1H, Ha of CH_2), 2.03 (ddt, *J* = 13.6, 7.5, 6.0 Hz, 1H, Hb of CH_2), 1.60 (d, *J* = 6.8 Hz, 3H, CH_3). **^{13}C NMR:** (126 MHz, DMSO-*d*6): δ 147.4, 142.2, 141.2, 128.0, 127.8, 124.6, 119.3, 118.4, 101.7, 57.1, 54.1, 39.2, 21.0. **HPLC:** (5-100% Solvent B, 2 min): R_t = 1.471 min, purity (220 nm): >99%. **HR-MS** (m/z): (ESI) calculated [M+H]+: 292.09596, found 292.09583.

(R)-3-(4-(5-chloro-1H-pyrrolo[2,3-b]pyridin-4-yl)-1H-1,2,3-triazol-1-yl)propane-1,2-diol (**14m**).

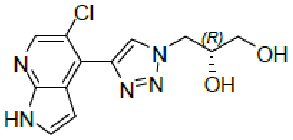

14m was synthesized following general procedure A starting from **13** (10.00 mg, 0.057 mmol, 1.00 equiv), (*R*)-3-azidopropane-1,2-diol **m** (11.45 mg, 0.011 mmol, 2.00 equiv), sodium ascorbate (2.24 mg, 0.011 mmol, 0.20 equiv) and $CuSO_4(H_2O)_5$ (2.83 mg, 0.011 mmol, 0.20 equiv). Purification was carried out by preparative HPLC (ACN/H_2O, 5:95 → ACN/H_2O, 4:6) to provide the title compound **14m** (5.2 mg, 31%) as a white solid. **TLC:** (DCM:MeOH = 9:1, v/v) R_f = 0.29 **^{1}H NMR:** (500 MHz, DMSO-*d*6): δ 11.96 (s, 1H, NH), 8.77 (s, 1H, Ar-CH triazole), 8.32 δ (s, 1H, Ar-CH adjacent to Cl), 7.60 (t, *J* = 3.0 Hz, 1H, Ar-CH adjacent to NH), 7.03 (dd, *J* = 3.5, 2.0 Hz, 1H, Ar-CH), 4.65 (dd, *J* = 13.8, 3.5 Hz, 1H, Ha of CH_2), 4.41 (dd, *J* = 13.8, 8.2 Hz, 1H, Hb of CH_2), 3.94 (dtd, *J* = 8.5, 5.6, 3.5 Hz, 1H, CH), 3.46 (dd, *J* = 11.0, 5.1 Hz, 1H, Ha of CH_2 adjacent to OH), 3.37 (dd, *J* = 11.0, 6.4 Hz, 1H, Hb of CH_2 adjacent to OH). **^{13}C NMR:** (126 MHz, DMSO-*d*6): δ 147.4, 142.2, 141.1, 128.0, 127.7, 126.9, 119.2, 118.3, 101.8, 70.4, 63.3, 53.1. **HPLC:** (5-100% Solvent B, 2 min): R_t = 1.269 min, purity (220 nm): > 99 %. **HR-MS** (m/z): (ESI) calculated [M+H]+: 294.07523, found 294.07507.

(S)-3-(4-(5-chloro-1H-pyrrolo[2,3-b]pyridin-4-yl)-1H-1,2,3-triazol-1-yl)propane-1,2-diol (**14n**)

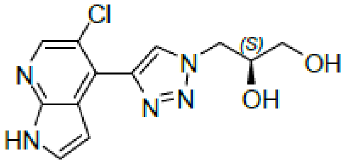

14n was synthesized following general procedure A starting from **13** (10.00 mg, 0.057 mmol, 1.00 equiv), (S)-3-azidopropane-1,2-diol **n** (13.26 mg, 0.011 mmol, 2.00 equiv), sodium ascorbate (2.24 mg, 0.011 mmol, 0.20 equiv) and $CuSO_4(H_2O)_5$ (2.83 mg, 0.011 mmol, 0.20 equiv). Purification was carried out by preparative HPLC

(ACN/H_2O, 5:95 → ACN/H_2O, 4:6) to provide the title compound **14n** (5.4 mg, 32%) as a white solid. **TLC:** (DCM/MeOH = 10:1, v/v) R_f = 0.29. **^{1}H NMR:** (500 MHz, DMSO-*d6*): δ 11.96 (s, 1H, NH), 8.76 (s, 1H, Ar-CH triazole), 8.32 (s, 1H, Ar-CH adjacent to Cl), 7.60 (t, *J* = 3.0 Hz, 1H, Ar-CH adjacent to NH), 7.03 (t, *J* = 2.7 Hz, 1H, Ar-CH), 4.65 (dd, *J* = 13.8, 3.5 Hz, 1H, Ha of CH_2), 4.41 (dd, *J* = 13.8, 8.2 Hz, 1H, Hb of CH_2), 3.93 (dtd, *J* = 8.6, 5.6, 3.7 Hz, 1H, CH), 3.46 (dd, *J* = 11.0, 5.1 Hz, 1H, Ha of CH_2 adjacent to OH), 3.36 (dd, *J* =11.0, 6.4 Hz, 1H, Hb of CH_2 adjacent to OH). **^{13}C NMR:** (126 MHz, DMSO-*d6*): δ 147.4, 142.2, 141.1, 128.0, δ 127.7, 126.9, 119.2, 118.3, 101.8, 70.4, 63.3, 53.1. **HPLC:** (5-100% Solvent B, 2 min): R_t = 1.278 min, purity (220 nm): 98%. **HR-MS** (m/z): (ESI) calculated [M+H]+: 294.07523, found 294.07511.

Synthesis of the amino-2-pyridine derivatives

4-bromo-5-chloropyridin-2-amine (**16**)

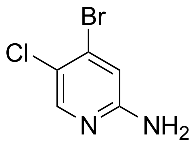

15 (3.25 g, 18.79 mmol, 1.00 equiv) was dissolved in DMF (40 mL) at -20°C. Then NCS (2.76 g, 20.67 mmol, 1.20 equiv) were added and the mixture was stirred from -20°C to rt for 5 h. Afterwards, the mixture was poured into iced-water (300 mL) and extracted with EA (3 x 100 mL), washed with 1.0 M NaOH (100 mL) and brine (100 mL). The solvents were removed under reduced pressure and the crude residue was purified twice with column chromatography (DCM/MeOH = 50:1 + 1% Et_3N) to give the title compound **16** (2.6 g, 67%) as a brown-yellow solid. **TLC:** (DCM/MeOH = 9:1, v/v + 1% Et_3N) R_f = 0.50. **^{1}H NMR:** (500 MHz, DMSO-*d*6): δ 8.01 (s, 1H, Ar-CH adjacent to Cl), 6.82 (s, 1H, Ar-CH adjacent to Br), 6.40 (s, 2H, NH_2). **^{13}C NMR:** (126 MHz, DMSO) δ 159.7, 147.7, 131.9, 117.7, 112.1. **HPLC:** (5-100% Solvent B, 2 min): R_t = 1.411 min, purity (220 nm): 98%.

5-chloro-4-(1-(tetrahydro-2H-pyran-2-yl)-1H-pyrazol-5-yl)pyridin-2-amine (**18**)

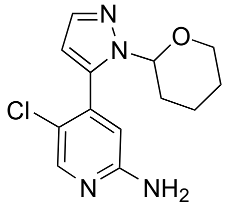

16 (150 mg, 0.72 mmol, 1.00 equiv) was dissolved in dioxane/H_2O (6:1 mixture, v/v, 7 mL) followed by the addition of 1-(tetrahydro-2H-pyran-2-yl)-5-(4,4,5,5-tetramethyl-1,3,2-dioxaborolan-2-yl)-1H-pyrazole **17** (603 mg, 2.17 mmol, 3.00 equiv), Pd(dppf)Cl_2 (30 mg, 0.04 mmol, 0.05 equiv), XPhos (34 mg, 0.07 mmol, 0.10 equiv) and K_2CO_3 (300 mg, 2.17 mmol, 3.00 equiv). The reaction mixture was heated up to 80°C overnight. Upon completion, the mixture was cooled to rt, filtered, diluted with H_2O (20 mL) and the acqueous layer extracted with EA (3 x 20 mL). The organic phase was dried over $MgSO_4$, filtered and concentrated under reduced pressure. The resulting crude residue was purified with column chromatography (CH/EA = 0-100% + 1% Et_3N) to obtain the title compound to give the title compound **18** (115 mg, 57%). 0.33 **TLC:** (CH/EA = 1:1, v/v) R_f = 0.33. **^{1}H NMR:** (500 MHz, $CDCl_3$) δ 8.07 (s, 1H, Ar-CH adjacent to Cl), 7.56 (d, J = 1.7 Hz, 1H, Ar-CH pyrazole), 6.54 – 6.44 (m, 1H, Ar-CH), 6.29 (d, J = 1.7 Hz, 1H, Ar-CH pyrazole), 4.98 (dd, J = 10.1, 2.5 Hz, 1H, CH of THP), 4.68 (s, 2H, NH_2), 3.92 (ddt, J = 11.6, 4.4, 2.2 Hz, 1H, CH_2 THP), 3.41 (td, J = 11.5, 2.5 Hz, 1H, CH_2 THP), 2.52 – 2.36 (m, 1H, CH_2 THP), 2.06 – 1.92 (m, 1H, CH_2 THP), 1.92 – 1.83 (m, 1H, CH_2 THP), 1.68 – 1.57 (m, 1H, CH_2 THP), 1.57 – 1.49 (m, 1H, CH_2 THP), 1.49 – 1.41 (m, 1H, CH_2 THP). **^{13}C NMR:** (126 MHz, $CDCl_3$) δ 157.1, 147.8, 139.6, 138.7, 138.3, 120.1, 108.1, 84.8, 67.8, 29.6, 24.8, 22.7. **HPLC:** (5-100% Solvent B, 2 min): R_t = 1.485 min, purity (220 nm): >99 %.

5-chloro-4-(1H-pyrazol-5-yl)pyridin-2-amine (**19**)

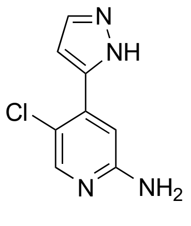

18 (84 mg, 0.30 mmol, 1.00 equiv) was dissolved in methanol (3.0 mL) and HCl (0.30 mL, 3.0 M, 0.90 mmol, 3.00 equiv) was added and the resulting mixture was stirred at room temperature overnight. Upon completion, the mixture was concentrated and the resulting residue was purified with preparative HPLC (ACN/H_2O, 5:95 → ACN/H_2O, 4:6) to obtain the title compound **19** (8mg, 14%). **TLC:** (EA/CH = 3:1, v/v) R_f = 0.31. **^{1}H NMR:** (500 MHz, DMSO-*d6*) δ 8.16 (s, 1H, Ar-CH adjacent to Cl), 7.91 (d, J = 2.4 Hz, 1H, Ar-CH pyrazole), 7.25 (s, 1H, Ar-CH), 6.91 (d, J = 2.3 Hz, 1H, Ar-CH pyrazole). **^{13}C NMR:** (126 MHz, DMSO-*d6*) δ 158.5, 158.2, 155.3, 140.9, 115.4, 109.4, 105.3. **HPLC:** (5-100% Solvent B, 2 min): R_t = 1.070 min, purity (220 nm): 91%. **HR-MS** (ESI): calculated (m/z) = 195.04320 [M + H^+], found (m/z) = 195.04307.

4-(2-amino-5-chloropyridin-4-yl)but-3-yn-2-ol (**20**)

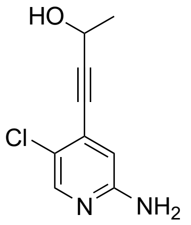

16 (300 mg, 1.45 mmol, 1.00 equiv), **7** (111 mg, 0.12 mL, 1.59 mmol, 1.10 equiv), $Pd(OAc)_2$ (3 mg, 0.01 mmol, 0.01 equiv), PPh_3 (19 mg, 0.07 mmol, 0.05 equiv), CuI (14 mg, 0.07 mmol, 0.05 equiv) and Et_3N (0.60 mL) were dissolved in MeCN (1.60 mL, degassed for 15 min) and the reaction mixture was stirred at 85°C for 20h. Upon completion, the reaction mixture was cooled down to rt and concentrated under reduced pressure. The residue was diluted with H2O (5 mL) and extracted with EA (3 x 5 mL). The organic phases were combined, dried over MgSO4, filtered and concentrated under reduced pressure. The crude product was purified with flash column chromatography (EA/CH = 2:1) to give the title compound **20** (144 mg, 51%); **TLC:** (EA/CH = 2:1, v/v) R_f = 0.15. **HPLC** (5-100% Solvent B, 2 min): R_t = 1.113 min, purity (220 nm): 98%.

4-(2-amino-5-chloropyridin-4-yl)but-3-yn-2-one (**21**)

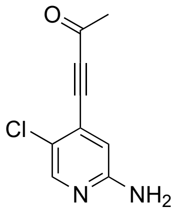

20 (92 mg, 0.47 mmol, 1.00 equiv), was dissolved in acetone (2.50 mL) at 0°C. The Jones reagent (0.25 mL; 0.54 ml/mmol, 0.54 equiv) was added and the reaction mixture was stirred for 2 h. Upon completion, isopropanol (5 mL) was added and the resulting mixture was diluted with H_2O (20 mL) and extracted with Et_2O (3 x 10 mL). The organic phases were combined, washed with a saturated aqueous solution of $NaHCO_3$ (1 x 15 mL) and brine (1 x 15 mL). The residue was dried over $MgSO_4$, filtered and the concentrated under reduced pressure. The crude was purified with flash column chromatography (CH/EA = 0-100%) to obtain the title compound **21** (46 mg, 51%). **TLC:** (CH/EA = 2:1, v/v) R_f = 0.33. **^{1}H NMR:** (500 MHz, $CDCl_3$): δ 8.12 (s, 1H, Ar-CH adjacent to Cl), 6.66 (s, 1H, Ar-CH), 4.67 (s, 2H, NH_2), 2.50 (s, 3H, CH_3). **^{13}C NMR:** (126 MHz, $CDCl_3$) δ 183.9, 156.7, 147.6, 129.0, 121.8, 112.1, 93.3, 83.47, 32.8. **HPLC:** (5-100% Solvent B, 2 min): R_t = 1.477 min, purity (220 nm): > 99%.

5-chloro-4-(3-methyl-1H-pyrazol-5-yl)pyridin-2-amine (**22**).

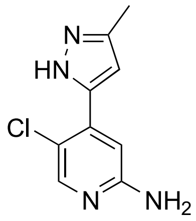

21 (46 mg, 0.24 mmol, 1.00 equiv) was suspended in EtOH (1.0 mL). Then $NH_2NH_2{\cdot}H_2O$ (13 mg, 0.26 mmol, 0.06 mL, 1.10 equiv) was added and the reaction mixture was stirred at 80 °C. Upon completion, the reaction was cooled down to rt, the reaction mixture was concentrated under reduced pressure and the residue was diluted with H_2O (10 mL), extracted with EA (3 x 5 mL). The organic phases were combined, dried over $MgSO_4$, filtered and the solvent was removed under reduced pressure. The resulting crude residue was purified with preparative HPLC (ACN/H_2O, 5:95 → ACN/H_2O, 4:6) to give the title compound **22** (17 mg, 35%). **TLC:** (CH/EA = 1:1, v/v) R_f = 0.30. **^{1}H NMR:** (500 MHz,DMSO-*d*6): δ 7.95 (s, 1H, Ar-CH adjacent to Cl), 6.90 (s, 1H, Ar-CH), 6.55 (s, 1H, Ar-CH pyrazole), 6.11 (s, 2H, NH_2), 2.28 (s, 3H, CH_3). **^{13}C NMR** (126 MHz, $CDCl_3$) δ 163.5, 159.3 148.1, 140.1, 115.9, 107.5, 105.5, 11.2. **HPLC** (5-100% Solvent B, 2 min): R_t = 1.242 min, purity (220 nm): 95%. %. **HR-MS** (ESI): calculated (m/z) = 209.05885 [M + H^+], found (m/z) = 209.05901.

Biochemistry and Biology

APH expression and purification and structure determination, Thermal shift assay, enzymatic assay, and Antibiotic susceptibility assays

These methods are described in details in the accompanying manuscript[7].

K_i measurements

K_i values were measured at 25°C using the coupled pyruvate kinase/lactate dehydrogenase (PK/LDH) enzyme system described in the accompanying manuscript[7]. Final concentrations were 0.1 µM APH(2'')-IVa, 100 µM kanamycin A, 0.1 to 2 mM MgATP (12 different concentrations), 2 mM phosphoenol pyruvate, 140 µM NADH and 1× PK/LDH. The inhibitors were tested at 4 to 8 different concentrations from 0 to 10 µM. Time courses were fitted globally with GraFit software (v 7.0.3, Erithacus Software Limited) using a competitive inhibition equation.

Uptake assays

Bacterial strains

The following strains were used: *S. aureus* ATCC 33591 (MRSA), *K. pneumoniae* ATCC 43816, *P. aeruginosa* ATCC 27853, *E. coli* ATCC 25922, *E. faecalis* ATCC 29212 and an *E. coli* Δ*tolC* strain as described previously.[2] The *E. coli* Δ*tolC* strain was kindly provided by Prof. Mark Brönstrup, Department of Chemical Biology, Helmholtz Centre for Infection Research. *P. aeruginosa* and *E. coli* strains were grown in LB medium (the Δ*tolC* strain was supplemented with kanamycin as described previously).[2] *K. pneumoniae* was grown in MHB medium. *E. faecalis* and *S. aureus* strains were grown in BHI medium.

Cellular uptake assay

Cellular uptake was performed as previously established for *E. coli*.[2] All compounds were incubated with bacteria at terminal concentrations of 1 mg·mL^{-1} with a maximal DMSO content of 1%. To avoid differences in bacterial growth, a growth control without compound, but 1% DMSO was used. The assays were performed in triplicate. Cellular uptake of the different fractions was determined using HPLC-MS/MS. For all compounds, a calibration curve and quality control (QC) samples were prepared using the same buffer as used for the subcellular fractions as matrix. Then 50 µl of the calibration or QC or subcellular fraction sample were extracted with 50 µl acetonitrile including 1 µg·mL^{-1} caffeine as internal standard for 5 min at 800 rpm on an Eppendorf MixMate® vortex mixer. Then, samples were spun down at 4000 rpm for 20 min at 4°C. Supernatants were transferred to standard 96well Greiner V-bottom plates and sealed with a septum mat. Ciprofloxacin was used as positive control for *E. coli* wild-type and *E. coli* Δ*tolC* strain (Supplementary Figure S1) as described previously.[2]
Samples were analysed using an Agilent 1290 Infinity II HPLC system coupled to an AB Sciex QTrap 6500plus mass spectrometer. LC conditions were as follows: column: Agilent Zorbax Eclipse Plus C18, 50 × 2.1 mm, 1.8 µm; temperature: 30°C; injection volume: 5 µL per sample; flow rate: 700 µL·min^{-1}. Samples were run under the following conditions. Solvents for acidic conditions: A: 100% water + 0.1% HCOOH; solvent B: 95% ACN + 5% water + 0.1% HCOOH. The gradient was as follows: 99% A at 0 min, 99% A until 1 min, 99–0% A from 1 min to 2.2 min, 0% A until 3.2 min. Mass transitions are depicted in Table S1. Peaks of samples were quantified using the calibration curve. The accuracy of the calibration curve was determined using QCs independently prepared on different days.

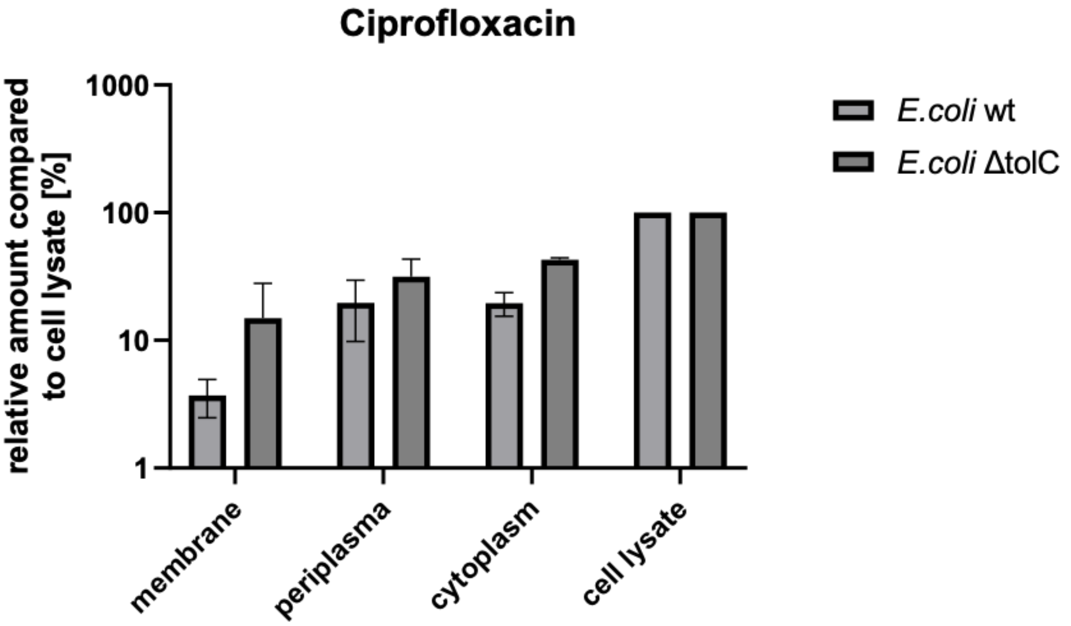

Supplementary Figure 1. Cellular uptake of ciprofloxacin in *E. coli* wild-type and *E. coli ΔtolC* strains. The relative amount of ciprofloxacin in membrane, periplasm, cytoplasm and total cell lysate is shown and expressed as a percentage of the amount detected in the cell lysate.

Supplementary Table 1: Mass transitions for HPLC-MS/MS of APH-inhibitors.

Compound name	Q1 [m/z]	Q3 [m/z]	DP [V]	CE [V]	CXP [V]
**1**	218.858	156	1	33	16
		183	1	29	20
		155	1	41	16
**14a**	219.989	165	101	29	16
		157	101	29	18
**14e**	334.069	192	1	29	22
		57	1	57	8
**14k**	310.043	90.9	1	27	10
		191	1	27	22
**25**	367.057	191	126	31	20
		192	126	29	22
		91	126	73	10
**10**	232.994	170	1	31	18
		197	1	29	20
**14b**	264.008	192	106	25	22
		165	106	37	18
**14c**	278.023	192	1	25	22
		165	1	39	18
**14d**	277.983	191	86	25	22
		156	86	47	16
**14j**	297.031	269	71	21	22
		233	71	29	26

**14h**	314.023	251	81	29	26
		286	81	23	24
**14i**	314.011	286	96	23	24
		251	96	29	30
**26**	397.098	121	126	31	14
		77.9	126	113	8
**24**	277.029	192	86	27	22
		191	86	25	20
		156	86	49	16
**14g**	310.069	247.1	111	29	22
		282	111	21	16
**14f**	296.048	233	111	31	22
		268	111	21	24
**14c2**	292.003	192	1	27	20
		165	1	41	16
	289.961	188.9	-10	-22	-21
		218	-10	-18	-11
**14c3**	292.004	192	1	29	22
		165	1	41	18
		219.9	1	25	12
	289.963	188.8	-15	-24	-19
		232	-15	-20	-11
**14c4**	291.993	192	1	27	24
		165	1	41	22
		157	1	43	16
	289.961	188.9	-20	-24	-21
		189.4	-20	-20	-19
		232	-20	-20	-11
Caffeine	195.024	138	130	25	14
		110	130	31	18

Determination of *in vivo* ADME properties

ADME assays (plasma stability, metabolic stability and plasma protein binding)

Plasma stability assay, the plasma protein binding and the metabolic stability assay were conducted as described previously.[3] For all compounds, 1 µg·mL^{-1} were used terminally in each assay. Samples were extracted as described previously for the respective assays.[3] Then samples were subjected to HPLC-MS/MS analysis using the same gradient and the same MS/MS conditions as described for the cellular uptake assay.

Human kinase panel screening

DSF-based selectivity screening against a curated kinase library

The assay was performed as previously described.[45] Briefly, recombinant protein kinase domains at a concentration of 2 µM were mixed with 10 µM compound in a buffer containing 20 mM HEPES, pH 7.5, and 500 mM NaCl. SYPRO Orange (5000×, Invitrogen) was added as a fluorescence probe (1 µL per mL). Subsequently, temperature-dependent protein unfolding profiles were measured using the QuantStudio™ 5 realtime PCR machine (Thermo Fisher). Excitation and emission filters were set to 465 nm and 590 nm, respectively. The temperature was raised with a step rate of 3°C per minute. Data points were analysed with the internal software (Thermal Shift SoftwareTM Version 1.4, Thermo Fisher) using the Boltzmann equation to determine the inflection point of the transition curve.

Supplementary Table 2. Full kinase panel screening of compounds **1**, **14c**, **14f**, **14m**, **14b3-4**, **14c2-4** and **24**.

Entry	14f	14c4	14c	14b3	14c2	14c3	14m	14b4	24	1	Reference Shift (°C)	Reference (nM)	Reference (compound)
**ABL1**	1.2	2.0	1.5	1.7	1.4	2.1	1.6	1.7	1.3	1.9	8.6	60	Staurosporine
**BMX**	-0.3	0.5	0.4	0.1	0.3	0.5	0.2	0.0	0.0	0.3	6.8	170	Staurosporine
**CASK**	0.7	0.1	0.2	0.1	-0.1	-0.5	-0.7	-0.5	-0.1	0.1	5.2	19	Staurosporine
**CK1d**	1.4	3.7	4.5	3.9	3.8	3.8	4.1	4.7	3.8	1.8	9.0	8	PF-670462
**CK2a1**	1.2	2.0	1.4	1.0	1.2	1.8	1.0	1.3	0.7	0.7	14.6	1	Similtasertib
**BMPR2**	5.4	6.4	5.6	5.3	5.1	6.2	4.8	5.4	3.8	4.0	3.0	670	Staurosporine
**CDK2**	0.0	1.5	0.9	1.5	0.6	1.7	0.2	1.3	0.0	-0.2	15.2	1	Staurosporine
**BRAF**	0.5	1.2	1.2	1.0	1.0	1.3	0.9	1.1	0.9	1.0	26.6	1	Dabrafenib
**AAK1**	4.5	5.4	6.0	5.1	6.0	5.8	5.2	4.8	4.8	5.4	15.3	1	Staurosporine
**CAMK2B**	-0.1	0.9	0.4	0.7	0.0	0.1	0.7	0.0	0.0	-0.5	11.3	0	Staurosporine
**CAMK2D**	-0.4	-0.9	-0.4	-0.1	-0.6	0.0	-0.9	-0.5	0.1	-0.6	15.8	1	Staurosporine
**CAMK4**	-0.1	1.0	0.6	-2.5	0.3	0.6	-0.9	0.1	-1.0	-1.3	8.2	141	Staurosporine
**MRCKa**	0.1	0.2	0.2	0.5	0.2	0.1	0.2	0.1	0.1	0.1			
**CLK3**	1.0	1.7	1.2	1.3	0.6	1.4	0.8	0.9	0.7	0.9	15.0	110	CLK-T3
**DYRK2**	3.5	3.2	3.1	3.9	2.2	3.2	3.9	4.7	3.4	2.3	7.3	280	Staurosporine
**CDKL1**	-0.4	0.2	0.3	0.6	0.0	0.5	0.2	0.5	-0.1	-0.3	6.6	0	CEP-32496
**CHK2**	1.9	2.8	2.5	2.2	2.4	2.4	2.7	2.2	2.4	2.9	17.1	0	Staurosporine
**CLK1**	3.5	5.3	4.1	4.1	3.8	4.8	3.5	4.5	3.3	2.4	15.7	4	Staurosporine
**DAPK1**	3.0	3.2	2.7	2.4	2.6	3.1	2.2	2.5	1.5	1.1	9.0	4	Staurosporine
**CK2a2**	0.7	2.5	1.8	1.5	2.0	2.4	1.6	1.7	1.2	0.8	15.7	1	Similtasertib
**DAPK3**	4.7	5.6	4.7	4.6	5.3	5.6	4.5	5.5	4.3	1.6	16.2	1	Staurosporine
**FES**	-0.7	1.0	0.6	0.9	0.8	1.0	0.5	0.6	0.7	1.5	7.5	2	Staurosporine
**Haspin**	1.0	2.3	2.7	3.2	1.2	1.1	3.2	2.7	3.0	1.3	8.9	50	Staurosporine
**TTK**	1.4	2.2	1.6	1.9	2.2	2.6	1.6	1.7	1.4	0.8	9.0	61	Staurosporine
**EphA2**	-0.6	-0.3	-0.3	-0.4	-0.3	-0.2	-0.4	-0.4	-0.3	-0.3	7.1	53	Staurosporine
**Erk2**	0.2	0.8	0.7	0.6	0.6	0.9	0.6	0.5	0.5	0.0	7.5	1	GDC-0994
**MER**	-0.1	0.7	0.3	0.4	0.0	0.4	0.2	0.0	-0.6	-0.6	5.5	6	Staurosporine
**MST3**	-0.9	-1.0	-0.9	-1.0	-1.9	-1.5	-1.5	-1.6	-1.8	-1.5	5.5	120	Staurosporine

**DCAMKL1**	0.6	1.1	0.6	0.6	0.1	0.6	-0.2	0.0	-0.5	-0.9	11.4	120	Staurosporine
**DMPK1**	0.9	2.0	1.5	1.6	1.6	2.0	1.4	1.6	1.0	0.9	9.3	23	Staurosporine
**EPHA5**	-0.5	-0.4	-0.4	-0.4	-0.6	-0.6	-0.6	-0.6	-0.7	-0.6	6.9	19	Staurosporine
**EPHA7**	1.3	1.5	1.1	1.2	0.9	1.3	0.9	0.9	0.7	0.4	10.5	30	Staurosporine
**EPHB3**	-2.3	-0.8	0.1	1.3	-1.5	-1.1	-1.8	-1.6	-1.6	-1.9	6.1	825	Staurosporine
**FGFR1**	0.7	1.3	1.1	1.6	1.2	1.2	1.3	1.4	1.1	1.8	5.7	3	Staurosporine
**FGFR2**	-0.3	-0.2	-0.2	-0.1	0.0	-0.1	-0.2	-0.1	-0.1	-0.1	8.3	3	Staurosporine
**GAK**	1.1	3.1	2.3	3.1	3.3	3.4	2.3	2.6	2.4	1.5	8.8	17	Staurosporine
**GPRK5**	0.3	1.5	1.1	1.1	0.1	1.1	1.2	0.9	1.0	2.3	5.9	150	Staurosporine
**MAP2K1**	0.1	0.7	0.6	0.3	0.3	1.2	0.3	0.2	0.2	-0.1	3.2	24	Staurosporine
**MEK4**	3.2	4.7	4.3	4.5	3.9	4.7	4.4	4.5	4.5	2.9	10.5	1	Staurosporine
**MAP2K6**	1.9	4.1	3.7	3.6	3.3	4.1	3.6	3.8	3.7	2.1	11.3	1	Staurosporine
**MAP3K5**	4.9	6.3	6.6	6.5	5.0	5.2	6.4	7.1	9.4	6.5	15.5	24	Staurosporine
**JNK3**	1.2	1.8	1.4	1.9	1.4	1.7	1.7	2.1	1.4	1.5	8.8	0	CEP-32496
**p38d**	0.2	0.9	0.5	0.5	0.6	1.1	0.6	0.6	0.3	0.3			
**p38a**	-0.7	-0.5	-0.6	-0.6	-0.8	-0.7	-0.7	-0.6	-0.7	-0.7	19.8	1	Doramapimod
**JNK1**	1.1	1.7	1.3	1.8	1.3	1.6	1.4	1.8	0.8	0.9	7.7	220	Staurosporine
**JNK2**	-0.1	0.6	0.3	0.6	0.0	0.2	0.4	0.3	0.1	-0.3	8.6	0	SBI-0069279
**MARK3**	1.3	2.1	2.4	2.5	-0.7	1.2	2.3	2.6	1.9	1.5	18.0	1	Staurosporine
**MARK4**	2.3	2.4	2.3	2.0	1.7	2.1	2.0	2.0	1.7	1.1	14.3	1	Staurosporine
**MELK**	1.9	4.3	4.1	3.6	3.3	4.1	3.7	3.4	3.2	2.5	13.2	1	Staurosporine
**PIM3**	1.4	1.2	1.1	0.9	0.9	0.9	0.8	-0.4	-0.3	-0.1	19.0	0	Staurosporine
**FLT1**	3.2	3.9	3.9	3.8	3.9	4.4	3.6	3.7	4.6	3.0	12.0	11	Staurosporine
**NEK1**	0.8	0.7	1.0	1.1	-0.2	1.7	0.7	0.3	0.6	0.3			
**NEK2**	-1.0	-3.3	-1.6	-0.1	-0.5	-0.7	-1.5	-1.8	-1.0	-1.5	4.3	650	Staurosporine
**NEK7**	-0.3	-0.3	-0.3	-0.6	-0.7	-0.5	-0.5	-1.2	-1.0	-1.1			
**OSR1**	0.3	2.8	0.4	0.5	2.7	1.5	1.0	0.4	-0.1	-0.7	5.7	91	Staurosporine
**PAK1**	-0.4	-0.6	-0.5	-0.5	-0.5	-0.4	-1.0	-0.9	-0.9	-0.7	7.3	0	Staurosporine
**MST4**	-0.7	-0.4	-0.6	-0.1	-1.2	-0.9	-1.2	-0.9	-1.5	-1.6	6.0	7	Staurosporine
**PAK4**	1.8	3.3	2.7	2.5	2.8	3.3	2.5	2.7	1.7	3.6	12.2	6	Staurosporine
**PHKg2**	0.7	1.5	1.5	1.4	0.9	1.1	1.1	0.9	1.1	2.9	21.2	0	Staurosporine
**PIM1**	0.5	1.2	0.9	0.7	0.5	1.0	0.8	0.6	0.2	0.7	11.9	3	Staurosporine

**RSK1_b**	1.5	2.0	1.2	1.3	0.9	1.5	1.0	1.0	1.2	0.2	3.2	0	Staurosporine
**LOK**	-1.5	-1.3	-1.3	-1.1	-1.8	-1.7	-1.5	-1.6	-1.9	-1.6	23.5	0	Staurosporine
**ATK3**	0.2	0.2	0.0	0.3	-0.1	0.2	-0.2	0.2	0.0	0.1	7.0	5	Staurosporine
**BIKE**	8.5	10.2	10.2	9.2	10.0	10.2	9.1	8.8	8.7	9.6	18.4	1	Staurosporine
**CAMK1D**	1.2	1.6	1.4	1.3	1.0	1.6	1.6	0.9	1.4	1.0	8.9	0	Staurosporine
**CAMK1G**	0.9	2.2	1.7	1.8	1.2	1.9	1.6	1.5	1.4	1.0	8.8	23	Staurosporine
**PCTAIRE1**	0.0	0.6	0.0	0.1	0.6	0.6	-0.1	0.1	-0.6	-0.2	9.0	14	Staurosporine
**MYT1**	-0.5	-0.4	-0.5	-0.7	0.8	-0.4	-0.6	-0.6	-0.7	-0.7	4.5	130	Dasatinib
**PLK4**	3.7	4.5	3.8	3.8	4.2	4.2	3.1	3.5	2.7	3.6	18.0	4	Staurosporine
**MSK1_b**	4.7	7.5	8.0	7.1	6.1	6.1	8.0	7.2	5.6	4.1	15.6	5	Staurosporine
**SLK**	-0.6	-0.4	-0.2	-0.2	-0.4	0.1	-0.6	-0.9	-0.6	-0.4	16.9	4	Staurosporine
**SRC**	-0.3	0.2	-0.1	0.2	1.1	1.4	0.1	-0.1	-0.2	-0.2	5.0	2	Staurosporine
**SRPK1**	-0.3	0.3	-0.2	0.0	0.0	0.4	-0.2	-0.1	-0.3	-0.4	7.0	120	Staurosporine
**MST2**	0.7	2.3	2.1	2.7	1.2	2.5	3.0	2.3	0.8	1.5	13.4	0	Staurosporine
**MST1**	1.0	2.5	2.3	2.7	2.3	2.0	2.4	2.0	1.2	1.1	15.5	1	Staurosporine
**AurA**	5.5	8.1	7.4	7.0	7.1	7.6	7.0	7.0	7.2	6.9	16.5	2	Staurosporine
**DRAK1**	8.0	4.8	4.9	4.0	4.6	4.5	2.6	2.9	4.5	3.0	7.7	14	Staurosporine
**DRAK2**	1.6	2.9	2.2	2.2	1.7	3.0	2.6	1.8	1.1	0.5	10.8	21	Staurosporine
**NDR2**	-1.1	-1.0	-0.7	-0.8	-1.1	-1.0	-0.9	-1.0	-1.1	-1.1	11.6	1	Staurosporine
**STLK3**	0.6	1.4	1.1	1.3	1.1	1.1	1.2	0.9	0.9	1.5	11.4	22	Staurosporine
**BRD4**	4.9	1.6	1.7	1.7	0.5	1.5	1.4	0.8	0.8	1.1	7.0	60	JQ1
**BRPF1**	-0.9	-0.1	-0.5	-0.1	-1.4	-1.0	-1.2	-1.2	-1.4	-0.8	14.0	20	GSK6853
**TAF1**	2.1	0.7	-0.2	-0.8	0.7	2.3	0.0	-15.0	-15.1	0.7	7.4	0	Bromosporine
**TIF1**	-0.3	-0.5	-0.5	-0.4	-0.3	-0.4	-0.3	-0.5	-0.5	-0.4			
**ULK1**	2.9	3.8	2.8	2.8	2.2	3.3	2.6	2.8	1.8	2.2	12.0	0	Staurosporine
**ULK3**	8.9	7.1	6.1	6.3	6.1	7.1	5.6	6.3	4.4	4.4	17.3	2	Staurosporine
**VRK1**	-0.5	0.1	0.1	-0.1	-0.5	-0.3	-0.3	-0.4	-0.5	-0.4			
**WNK1**	-0.6	-0.1	-0.2	-0.2	-0.3	0.0	-0.2	0.3	-0.2	-0.2			
**CSNK1E**	1.1	3.1	3.4	2.8	2.9	2.9	2.9	3.6	2.3	1.0	8.0	14	PF-670462
**EPHA4**	0.2	0.4	0.3	0.2	-0.5	0.5	0.0	0.5	-0.2	0.3	5.3	7	Staurosporine
**FECH**	-2.4	-0.7	-1.5	-0.7	-3.1	-1.1	-1.2	-1.8	7.0	-0.6	5.5	1000	Vemurafenib
**HIPK2**	1.2	1.9	1.3	0.9	1.0	1.6	1.0	1.1	0.9	0.3	4.2	791	Staurosporine

**MAP2K7**	-0.4	0.0	-0.3	0.0	-0.8	-0.6	-0.7	-0.6	-0.4	-0.3	7.0	440	Staurosporine
**EPHB1**	0.0	0.4	0.3	0.3	0.2	0.5	0.2	0.3	0.2	0.6	6.4	25	Staurosporine
**AURKB**	4.9	6.3	6.1	6.0	6.1	6.0	6.0	5.8	6.0	6.5	8.0	5	Staurosporine
**DYRK1A**	1.2	1.6	1.7	1.4	2.1	1.6	1.0	1.2	1.3	1.4	9.9	4	Staurosporine
**MAPK15**	1.9	4.5	4.0	3.2	3.9	4.9	2.7	3.0	2.6	1.6	14.0	6	Staurosporine
**MAPKAPK 2**	-0.3	-0.1	0.0	0.5	-0.5	-0.3	-2.3	0.1	0.0	-0.3	4.0	196	Staurosporine
**MSSK1**	-0.2	0.2	-0.1	-0.1	-0.2	0.0	-0.6	-0.1	-0.4	-0.4			
**NQO2**	-0.8	-0.5	-0.5	-0.3	-0.7	-0.6	-0.7	-0.7	-0.7	-0.9		43	Imatinib
**TLK1**	-1.0	-0.5	-0.9	-0.4	-1.3	-1.1	-1.2	-1.1	-1.4	-1.3	8.9	44	Staurosporine

Waterfall plot of compound **1**

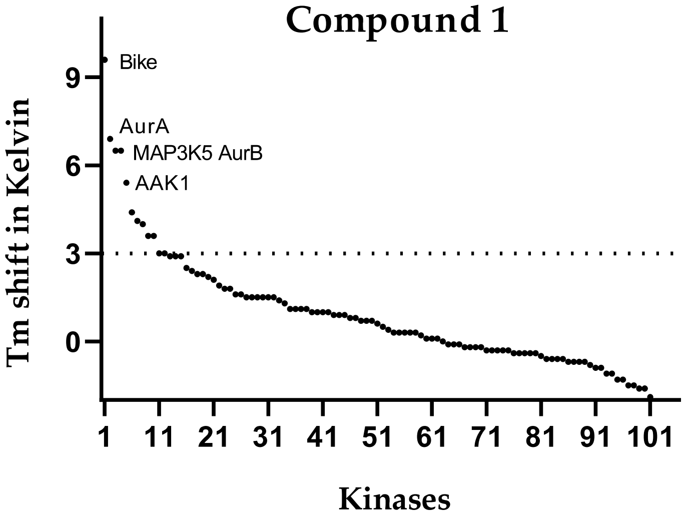

Supplementary Figure 2. Waterfall plot for data from the human kinase panel screening, determined by thermal shift assays in absence or presence of 10 μM of compound **1**.

Inhibition data of compound **1** for Erk-2

Thermo Fisher Scientific's SelectScreen™ Profiling Service: Single Point Results

SelectScreen Scientist: Joseph Vance **Date:** 23/02/2021 **SSBK-Z'-LYTE (Madison, WI USA)**

Quality Assurance Review: Christiana Holguin **Date:** 23/02/2021

Legend

< 40% Inhibition
40% - 80% Inhibition
≥ 80% Inhibition

% Phosphorylation	Pass
Z' Determination	Pass

Project #	Compound Name	1X Test Compound Concentration (nM)	[ATP] Tested (μM)	Kinase Tested	% Inhibition Point 1	% Inhibition Point 2	% Inhibition mean	Difference Between Data Points |Point 1 - Point 2|	Development Reaction Interference	Test Compound Interference Coumarin	Test Compound Interference Fluorescein	Z'	Kinase Part# / Lot#
SSBK13778_59739	L44	1000	Km app	MAPK1 (ERK2)	6	7	6.5	0	Pass	Pass	Pass	0.93	PV3313/1744814

Supplementary Figure 3. **1** (1 μM) inhibited recombinant human Erk-2 to 6% as determined in a Z'-LYTE assay (single concentration, duplicate, ATP concentration at $K_{m\ app}$) performed by Eurofins (Madison, WI USA).

Bacterial Growth Assessment in the Presence of Antibiotics and Test Compounds

Bacterial Strains and Culture Conditions

Pseudomonas aeruginosa C0214 was pre-cultured in Mueller-Hinton (MH) broth at 37°C under agitation overnight. The optical density at 600 nm (OD_{600}) was then measured to adjust the bacterial concentration. An initial suspension was prepared at an OD_{600} of 0.1 (~1×10^7 CFU·mL^{-1}) and further diluted to obtain a final working concentration of 5×10^5 CFU·mL^{-1}.

Preparation of compounds and antibiotic

Test compounds were initially dissolved at 100 mM in 100% DMSO and serially diluted to obtain a 2 mM intermediate stock solution in 4% DMSO. Working concentrations (125, 250, and 500 µM) were then prepared by additional twofold dilutions in the same solvent. Finally, 25 µL of each solution was added to the wells, yielding final concentrations of 0, 125, 250, and 500 µM with a DMSO concentration of 1%. Kanamycin was prepared as an 8,192 mg·L^{-1} stock solution in sterile water and serially diluted twofold. For each condition, 50 µL of the diluted solution was added to the wells. Final concentrations ranged from 4,096 mg·L^{-1} to 8 mg·L^{-1}.

Bacterial growth assay

Bacterial suspensions were added to 96-well plate in a final volume of 100 µL per well with the following setup: Column 1: Positive control (MH medium + compound + bacteria, no antibiotic); Columns 2 to 11: Antibiotic conditions with decreasing kanamycin concentrations (4,096 to 8 mg·L^{-1}); Column 12: Negative control (MH medium + compound, no bacteria). Plates were incubated at 37°C for 24 h, and OD600 was measured using an Infinite M Nano absorbance reader (Tecan, Männedorf, Switzerland) each hour. The characteristics of the strain used, *P. aeruginosa* C0214 from Hamilton General Hospital (Canada), are given in the accompanying manuscript[7].

Each test compound was evaluated at four concentrations (0, 125, 250, and 500 µM), with experimental duplicates performed across two rows. Analysis and representations were conducted using GraphPad Prism 10.1.0. MIC were determined

Bacterial growth curve for compound **10**, **14n** and **14c**.

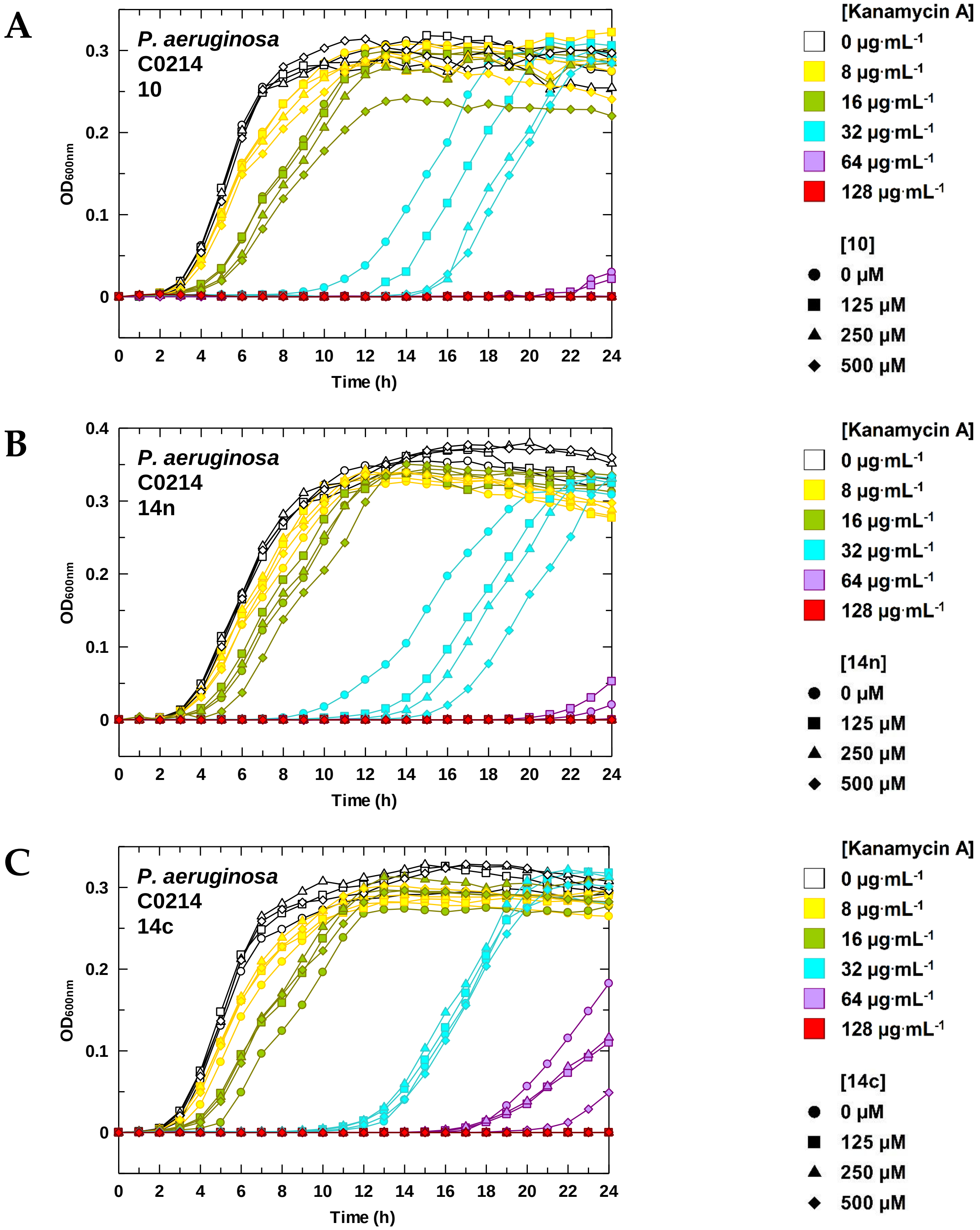

Supplementary Figure 4. Growth curves of *Pseudomonas aeruginosa* C0214 in the presence of increasing concentrations of kanamycin and **A**) **10**, **B**) **14n** or **C**) **14c**. Different kanamycin concentrations (ranging from 0 to 128 µg·mL^{-1}) are indicated by distinct colors, while varying concentrations of APH-ligand (0 µM, 125 µM, 250 µM and 500 µM) are represented by different symbols. In the absence of kanamycin, bacteria exhibited normal growth (black curves). For clarity, growth curves obtained at 256-1024 µg·mL^{-1} Kanamycin A are not shown as no growth were observed.

X-ray crystallography and structure refinement

The methods used with APH are described in detail in the accompanying manuscript[7]. X-ray data and refinement statistics are given in Tables S3-S9.

Erk-2 was produced, purified and crystallized as described in Gelin *et al.*[6]

Additional figures

Compound **1** in complex with Erk-2.

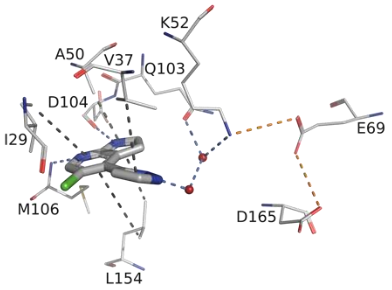

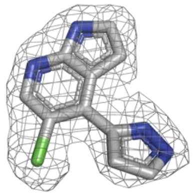

1

Supplementary Figure 5. Crystal structure of Erk-2 in complex with **1** (PDB 9QQJ) and corresponding omit map contoured at a sigma level of ± 1.

Compound **19** in complex with APH(2")-IVa.

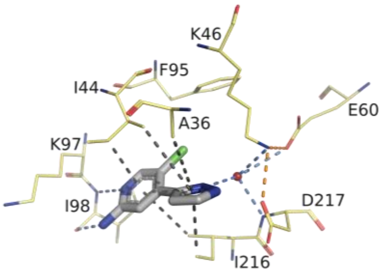

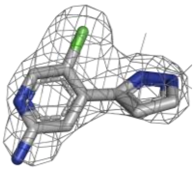

19

Supplementary Figure 6. Crystal structure of APH(2")-IVa in complex with **19** (PDB 9QOR) and corresponding omit map contoured at a sigma level of ± 1.

Supplementary Tables 3-10: Data collection and refinement statistics

Supplementary Table 3. Data collection and refinement statistics (molecular replacement).

Ligand name	**19**	**14a**
(PDB accession code)	9QOR	9QOT
**Data collection**		
Space group	$P2_1$	$P2_1$
Cell dimensions		
a, *b*, *c* (Å)	78.10 65.11 73.98	75.56 64.63 77.14
α, β, γ (°)	90 90.8 90	90 88.9 90
Resolution (Å)	50.01-2.44 (2.53-2.44)*	38.56 -2.30 (2.38-2.30)*
R_{merge}	0.037 (0.384)	0.091 (0.505)
R_{means}	0.052 (0.543)	0.111 (0.609)
R_{pim}	0.037 (0.384)	0.063 (0.336)
I / ◎*I*	14.05 (2.26)	11.97 (2.76)
Completeness (%)	98.33 (98.78)	99.75 (99.88)
Redundancy	1.9 (1.9)	3.2 (3.2)
$CC_{1/2}$	0.998 (0.838)	0.970 (0.790)
CC^*	1.000 (0.955)	0.992 (0.940)
Wilson B-factor	51.56	40.19
**Refinement**		
Resolution (Å)	2.44	2.30
No. reflections	52.054 (5349)	105269 (10470)
Unique reflections	27391 (2752)	33242 (3315)
R_{work} / R_{free}	20.69/27.90	21.16/24.99
No. atoms		
Protein	4836	4842
Ligand/ion	26	70
Water	25	69
B-factors		
Protein	66.27	48.56
Ligand/ion	54.86	51.10
Water	56.91	46.17
R.m.s. deviations		
Bond lengths (Å)	0.008	0.008
Bond angles (◎)	1.02	0.92
Number of TLS	10	9

*Values in parentheses are for the highest-resolution shell.

Supplementary Table 4. Data collection and refinement statistics (molecular replacement).

Ligand name	**14k**	**14b**
(PDB accession code)	9QOU	9QOX
**Data collection**		
Space group	$P2_1$	$P2_1$
Cell dimensions		
a, *b*, *c* (Å)	75.79 64.83 77.56	74.95 64.80 77.66
α, β, γ (°)	90 91.7 90	90 91.9 90
Resolution (Å)	37.88-2.55 (2.64-2.55)*	54.84 -2.06 (2.13-2.06)*
R_{merge}	0.048 (0.622)	0.052 (0.887)
R_{means}	0.069 (0.880)	0.065 (1.098)
R_{pim}	0.048 (0.622)	0.037 (0.637)
I / ◎*I*	10.75 (1.22)	15.77 (0.59)
Completeness (%)	98.97 (99.55)	97.13 (85.28)
Redundancy	2.0 (2.0)	3.0 (3.0)
$CC_{1/2}$	0.997 (0.592)	0.964 (0.860)
$CC^{*0.996\ (0.859)}$	0.999 (0.862)	0.991 (0.962)
Wilson B-factor	57.60	48.71
**Refinement**		
Resolution (Å)	2.55	2.06
No. reflections	48829 (4820)	137354 (13627)
Unique reflections	24518 (2415)	46052 (3912)
R_{work} / R_{free}	19.81/25.54	22.01/26.63
No. atoms		
Protein	4854	4833
Ligand/ion	72	56
Water	44	67
B-factors		
Protein	63.51	61.51
Ligand/ion	64.56	61.32
Water	58.11	52.88
R.m.s. deviations		
Bond lengths (Å)	0.009	0.008
Bond angles (◎)	1.01	0.99
Number of TLS	11	6

*Values in parentheses are for the highest-resolution shell.

Supplementary Table 5. Data collection and refinement statistics (molecular replacement).

Ligand name	**24**	**14f**
(PDB accession code)	9QOW	9QOZ
**Data collection**		
Space group	$P2_1$	$P2_1$
Cell dimensions		
a, *b*, *c* (Å)	77.76 64.38 74.06	78.46 64.70 78.60
α, β, γ (°)	90 90.9 90	90 90.03 90
Resolution (Å)	48.59-2.45 (2.54-2.45)*	49.96-2.50 (2.59-2.50)*
R_{merge}	0.046 (0.696)	0.057 (0.386)
R_{means}	0.058 (0.866)	0.071 (0.473)
R_{pim}	0.034 (0.508)	0.041 (0.268)
I / ◎*I*	13.68 (1.41)	11.37 (0.51)
Completeness (%)	95.95 (97.33)	87.8 (83.13)
Redundancy	2.7 (2.7)	2.6 (2.6)
$CC_{1/2}$	0.994 (0.780)	0.993 (0.798)
$CC^{*0.996\ (0.859)}$	0.998 (0.996)	0.998 (0.942)
Wilson B-factor	64.60	64.24
**Refinement**		
Resolution (Å)	2.45	2.50
No. reflections	69661 (7014)	62595 (6523)
Unique reflections	26126 (2635)	24446 (2281)
R_{work} / R_{free}	21.75/27.08	19.73/26.26
No. atoms		
Protein	4631	4717
Ligand/ion	82	50
Water	9	/
B-factors		
Protein	82.80	68.27
Ligand/ion	79.60	65.11
Water	76.34	/
R.m.s. deviations		
Bond lengths (Å)	0.010	0.009
Bond angles (◎)	1.16	1.08
Number of TLS	12	10

*Values in parentheses are for the highest-resolution shell.

Supplementary Table 6. Data collection and refinement statistics (molecular replacement).

Ligand name	**14c1**	**14c3**
(PDB accession code)	9QP0	9QP1
**Data collection**		
Space group	$P2_1$	$P2_1$
Cell dimensions		
a, *b*, *c* (Å)	78.61 64.52 78.61	78.64 64.58 78.66
α, β, γ (°)	90 90.00 90	90 90.02 90
Resolution (Å)	39.30-2.68 (2.77-2.68)*	42.15 -2.65 (2.74-2.65)*
R_{merge}	0.052 (0.611)	0.077 (0.716)
R_{means}	0.075 (0.865)	0.092 (0.849)
R_{pim}	0.052 (0.611)	0.049 (0.452)
I / ◎*I*	8.82 (1.37)	12.81 (1.72)
Completeness (%)	99.73 (99.82)	99.60 (99.96)
Redundancy	1.9 (1.9)	3.3 (3.4)
$CC_{1/2}$	0.996 (0.597)	0.985 (0.663)
$CC^{*0.996\ (0.859)}$	0.999 (0.865)	0.996 (0.893)
Wilson B-factor	55.36	49.00
**Refinement**		
Resolution (Å)	2.68	2.65
No. reflections	42527 (4142)	76802 (7958)
Unique reflections	22319 (2187)	23099 (2315)
R_{work} / R_{free}	20.44/25.70	19.71/25.22
No. atoms		
Protein	4750	4823
Ligand/ion	68	52
Water	11	16
B-factors		
Protein	65.54	54.24
Ligand/ion	66.47	56.38
Water	56.91	49.35
R.m.s. deviations		
Bond lengths (Å)	0.009	0.008
Bond angles (◎)	1.08	1.05
Number of TLS	/	/

*Values in parentheses are for the highest-resolution shell.

Supplementary Table 7. Data collection and refinement statistics (molecular replacement).

Ligand name	**14c4**	**14c5**
(PDB accession code)	9QP2	9QP3
**Data collection**		
Space group	$P2_1$	$P2_1$
Cell dimensions		
a, *b*, *c* (Å)	75.74 64.84 77.23	77.82 64.69 75.90
α, β, γ (°)	90 91.4 90	90 91.7 90
Resolution (Å)	49.25-2.05 (2.12-2.05)*	77.79 -2.00 (2.07-2.00)*
R_{merge}	0.047 (0.643)	0.035 (0.413)
R_{means}	0.067 (0.909)	0.049 (0.584)
R_{pim}	0.047 (0.643)	0.035 (0.413)
I / ◎*I*	8.81 (1.29)	12.46 (2.03)
Completeness (%)	99.48 (97.84)	99.61 (99.45)
Redundancy	1.9 (1.8)	1.9 (1.9)
$CC_{1/2}$	0.997 (0.609)	0.998 (0.733)
$CC^{*0.996\ (0.859)}$	0.999 (0.870)	0.999 (0.920)
Wilson B-factor	39.29	37.33
**Refinement**		
Resolution (Å)	2.05	2.00
No. reflections	88203 (8298)	96060 (9489)
Unique reflections	46945 (4583)	50959 (5054)
R_{work} / R_{free}	20.80/25.20	19.24/22.63
No. atoms		
Protein	4813	4844
Ligand/ion	62	100
Water	115	235
B-factors		
Protein	48.88	44.10
Ligand/ion	48.45	52.14
Water	43.93	43.38
R.m.s. deviations		
Bond lengths (Å)	0.008	0.008
Bond angles (◎)	0.89	0.93
Number of TLS	9	14

*Values in parentheses are for the highest-resolution shell.

Supplementary Table 8. Data collection and refinement statistics (molecular replacement).

Ligand name	**14c6**	**14b1**
(PDB accession code)	9QP5	9QP6
**Data collection**		
Space group	$P2_1$	$P2_1$
Cell dimensions		
a, *b*, *c* (Å)	77.60 65.25 76.41	75.78 64.97 77.75
α, β, γ (°)	90 91.6 90	90 91.5 90
Resolution (Å)	42.15-2.35 (2.43-2.35)*	53.53-2.35 (2.43-2.35)*
R_{merge}	0.084 (0.775)	0.102 (0.684)
R_{means}	0.100 (0.915)	0.124 (0.817)
R_{pim}	0.054 (0.482)	0.068 (0.438)
I / ◎*I*	10.96 (1.53)	8.35 (0.80)
Completeness (%)	99.52 (99.84)	99.59 (98.52)
Redundancy	3.4 (3.5)	3.3 (3.5)
$CC_{1/2}$	0.996 (0.704)	0.965 (0.702)
$CC^{*0.996\ (0.859)}$	0.999 (0.909)	0.991 (0.908)
Wilson B-factor	45.92	45.83
**Refinement**		
Resolution (Å)	2.35	2.35
No. reflections	107522 (11217)	103691 (10978)
Unique reflections	31915 (3164)	31686 (3128)
R_{work} / R_{free}	21.26/27.20	21.39/27.57
No. atoms		
Protein	4830	4805
Ligand/ion	72	62
Water	43	54
B-factors		
Protein	57.11	51.45
Ligand/ion	56.45	50.65
Water	52.04	43.94
R.m.s. deviations		
Bond lengths (Å)	0.009	0.009
Bond angles (◎)	1.01	1.00
Number of TLS	11	8

*Values in parentheses are for the highest-resolution shell.

Supplementary Table 9. Data collection and refinement statistics (molecular replacement).

Ligand name	**14m**	**14n**
(PDB accession code)	9QP7	9QPA
**Data collection**		
Space group	$P2_1$	$P2_1$
Cell dimensions		
a, *b*, *c* (Å)	77.52 64.85 75.70	77.42 64.31 75.48
α, β, γ (°)	90 91.2 90	90 91.7 90
Resolution (Å)	41.31-2.50 (2.59-2.50)*	41.73-2.50 (2.59-2.50)*
R_{merge}	0.059 (0.762)	0.135 (0.961)
R_{means}	0.070 (0.902)	0.160 (1.134)
R_{pim}	0.038 (0.658)	0.086 (0.597)
I / ◎*I*	14.61 (1.58)	7.29 (1.13)
Completeness (%)	99.54 (99.89)	99.48 (99.81)
Redundancy	3.3 (3.5)	3.3 (3.5)
$CC_{1/2}$	0.998 (0.658)	0.990 (0.612)
$CC^{*0.996\ (0.859)}$	1.000 (0.891)	0.998 (0.871)
Wilson B-factor	58.72	46.33
**Refinement**		
Resolution (Å)	2.50	2.50
No. reflections	87279 (9145)	86324 (8986)
Unique reflections	26104 (2609)	25776 (2563)
R_{work} / R_{free}	20.63/25.50	21.55/27.58
No. atoms		
Protein	4770	4859
Ligand/ion	64	84
Water	20	33
B-factors		
Protein	66.90	52.14
Ligand/ion	65.32	52.00
Water	62.24	47.53
R.m.s. deviations		
Bond lengths (Å)	0.008	0.009
Bond angles (◎)	0.93	1.06
Number of TLS	8	9

*Values in parentheses are for the highest-resolution shell.

Supplementary Table 10. Data collection and refinement statistics (molecular replacement).

Ligand entry	**1**	**23**
(PDB accession code)	9QQJ	9RL1
**Data collection**		
Space group	$P2_1$	$P2_1$
Cell dimensions		
a, b, c (Å)	48.73 70.23 60.29	79.20 63.72 79.13
α, β, γ (°)	90 109.1 90	90 89.9 90
Resolution (Å)	43.43-1.69 (1.90-1.69)*	79.21-2.30 (2.38-2.30)*
R_{merge}	0.032 (0.467)	0.116 (0.741)
R_{means}	0.039 (0.559)	0.142 (0.877)
R_{pim}	0.021 (0.304)	0.079 (0.465)
I / ◎*I*	17.98 (2.00)	7.47 (0.56)
Completeness (%)	98.95 (98.07)	99.33 (95.48)
Redundancy	3.2 (3.2)	3.3 (3.5)
$CC_{1/2}$	0.999 (0.844)	0.990 (0.715)
CC^*	1.000 (0.957)	0.994 (0.914)
Wilson B-factor	28.63	47.43
**Refinement**		
Resolution (Å)	1.69	2.30
No. reflections	134915 (13221)	117086 (12220)
Unique reflections	42306 (4175)	35237 (3335)
R_{work} / R_{free}	18.56/22.17	21.07/26.39
No. atoms		
Protein	2815	4829
Ligand/ion	28	56
Water	241	23
B-factors		
Protein	37.05	56.12
Ligand/ion	47.00	51.89
Water	38.75	50.85
R.m.s. deviations		
Bond lengths (Å)	0.007	0.009
Bond angles (◎)	0.90	1.06
Number of TLS	4	10

*Values in parentheses are for the highest-resolution shell.

NMR spectra

1D NMR spectra are sorted in the order ^{1}H, ^{13}C.

Compound 10

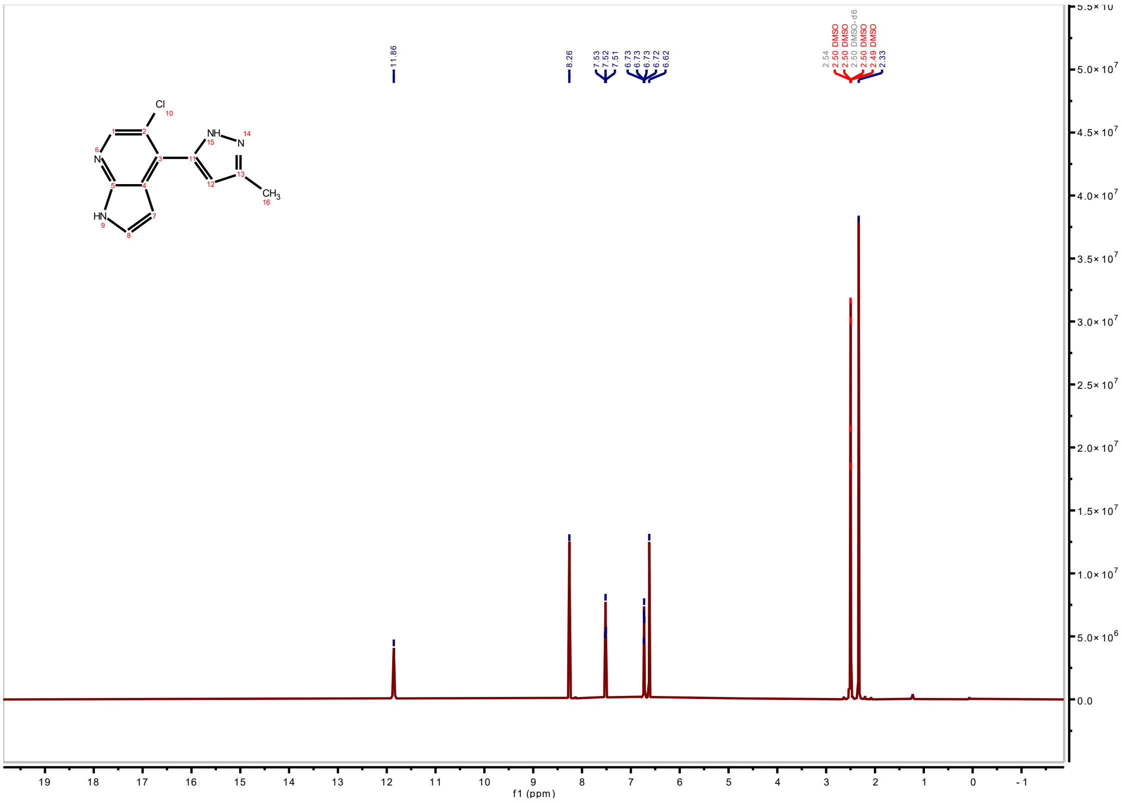

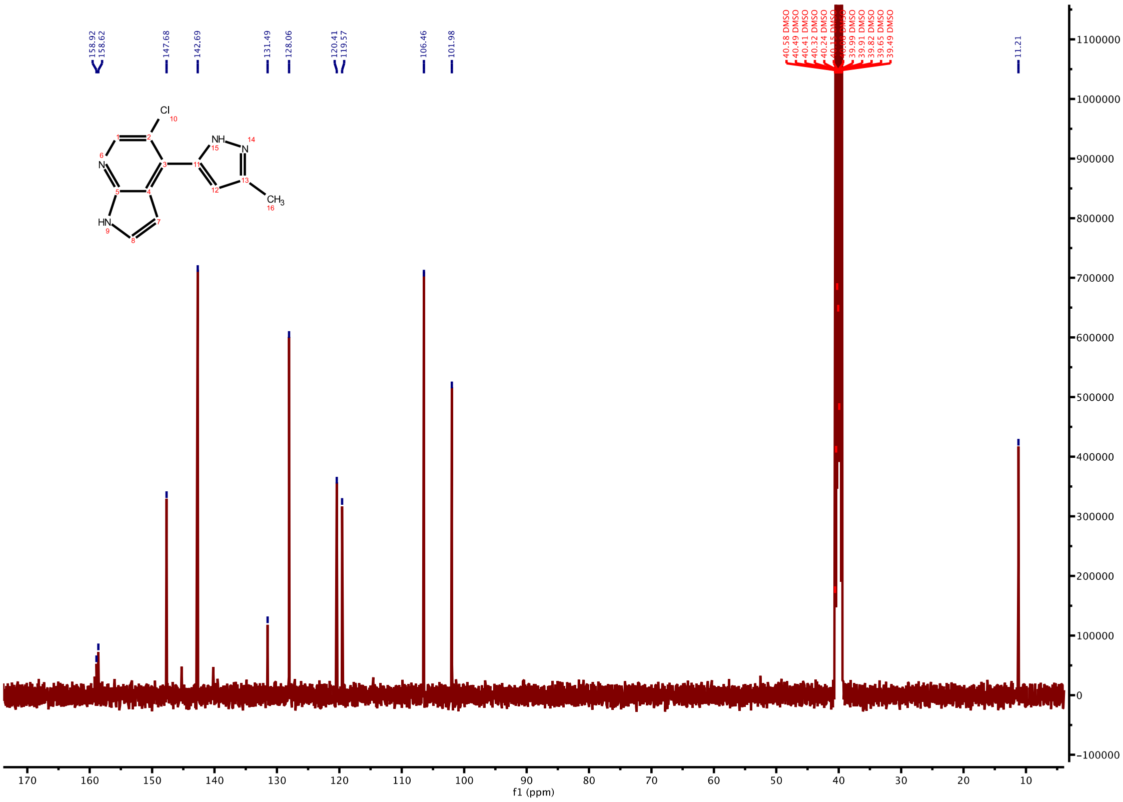

Compound 14a

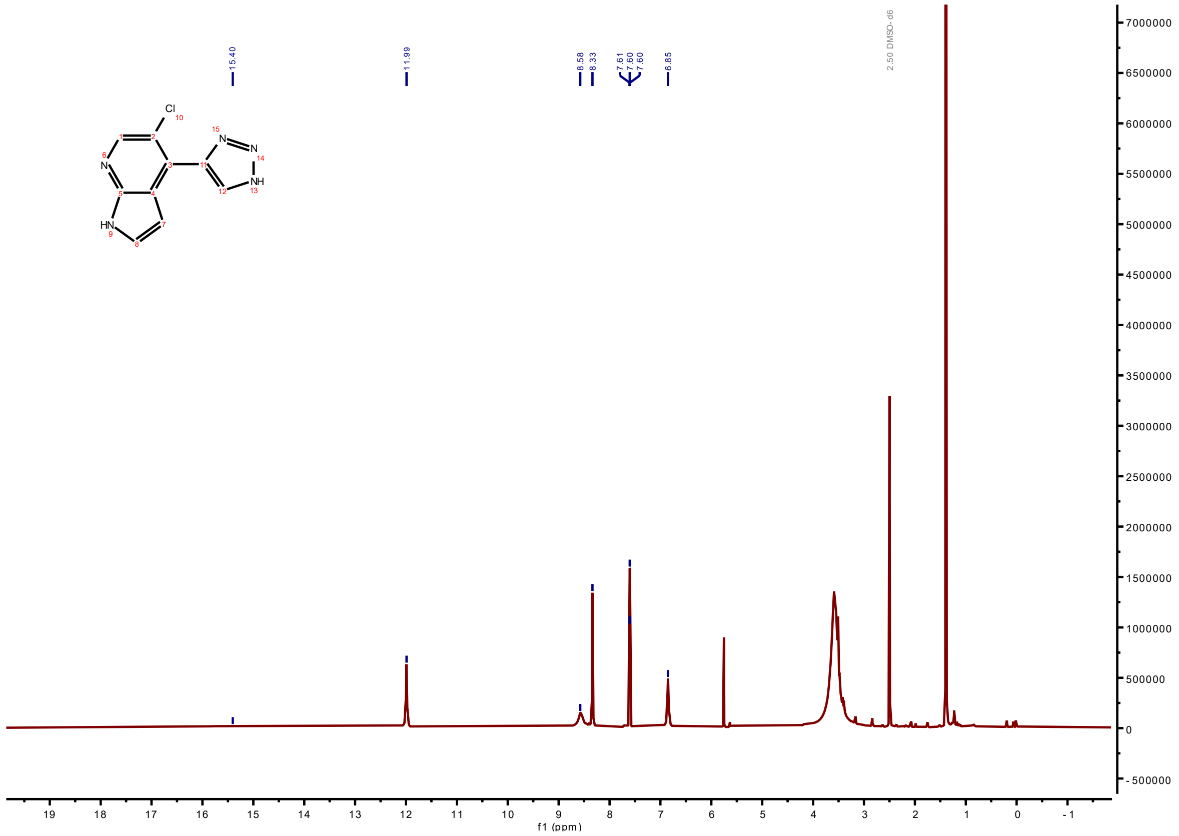

Compound 14b

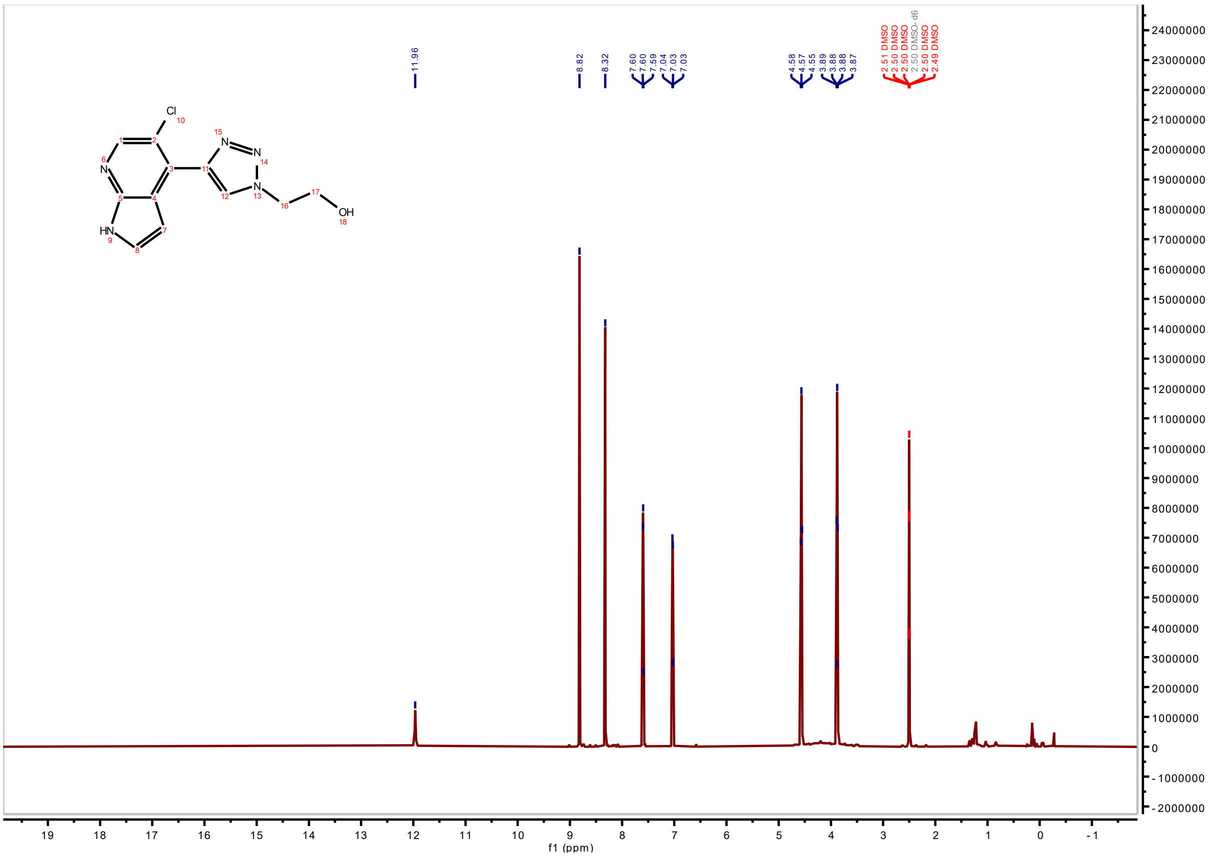

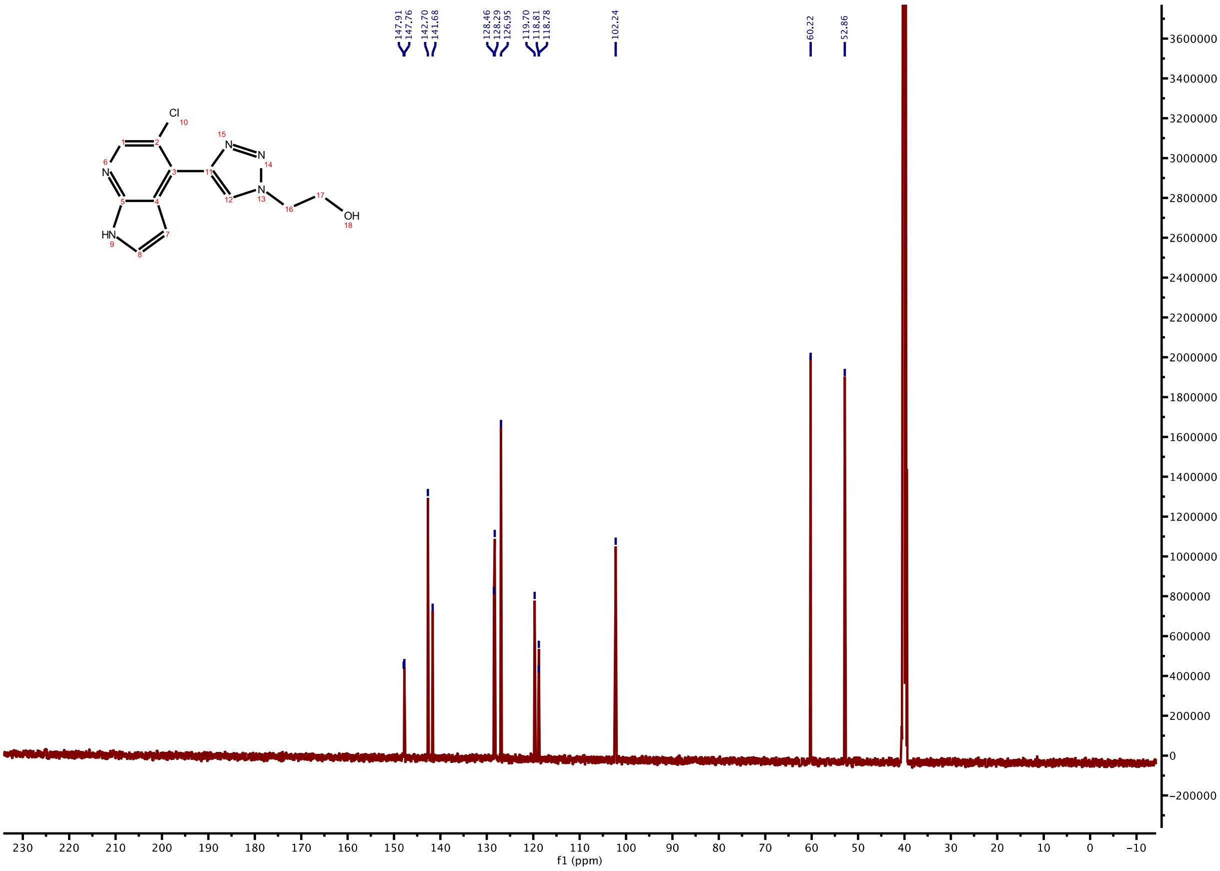

Compound 14c

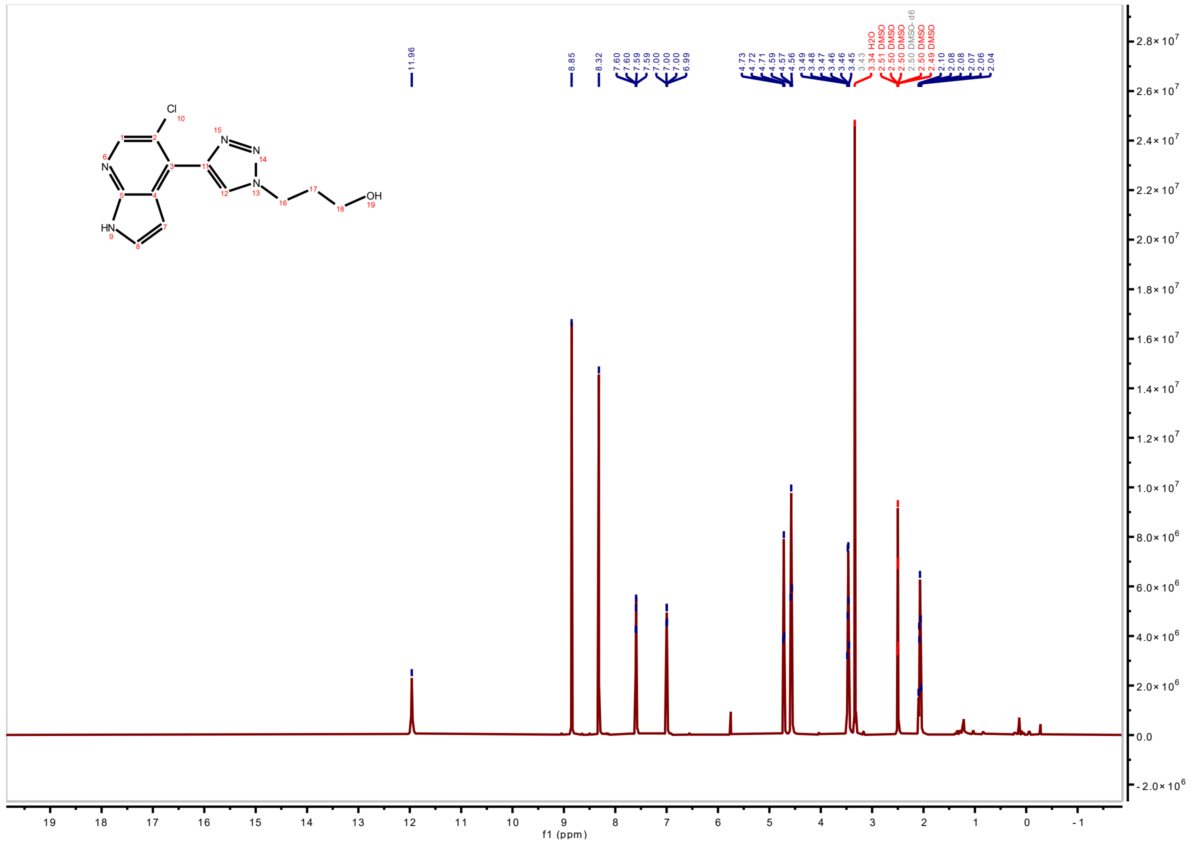

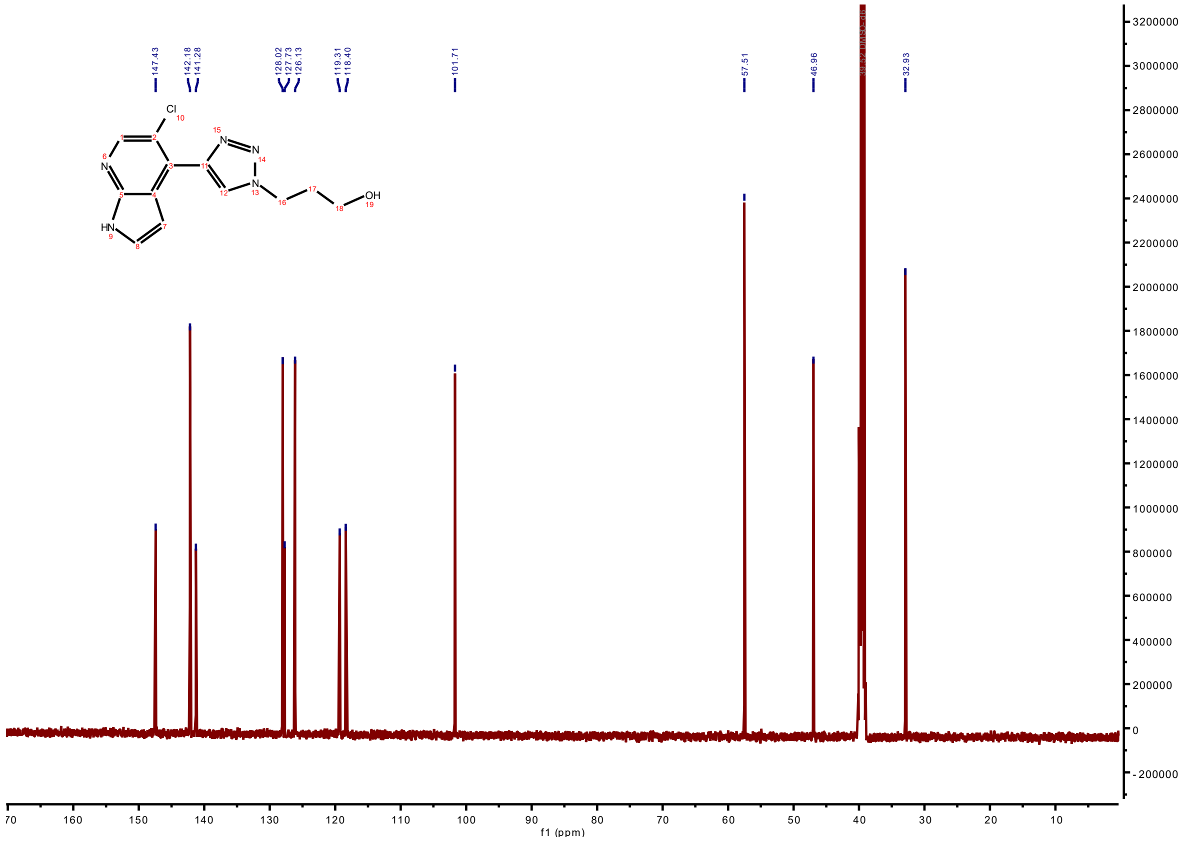

Compound 14d

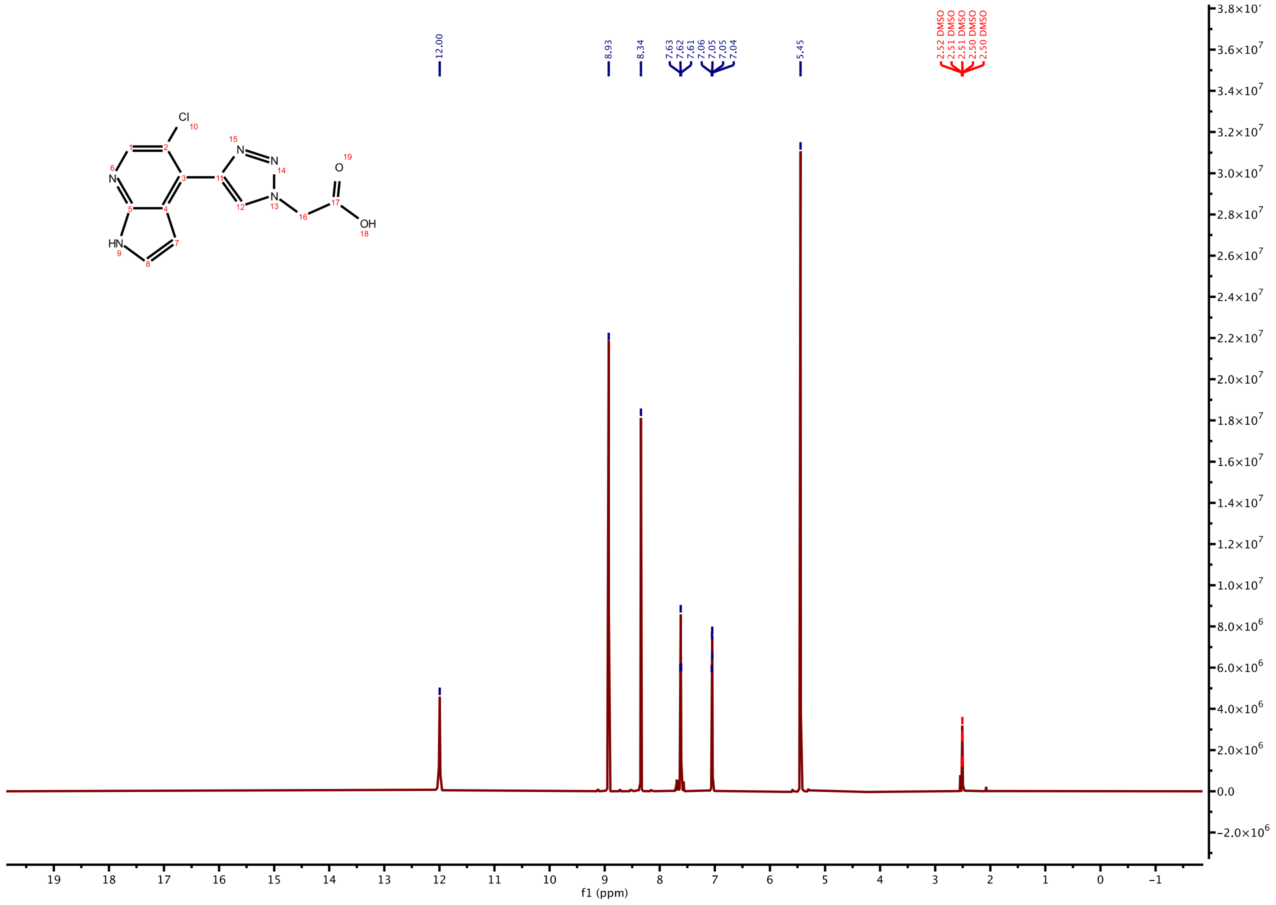

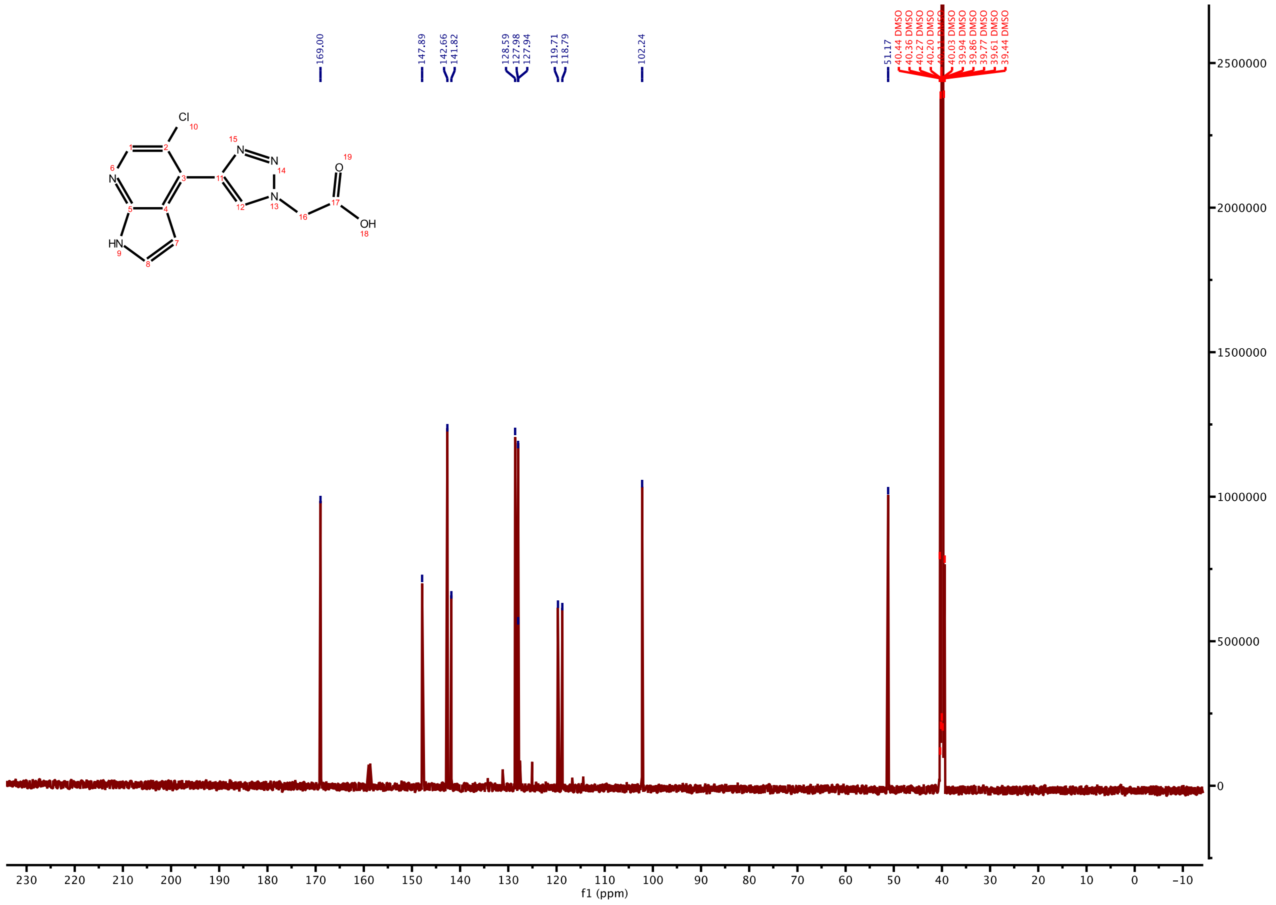

Compound 14e

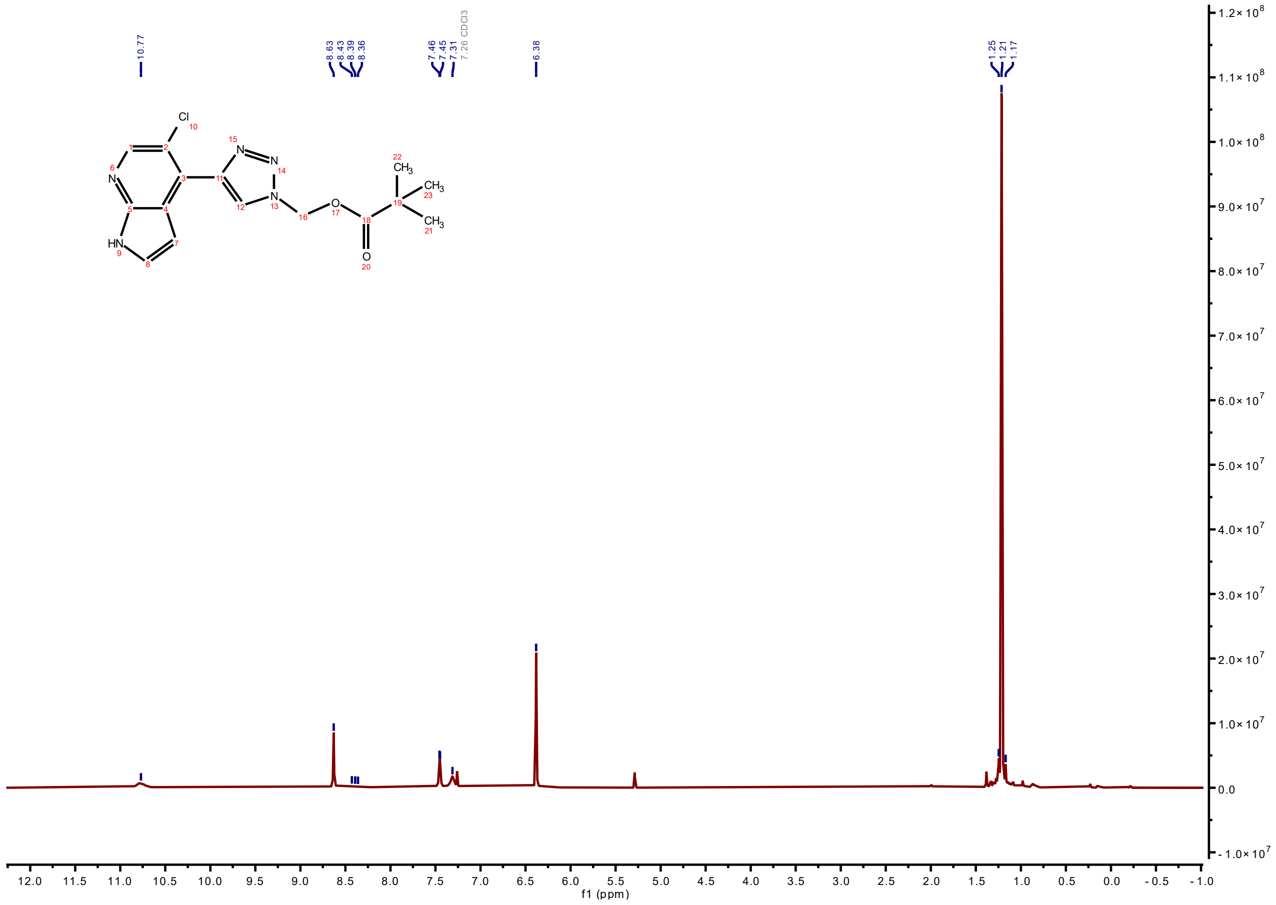

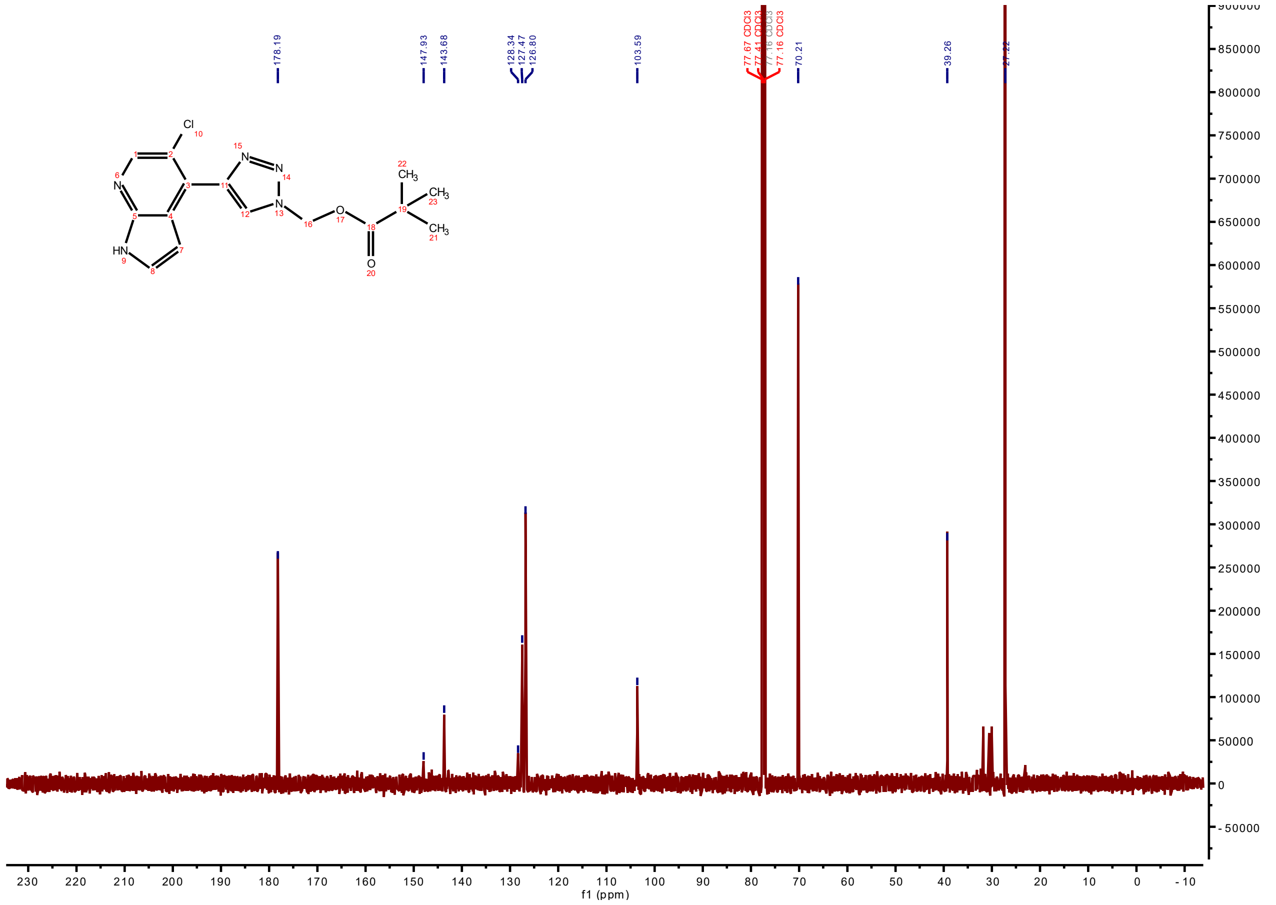

Compound 14f

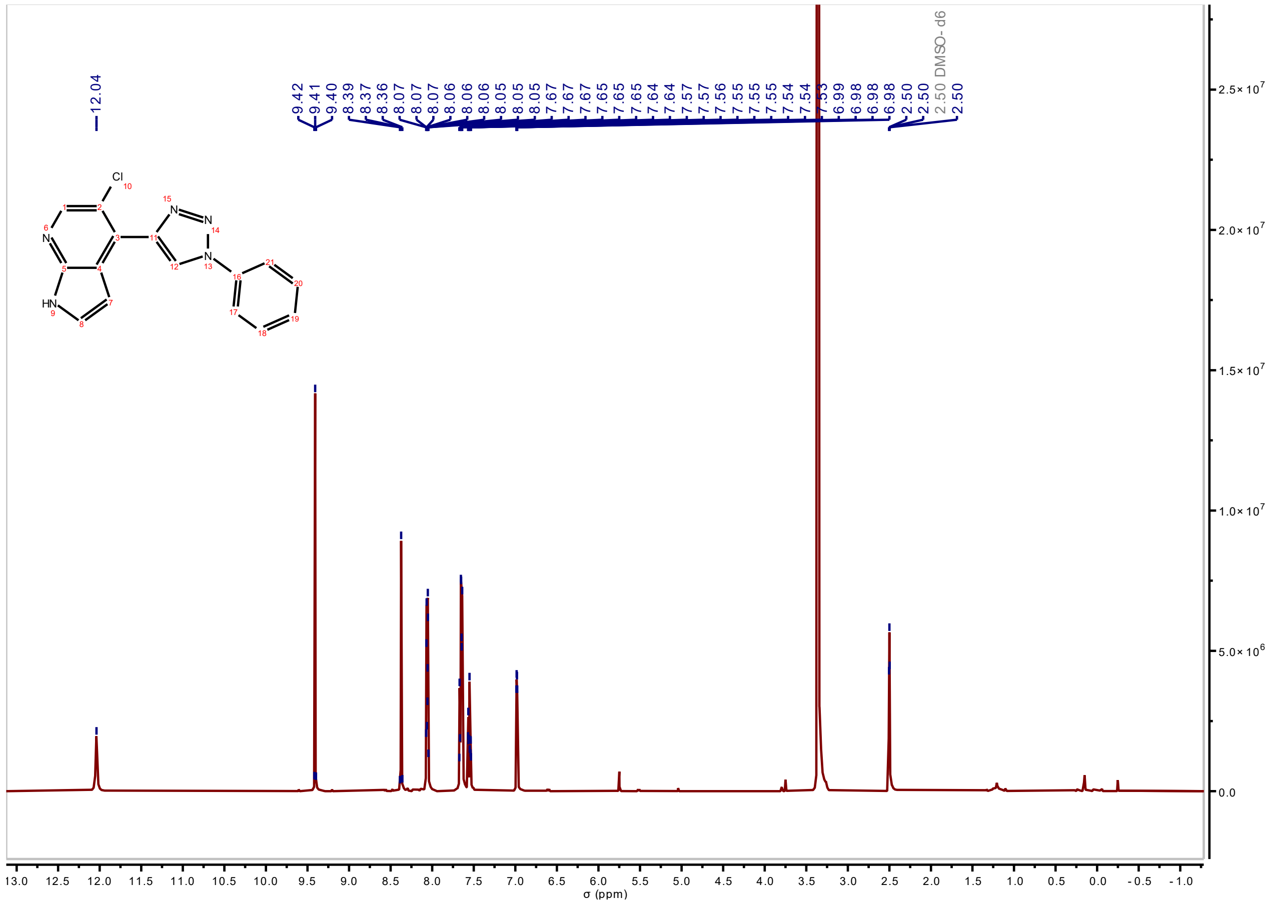

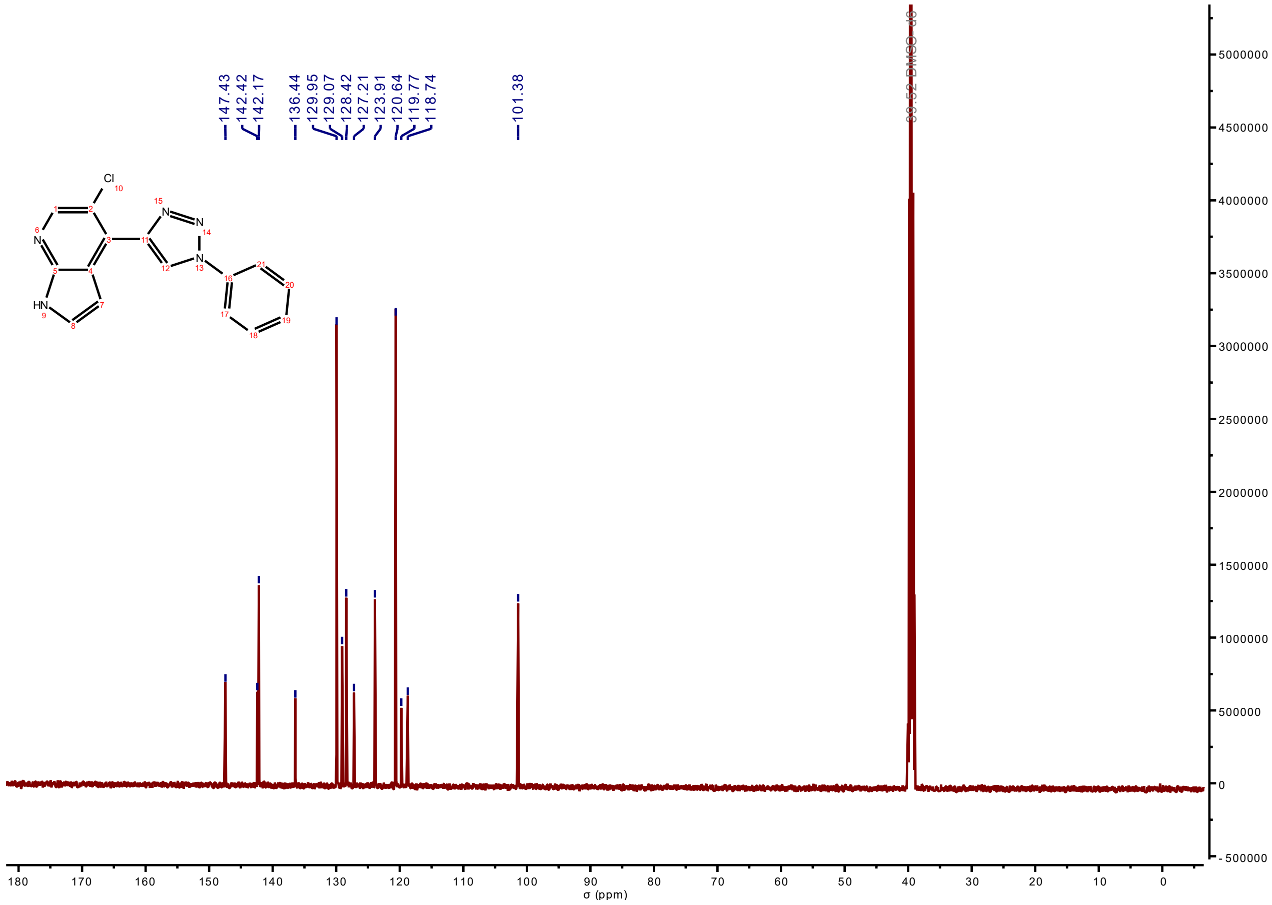

Compound 14g

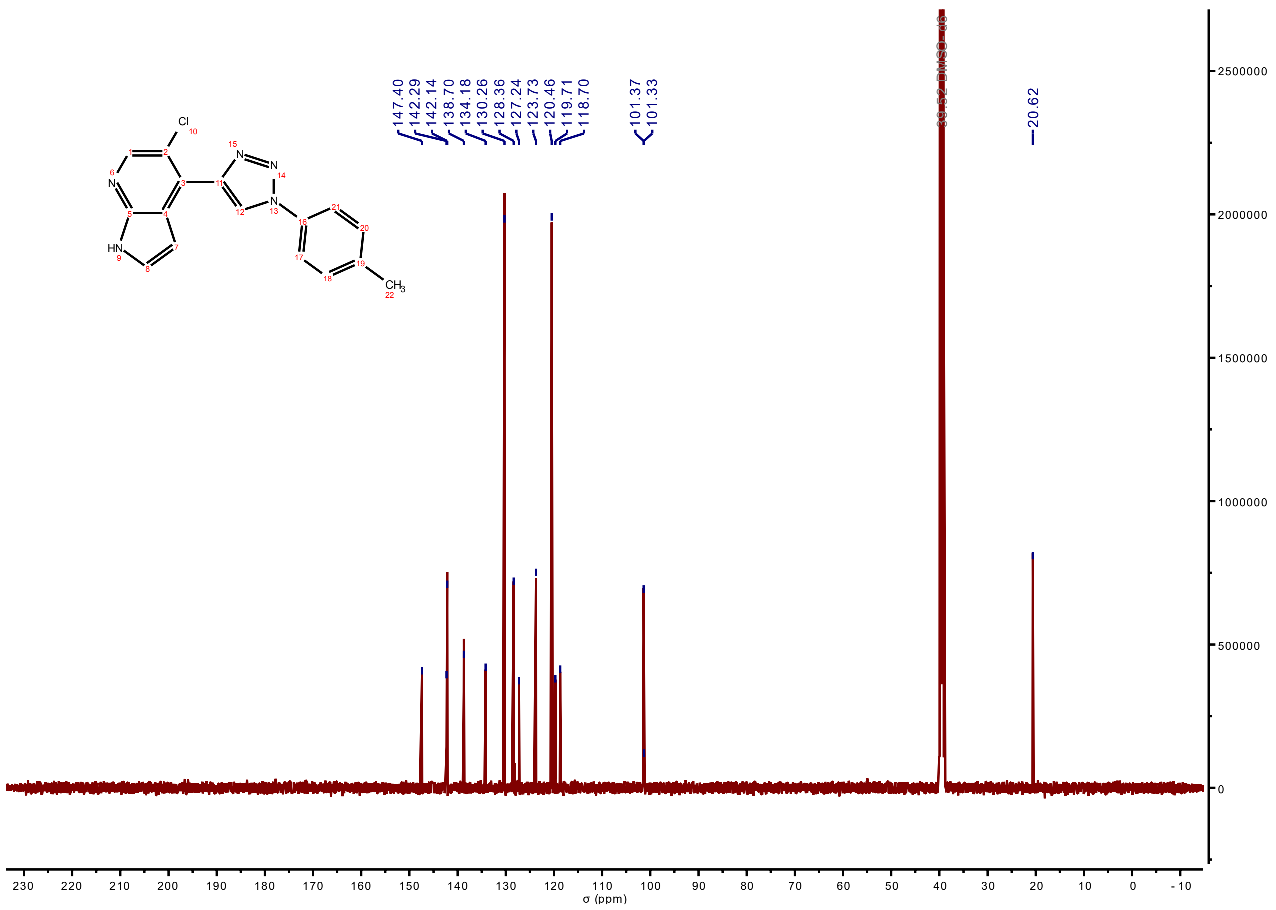

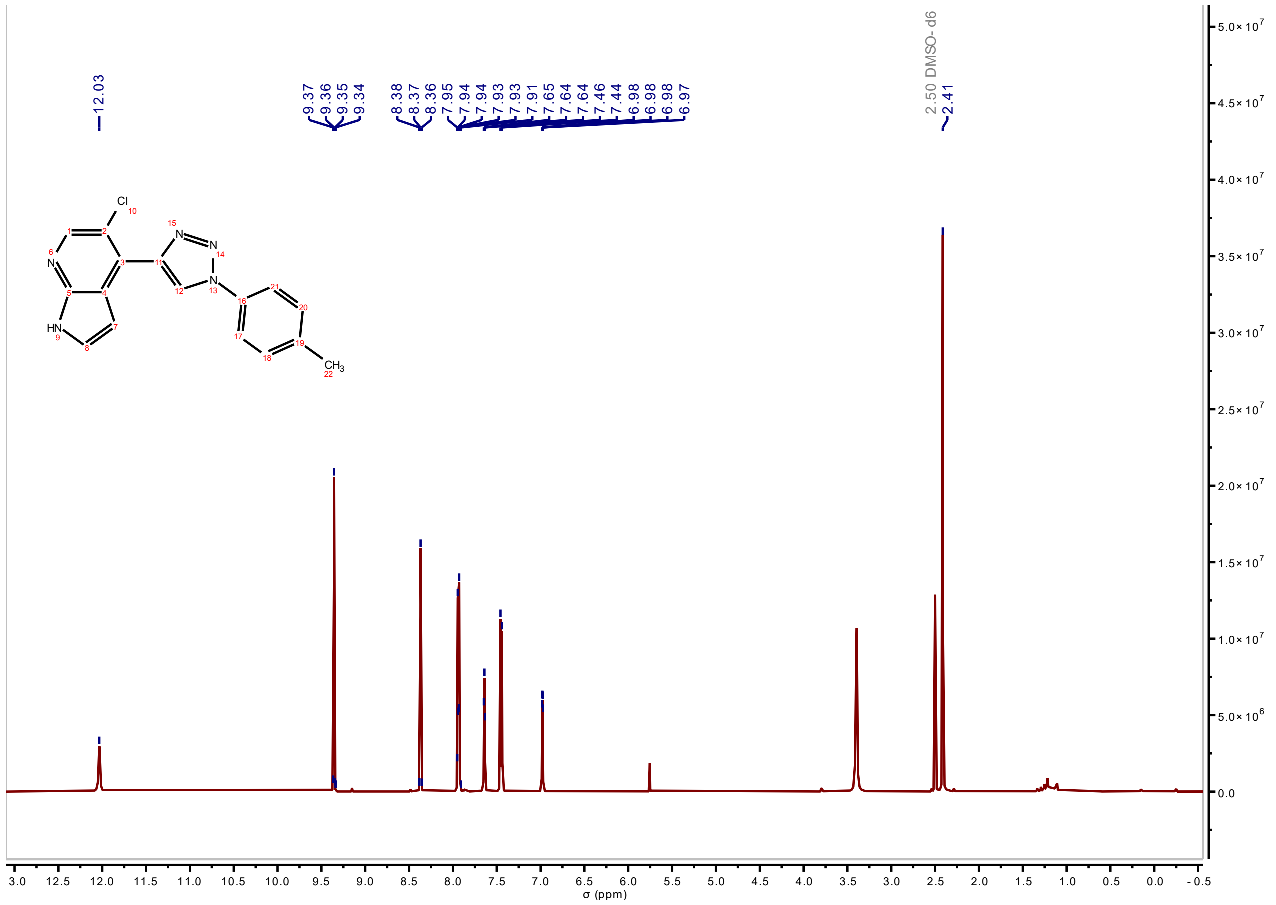

Compound 14h

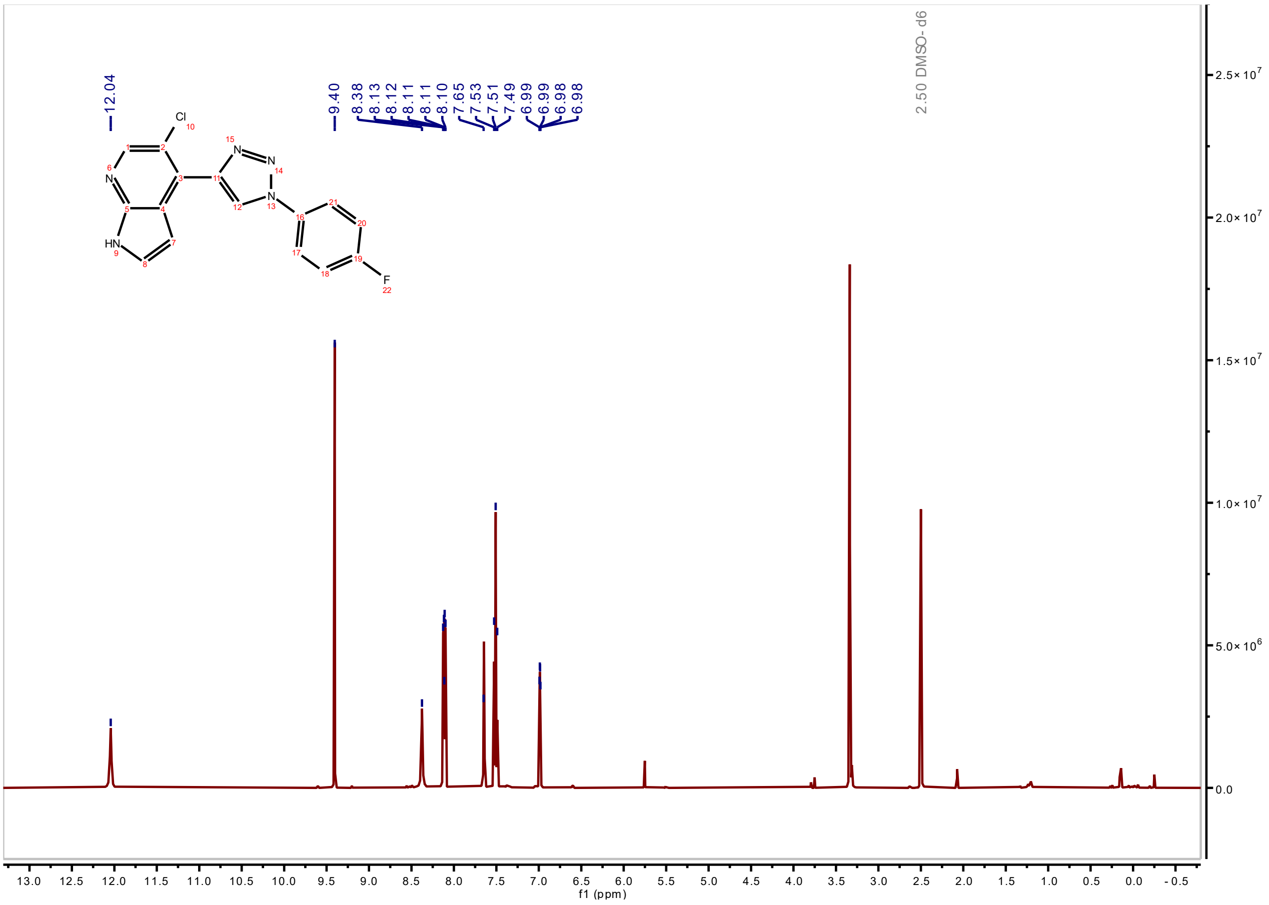

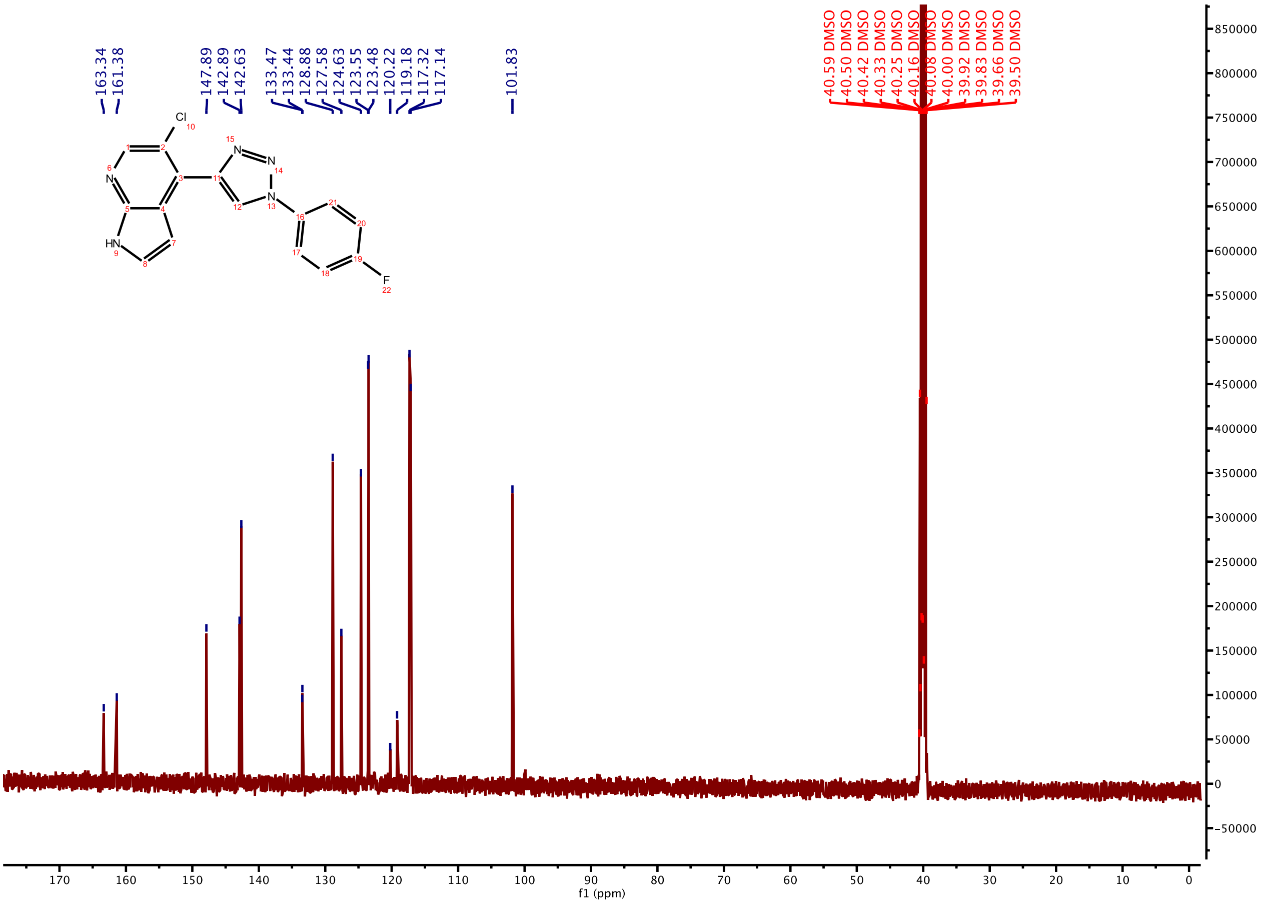

Compound 14i

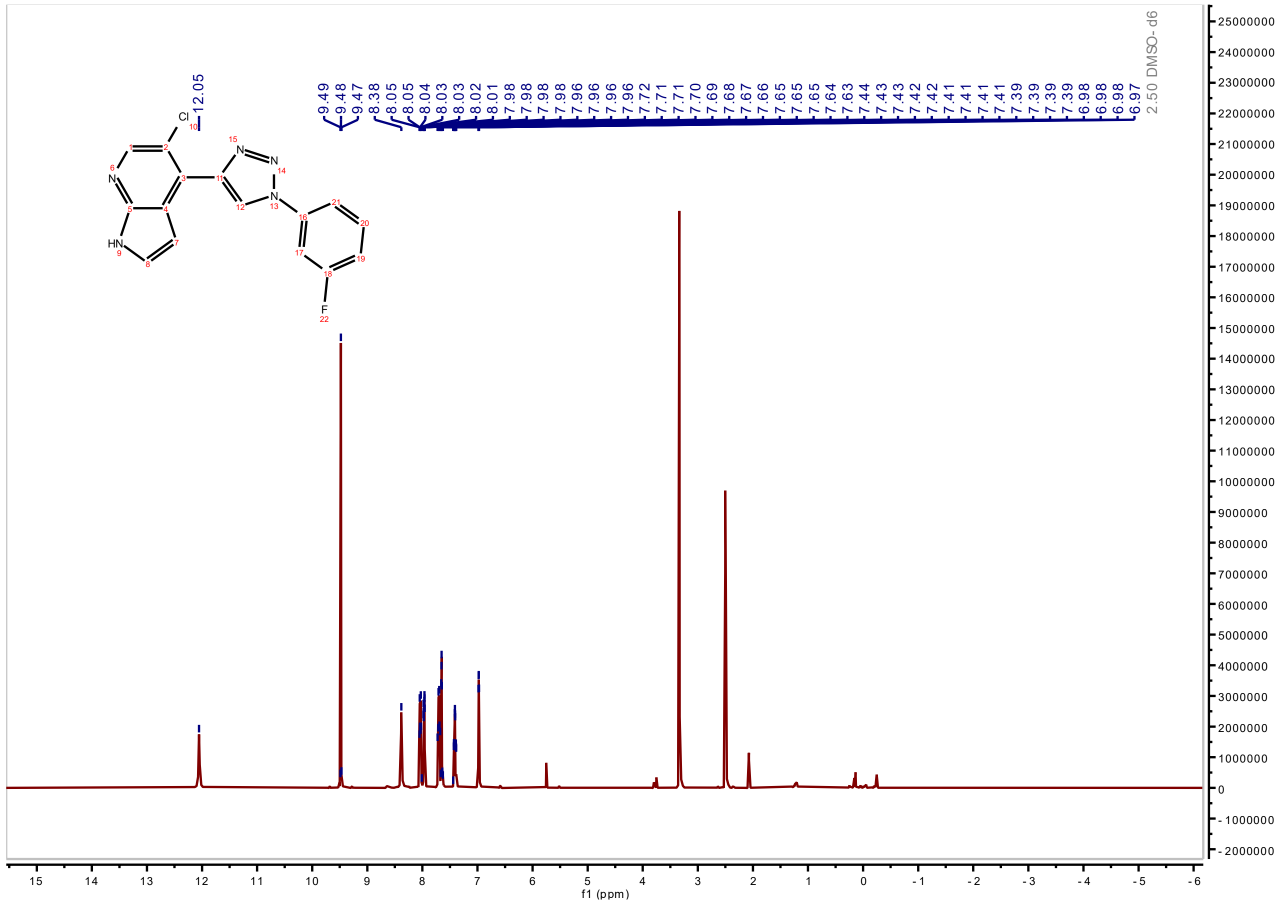

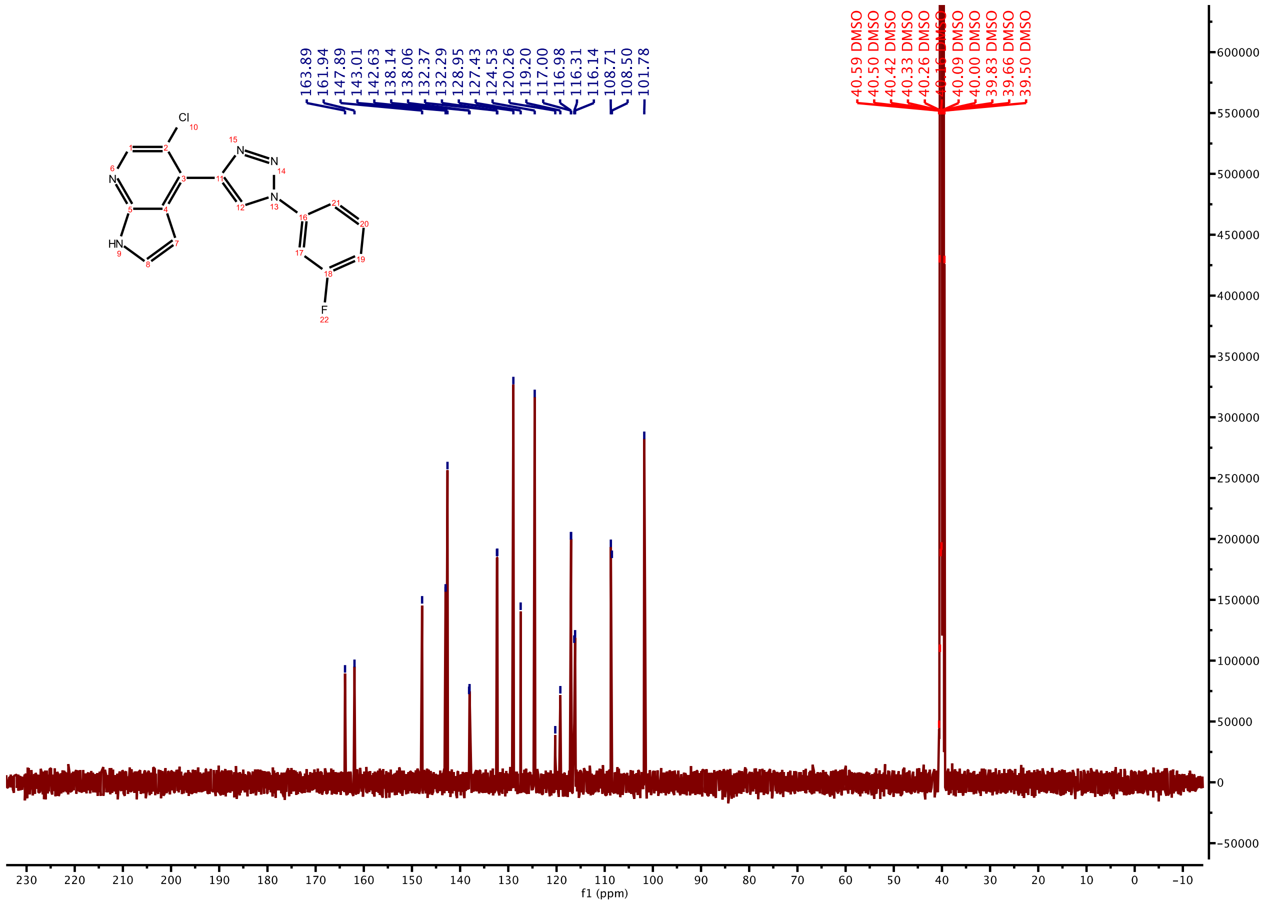

Compound 14j

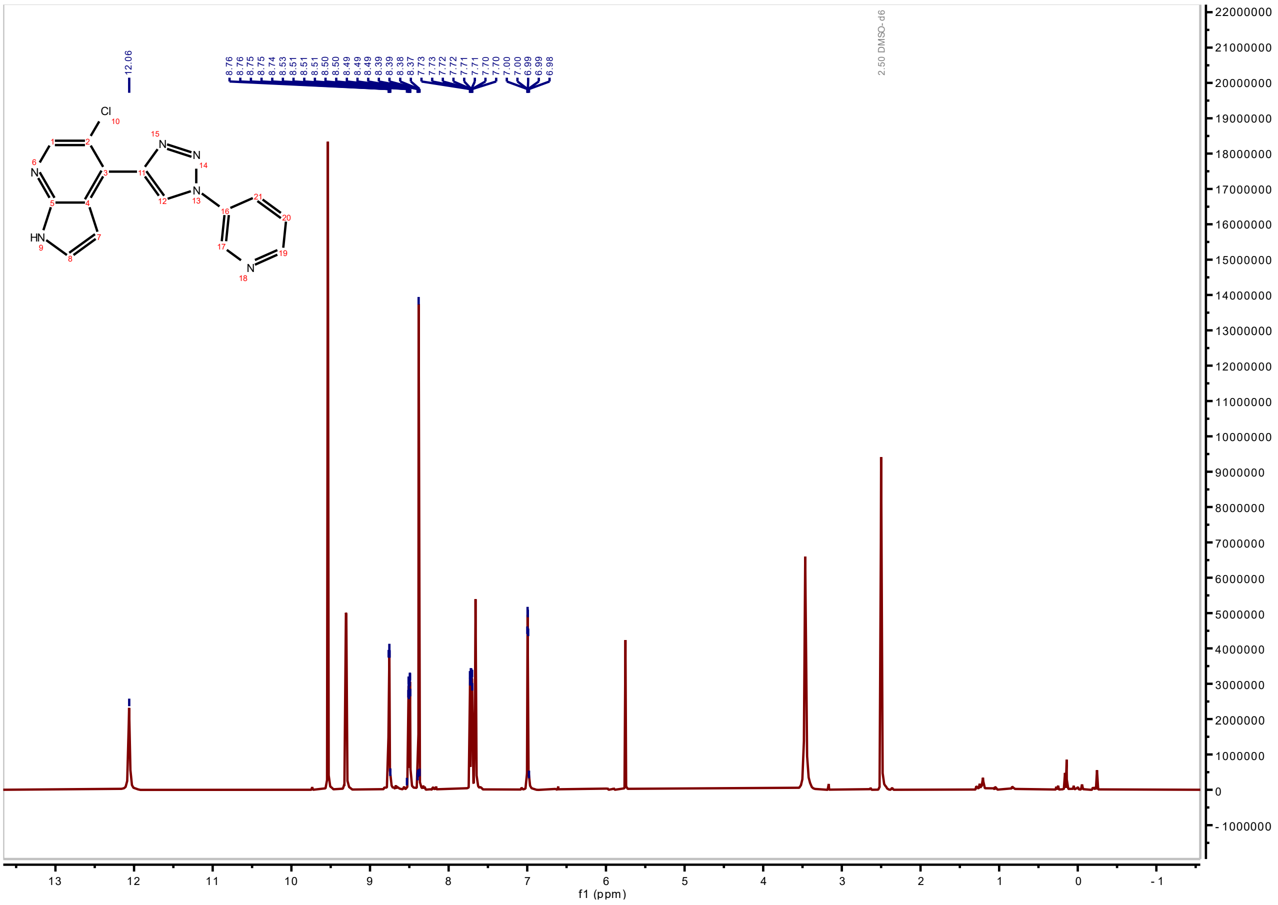

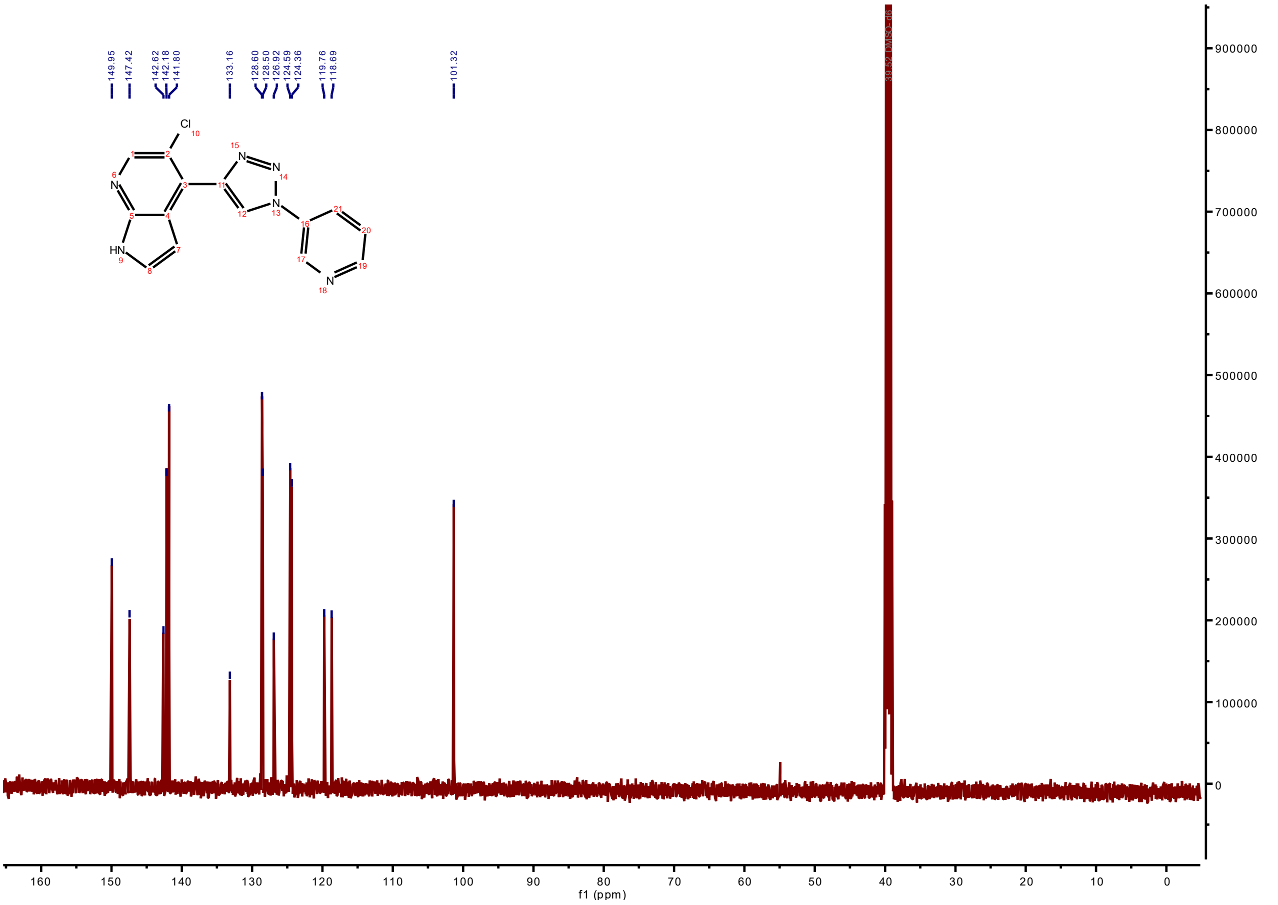

Compound 14k

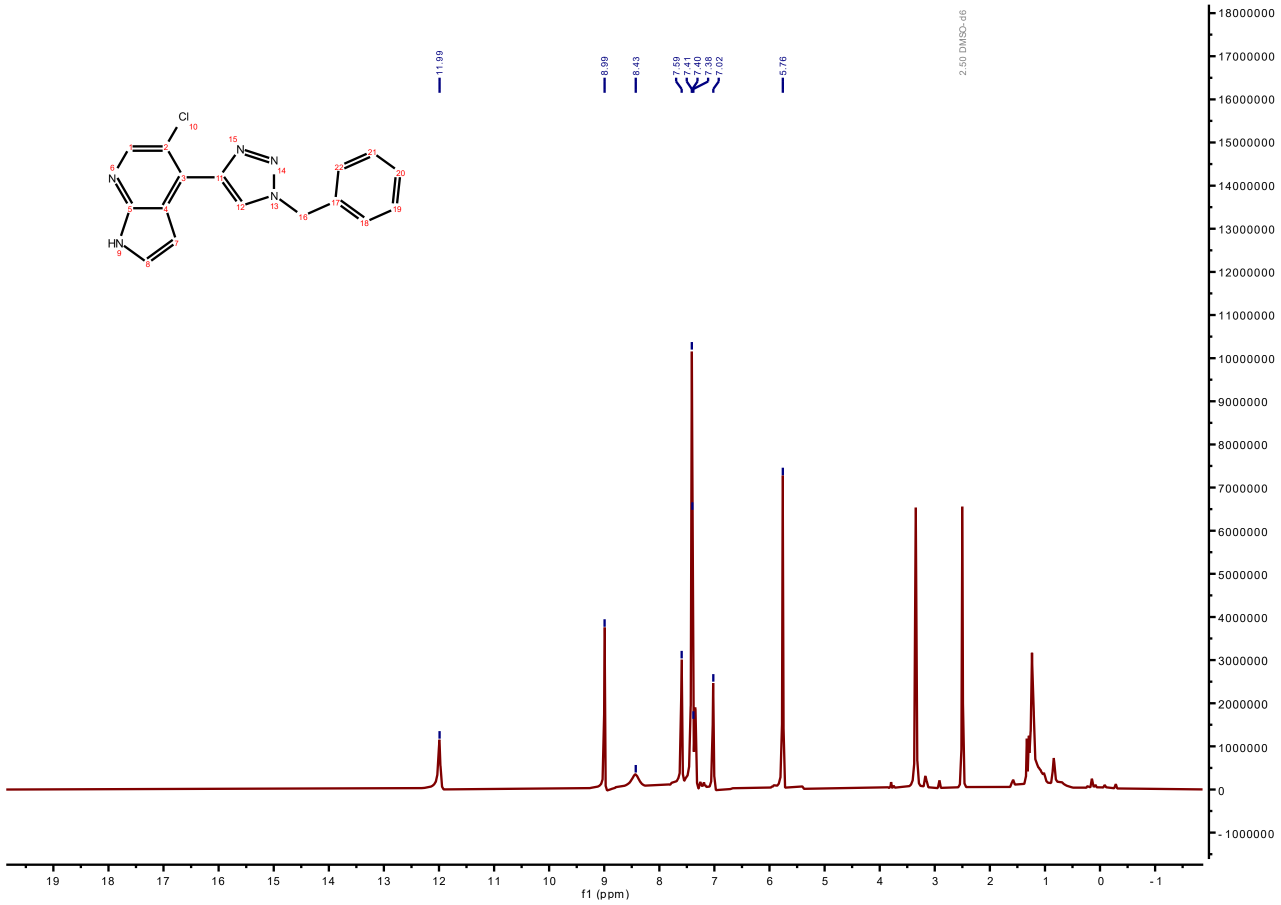

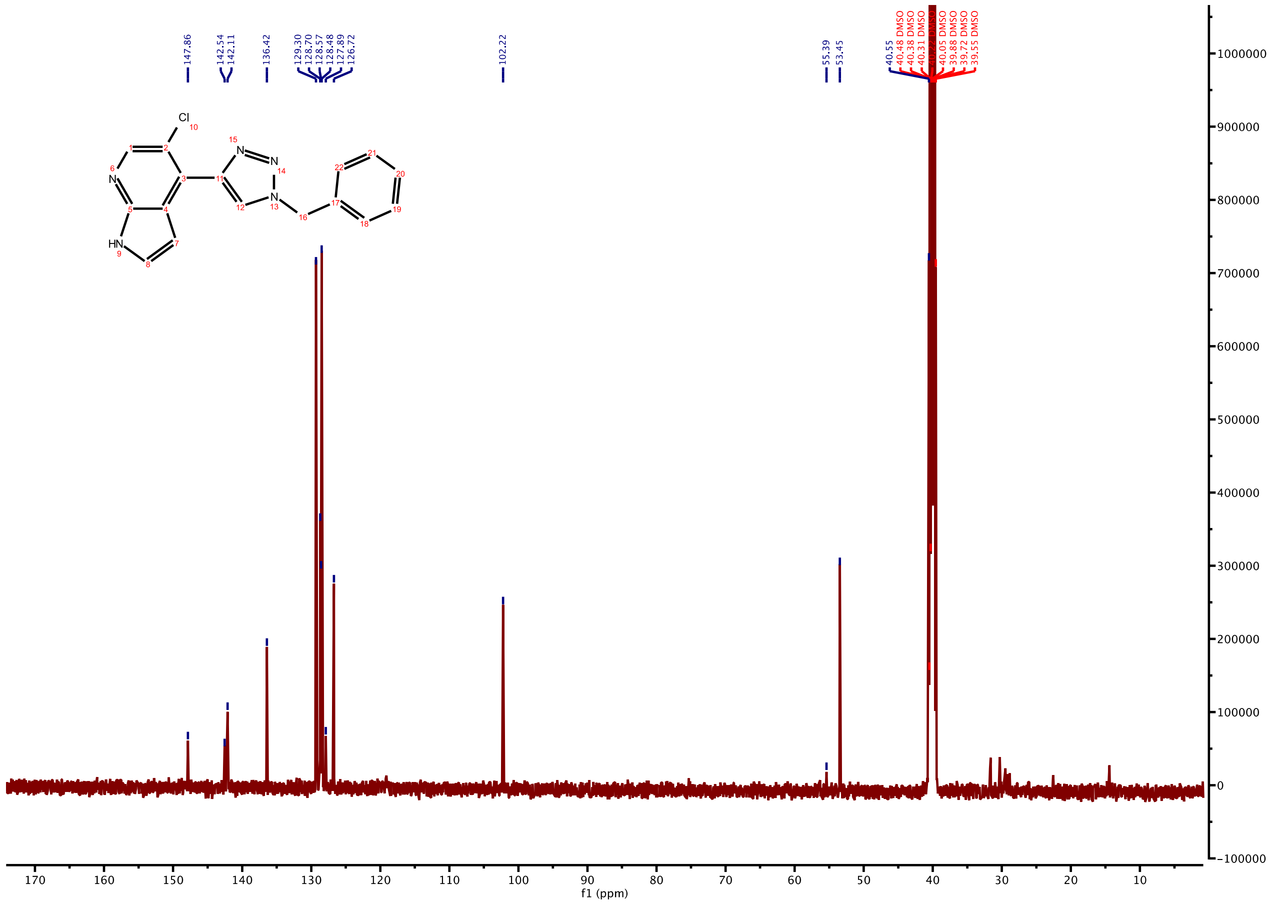

Compound 14l

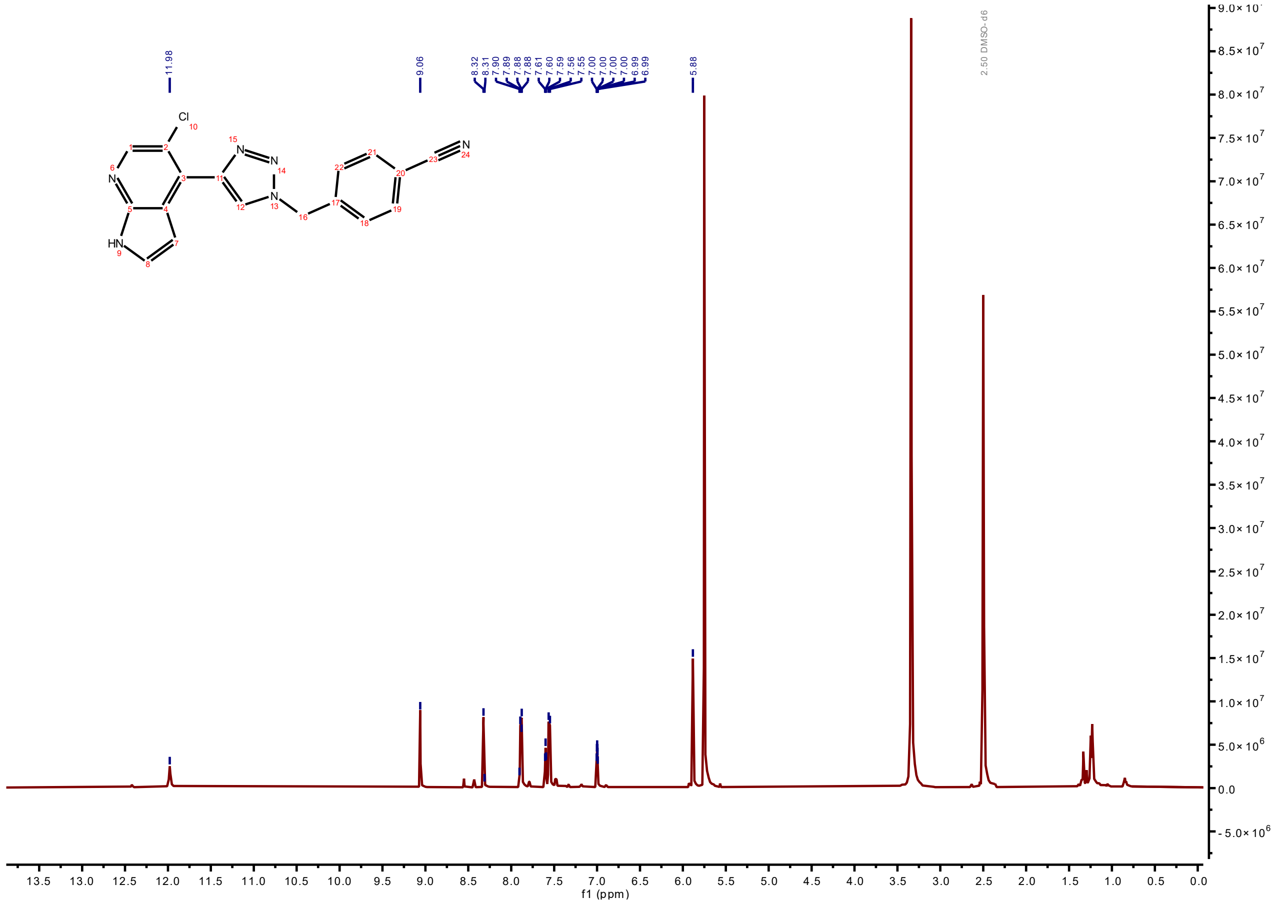

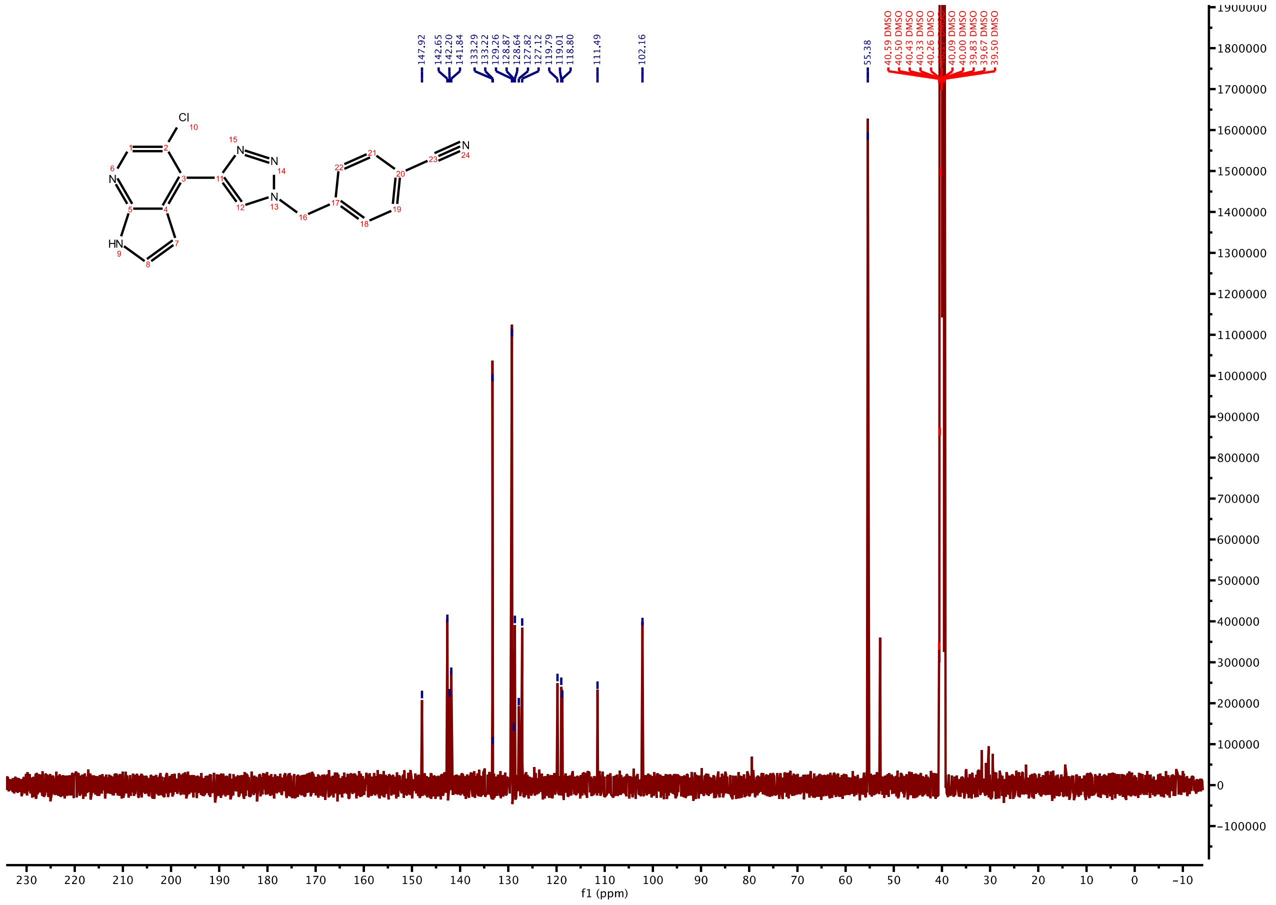

Compound 23

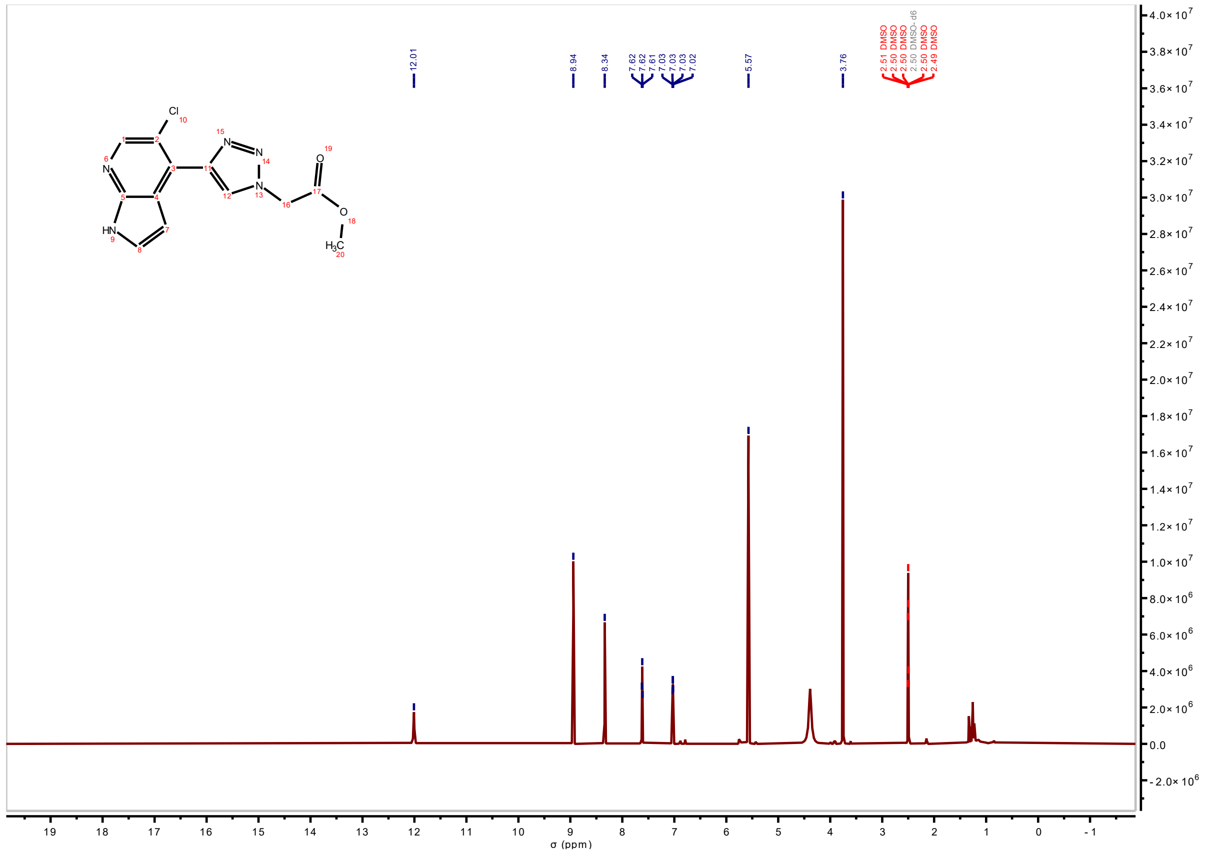
12.01
8.94
8.34
7.62
7.62
7.61
7.03
7.03
7.03
7.02
5.57
3.76
2.51 DMSO
2.50 DMSO
2.50 DMSO
2.50 DMSO- d6
2.50 DMSO
2.50 DMSO
2.49 DMSO
σ (ppm)

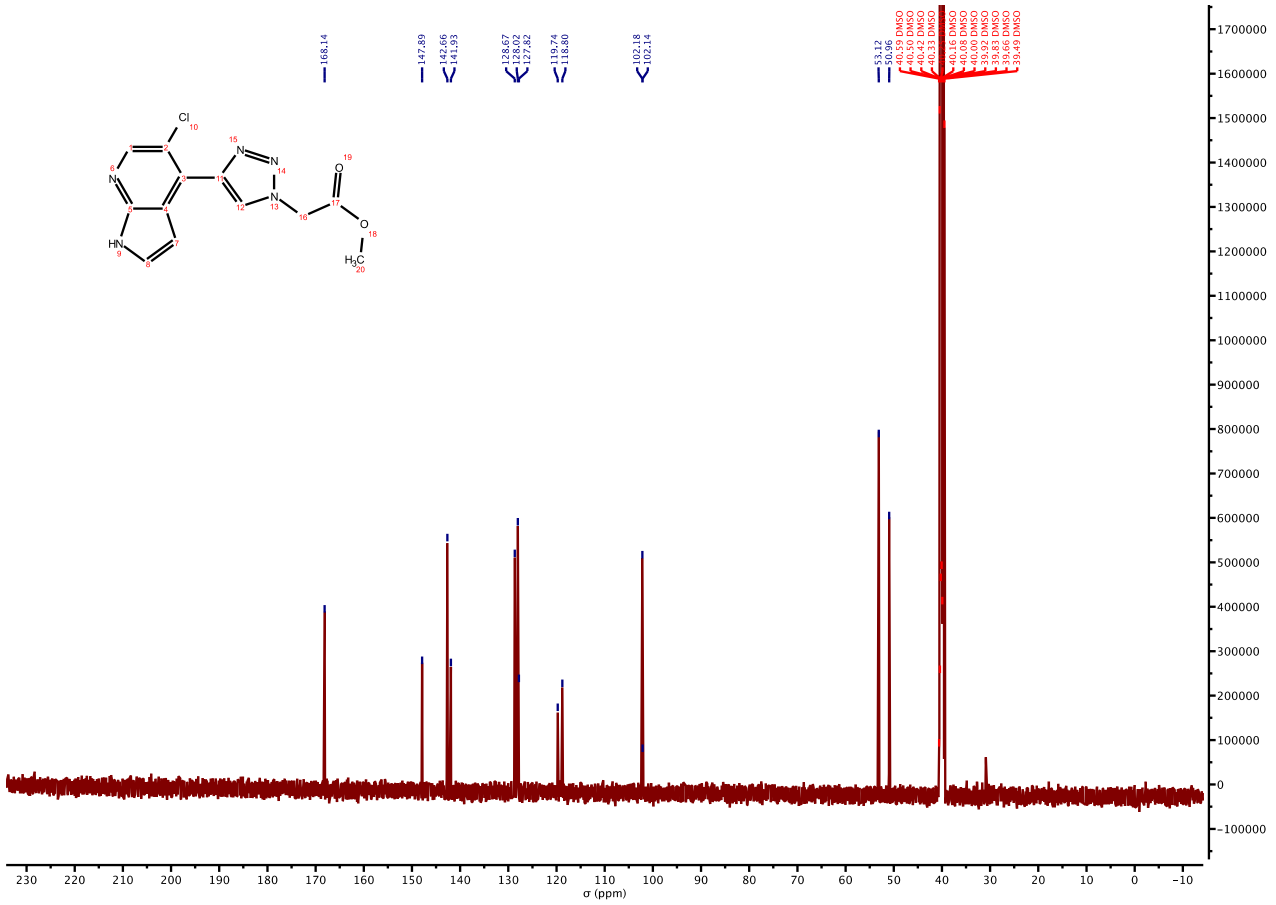
168.14
147.89
142.66
141.93
128.67
128.02
127.82
119.74
118.80
102.18
102.14
53.12
50.96
40.59 DMSO
40.50 DMSO
40.42 DMSO
40.33 DMSO
40.16 DMSO
40.08 DMSO
40.00 DMSO
39.92 DMSO
39.83 DMSO
39.66 DMSO
39.49 DMSO
σ (ppm)

Compound 24

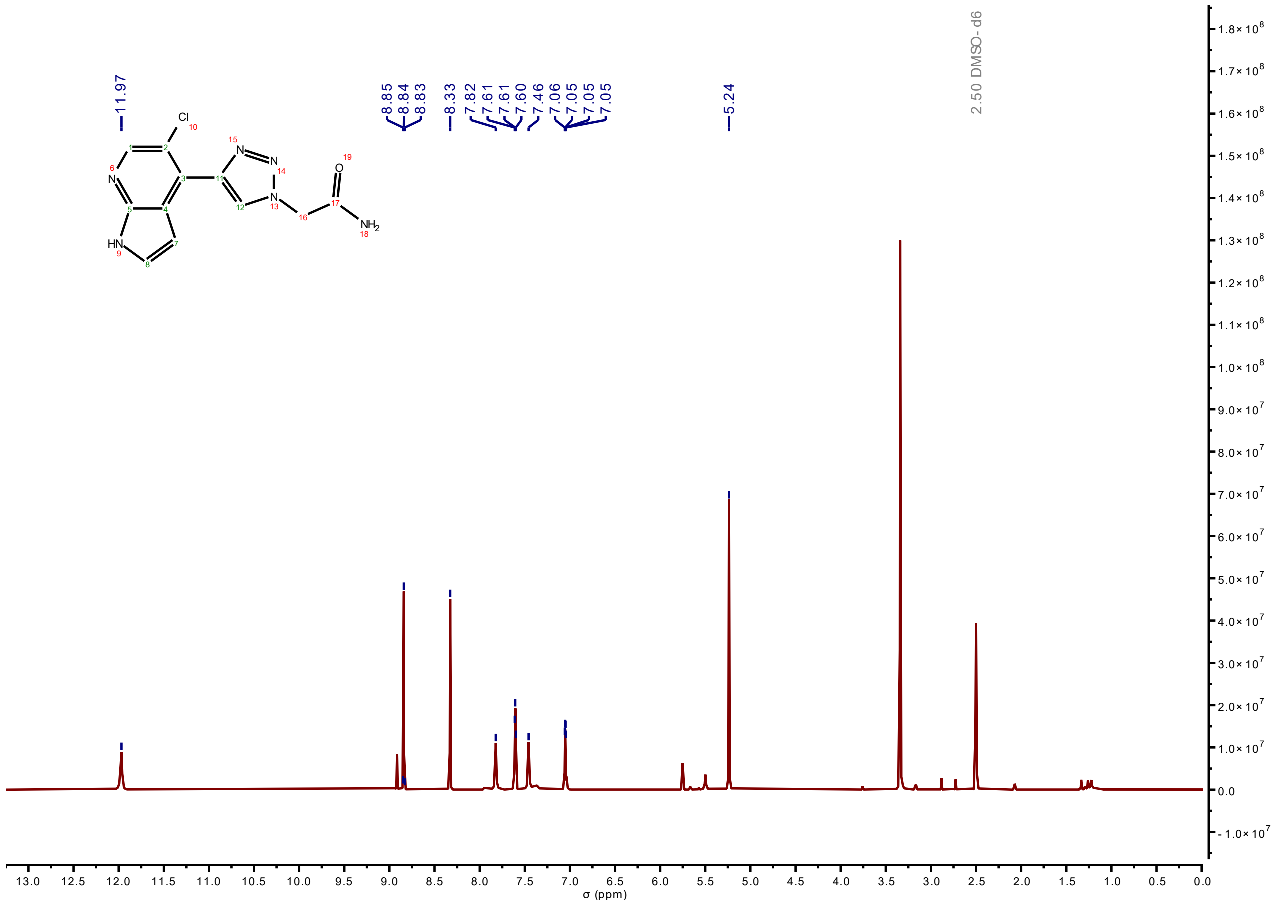

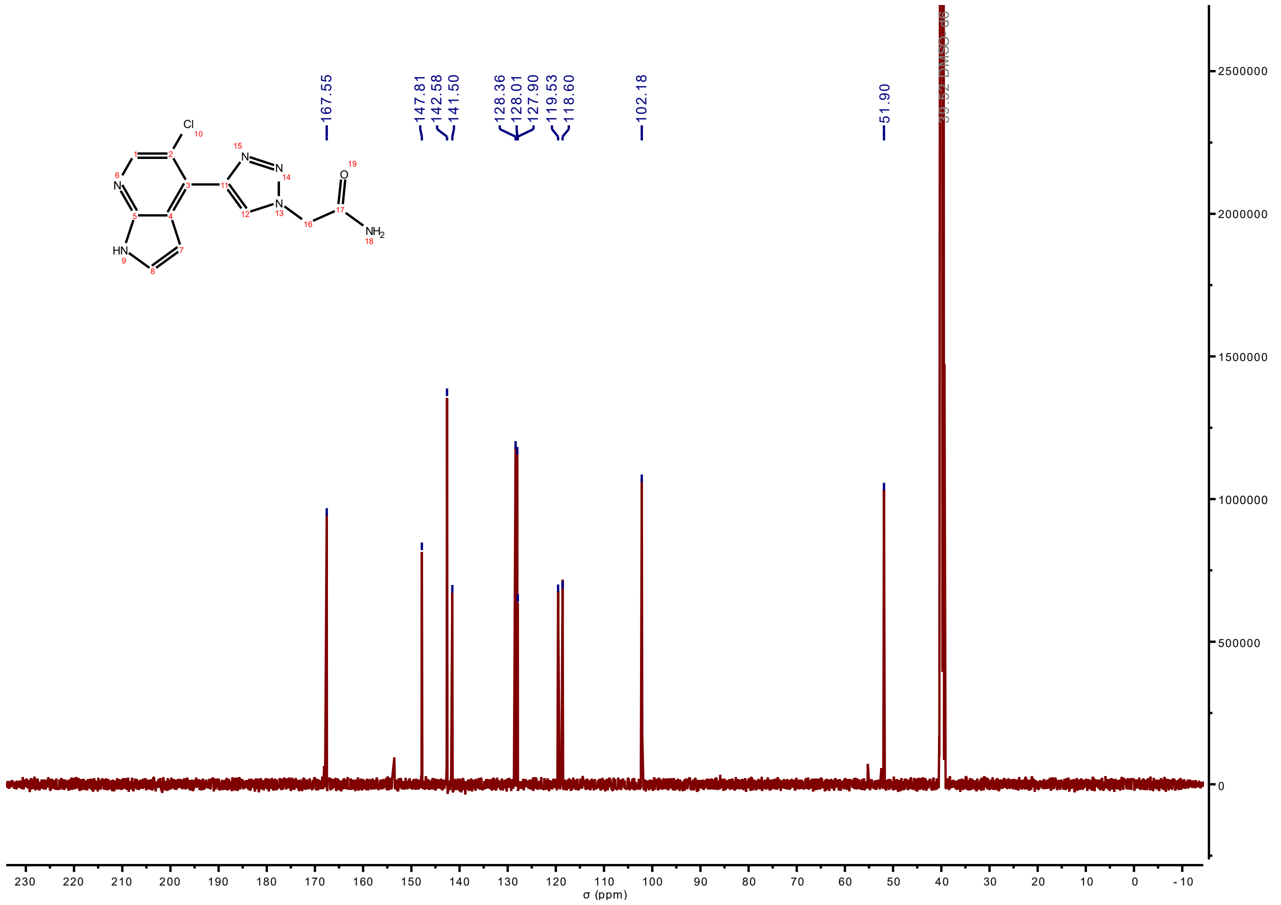

Compound 25

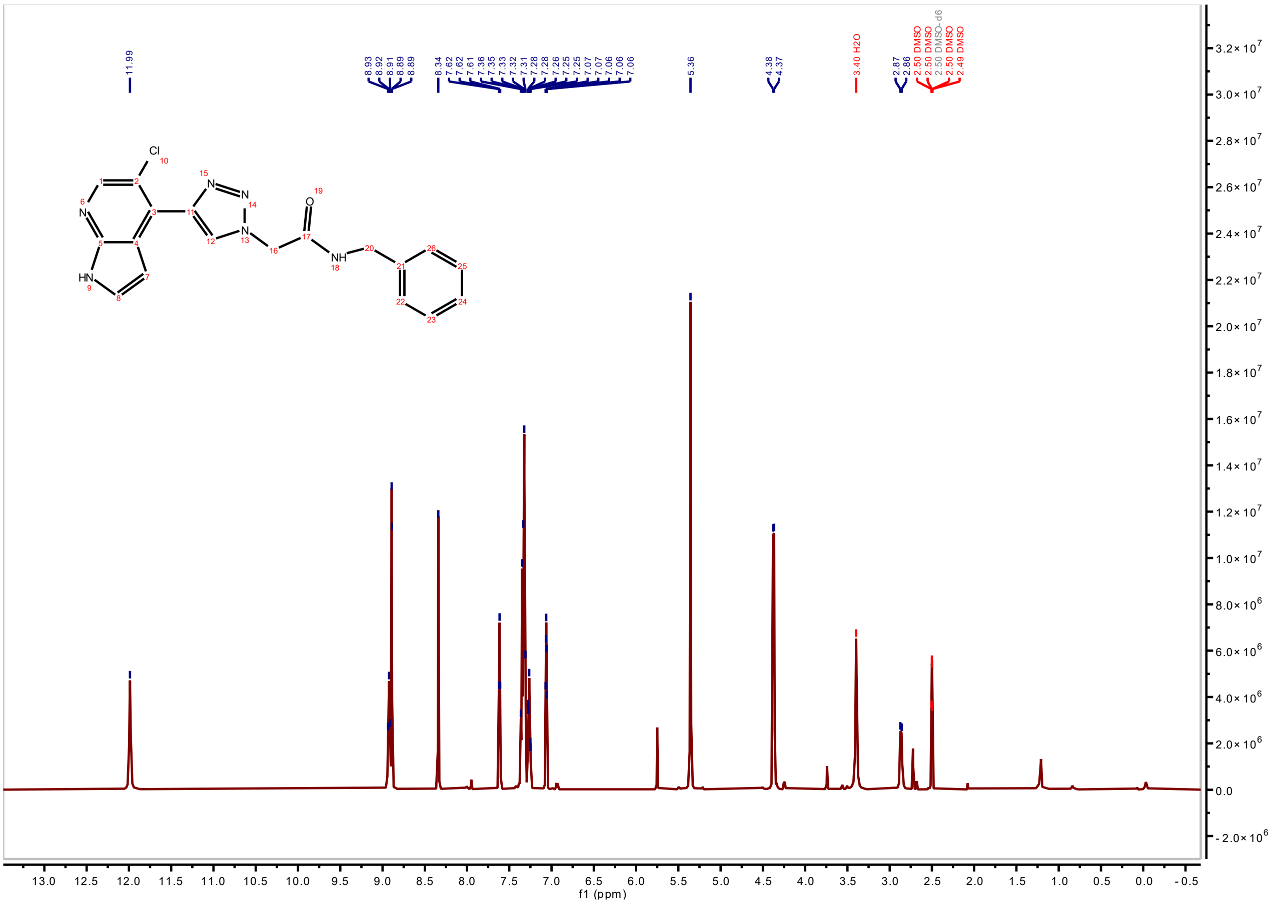

11.99
8.93
8.92
8.91
8.89
8.89
8.34
7.62
7.62
7.61
7.36
7.35
7.33
7.32
7.31
7.28
7.28
7.26
7.25
7.07
7.07
7.06
7.06
7.06
5.36
4.38
4.37
3.40 H2O
2.87
2.86
2.50 DMSO
2.50 DMSO
2.50 DMSO-d6
2.50 DMSO
2.50 DMSO
2.49 DMSO
f1 (ppm)

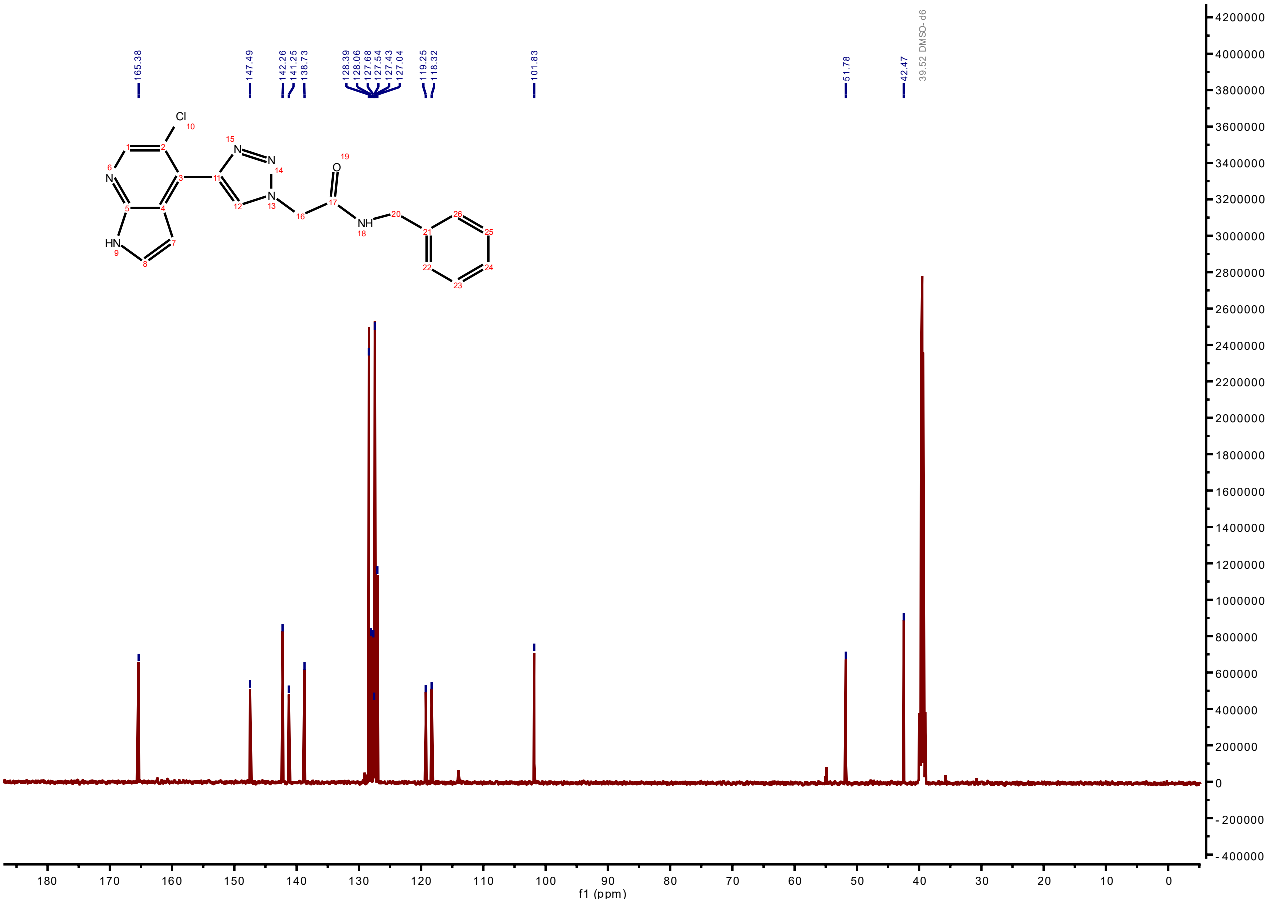

165.38
147.49
142.26
141.25
138.73
128.39
128.06
127.68
127.54
127.43
127.04
119.25
118.32
101.83
51.78
42.47
39.52 DMSO-d6
f1 (ppm)

Compound 26

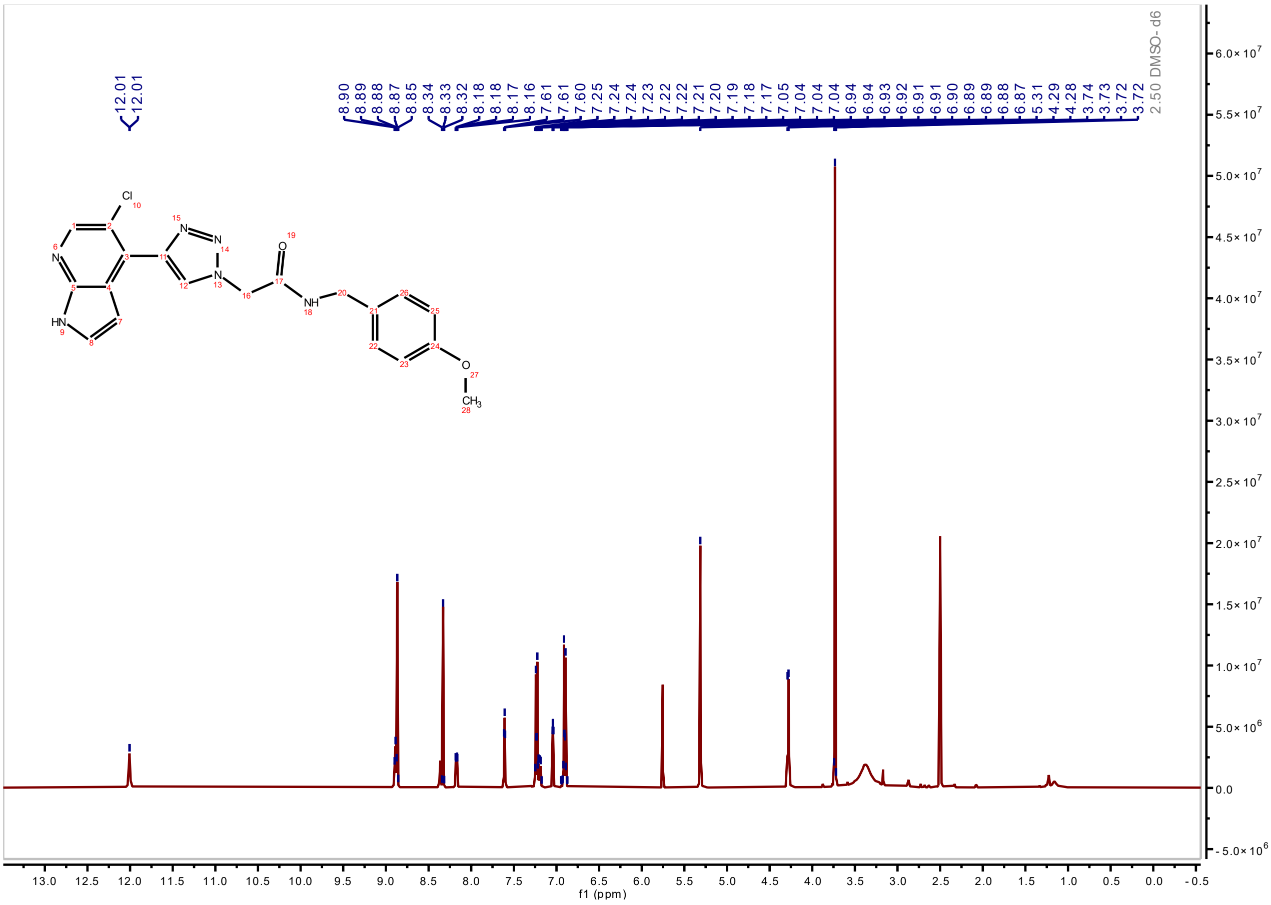
12.01
12.01
8.90
8.89
8.88
8.87
8.85
8.34
8.33
8.32
8.18
8.18
8.17
8.16
7.61
7.61
7.60
7.25
7.24
7.24
7.23
7.22
7.22
7.21
7.20
7.19
7.18
7.17
7.05
7.04
7.04
6.94
6.93
6.92
6.91
6.91
6.90
6.89
6.89
6.88
6.87
5.31
4.29
4.28
3.74
3.73
3.72
3.72
2.50 DMSO- d6
f1 (ppm)

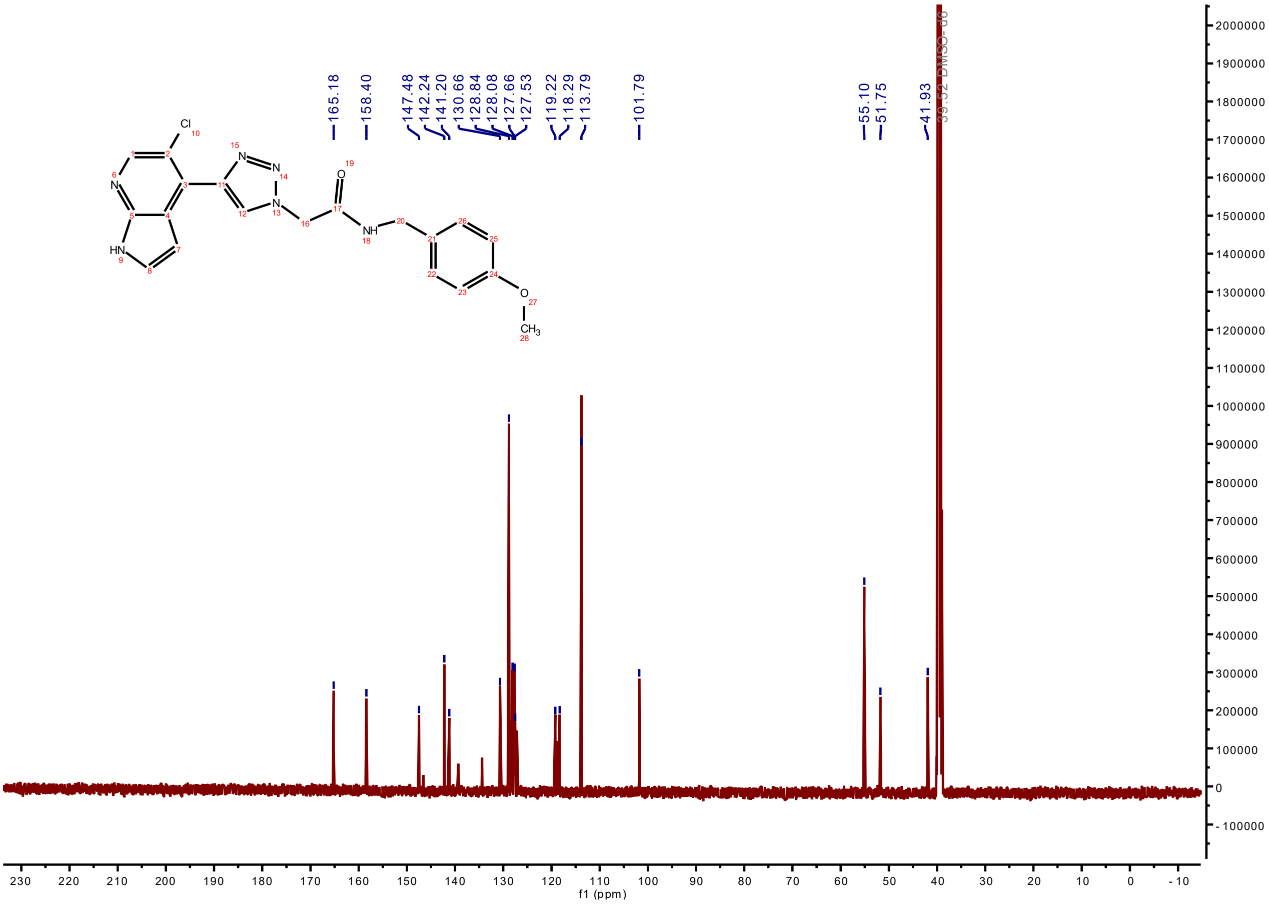
165.18
158.40
147.48
142.24
141.20
130.66
128.84
128.08
127.66
127.53
119.22
118.29
113.79
101.79
55.10
51.75
41.93
39.52 DMSO- d6
f1 (ppm)

Compound 14b1

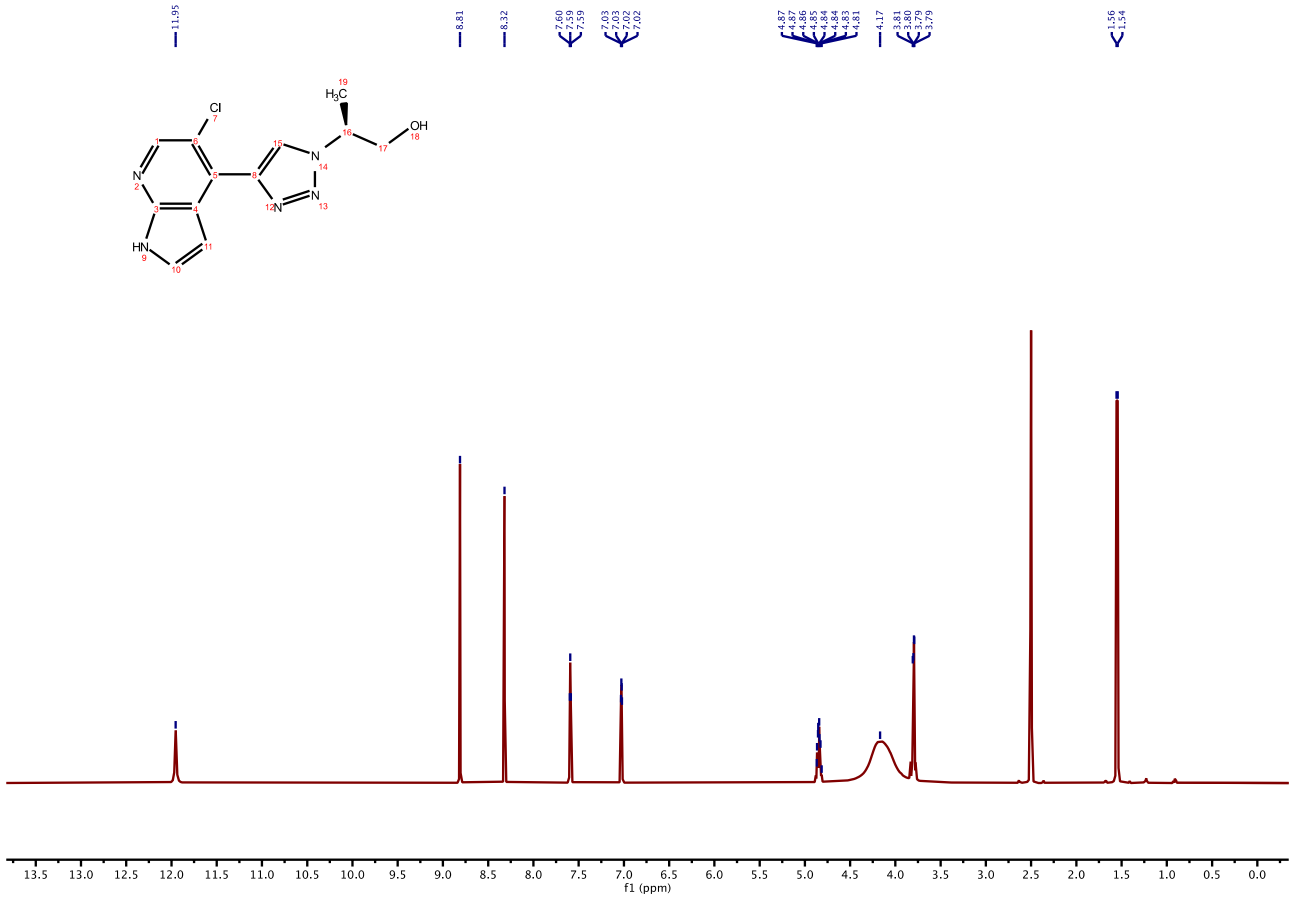

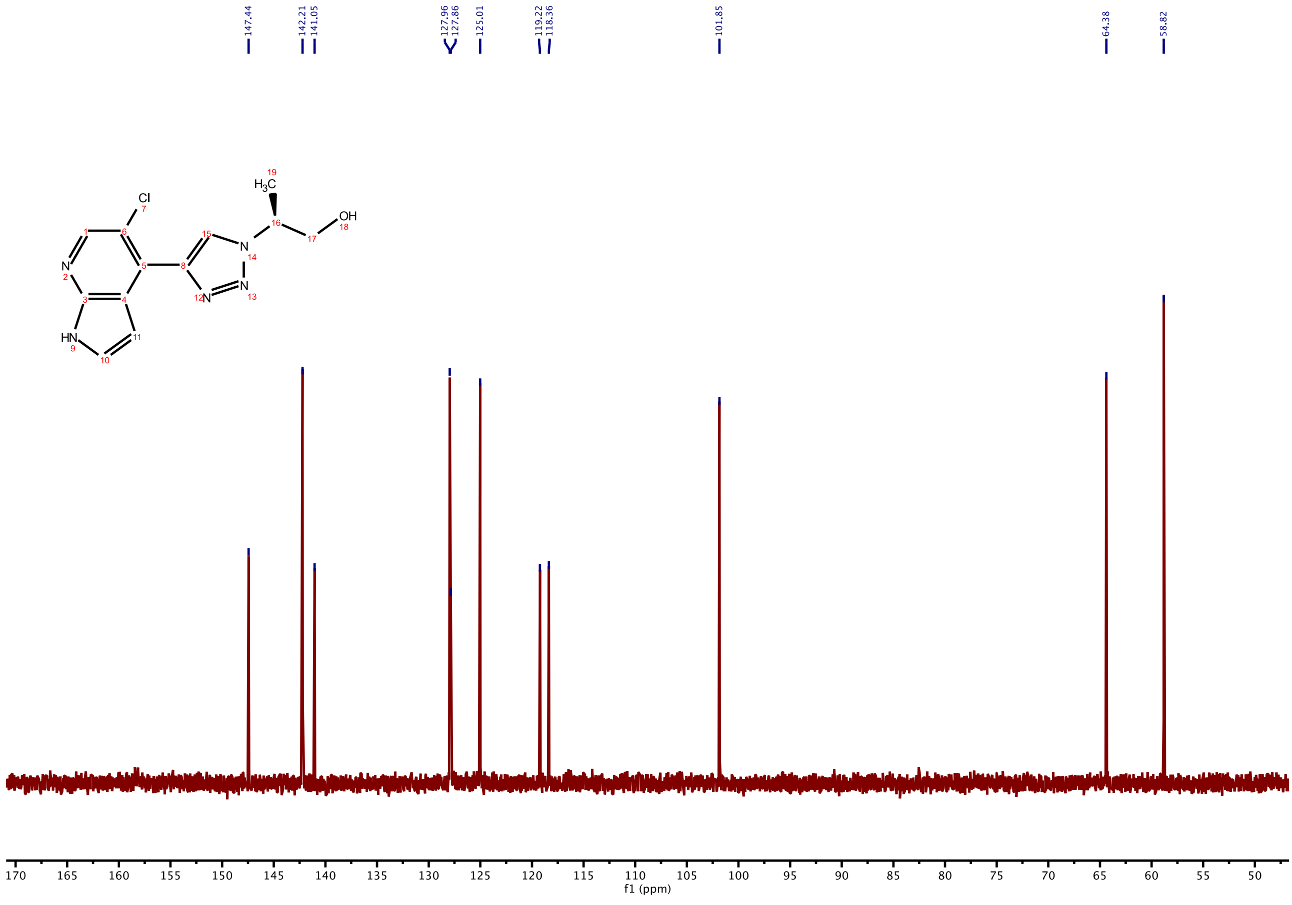

Compound 14b2

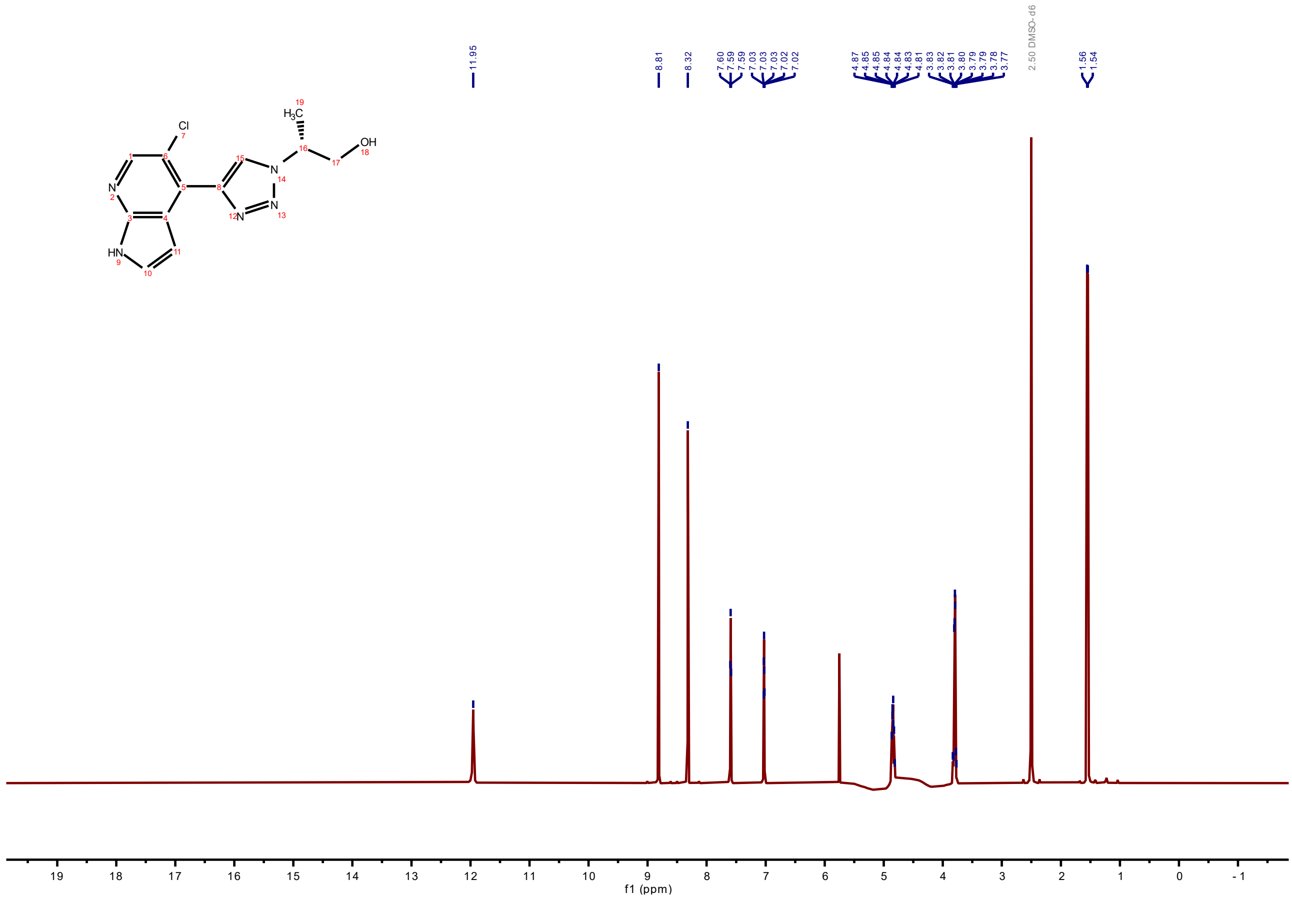

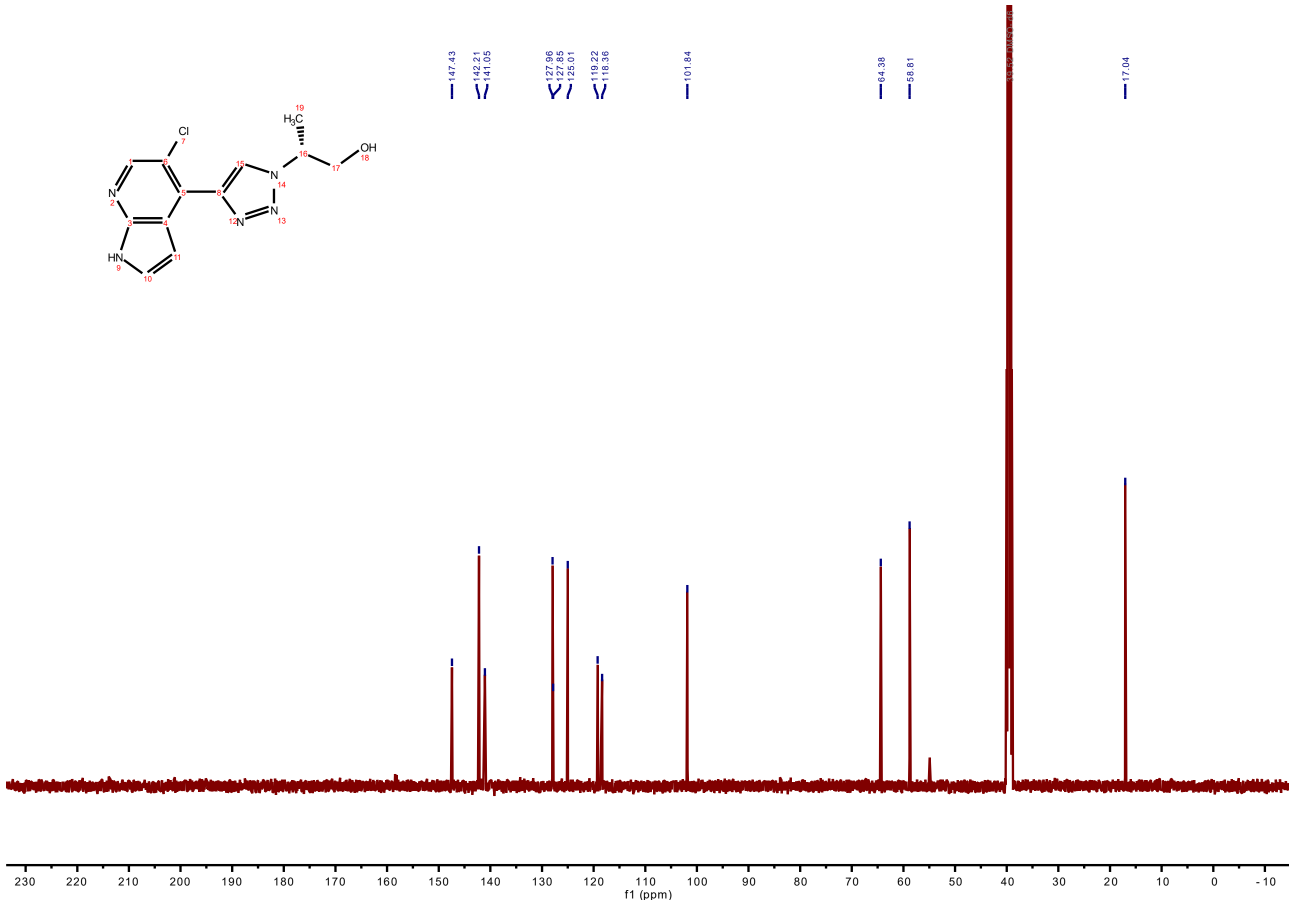

Compound 14b3

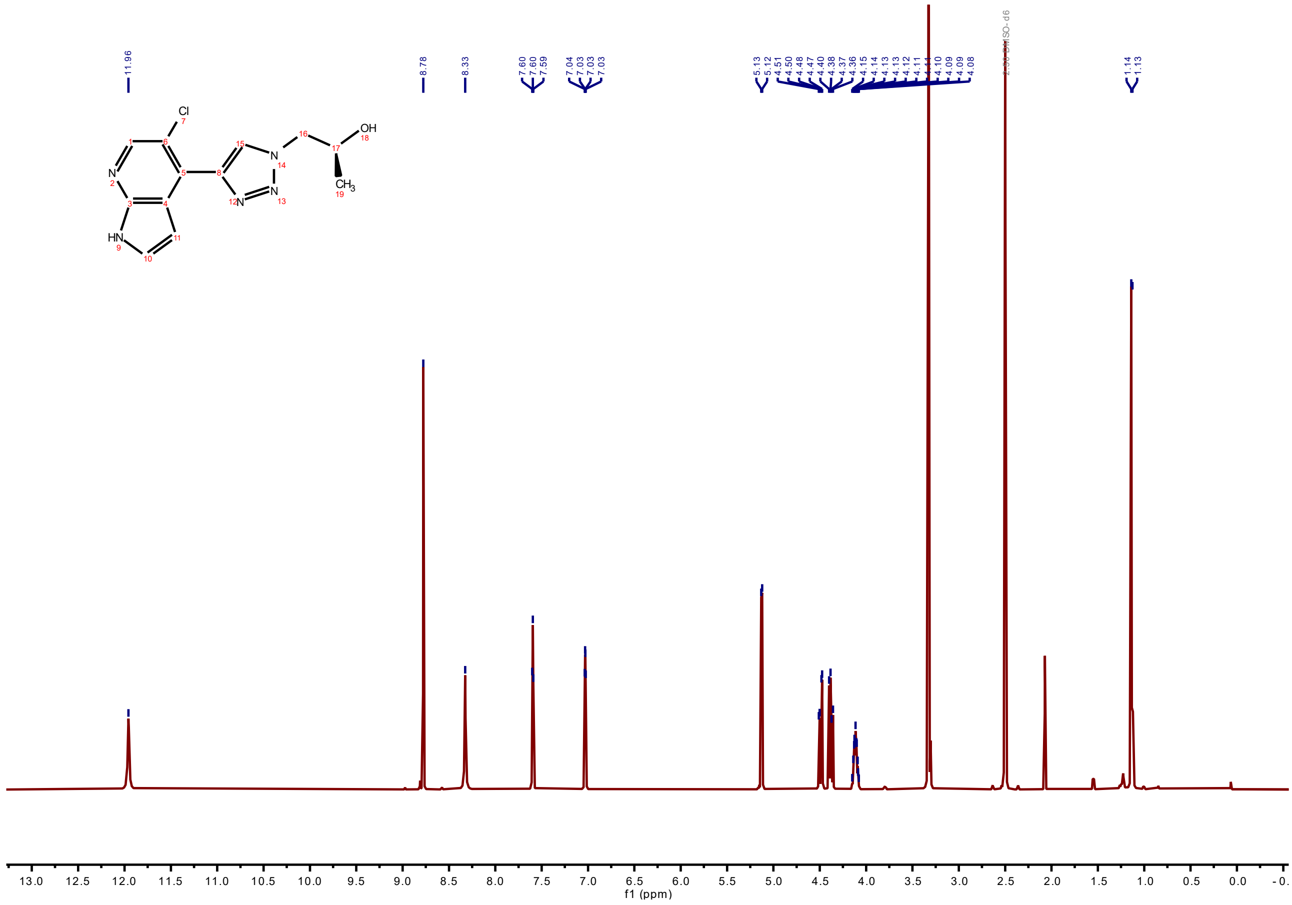

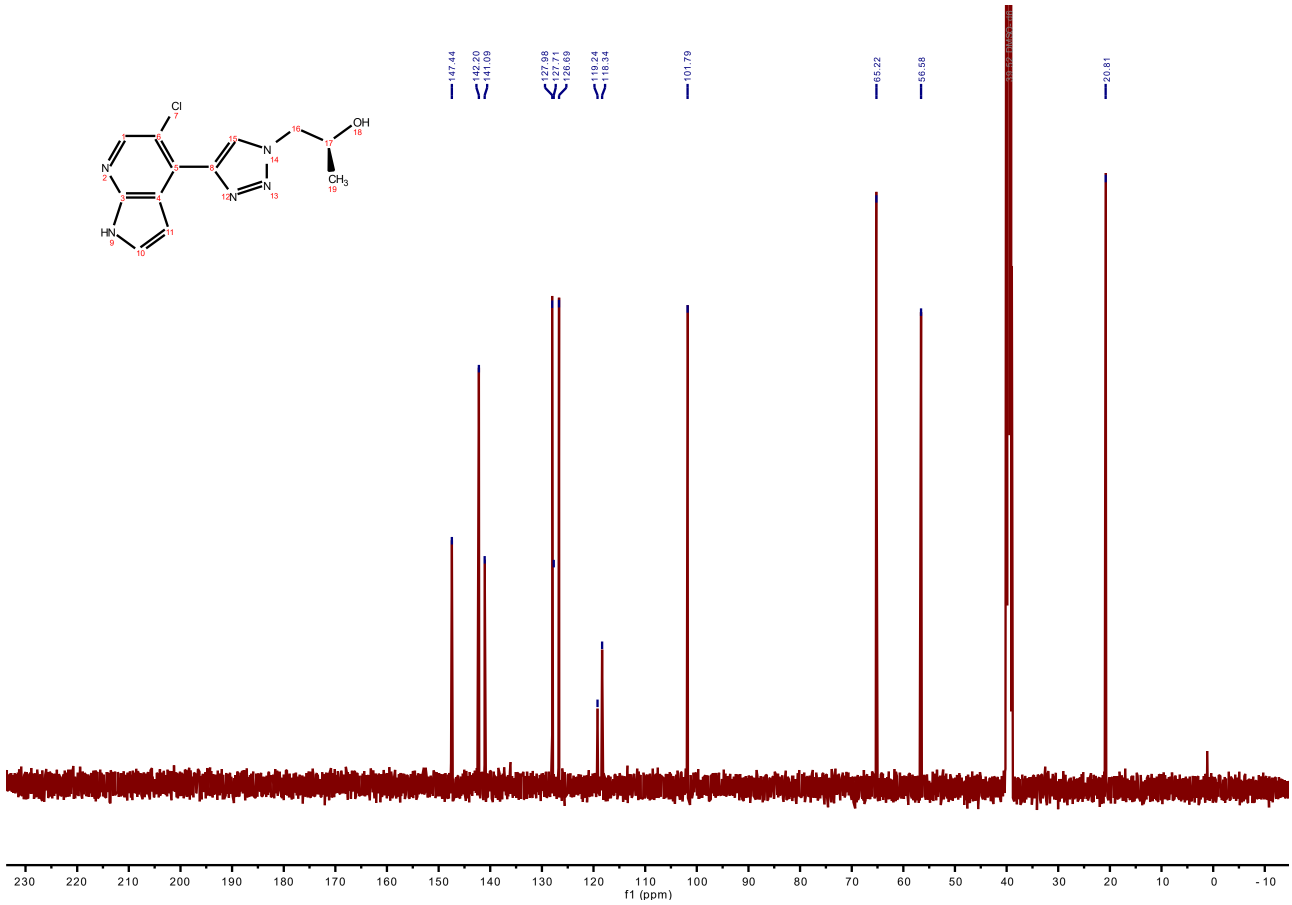

Compound 14b4

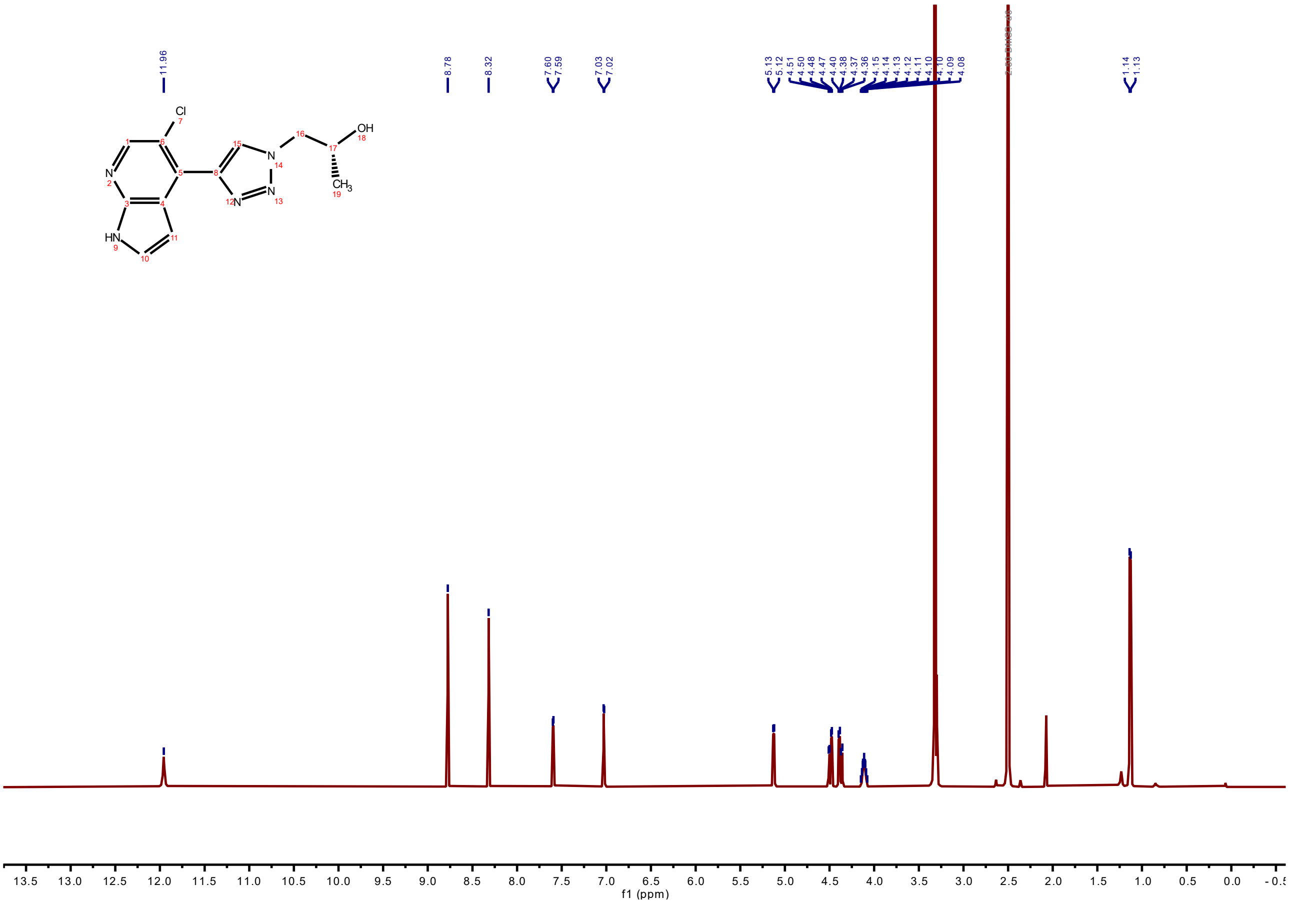

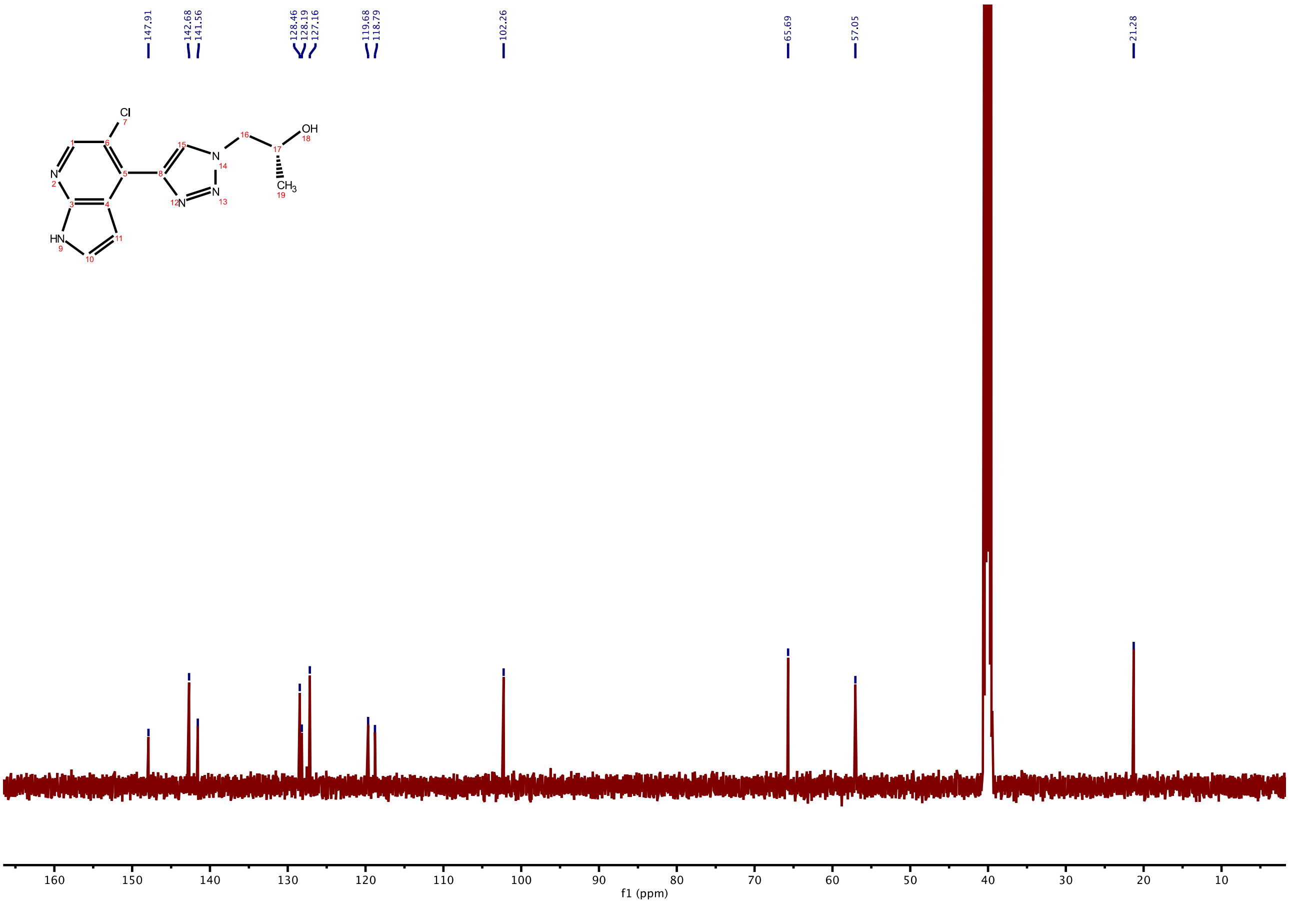

Compound 14c1

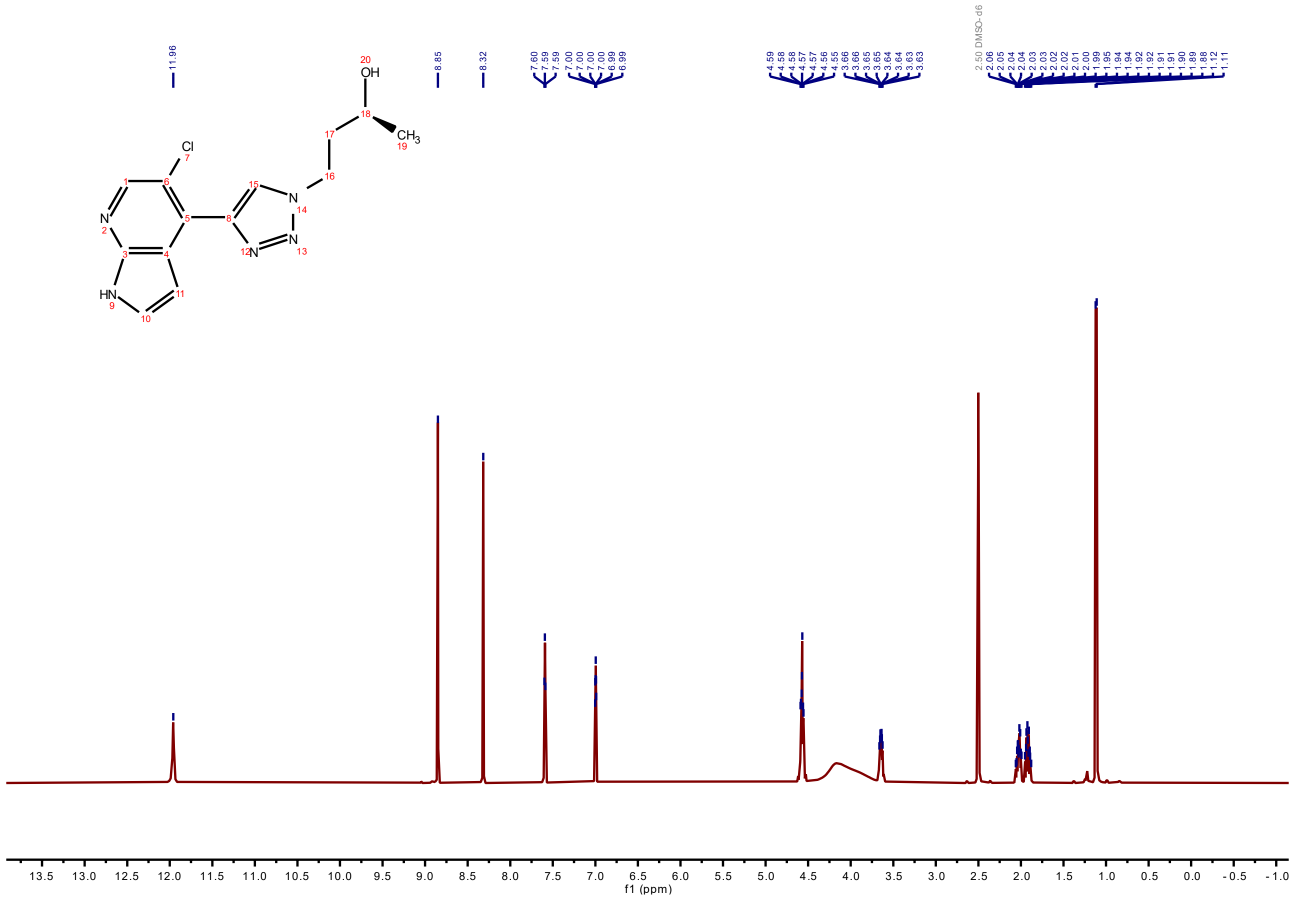

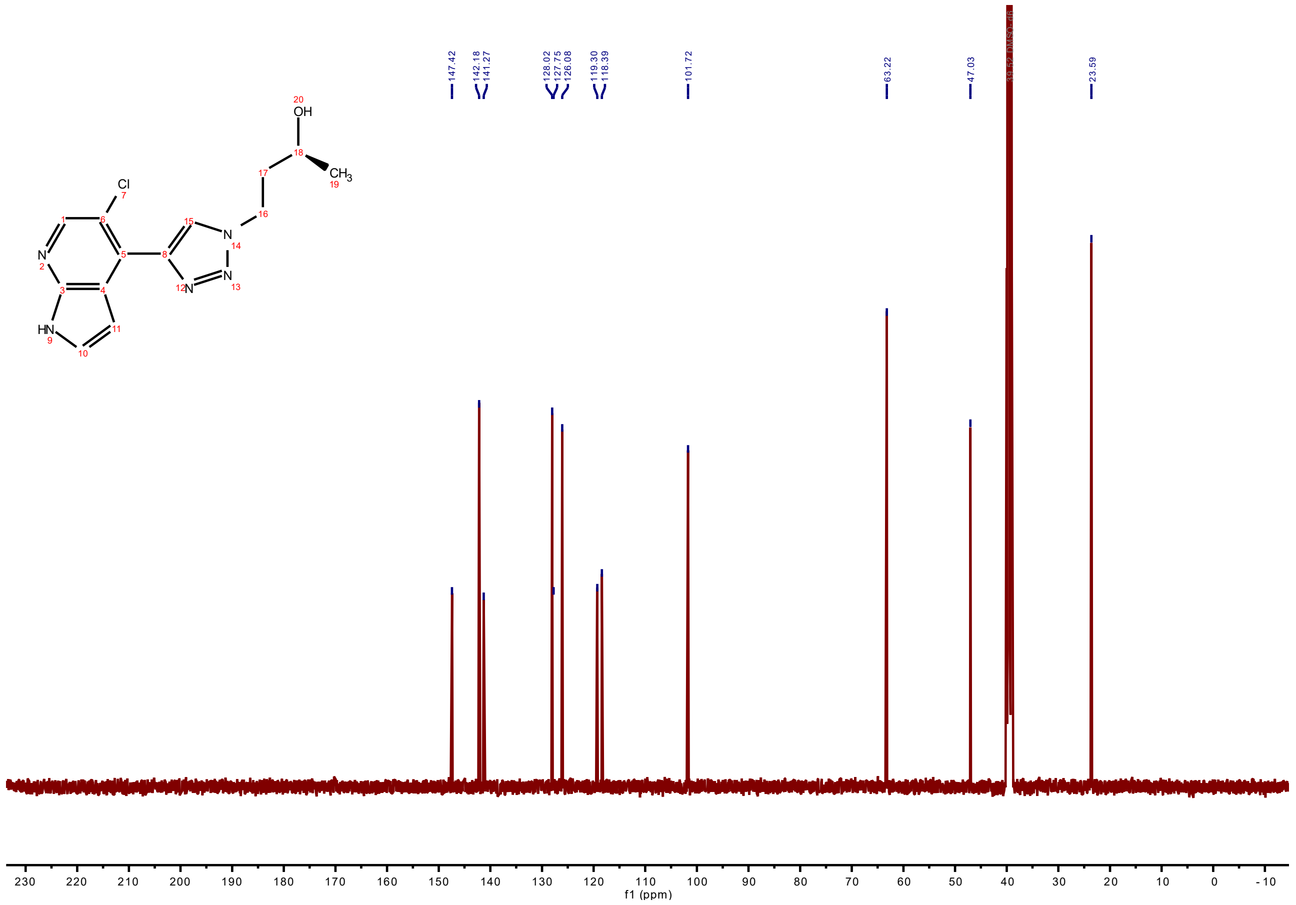

Compound 14c2

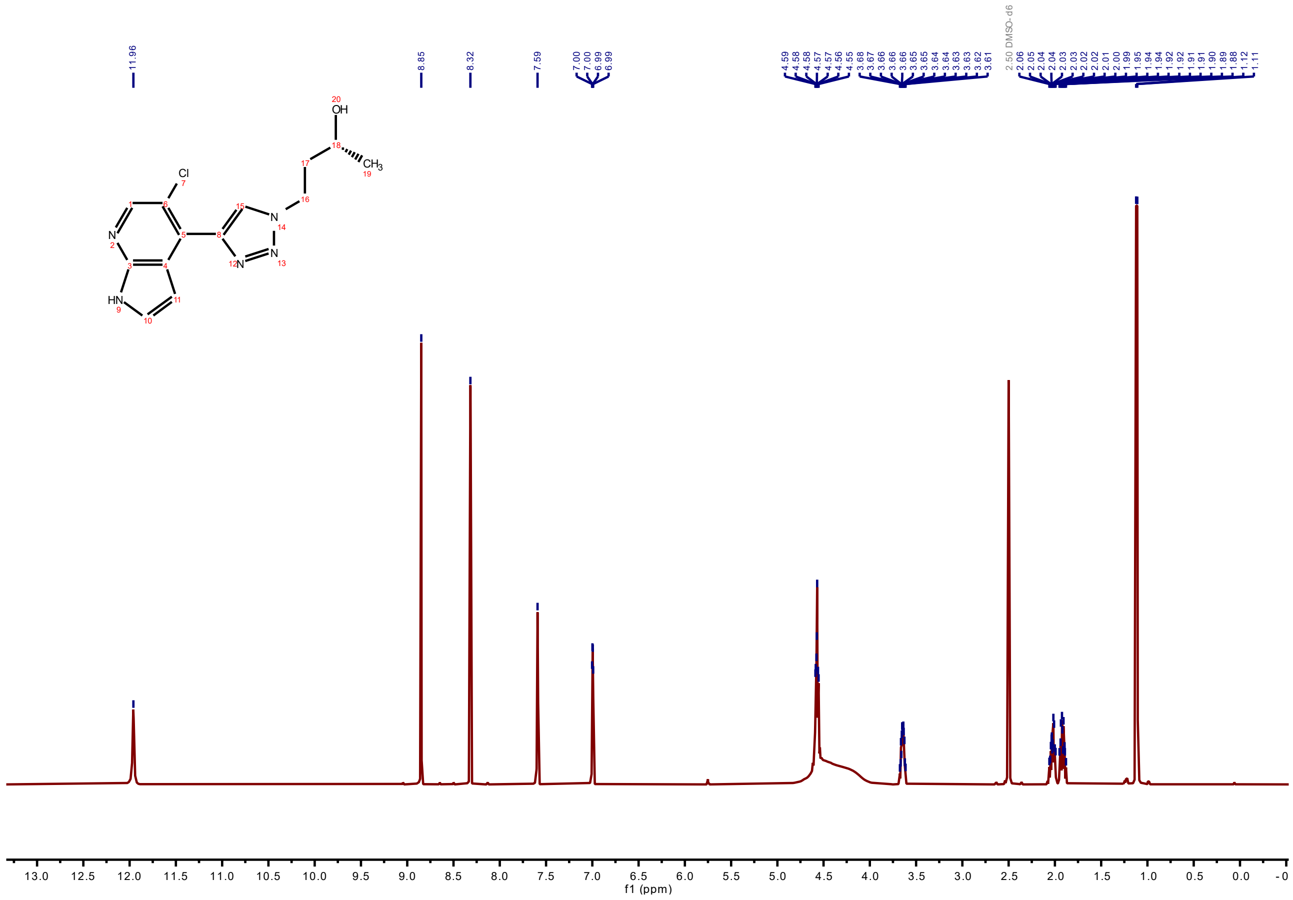

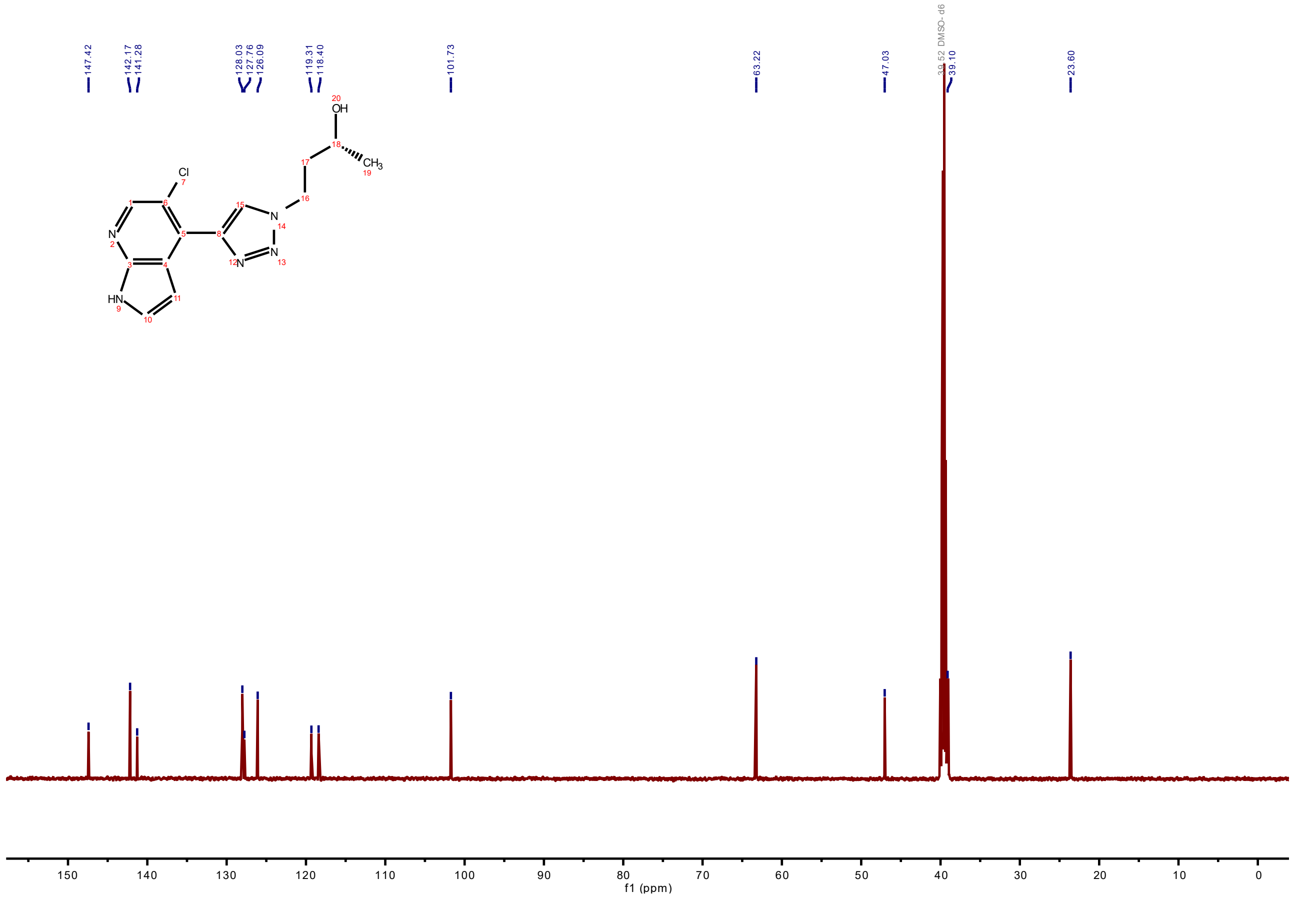

Compound 14c3

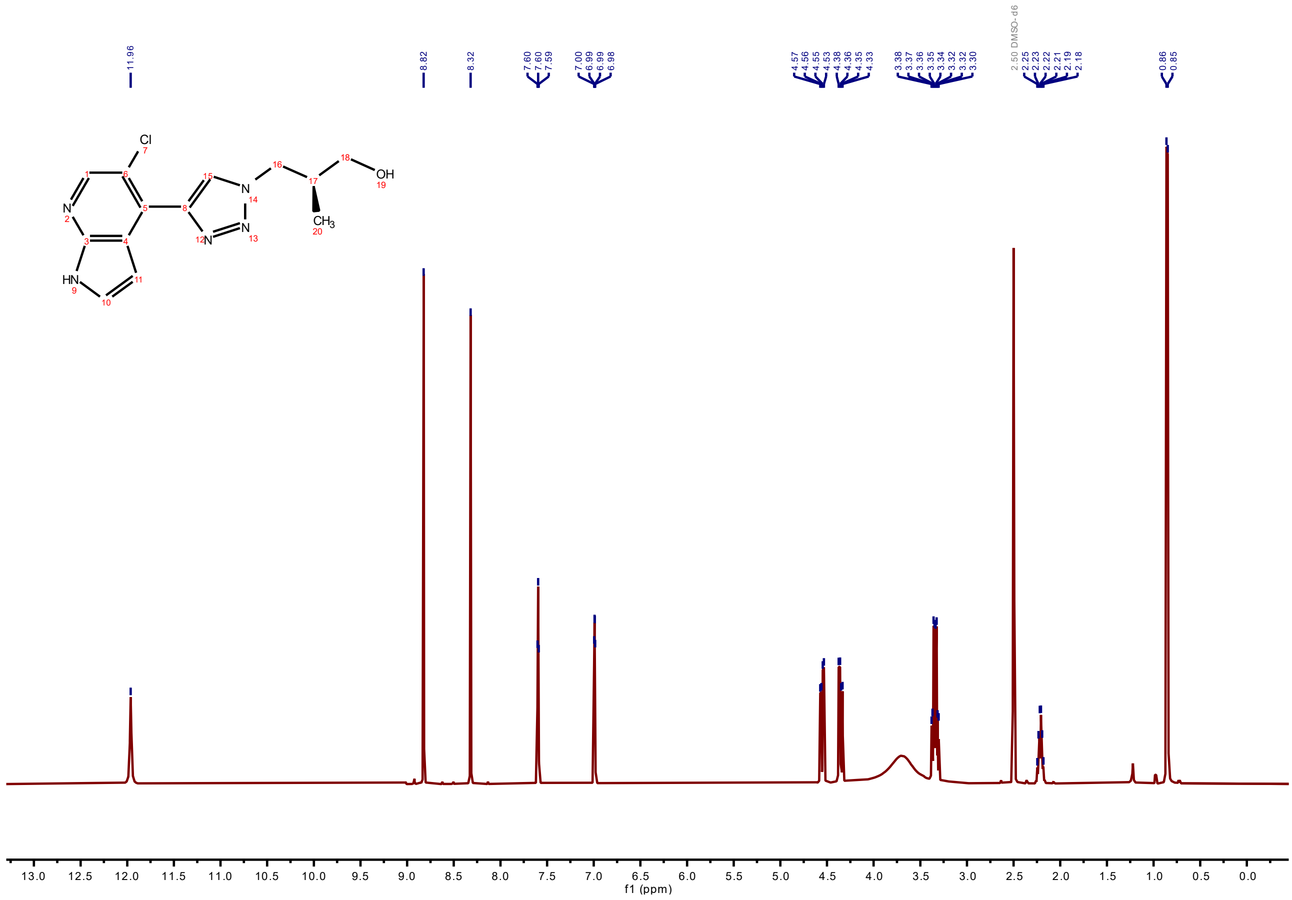

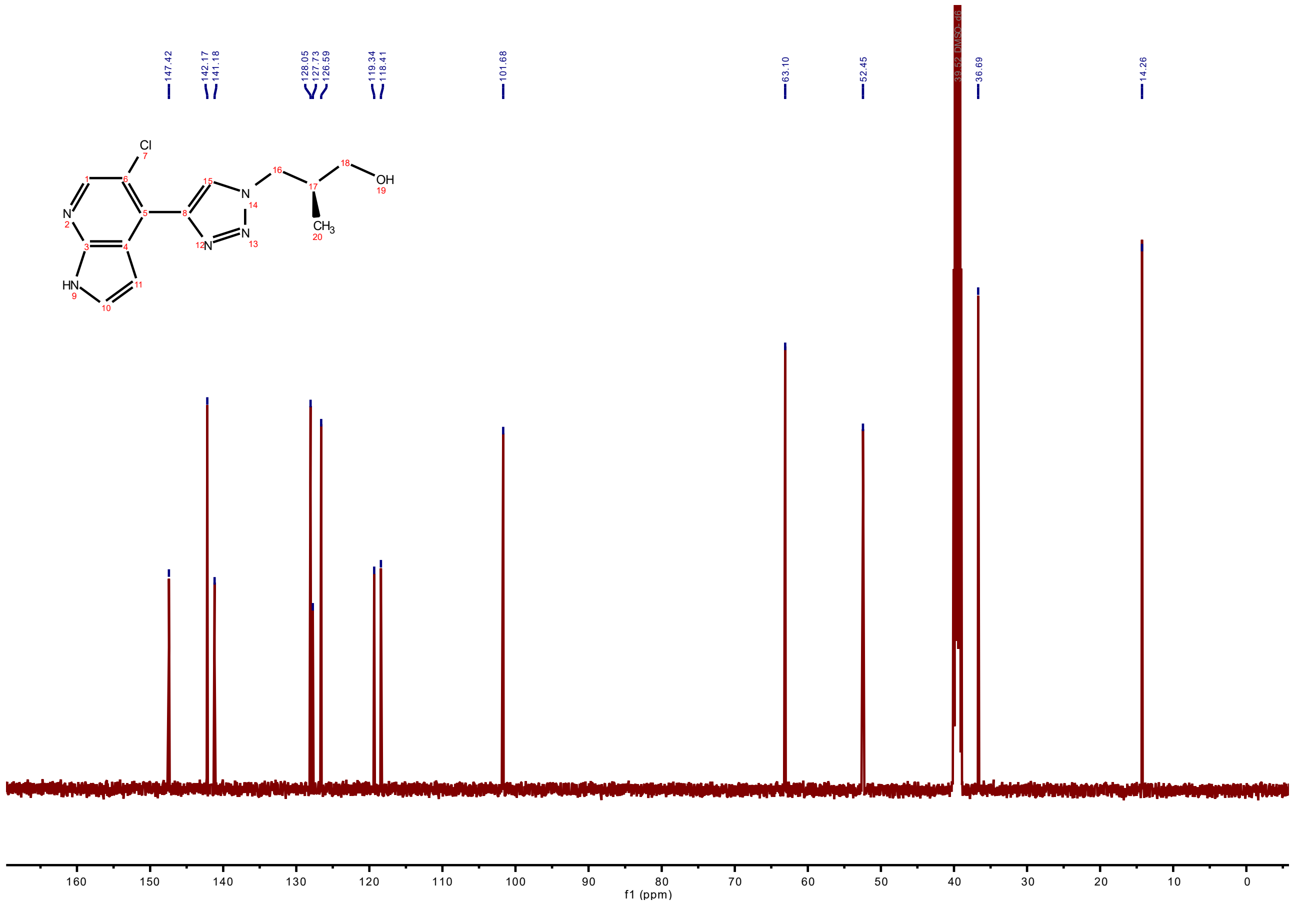

Compound 14c4

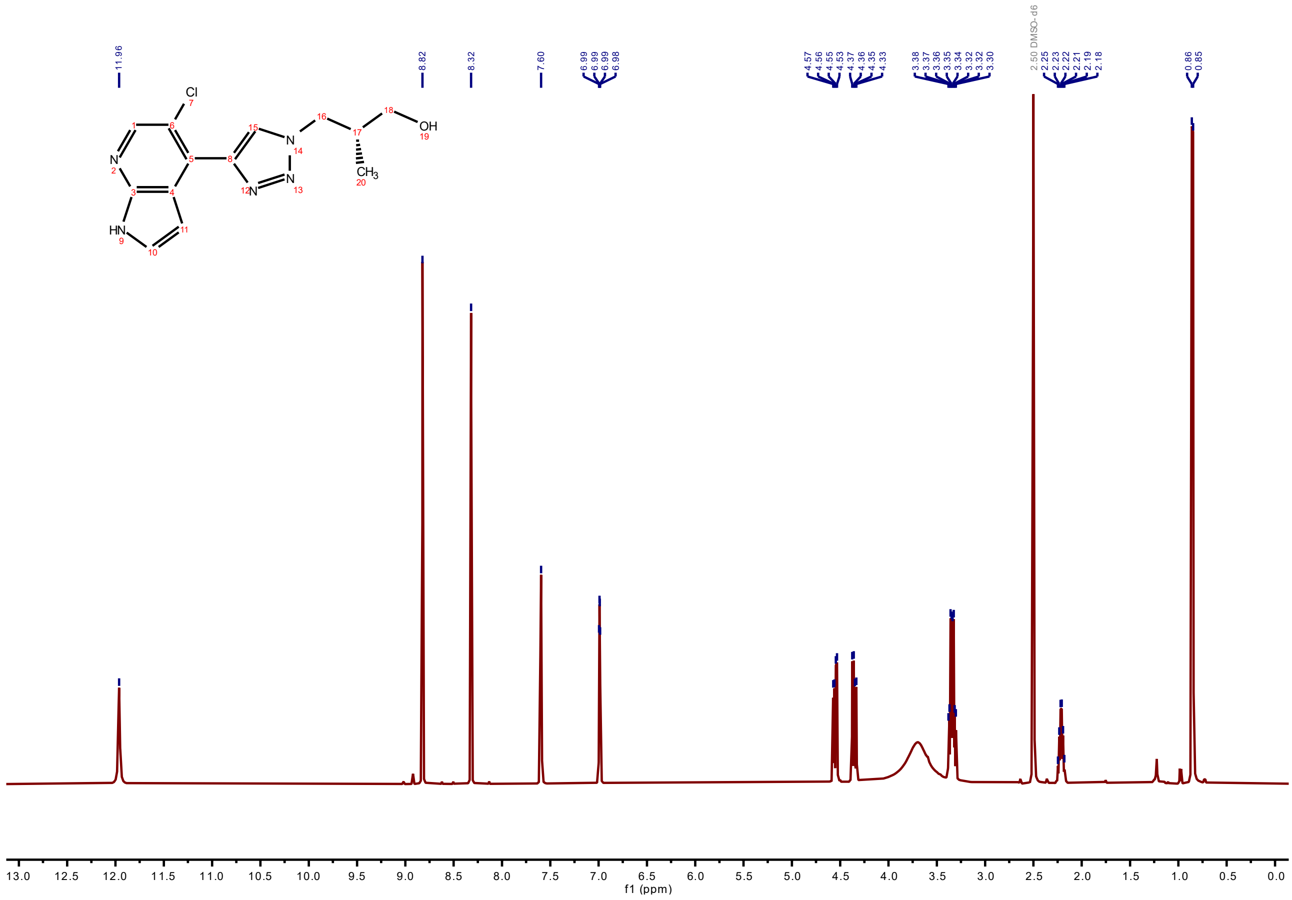

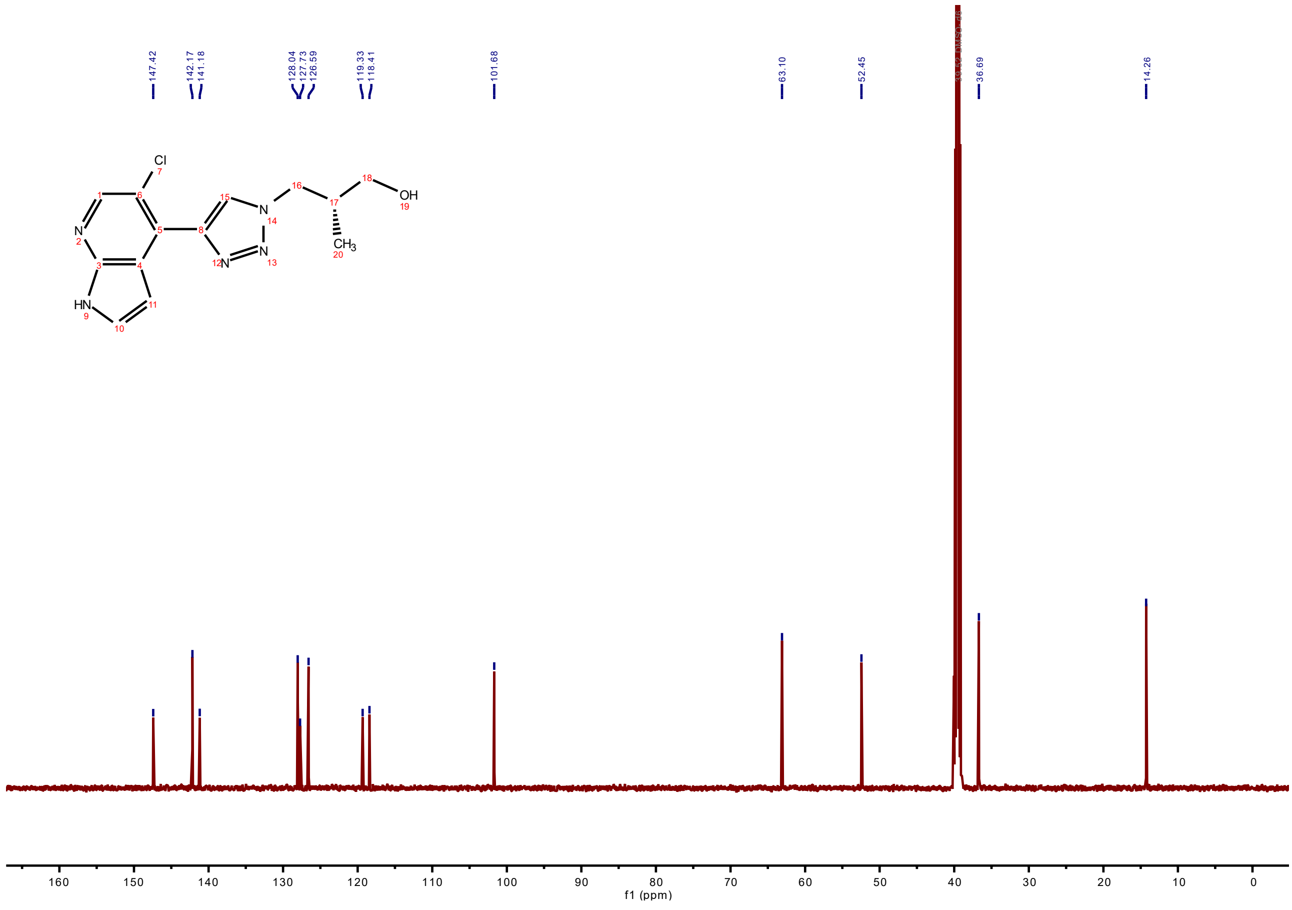

Compound 14c5

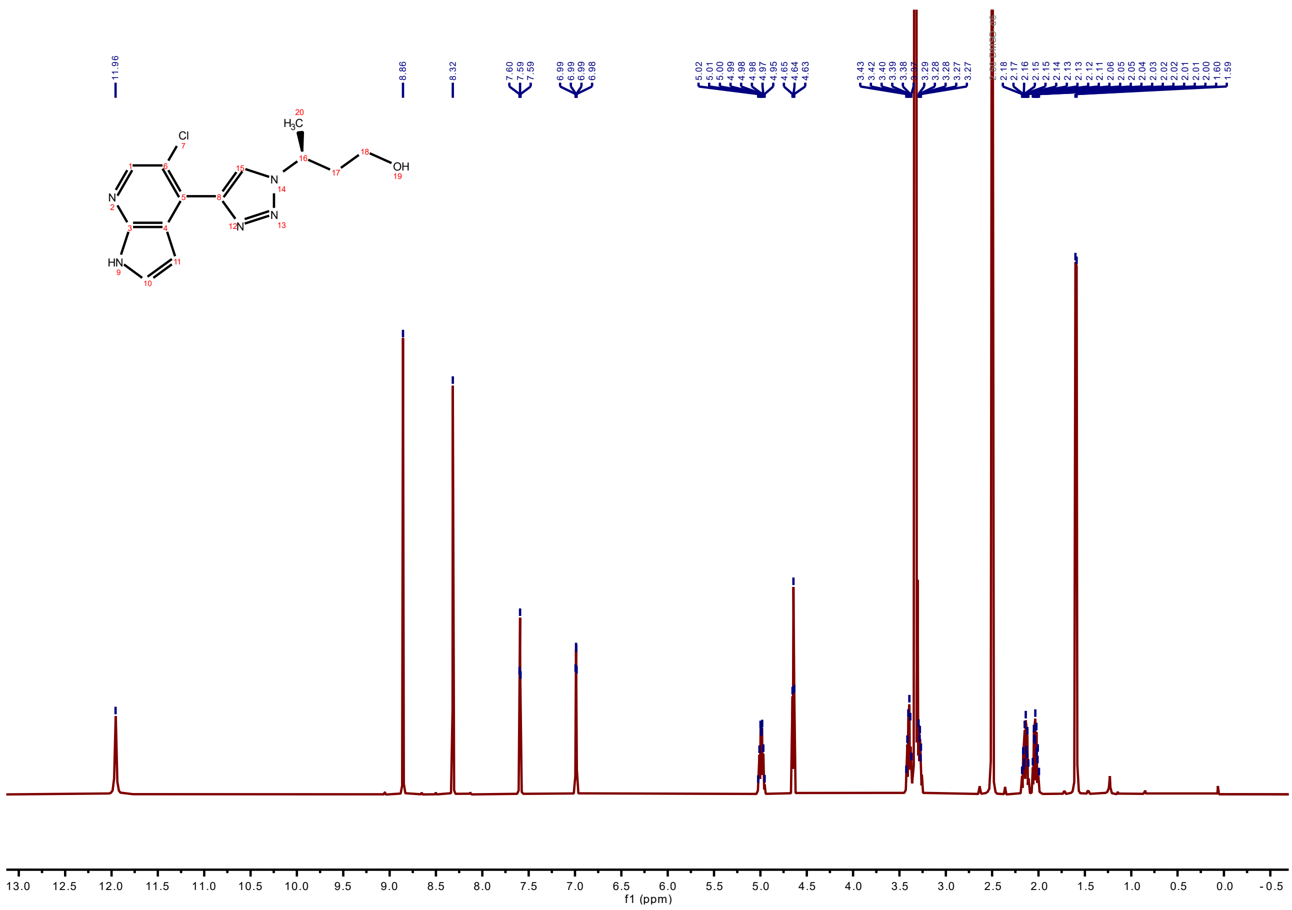

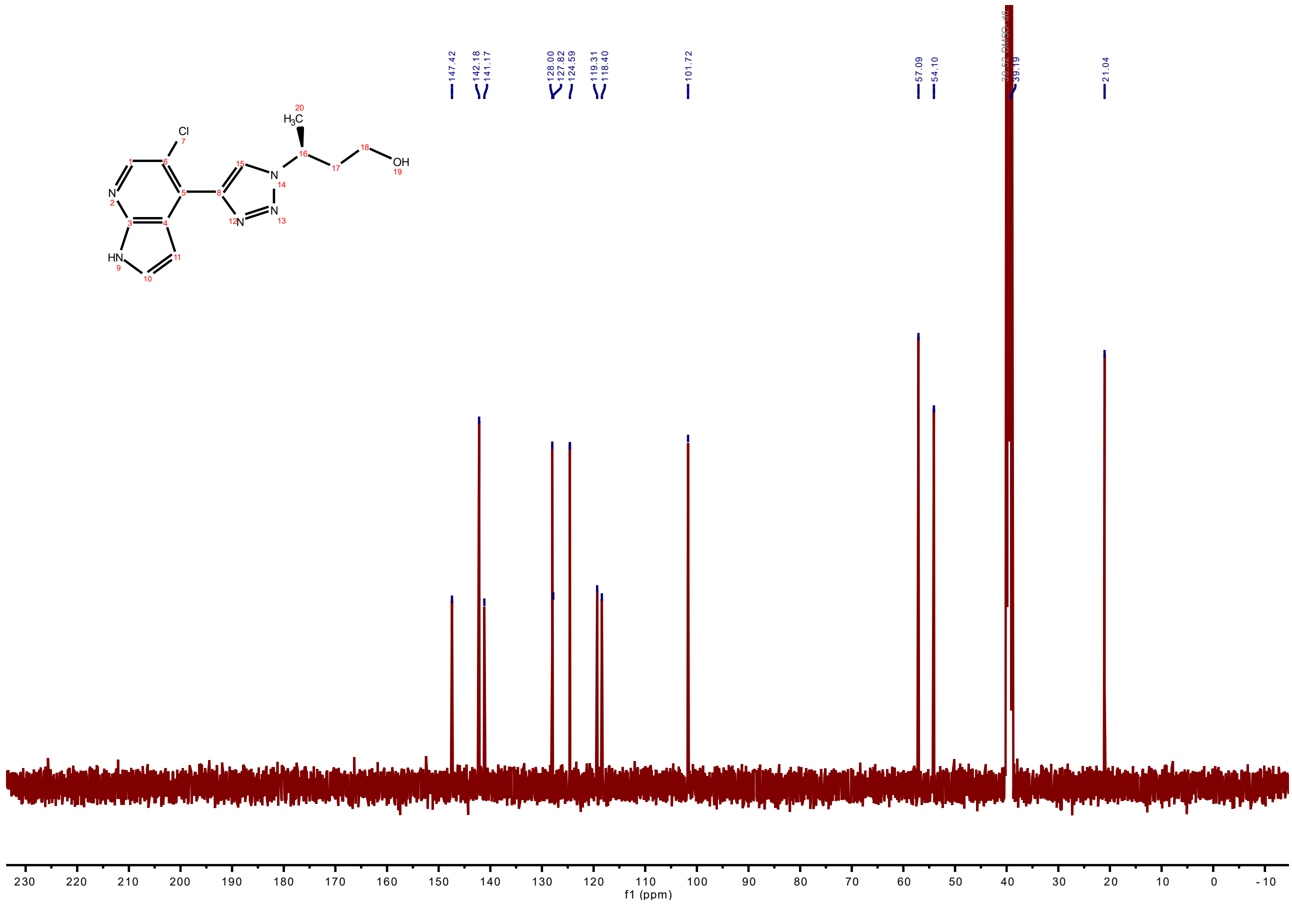

Compound 14c6

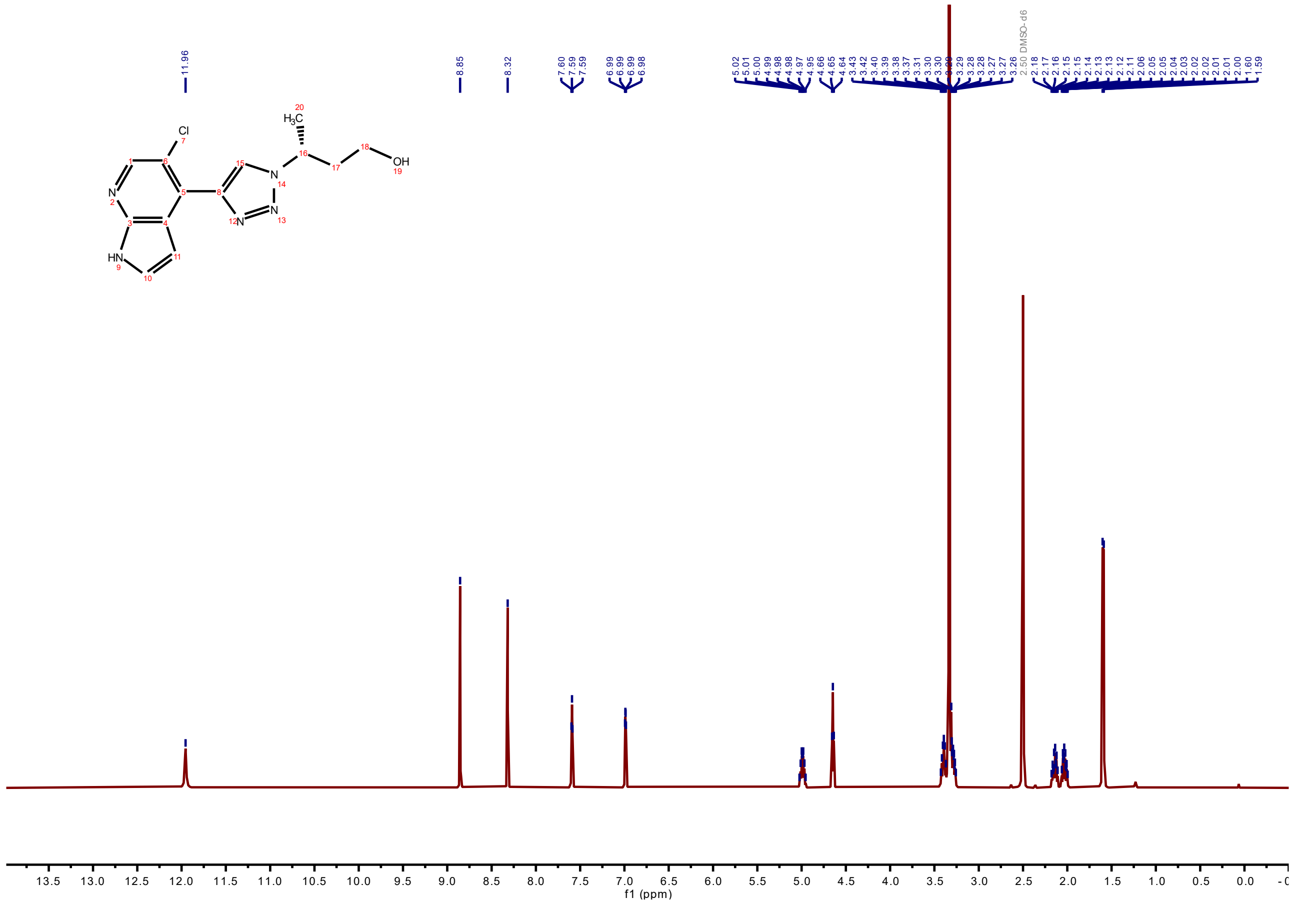

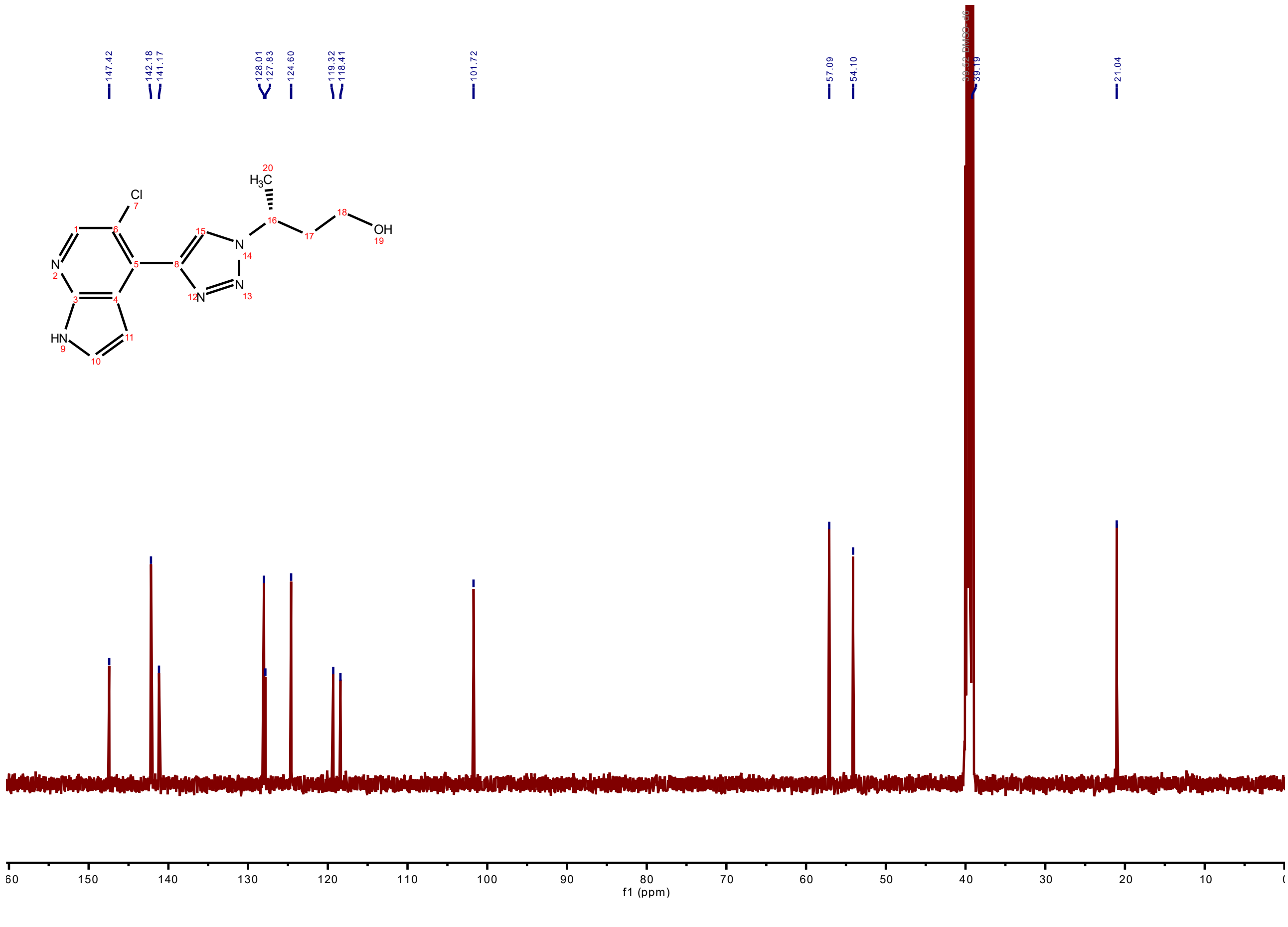

Compound 14m

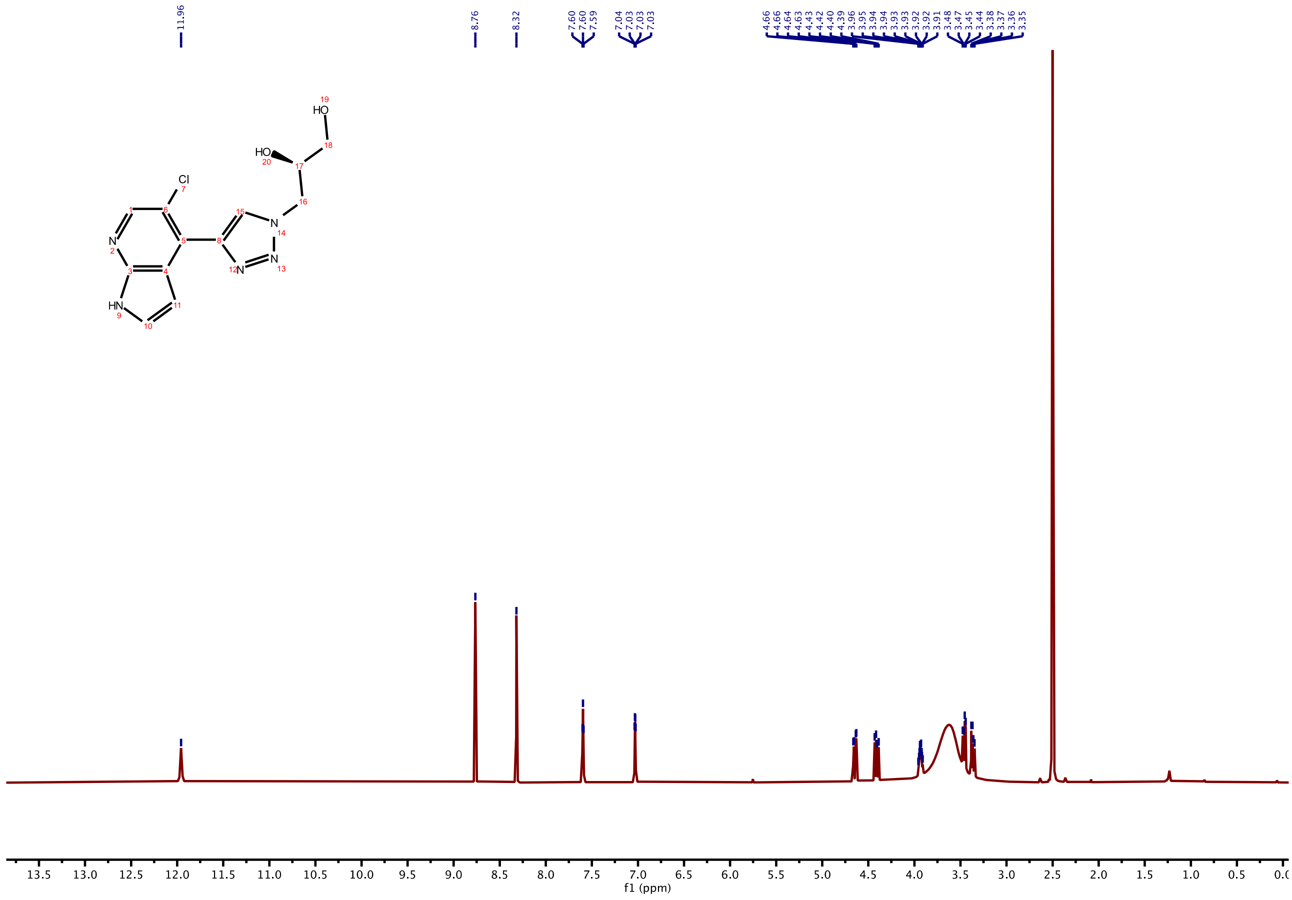

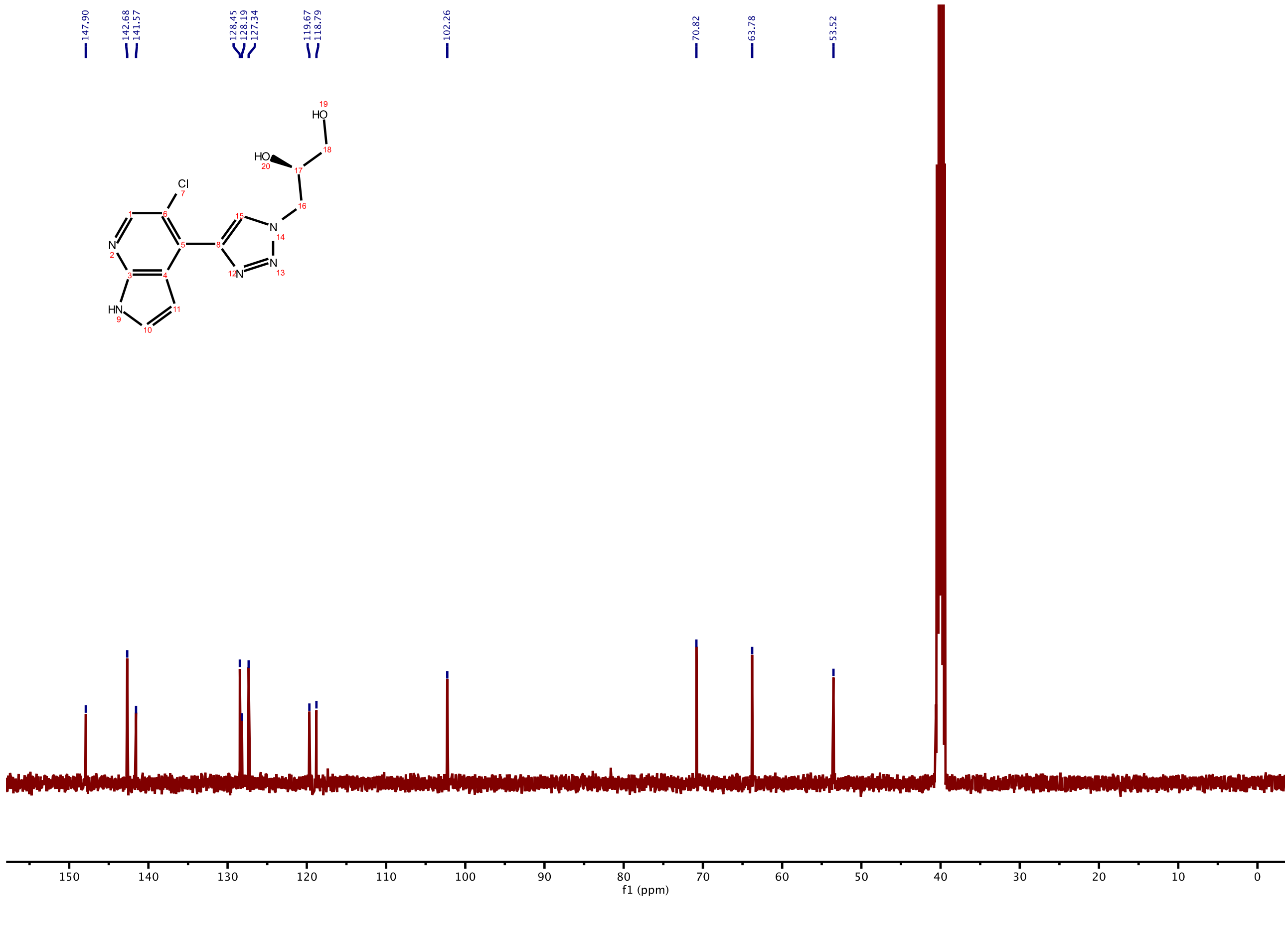

Compound 14n

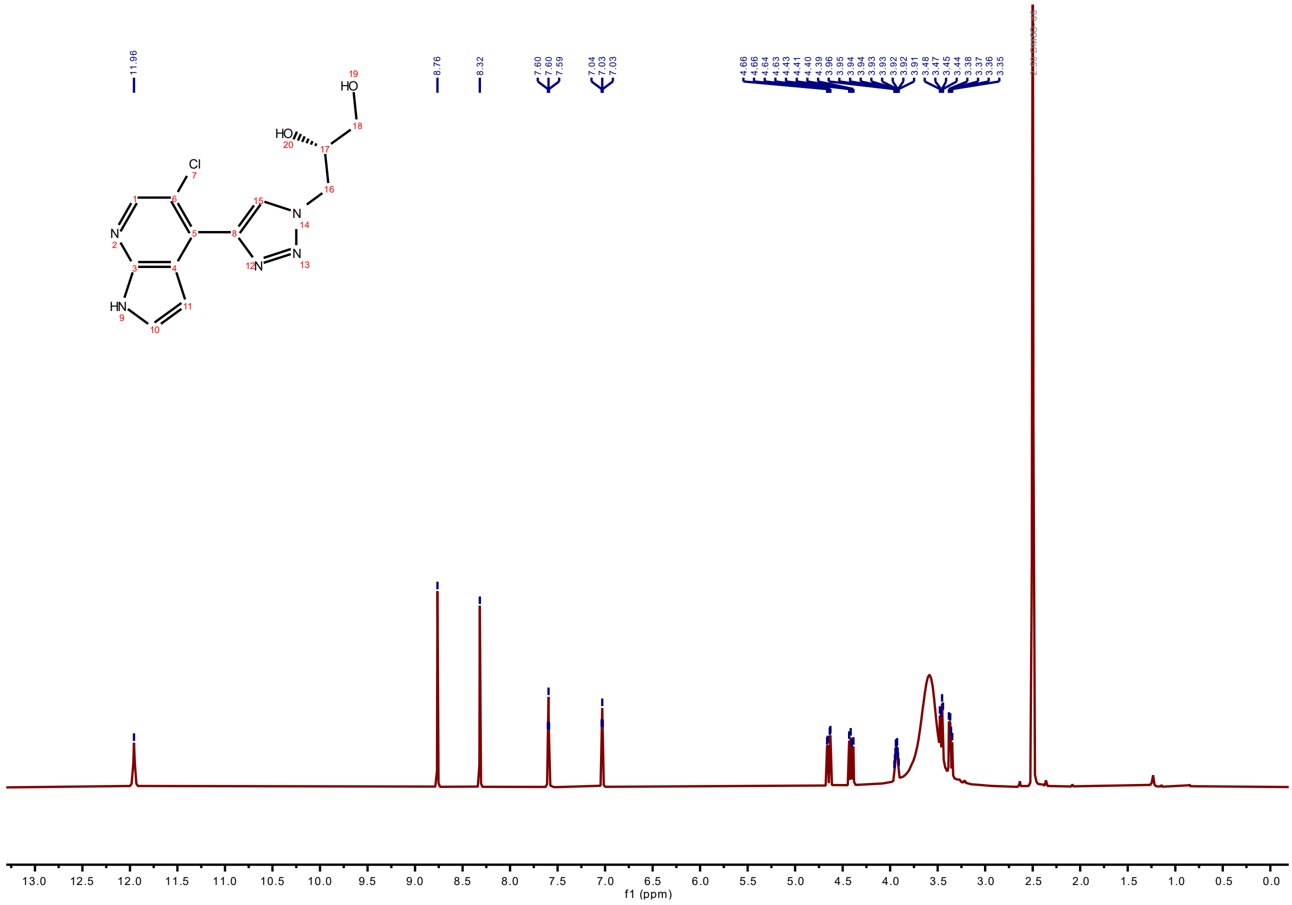

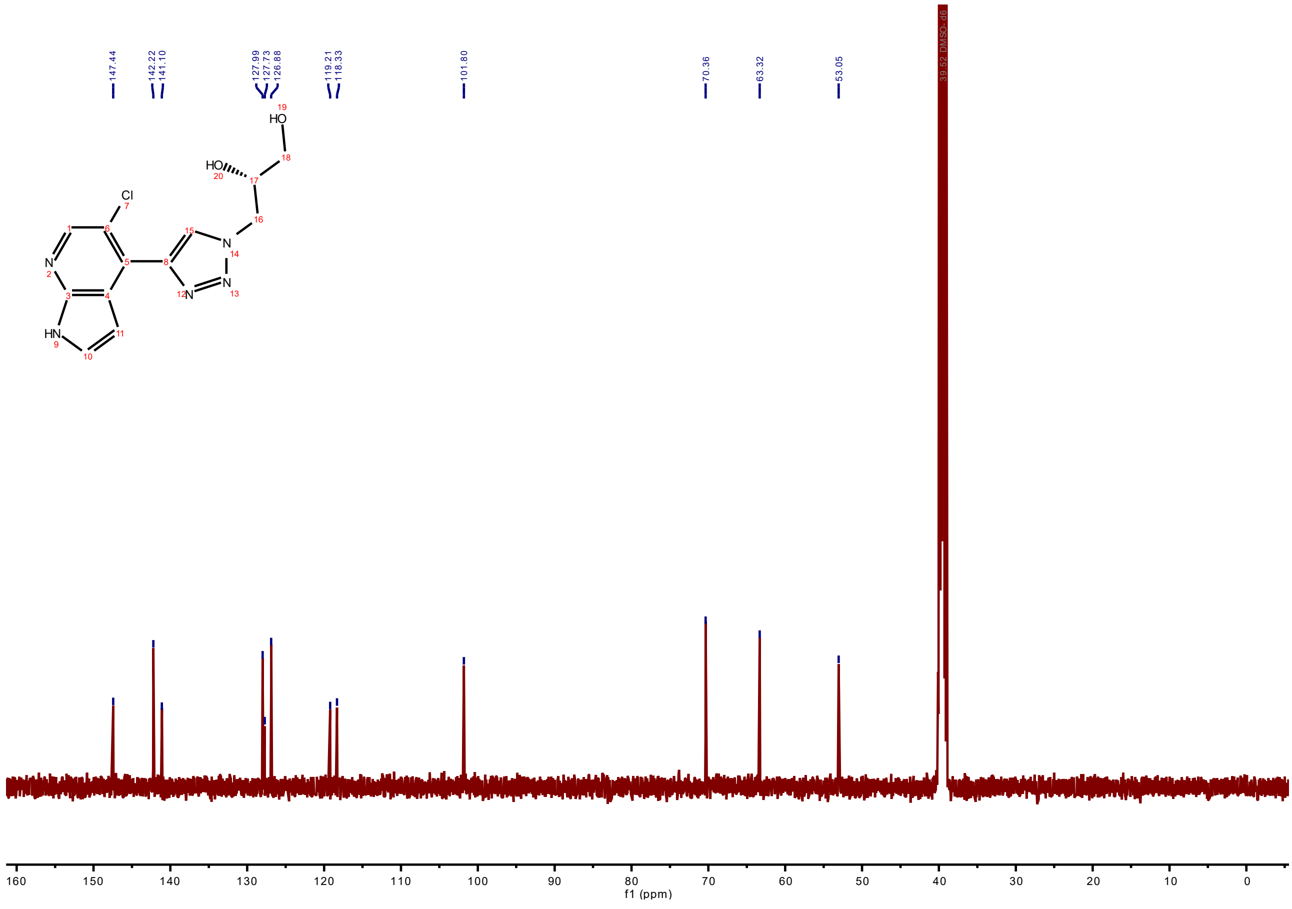

Compound 19

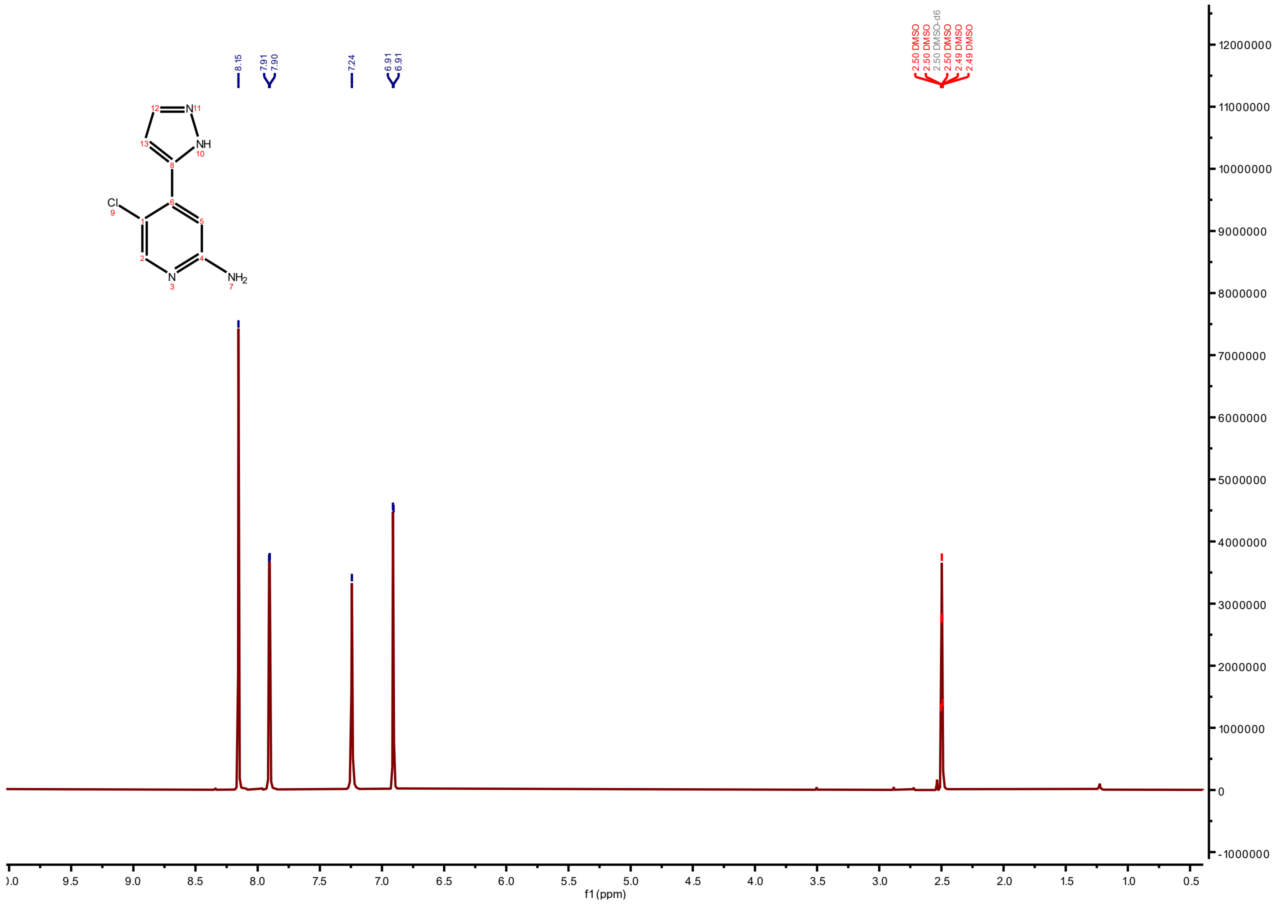

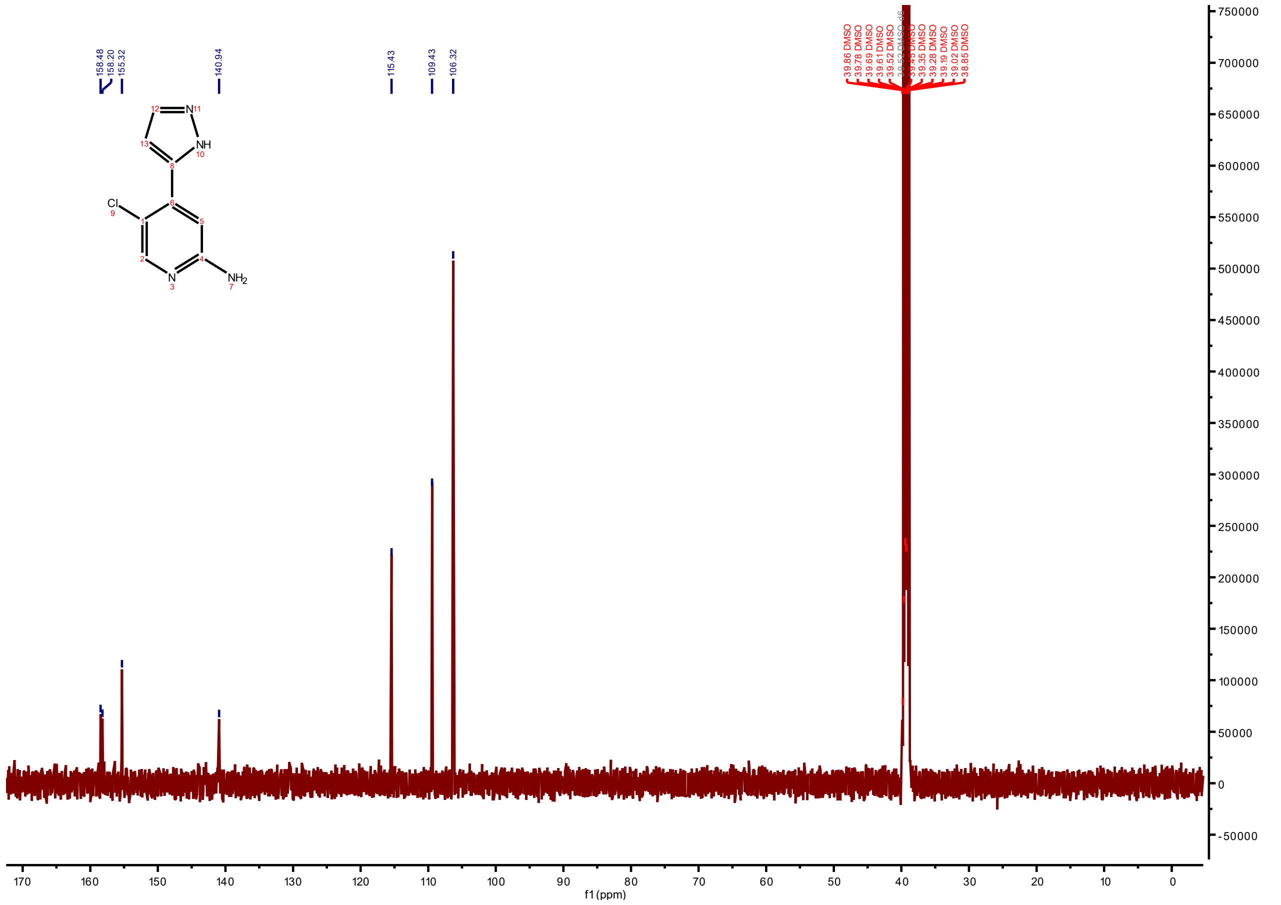

Compound 22

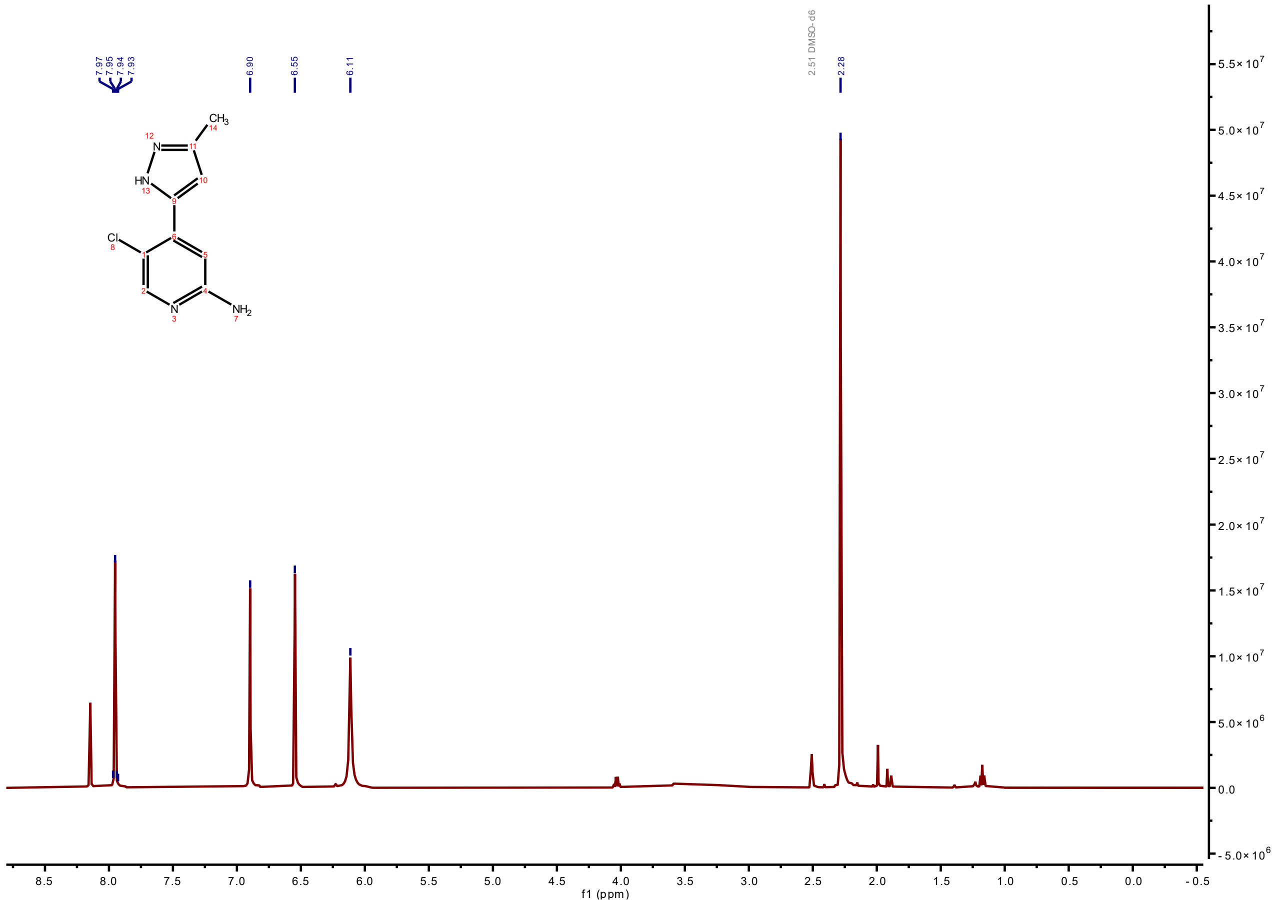

7.97
7.95
7.94
7.93
6.90
6.55
6.11
2.51 DMSO-d6
2.28
f1 (ppm)

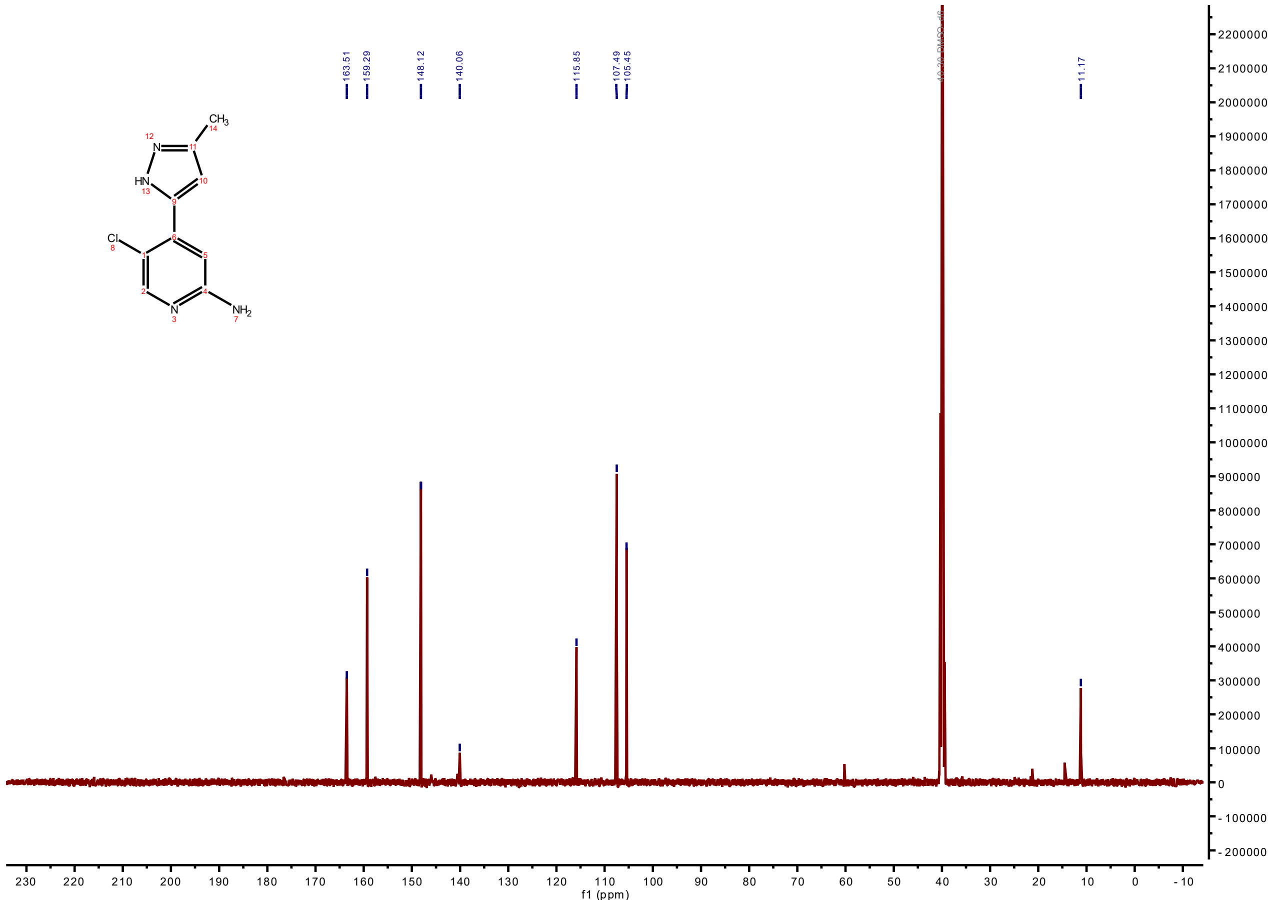

163.51
159.29
148.12
140.06
115.85
107.49
105.45
11.17
f1 (ppm)

HPLC profiles

Compound 10

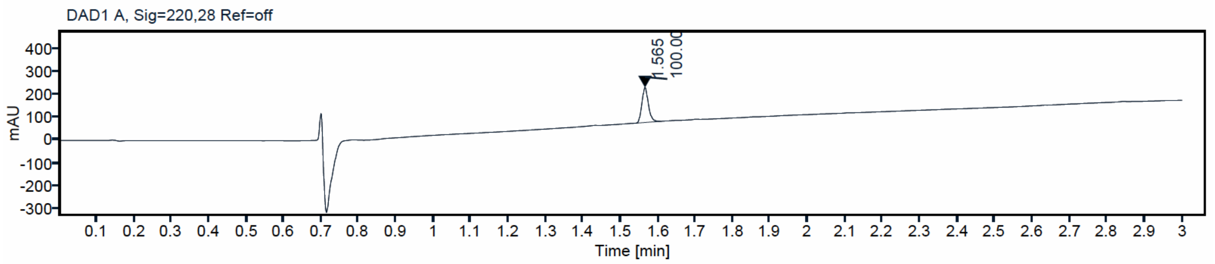

Compound 14a

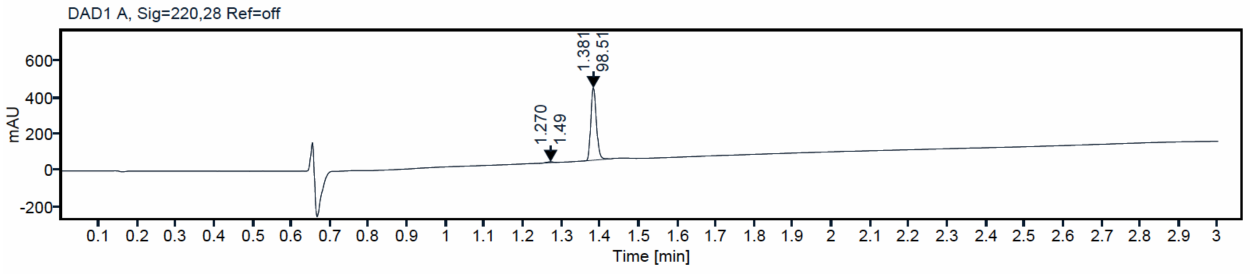

Compound 14b

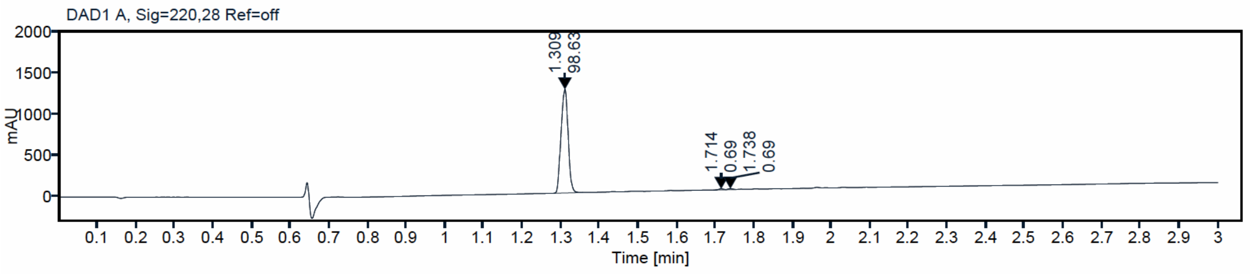

Compound 14c

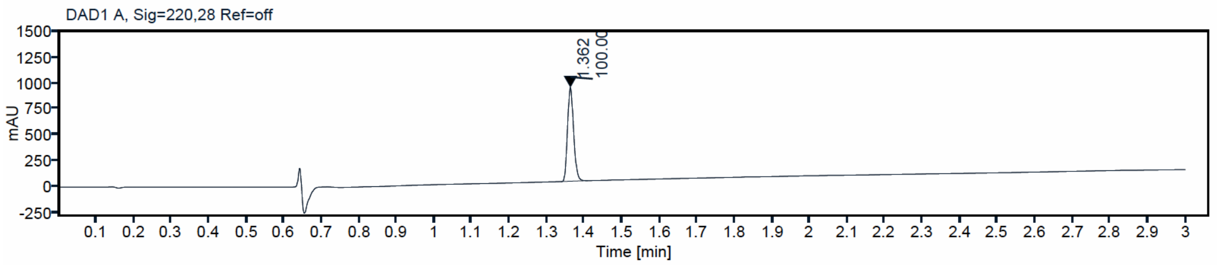

Compound 14d

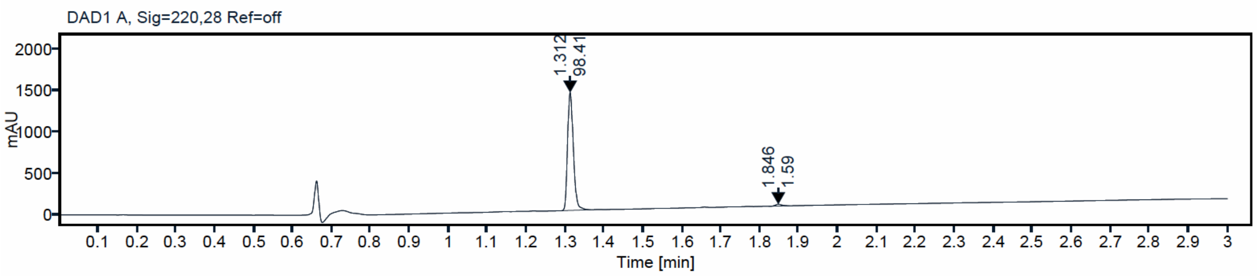

Compound 14e

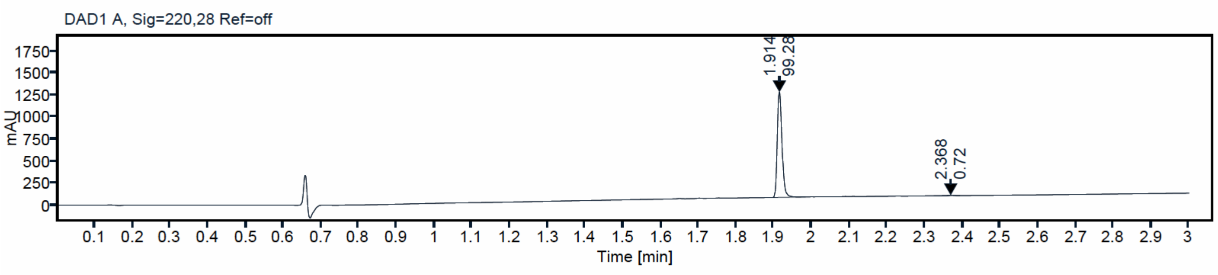

Compound 14f

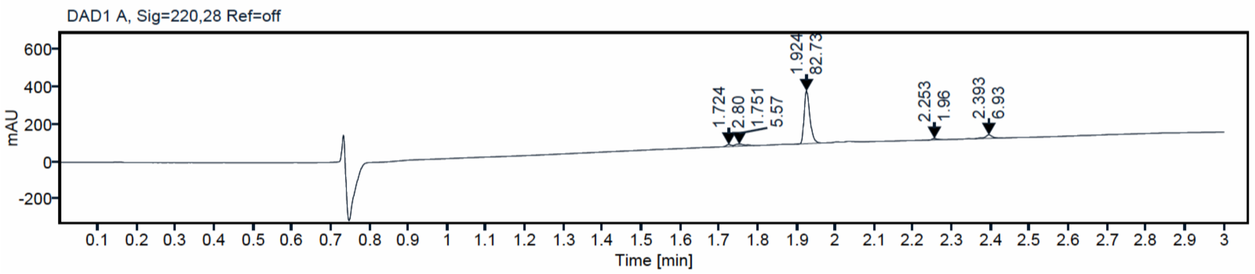

Compound 14g

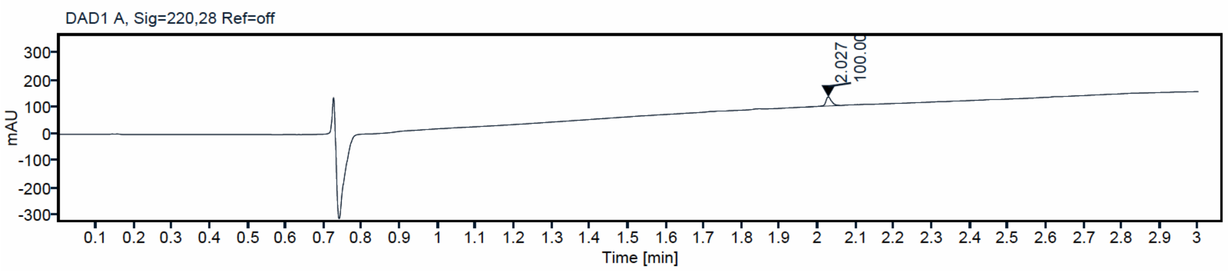

Compound 14h

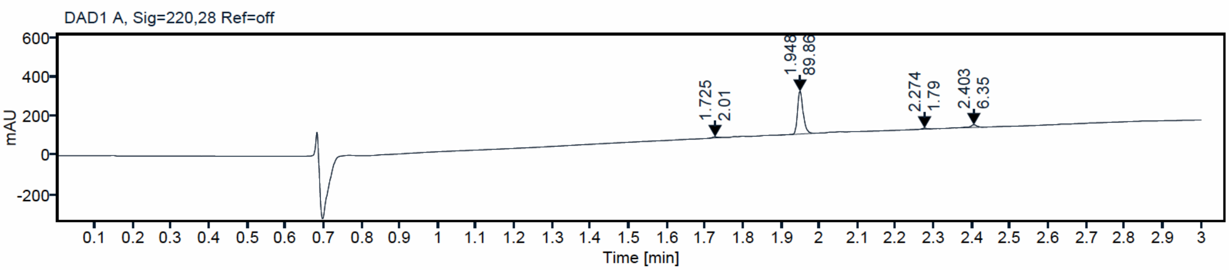

Compound 14i

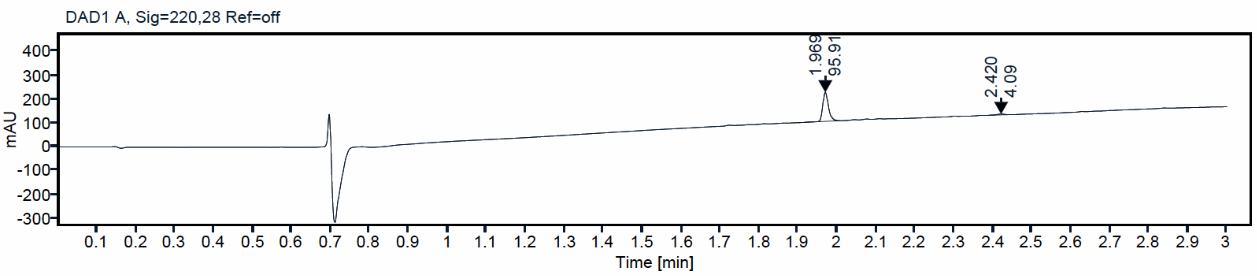

Compound 14j

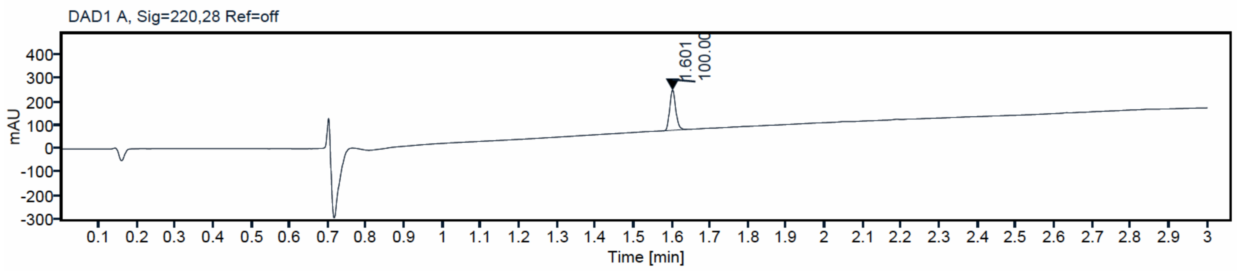

Compound 14k

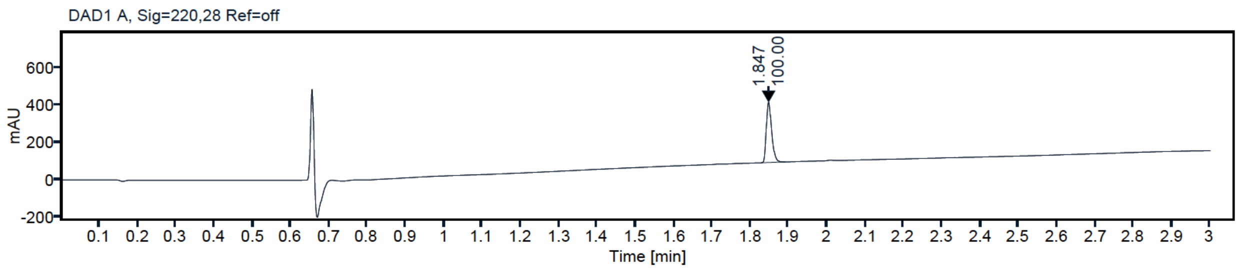

Compound 14l

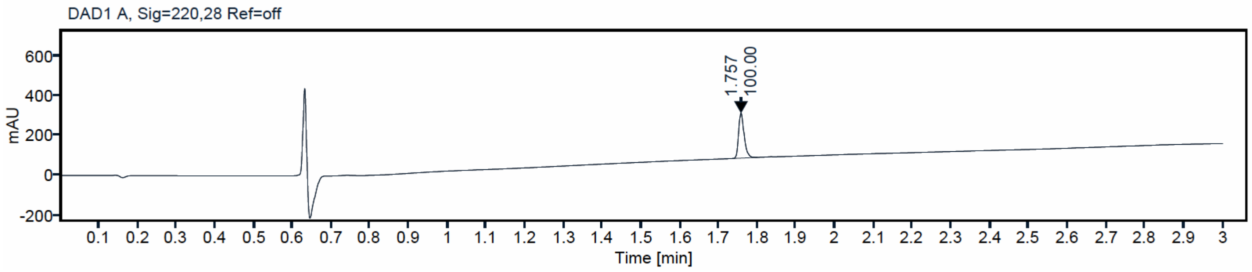

Compound 14m

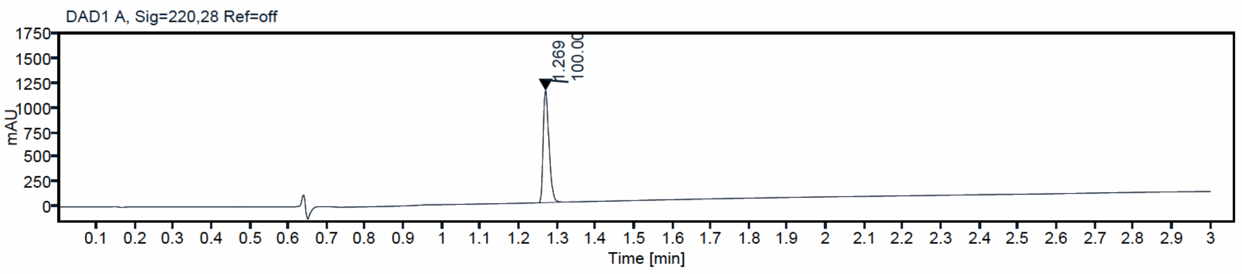

Compound 14n

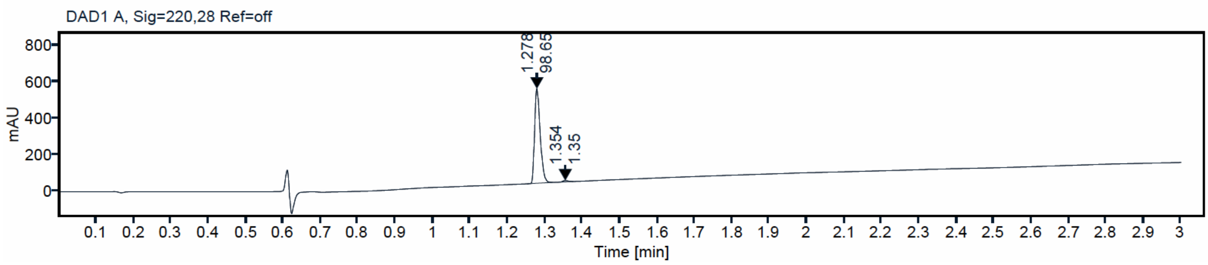

Compound 23

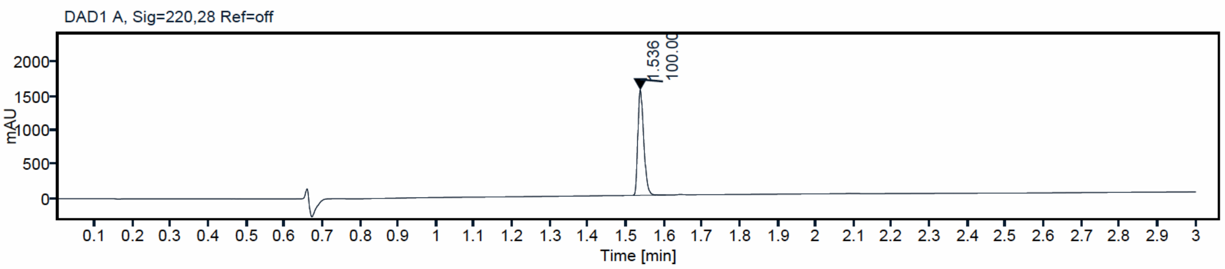

Compound 24

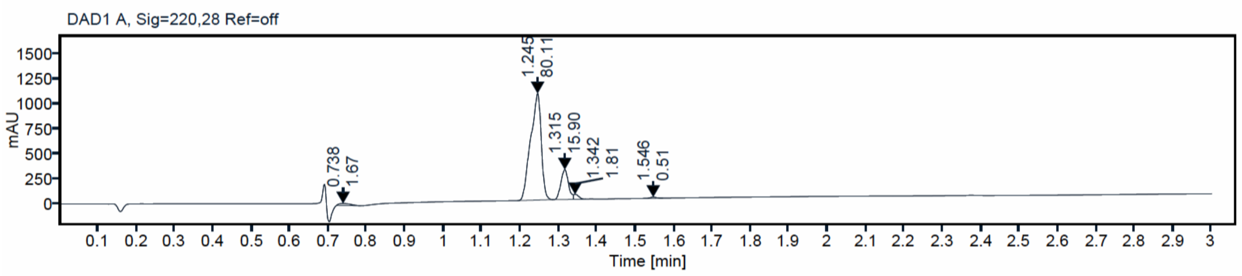

Compound 25

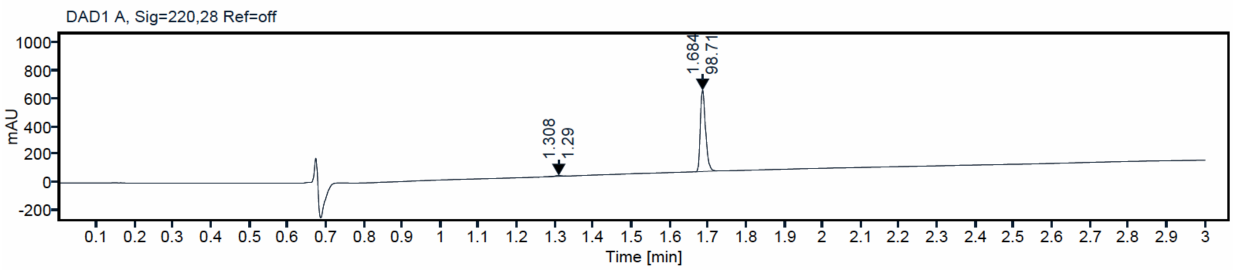

Compound 26

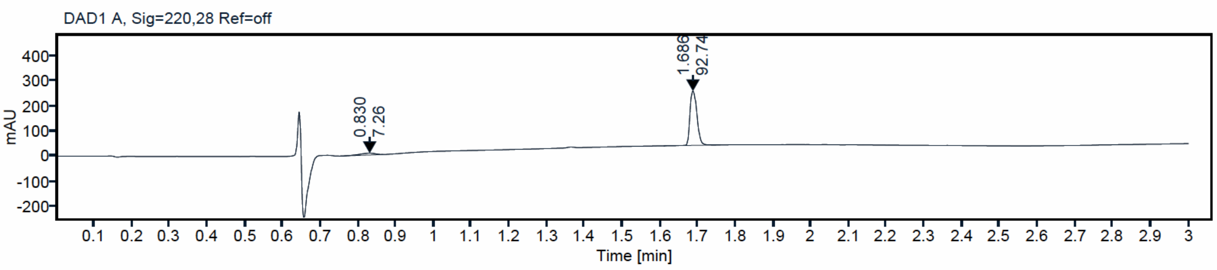

Compound 14b1

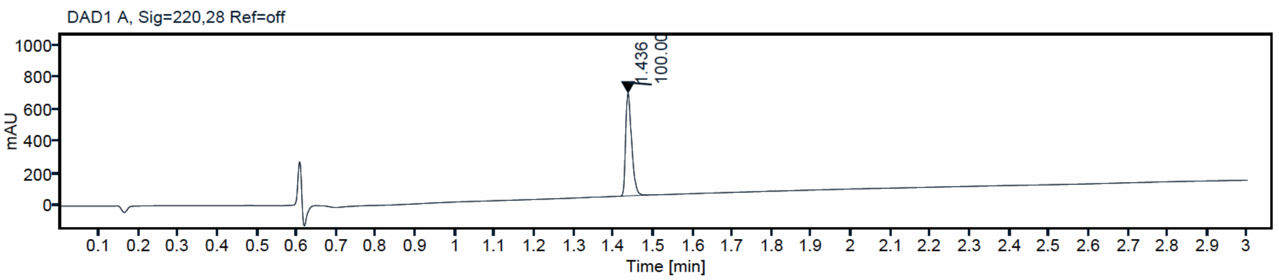

Compound 14b2

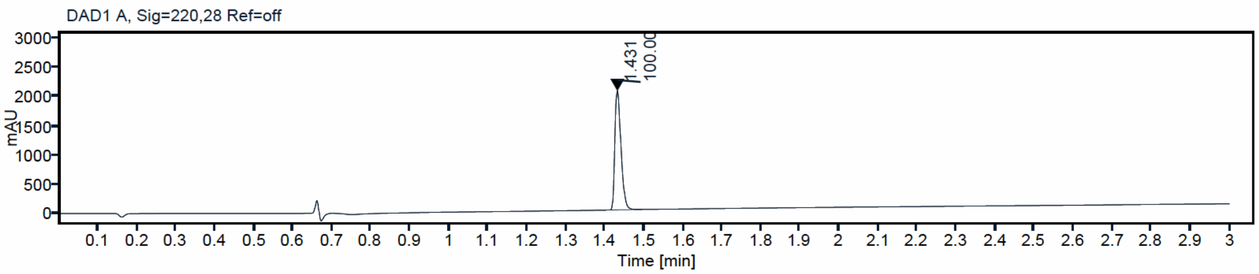

Compound 14b3

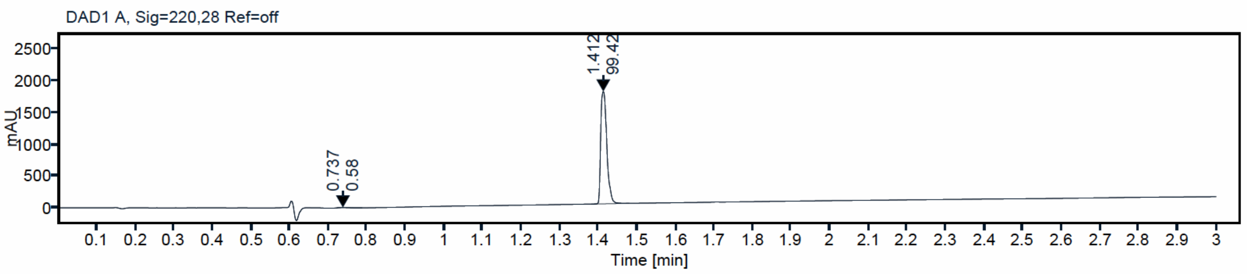

Compound 14b4

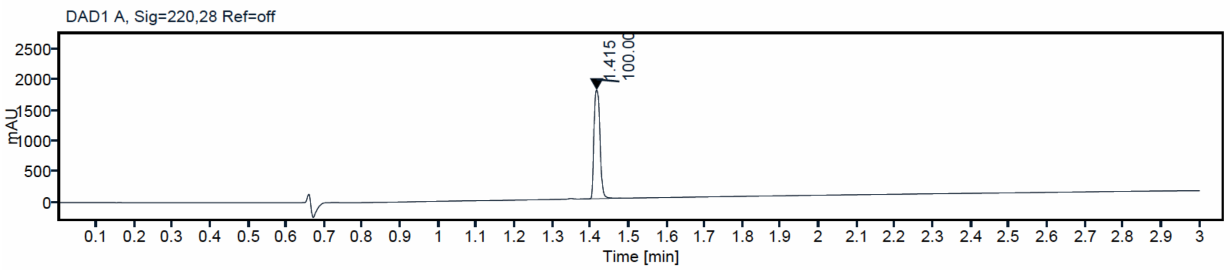

Compound 14c1

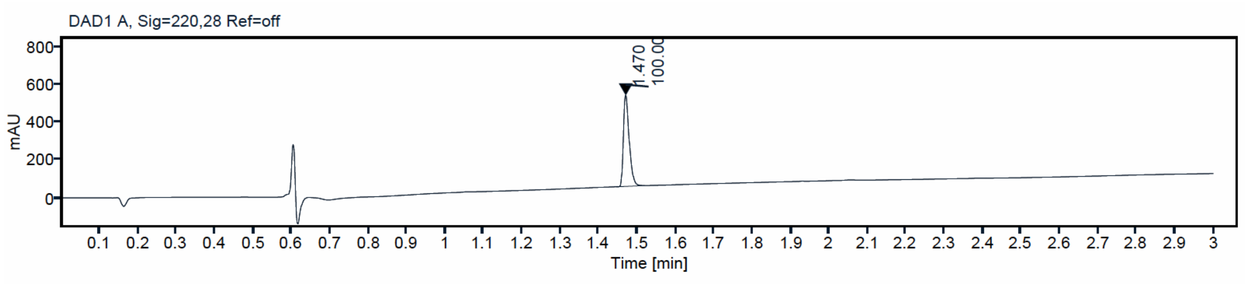

Compound 14c2

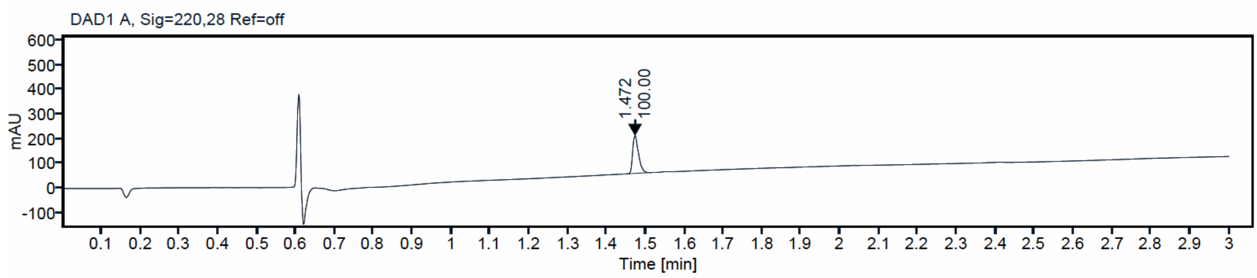

Compound 14c3

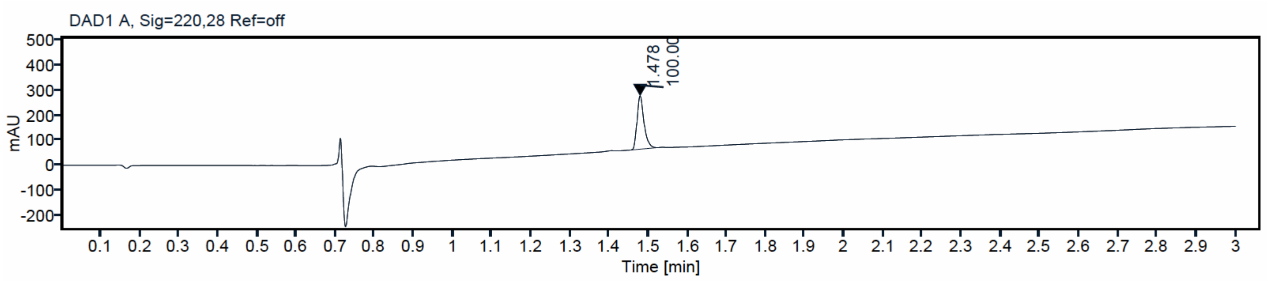

Compound 14c4

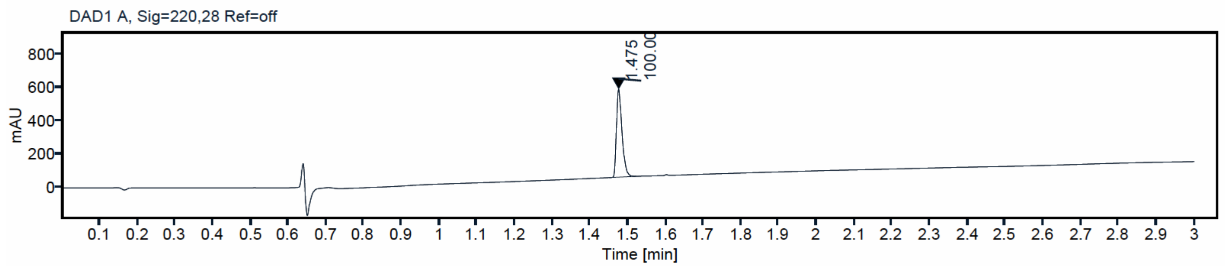

Compound 14c5

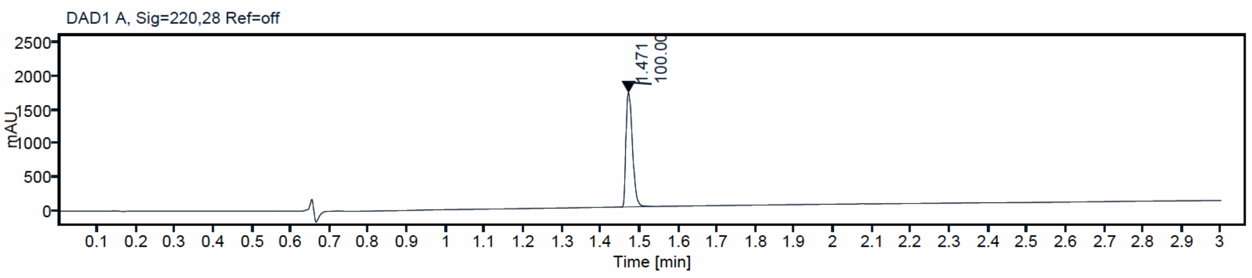

Compound 14c6

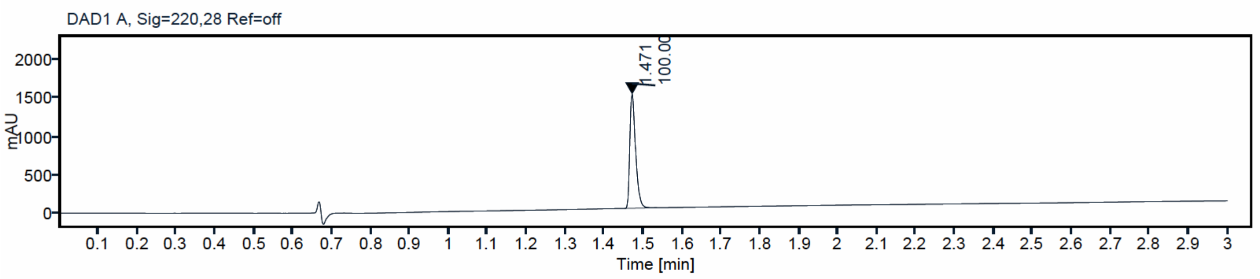

Compound 19

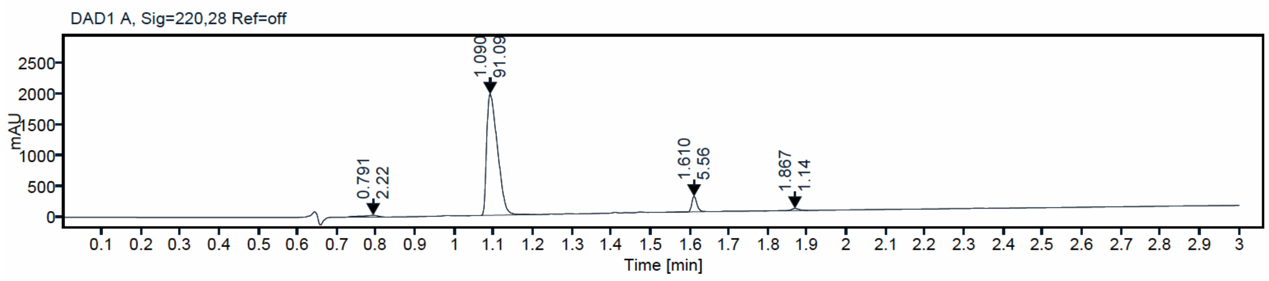

Compound 22

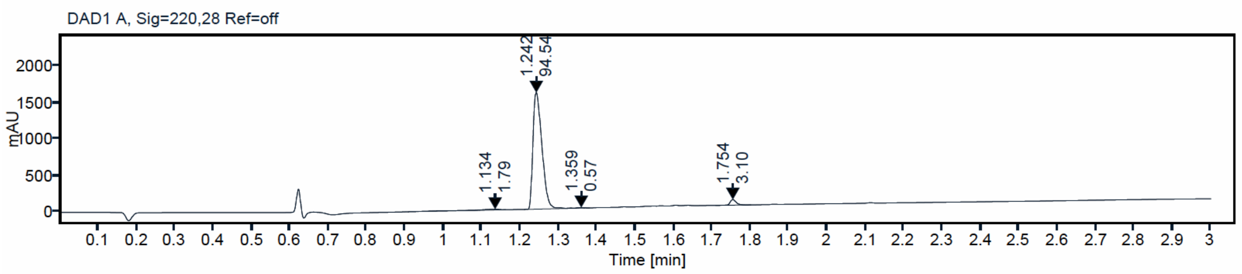

Supplementary references

1. Buffa, V. *et al.* Conformational Plasticity and Binding Affinity Enhancement Controlled by Linker Derivatization in Macrocycles. *Angew. Chemie - Int. Ed.* **64**, e202418512 (2025).
2. Prochnow, H. *et al.* Subcellular Quantification of Uptake in Gram-Negative Bacteria. *Anal. Chem.* **91**, 1863–1872 (2019).
3. Zahorska, E. *et al.* Neutralizing the Impact of the Virulence Factor LecA from Pseudomonas aeruginosa on Human Cells with New Glycomimetic Inhibitors. *Angew. Chemie - Int. Ed.* **62**, (2023).
4. Fedorov, O.; Niesen, F. H.; Knapp, S. Kinase inhibitor selectivity profiling using differential scanning fluorimetry. *Methods Mol Biol* **795**, (2012).
5. Amrhein, J. A. *et al*. Total Synthesis of Pyrazole-Based Macrocycles Leads to a Highly Selective Inhibitor for MST3. *J Med Chem* **67**, 674–690 (2024).
6. Gelin, M. *et al.* Combining 'dry' co-crystallization and in situ diffraction to facilitate ligand screening by X-ray crystallography. *Acta Crystallogr. Sect. D Biol. Crystallogr.* **71**, 1777–1787 (2015).
7. Kowalewski, J. *et al.* Fragment-based drug design of a bacterial kinase inhibitor capable of increasing antibiotic sensitivity of clinical isolates. Under revision.
