# Supplementary material for: Targeting bacterial kinases as a strategy to counteract antibiotic resistance
Source: Commun Chem. 2025 Dec 4;8:390. doi: 10.1038/s42004-025-01794-7 (PMC12678819; doi:10.1038/s42004-025-01794-7)
Supplement: Supplementary file 2 — Description of Additional Supplementary Files [file 42004_2025_1794_MOESM2_ESM.pdf]

Description of Additional Supplementary Files:

File: Supplementary Data 1

Description: raw data used to generate Table 3, Figure 7, Figure 8, Supplementary Figure 4.
